# Structure-Guided Optimization of Novel Inhibitors of *Plasmodium* Lysyl-tRNA Synthetase with Multistage Activity against Malaria Parasites

**DOI:** 10.1021/acs.jmedchem.6c00823

**Published:** 2026-06-02

**Authors:** Barbara Forte, Fiona Bellany, Peter S. Campbell, Giulia Chemi, Alice Dawson, Mark Anderson, Yaw Aniweh, Anna Y. Burkhard, Anna Caroline Campos Aguiar, Alisje Churchyard, Caitlin A. Cooper, Amália dos Santos Ferreira, Mufuliat Toyin Famodimu, Francis G. Fang, Xiao Hu, Tonnie Huijs, Delphine Baud, Chimed Jansen, María Belén Jiménez Díaz, Roger Bonnert, Susan Boyd, Benigno Crespo-Fernández, Branko Mitasev, Simone Montagna, Sachel Mok, Dinakaran Murugesan, Sunil K. Narwal, Neil R. Norcross, John Okombo, Heekuk Park, Caroline Peet, Dhelio B. Pereira, John M. Post, Janette Reader, Jennifer Riley, David A. Robinson, Raku Shinkyo, Frederick R.C. Simeons, Laura Simpson, Alasdair Smith, Dennis Smith, Josefine Striepen, Carolina B.G. Teles, Rianne van der Laak, Anne-Catrin Uhlemann, Amélie Vantaux, Caroline Wilson, Benoît Witkowski, Gavin Wood, Tomas Yeo, Fabio Zuccotto, Iñigo Angulo-Barturen, Jake Baum, Judith M. Bolscher, Rafael Victorio Carvalho Guido, Lyn-Marié Birkholtz, Michael J. Delves, Laurent Dembele, David A. Fidock, Francisco Javier Gamo, Dennis E. Kyle, Steven P. Maher, Jean Popovici, Chris Walpole, Fabian Gusovsky, Paul A. Willis, Kevin D. Read, Ian H. Gilbert, Beatriz Baragaña

**Affiliations:** † Drug Discovery Unit, Division of Biological Chemistry and Drug Discovery, Faculty of Life Sciences, 3042 University of Dundee, Dundee DD1 5EH, U.K.; ‡ West African Centre for Cell Biology of Infectious Pathogens (WACCBIP), College of Basic and Applied Sciences, 58835 University of Ghana, Accra LG54, Ghana; § Department of Microbiology & Immunology, 21611 Columbia University Irving Medical Center, New York, New York 10032, United States; ∥ Center for Malaria Therapeutics and Antimicrobial Resistance, Division of Infectious Diseases, Department of Medicine, 21611 Columbia University Irving Medical Center, New York, New York 10032, United States; ⊥ Department of Microbiology, Immunology, and Parasitology, 28105 Federal University of São Paulo, São Paulo, São Paulo CEP 04023-062, Brazil; # Department of Life Sciences, 4906 Imperial College, London SW7 2AZ, U.K.; ∇ Center for Tropical and Emerging Global Diseases, 1355 University of Georgia, Athens, Georgia 30602-0002, United States; ○ Oswaldo Cruz Foundation, 643873 Leishmaniasis and Malaria Bioassay Platform, Porto Velho, Rondônia CEP 76812-245, Brazil; ◆ Department of Infection Biology, London School of Hygiene and Tropical Medicine, London WC1E 7HT, U.K.; ¶ 42372 Eisai, Inc., 35 Cambridge Park Drive Suite 200, Cambridge, Massachusetts 02140, United States; ▶ 561119 TropIQ Health Sciences, Nijmegen 6534 AT, The Netherlands; ▷ 127356 MMV Medicines for Malaria Venture, ICC, Geneva 1215, Switzerland; ◀ The Art of Discovery, Derio 48160, Spain; ◁ Global Health Medicines R&D, GSK, Tres Cantos, Madrid 28760, Spain; ⧨ Division of Infectious Diseases, Department of Medicine, Columbia University Irving Medical Center, New York, New York 10032, United States; ⧩ Research Center in Tropical Medicine of Rondônia, Porto Velho, Rondônia CEP 76812-329, Brazil; ◭ Department of Biochemistry, Genetics and Microbiology, 56410 Institute for Sustainable Malaria Control University of Pretoria, Hatfield, Pretoria 0028, South Africa; ◮ Malaria Molecular Epidemiology Unit, Institut Pasteur du Cambodge, Phnom Penh 120210, Cambodia; ◬ São Carlos Institute of Physics, University of São Paulo, São Carlos, São Paulo CEP 13563-120, Brazil; ▼ Department of Biochemistry, Stellenbosch University, Stellenbosch, Matieland 7602, South Africa; ▽ 225803 Univerité Des Sciences, Des Techniques Et Des Technologies de Bamako (USTTB), Parasite and Microbe Research and Training Centre (P-MRTC), Faculty of Pharmacy, Point G, Bamako BP 1805, Mali; ▲ Malaria Research Unit, 533891 Institut Pasteur du Cambodge, Phnom Penh 120210, Cambodia; △ Infectious Disease Epidemiology and Analytics G5 Unit, Institut Pasteur, Université Paris Cité, Paris 75015, France; ⟁ Structural Genomics Consortium, 105607 Research Institute of the McGill University Health Centre, Montreal, Québec H4A 3J1, Canada

## Abstract

A fused dihydropyrrolidino-pyrimidine hit with low lipophilicity and excellent ligand efficiency was identified in a biochemical screen of the Global Health Chemical Diversity Library (GHCDL) against *Plasmodium* lysyl-tRNA synthetase (KRS). Structure-guided lead optimization delivered analogues with potent parasite growth inhibition, excellent biochemical and cellular selectivity (>1000-fold), and oral efficacy in the malaria NOD-scid-IL2Rγ^null^ (SCID) mouse model. Structural information and computational methods were deployed to identify a potent and selective basic KRS inhibitor (**30**) with an extended half-life to reduce the dose regimen to a single-dose cure. Compound **30** displayed a long half-life across preclinical species, favorable safety, and activity across *Plasmodium* species as well as against drug-resistant and sensitive *P. falciparum* strains and field isolates. Unfortunately, **30** lacked oral bioavailability, which could not be mitigated with a prodrug approach. Nevertheless, learnings from this series will assist future KRS programs in delivering a clinical candidate with this novel mode of action.

## Graphical abstract

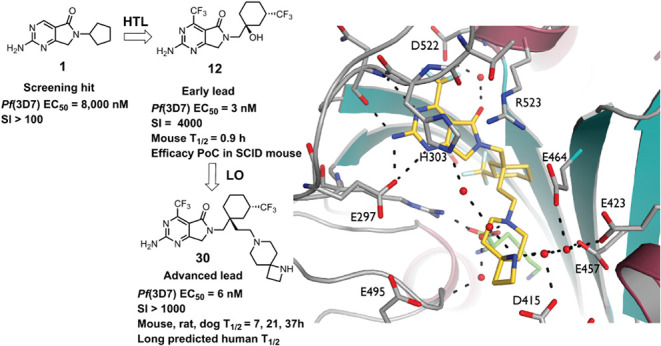

## Introduction

Malaria is a devastating infection that affected 263 million people worldwide and led to 610,000 deaths in 2024.^1^ It is caused by protozoan *Plasmodium* parasites transmitted by *Anopheles* mosquitoes. Five species of *Plasmodium* parasites cause human disease: *P. falciparum*, *P. vivax*, *P. ovale*, *P. malariae*, and *P. knowlesi*. Of these, *P. falciparum* is associated with most deaths, although infections due to *P. vivax* and *P. knowlesi* can cause severe disease. Infection with *P. vivax* and *P. ovale* is associated with dormant liver-stage forms (hypnozoites) that can be activated months to years after the primary infection, leading to relapse and recurring disease.

Malaria remains a major threat to human health, imposing a heavy social and economic burden, particularly in Africa. Children under five and pregnant women are at higher risk of severe malaria that can lead to death. While there has been a 50% reduction in mortality from 2000 to 2015, the rate of reduction has slowed down to just 3% between 2015 and 2022. The reasons for this slower reduction rate are complex, including disruptions to services during the COVID-19 pandemic, a changing climate, conflict and humanitarian crises, and resistance to drugs and insecticides.^1^ Drug resistance is of particular concern as partial resistance to artemisinin, the core component of first-line artemisinin-based combination therapies (ACTs), is present in sub-Saharan Africa and in the Greater Mekong subregion.^2^ Thus, the identification of new drugs with differentiated modes of action is a constant and urgent need. Multiple new types of antimalarials are needed: (a) for treatment of asexual blood stage disease; (b) for chemoprevention for those vulnerable to infection or asymptomatic; (c) to prevent transmission; and (d) to prevent relapse. Medicines for Malaria Venture (MMV) has detailed target product profiles (TTPs) and target candidate profiles (TCPs) to direct the drug discovery process.^3,4^ One of the most challenging aspects in current antimalarial drug development is the long half-life required to deliver candidates with potential for single-dose cure of malaria infections (TCP1, blood stage killer) to replace the current three-days dose treatment with ACTs.^3^

In the past decade, *Plasmodium* aminoacyl-tRNA synthetases have received increased attention as new targets for antimalarial drug discovery.^5–11^ Malaria parasite genomes encode two different lysyl-tRNA synthetases that play a role in translation in either the cytoplasm (*Pf*KRS1) or in the apicoplast (*Pf*KRS2), while humans encode one copy.^12^ Human KRS (*Hs*KRS) is found in both the cytosol and mitochondrion and has additional roles within human cells. Cladosporin, a fungal metabolite, inhibits *Pf*KRS1 with nanomolar potency and is selective against the human isoform.^13–15^ However, it exhibits low metabolic stability, and synthetic analogues have failed to progress to efficacy studies due to the absence of sufficiently improved *in vitro* ADME profiles.^15,16^ To identify novel lead-like inhibitors for this target we carried out biochemical screens of several compound libraries. We previously reported a series of chromones as potent and selective inhibitors of *Pf*KRS1 and the structure-guided optimization to achieve *in vivo* proof of concept of efficacy in a SCID mouse model of malaria.^17^ Overall, *Pf*KRS1 stands out among *Plasmodium* aminoacyl-tRNA synthetases, supported by robust genetic and chemical validation both *in vitro* and *in vivo*.

Herein, we report the discovery and optimization of a novel series of drug-like inhibitors of *Pf*KRS1. Assisted by multiple costructures, we optimized a low micromolar HTS hit to single-digit nanomolar leads and developed strategies to achieve >1,000-fold selectivity for *Pf*KRS1 over the human enzyme. We demonstrated that inhibitors of *Pf*KRS1 have multistage antiplasmodial activity, retain activity against field isolates, and have a moderate rate of kill and low risk of resistance *in vitro*. Our optimized neutral leads are potent and selective antimalarials with oral bioavailability and efficacy in the SCID mouse model of malaria, but have a short *in vivo* half-life. We also designed potent and selective basic *Pf*KRS1 inhibitors with an extended half-life and favorable safety profile but with poor oral bioavailability. Finally, *in vitro* evolution of compound resistance followed by whole genome sequencing supports that our lead compound kills the parasite through inhibition of *Pf*KRS1.

## Results and Discussion

### Hit Identification and Hit-to-Lead Phase

A biochemical screen of the Global Health Chemical Diversity Library (approximately 70,000 compounds) against *Pf*KRS1 led to the identification of a fused dihydropyrrolidino-pyrimidine hit 1 (Table 1 and Figure 1A). Compound **1** was an attractive hit for a drug discovery program due to its low molecular weight and low lipophilicity resulting in excellent ligand efficiency (LE 0.4, LLE 4.9). Following a pathogen hop strategy, compound **1** served as starting point for parallel optimization for three different infectious diseases of interest for the Drug Discovery Unit: tuberculosis, cryptosporidiosis, and malaria. We have previously reported our results for tuberculosis^18^ and cryptosporidiosis^19^ and here we describe the optimization of this series for malaria.

**Figure 1:**
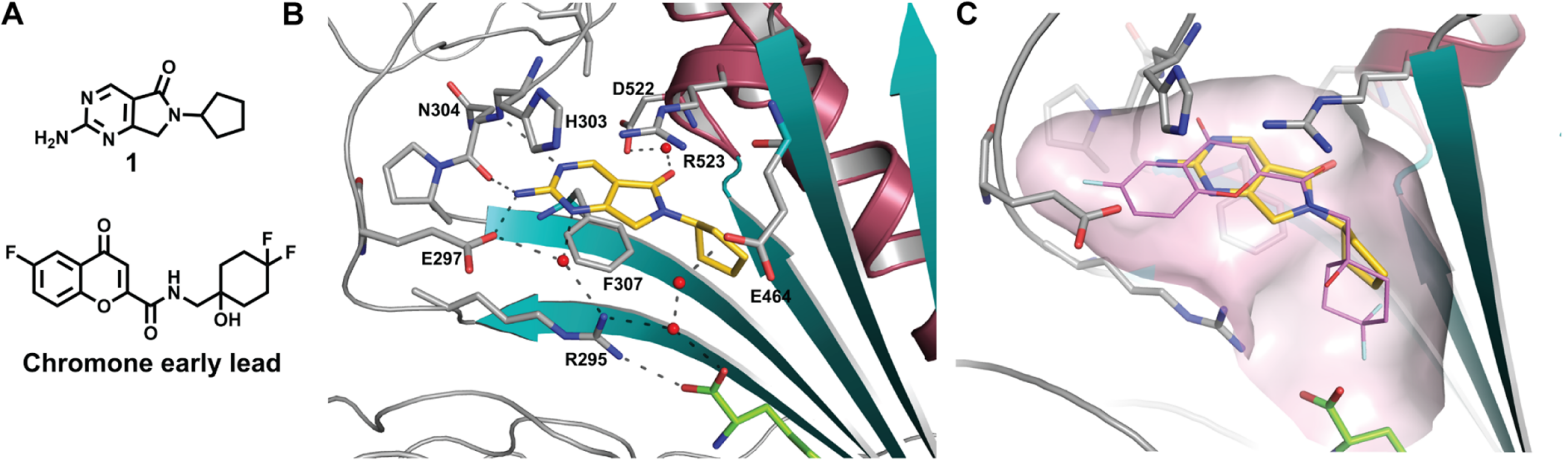
(a) Chemical structure of aminopyrimidine **1** and a previously reported chromone early lead.^17^ b) Co-structure of *Cryptosporidium parvum* KRS (*Cp*KRS) bound to lysine (green) and **1** (yellow) (PDB ID code 9r2c). Compound **1** binds to this enzyme’s ATP binding site. c) Overlay of costructures of *Cp*KRS bound to compound **1** (yellow) and chromone early lead (pink) (PDB ID codes 9r2c and 6hcw, respectively). Figures were prepared using PyMol: The PyMOL Molecular Graphics System, Version 2.5.5 Schrödinger, LLC.

**Table 1:**
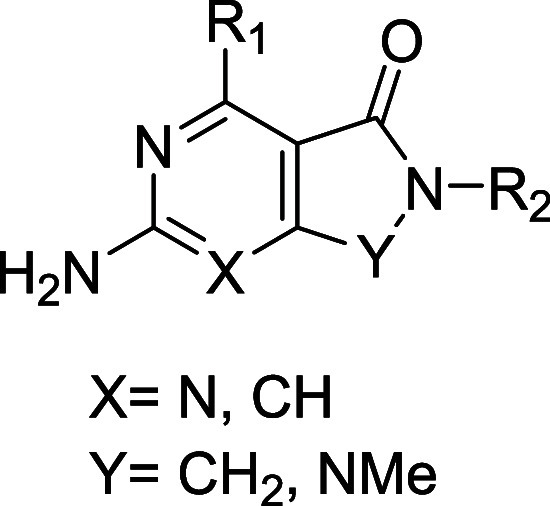

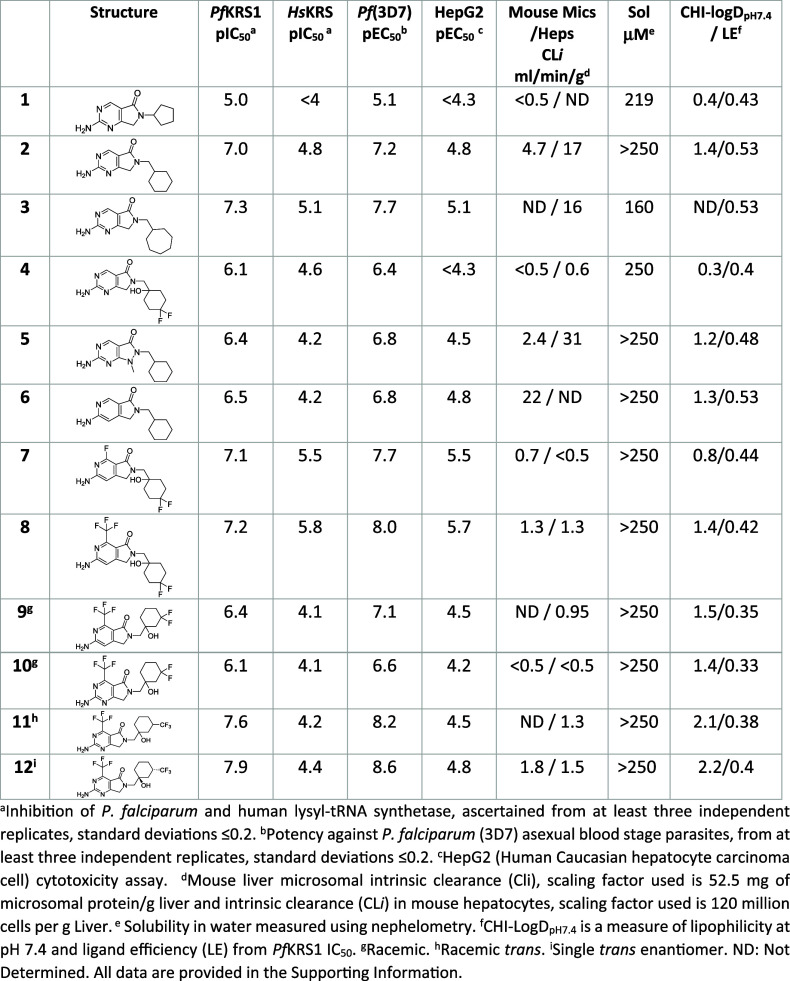
Hit-to-Lead Structure–Activity Relationships (SAR)

Although obtaining high-resolution structures of *Pf*KRS1 is possible, our attempts to generate costructures using high-throughput crystallization techniques were unsuccessful with this protein. We therefore developed an alternative method of soaking inhibitors in cocrystals of *Cryptosporidium parvum* KRS (*Cp*KRS) and lysine, which gave us access to rapid structure determination to support the drug discovery program.^17^ *Cp*KRS and *Pf*KRS1 differ in the ATP binding site by only 3 amino acids (P272T, N293V, A309S). As we previously described, we mutated the *Cp*KRS active site to incorporate these 3 different residues present in *Pf*KRS1, thus generating a “soakable” *Cp-Pf*KRS chimeric protein with all the residues in the active site identical to *Pf*KRS1.^20^ Optimization of compound **1** was supported by multiple costructures of analogues with either *Cp*KRS or the *Cp-Pf*KRS chimeric protein. For consistency with the use of *C. parvum* KRS structures all residue numbers quoted here refer to *Cp*KRS instead of *Pf*KRS1 unless explicitly indicated.

At the start of the project a crystal structure of **1** bound to *Cp*KRS showed that **1** binds in the ATP binding site of KRS (Figure 1B). The amino-pyrimidine core stacks between the side chain of F307 on one face and the side chains of H303, and R523 on the other. The pyrimidine nitrogen forms an H-bond to the backbone NH of N304, mimicking the N1 of adenine, and the NH_2_ makes a H-bond with the E297 side chain and the P305 backbone carbonyl. The lactam carbonyl H-bonds to a structural water molecule. The cyclopentyl moiety projects into a pocket pointing toward the substrate lysine (in green in Figure 1B). Compound **1** binds in the same pocket as our previously reported chromone series.^17^ The overlay of crystal structures for compound **1** and chromone early lead showed that the cyclopentyl does not fill the hydrophobic pocket delimited by the lysine substrate and the protein (Figure 1C). This prompted us to start the optimization of our hit by replacing the cyclopentyl substituent for larger cycloalkyls with a methylene linker to the lactam nitrogen. Encouragingly, compounds **2** and **3** led to 100-fold increased enzyme and parasite inhibition and retained excellent ligand efficiency (LE 0.53). However, both compounds suffered from poor metabolic stability (Table 1).

Initially, the hit-to-lead phase focused on improving metabolic stability while retaining potency with modifications both on the cyclohexyl moiety and the scaffold. Transferring learnings from the chromone series, we introduced a hydroxyl substituent on C1 and geminal fluorine atoms on C4 of the cyclohexyl ring.^17^ Compound **4** displayed a significant improvement in *in vitro* metabolic stability in mouse microsomes and hepatocytes combined with a small reduction in potency (10-fold) (Table 1). However, despite the favorable *in vitro* metabolic stability, in a mouse oral pharmacokinetic study blood concentration remained above *in vitro* antimalarial potency for insufficient time to justify the progression of **4** to an *in vivo* mouse efficacy study (Figure S1 and Table S2). Met-ID analysis of urine samples from this mouse pharmacokinetic study showed a major metabolite resulting from the oxidation of the scaffold. However, we were unable to determine whether the pyrimidine or the lactam ring was oxidized.

Thus, we replaced the CH_2_ on the lactam for NMe (**5**) to block a possible metabolic hotspot. This modification was well tolerated resulting in a modest improvement in mouse microsomal clearance (MLM CLint 4.7 for compound **2** vs 2.4 mL/min/g compound **5**) but mouse hepatocyte clearance remained high. With this result, we hypothesized that aldehyde oxidase may be mediating the oxidation of the unsubstituted pyrimidine carbon.

Parallel to the modification of the cyclohexyl ring and guided by the structural information, we prepared an aminopyridine analogue (**6**) by removing the nitrogen on the pyrimidine ring not directly interacting in the active site. As expected, aminopyridine **6** and its matched pair aminopyrimidine **2** were equipotent and displayed similar *in vitro* DMPK (drug metabolism and pharmacokinetics) properties (Table 1).

To block the potential aldehyde oxidase metabolism, we introduced a fluorine atom on the carbon adjacent to the N of the aminopyridine ring (**7**, Table 1). The introduction of this substitution resulted in an improvement in both potency and metabolic stability. The F atom occupies a small buried lipophilic pocket that can accommodate small substituents, thus explaining the improvement in potency. A further enhancement in potency was obtained with compound **8**, bearing a CF_3_ instead of a F (Table 1). The introduction of a CF_3_ at this position induced the rotation of I534 side chain increasing the volume of this subpocket to accommodate a larger substitution (Figure 2). Compounds **7** and **8** were stable in mouse microsomes and hepatocytes and retained good solubility (Table 1).

**Figure 2:**
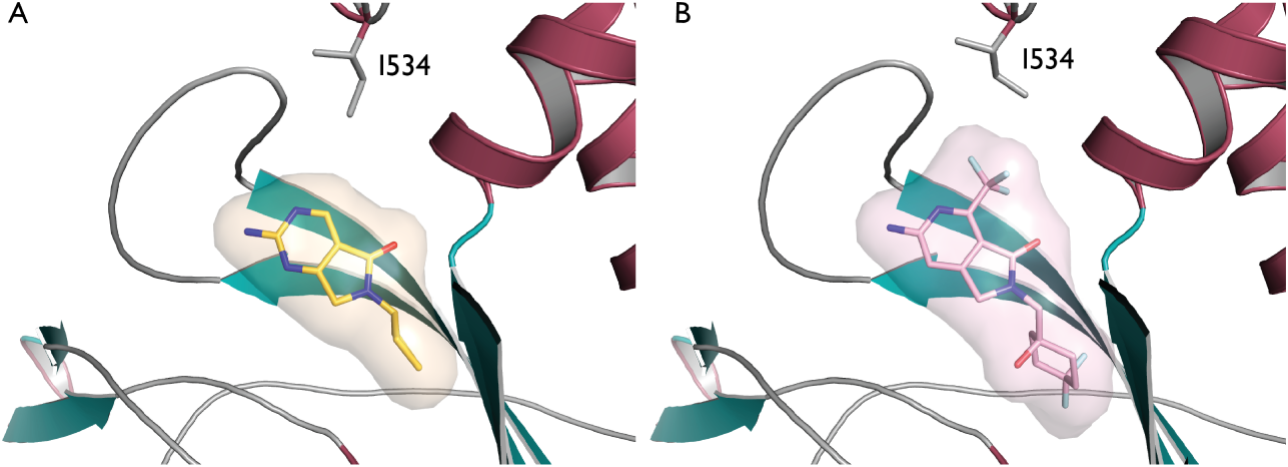
(a) Co-structure of *Cp*KRS bound to **1** (yellow) (PDB ID code 9r2C). b) Co-structure of *Cp*KRS bound to **8** (pink) (PDB ID code 9r3r). Both panels show the surface of the ligand and the position of the I534 side chain. Figures were prepared using PyMol: The PyMOL Molecular Graphics System, Version 2.5.5 Schrödinger, LLC.

Compounds **7** and **8** were selected for safety profiling. The *in vitro* selectivity for both compounds was excellent against hERG (hERG-CHO, automated patch-clamp, IC_50_ > 100 μM) as well as against human CYP3A4, CYP2D6, CYP2C9, CYP2C19 and CYP1A2 (**7**: pIC_50_ 4, <4, 4.3, 4.5 and <4; **8**: pIC_50_ 4.1, <4, 4.8, 4.8 and <4, respectively). Compounds **7** or **8** did not display significant inhibition (>50%) when screened in a panel of 44 enzymes, ion channels, and receptors (Eurofins SafetyScreen 44 panel) at 10 μM (Tables S3 and S4). Evaluation of compound **8** in the Dundee panel of 140 human kinases revealed a clean kinome profile with enzymatic activity remaining above 50% for all kinases in the panel at 10 μM (Table S5). With these positive results the only recognized safety concern for these two molecules was the low micromolar inhibition of human KRS (**7**: pIC_50_ 5.5; **8**: pIC_50_ 5.8) and the cytotoxicity (HepG2 **7**: pIC_50_ 5.5; **8**: pIC_50_ 5.7).

Therefore, we focused on improving selectivity by targeting differences in amino acids between *Pf*KRS1 and *Hs*KRS binding pockets, particularly focusing on the region where the cyclohexyl group binds. Assisted by compound modeling we selected C-3 on the cyclohexyl to introduce small substituents designed to clash with T337 in *Hs*KRS and that should be well accommodated by *Pf*KRS1 which contains a smaller serine residue (S344) (Figure 3). Thus, moving the gem-difluoro substituent from C4 of cyclohexyl to C3 position (**9** and **10**) resulted in a 10-fold decrease in *Pf*KRS1 inhibition, but a 100-fold improvement in selectivity compared with **8**. As in previous examples, pyridine **9** and pyrimidine **10** displayed similar potency, solubility, and permeability. Pyrimidine **10** had excellent metabolical stability in hepatocytes across species (Heps CLi < 0.5 mL/min/g for mouse, rat, dog and human) and was selected for further profiling. The replacement of the gem-diF for a CF_3_ substituent *trans* to the OH (**11**) led to a 30-fold improvement in enzyme inhibition. All four possible enantiomers were prepared with one of the *trans* enantiomers (**12**) being the eutomer (Table 2). Compound **12** absolute stereochemistry was unambiguously assigned from a costructure of *Cp*KRS bound to **12** (Figure S2). The CF_3_ in compound **12** clashes with T337 in *Hs*KRS increasing selectivity and it fills the larger lipophilic pocket in *Pf*KRS1 improving potency. In addition, in the *trans* configuration, the OH establishes a water-mediated interaction with E464 (Figure 3). Pleasingly, **12** was a potent inhibitor of parasite growth (pEC_50_= 8.6) with excellent selectivity against mammalian cells (>1,000 fold), low microsomal and hepatocyte clearance, and moderate solubility and permeability (PAMPA Pe = 71 nm/s).

**Figure 3:**
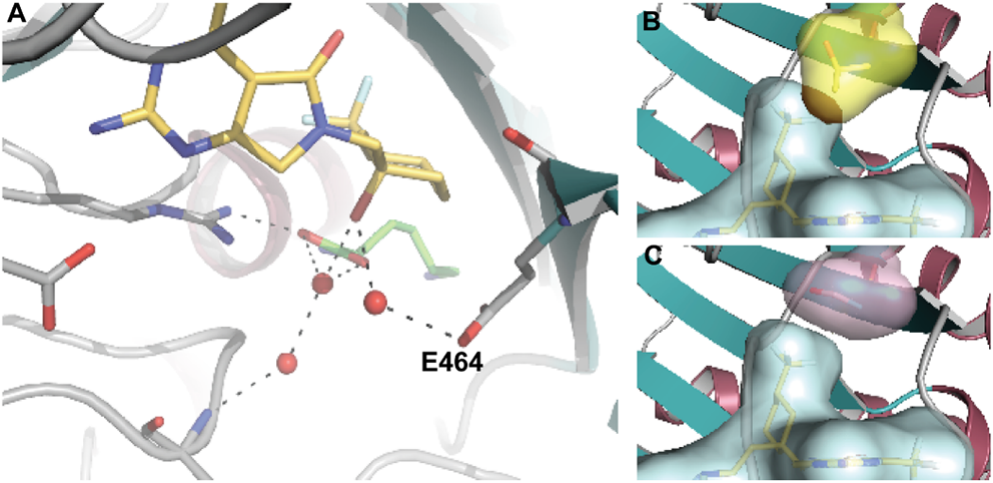
(a) Interactions formed by **12** with the water network (red spheres) to E464 (PDB ID code 9r32). (b) Shows the clash between the CF_3_ and T337 (yellow surface) by superimposing the ligand in *Hs*KRS (PDB ID code 6chd). (c) Space for S344 (pink surface) in *CpPf*KRS1 (PDB ID code 9r3g). Figures were prepared using PyMol: The PyMOL Molecular Graphics System, Version 2.5.5 Schrödinger, LLC.

**Table 2:**
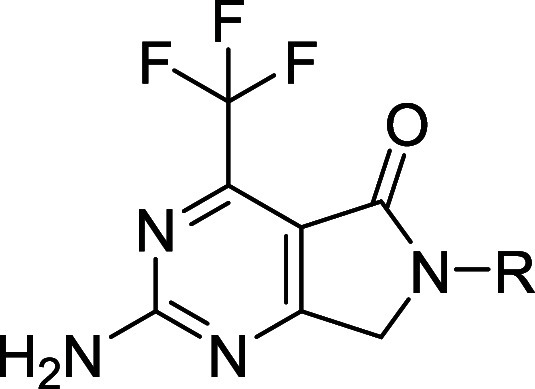

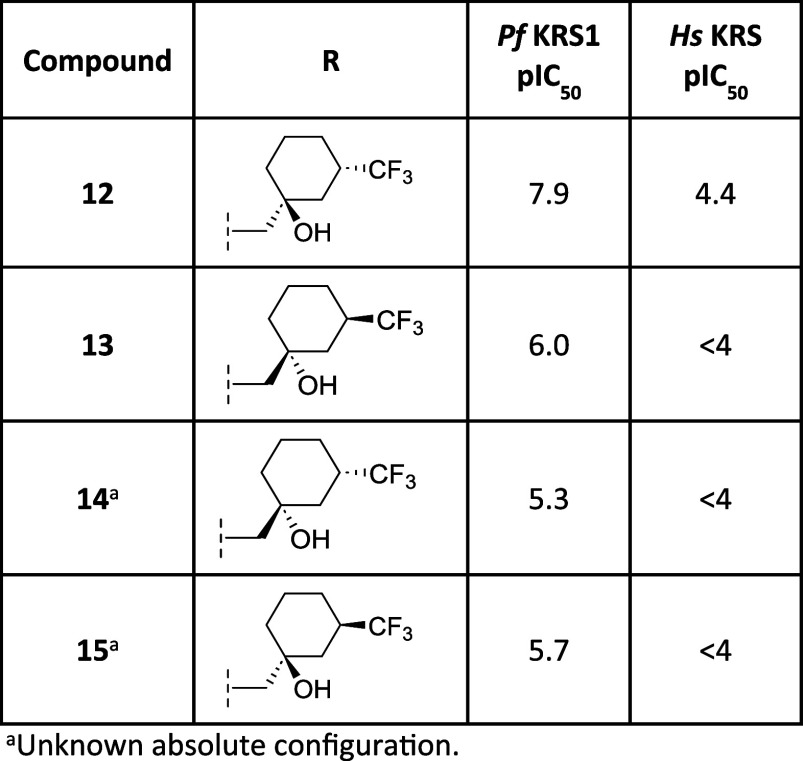
Biochemical Inhibition of *P. falciparum* and Human KRS for Separated Enantiomers **12–15**

Next, we evaluated **10** for hERG inhibition (IC_50_ > 100 μM) and **10** and **12** for inhibition of human CYP3A4, CYP2D6, CYP2C9, CYP2C19 and CYP1A2 (**10**: pIC_50_ < 4 for all; **12**: pIC_50_ < 4.0, 5.1, 6.2, 5.4 and <4). In mouse intravenous and oral pharmacokinetic studies, **10** and **12** showed low *in vivo* unbound clearance (8 and 31 mL/min/g respectively), moderate unbound volume of distribution (1.1 and 2.2 L/kg respectively) and moderate to low bioavailability (F: 49% and 12% respectively) (Table 3). For both compounds, free blood concentration remained above the *P. falciparum* asexual blood stage EC_90_ between 6 to 8 h following a single oral dose of 10 mg/kg (Figure S3 and Table S6).

**Table 3:** Mouse Pharmacokinetic Data for Hit-to-Lead Compounds

Compound	10^a^		12^b^	
Route	IV	PO	IV	PO
Dose (mg/kg)	3	10	3.1	10
C_max_ (ng/mL)		4397		628
T_max_ (h)		1		1
T_1/2_ (h)	1.6		0.9	
AUC_0–8 h_ (ng-min/mL)	480682	781845	219890	87015
Vdss (L/kg)	0.8		1	
Vdss,u (L/kg)	1.1		2.2	
Clb (mL/min/kg)	6		14	
Clu (mL/min/kg)^c^	8.4		31.4	
F (%)		49		12

a Compound **10:** mouse PPB fu = 0.715.

b Compound **12:** mouse PPB fu = 0.446.

c For the purpose of calculating unbound plasma clearances a blood to plasma (B/P) ratio = 1 was assumed.

Despite **12** showing poor bioavailability and a risk for drug–drug interactions (DDI) due to its submicromolar inhibition of CYP2C9, we decided to still progress this compound to *in vivo* efficacy studies, with the aim of obtaining proof of concept of efficacy for the series to support the transition to lead optimization. We intended to address the DDI risk during the lead optimization phase. We selected compound **12** instead of **10** to progress to an efficacy study due to its improved potency (100-fold) while maintaining similar coverage above EC_90_ after oral dosing (Figure S3). Thus, **12** was evaluated in the malaria SCID mouse model at three oral doses (3, 10, and 30 mg/kg QD × 4 days). All dosing regimens resulted in reduced parasitemias compared with the untreated control. At the highest dose, **12** displayed a fast rate of parasitemia reduction, similar to chloroquine, reaching the limit of quantification (LQ) on the final day of treatment. The ED_90_ was estimated to be below 3 mg/kg (QD × 4) (Figure 4). With these positive efficacy results the series transitioned to lead optimization.

**Figure 4:**
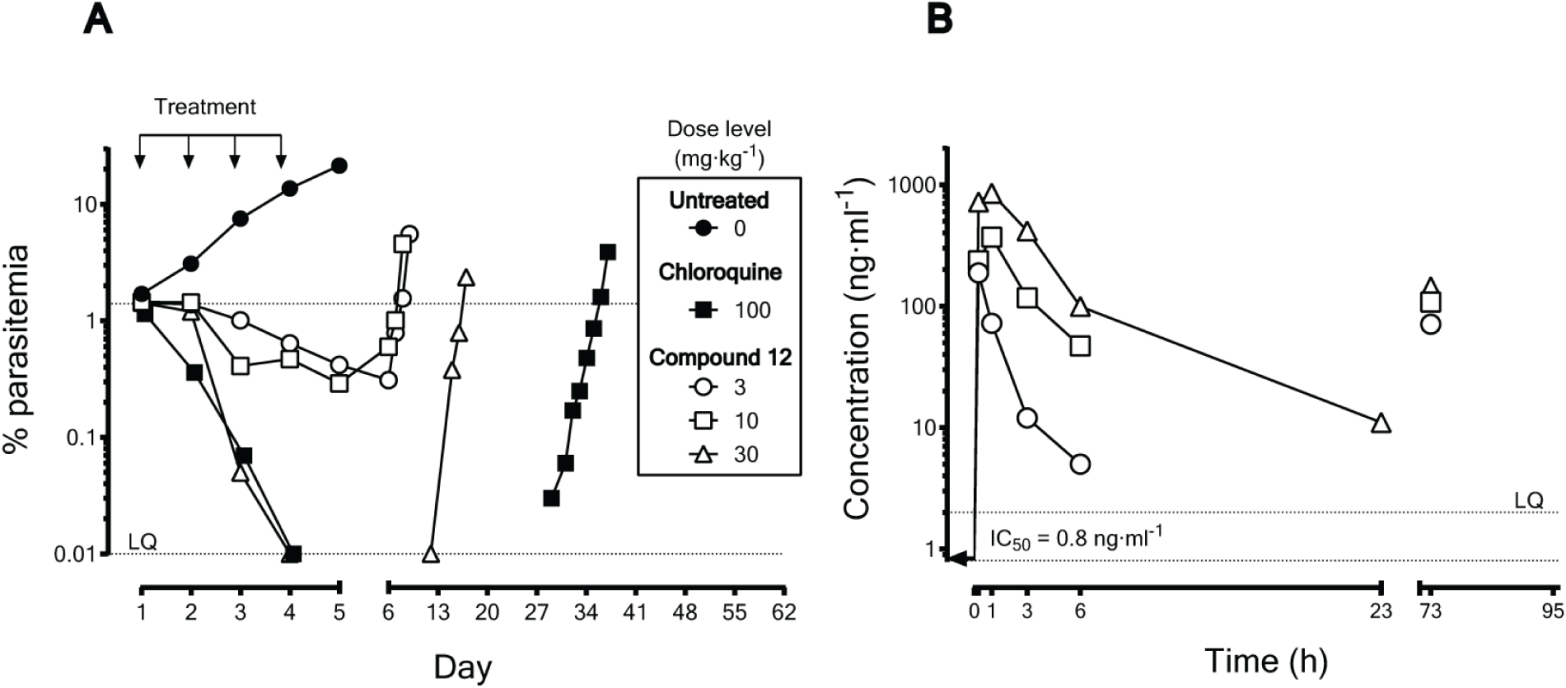
Efficacy study in the *P. falciparum* humanized NOD-scid IL-2Rγ^null^ mouse model. a) Compound **12** was dosed orally at 3, 10, and 30 mg/kg once a day for 4 days and chloroquine (positive control) was dosed orally at 100 mg/kg once a day for 4 days. Parasitemia is expressed as % of *P. falciparum* in human erythrocytes measured every 24 h by flow cytometry. Parasitemia below 0.01% (LQ) could not be quantified. b) Total blood concentration of compound **12** in infected mice on the first day and final day of dosing.

### Lead Optimization

#### Optimization of Bioavailability and CYP Inhibition of Neutral Leads

The goal of lead optimization was to identify a safe compound with an anticipated human single oral dose lower than 500 mg to cure malaria infections. This requires a potent and long half-life molecule.^3^ Our strategy to achieve this objective was to focus on the optimization of bioavailability and half-life and the mitigation of the CYP inhibition risk.

Initially, we decided to further explore the substitutions at C4 of the pyrimidine ring. The CF_3_ substituent occupies an induced fit pocket, and other substituents of similar size were likely to be tolerated. Analogues **12** and **16**, with electron withdrawing substituents, were more potent than **17** and **18,** with electron donating substituents. Potency dropped further for compound **19**, likely due to steric clashes in this small pocket (Table 4). Reduction in lipophilicity resulted, as expected, in an improvement in *in vitro* metabolic stability with the methoxy analogue **18** showing hepatic metabolic clearance below the limit of quantification across species. Permeability decreased as lipophilicity decreased while the correlation with kinetic solubility was not as clear. Interestingly, there was a remarkable difference on CYP inhibition among these four compounds. Reducing lipophilicity and electron withdrawing nature of the substituent at C4 of the pyrimidine ring reduced CYP2C9 inhibition (CYP2C9 pIC_50_: CF_3_ > CF_2_H > OCH_3_) and inhibition against other CYP isoforms. We decided to progress compounds **16** and **18** to intravenous and oral pharmacokinetic studies in mouse (Table 5). Mouse oral bioavailability for compound **16** was low (F% 8), unbound clearance (45 mL/min/g) was marginally higher than for compound **12** and unbound volume of distribution (2 L/kg) remained moderate resulting in a similar profile to **12**. More encouragingly, there was an improvement on mouse oral bioavailability for compound **18** (F% 23) combined with low unbound clearance (16 mL/min/g). However, unbound volume of distribution was low (0.5 L/kg) resulting in a similar half-life to **16**. Taken together, neutral leads were potent and offered low to moderate bioavailability and low clearance but did not deliver the long half-life required for a single dose treatment for malaria due to only having low to moderate volume of distribution.

**Table 4:**
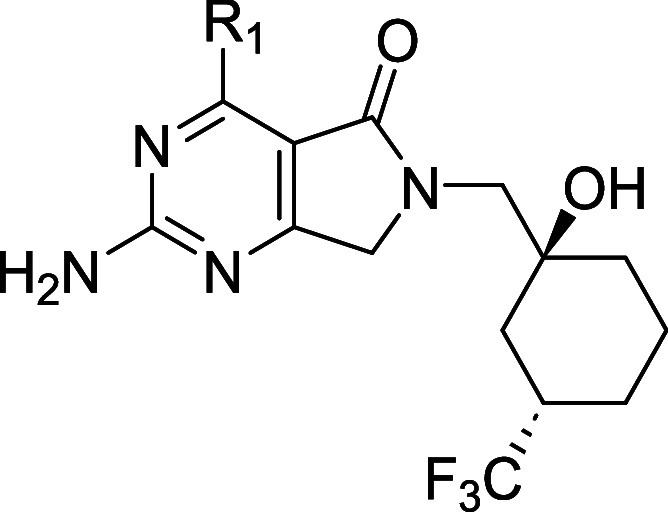
Pyrimidine C4 Substituent

Compound	R1	*Pf*KRS1 pIC_50_ ^a^	*Hs*KRS pIC_50_ ^a^	*Pf*(3D7) pEC_50_ ^b^	HepG2 pEC_50_ ^c^	Heps CL*i*(m, r, h) mL/min/g^d^	Sol μM^e^	PAMPA Pe nm/s^f^ ^f^	CYP2C9 pIC_50_	CHI-logD_pH7.4_ ^g^
**12** ^h^	CF_3_	7.9	4.4	8.5	4.8	1.5, 0.7, <0.5	190	71	6.5	2.2
**16** ^h^	CF_2_H	7.8	4.2	8.7	4.8	1.2, <0.5, <0.5	53	59	4.9	2.0
**17** ^i^	CH_3_	7.0	4.3	7.8	4.6	3.5, <0.5, ND	84	34	ND	1.5
**18** ^h^	OCH_3_	7.5	<4	7.7	<4	<0.5, <0.5, <0.5	175	17	<4	1.4
**19** ^i^	OCF_2_H	6.7	<4	7.0	<4	2.0, 2.6, <0.5	231	32	4.3	2.0

a Inhibition of and human lysyl-tRNA synthetase. Data were from at least three independent replicates, with standard deviations ≤0.2.

b Potency against (3D7) asexual blood stage. Data were from at least three independent replicates, with standard deviations ≤0.2.

c HepG2 (Human Caucasian hepatocyte carcinoma cell) cytotoxicity assay.

d Intrinsic clearance (CL*i*) in mouse, rat and human hepatocytes respectively, scaling factor used is 120 million cells per g Liver.

e Kinetic aqueous solubility at pH 7.4 measured by a UHPLC system equipped with a UV/visible detector and single-quadrupole mass spectrometer.

f PAMPA = parallel artificial membrane permeability assay.

g CHI-LogD_pH7.4_ is a measure of lipophilicity at pH 7.4.

h Single *trans* enantiomer.

i Racemic *trans*. ND: Not Determined. Detailed results are provided in the Supporting Information.

**Table 5:** Mouse Pharmacokinetic Data for **12**, **16,** and **18**

Compound	12^a^		16^b^		18^c^	
Route	IV	PO	IV	PO	IV	PO
Dose (mg/kg)	3.1	10	3	10	2.8	10
C_max_ (ng/mL)		628		301		2883
T_max_ (h)		1		0.5		0.5
T_1/2_ (h)	0.9		0.5		0.4	
AUC_0–8 h_ (ng-min/mL)	219890	87015	141127	37448	274922	229244
Vdss (L/kg)	1		1		0.3	
Vdss,u (L/kg)	2.2		2.0		0.5	
Clb (mL/min/kg)	14		22		10	
Clu (mL/min/kg)^d^	31		45		16	
F (%)		12		8		23

a Compound **12:** mouse PPB fu = 0.446.

b Compound **16:** mouse PPB fu = 0.490.

c Compound **18** mouse PPB fu = 0.640.

d For the purpose of calculating unbound plasma clearances a blood to plasma (B/P) ratio = 1 was assumed.

### Optimization of Basic Leads: Introducing Basicity to Increase Half-Life

A common strategy to increase half-life is the introduction of basicity to increase volume of distribution.^21^ Guided by the structural information available, we selected C1 of the cyclohexyl for the introduction of a basic substituent. This vector is in proximity to a glutamic acid (E464) and allows access to several negatively charged residues at the opening of the ATP binding pocket towards a more solvent exposed region. We introduced a variety of basic substituents at C1 designed to interact with E464 (**20**) or to occupy the opening of the pocket (**20–36**) (Table 6).

**Table 6:**
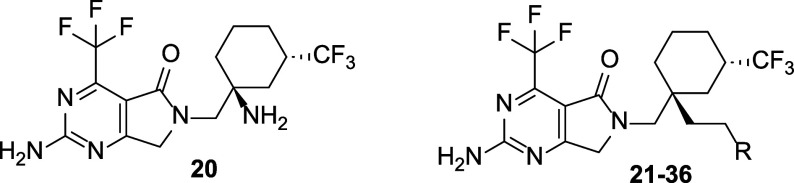

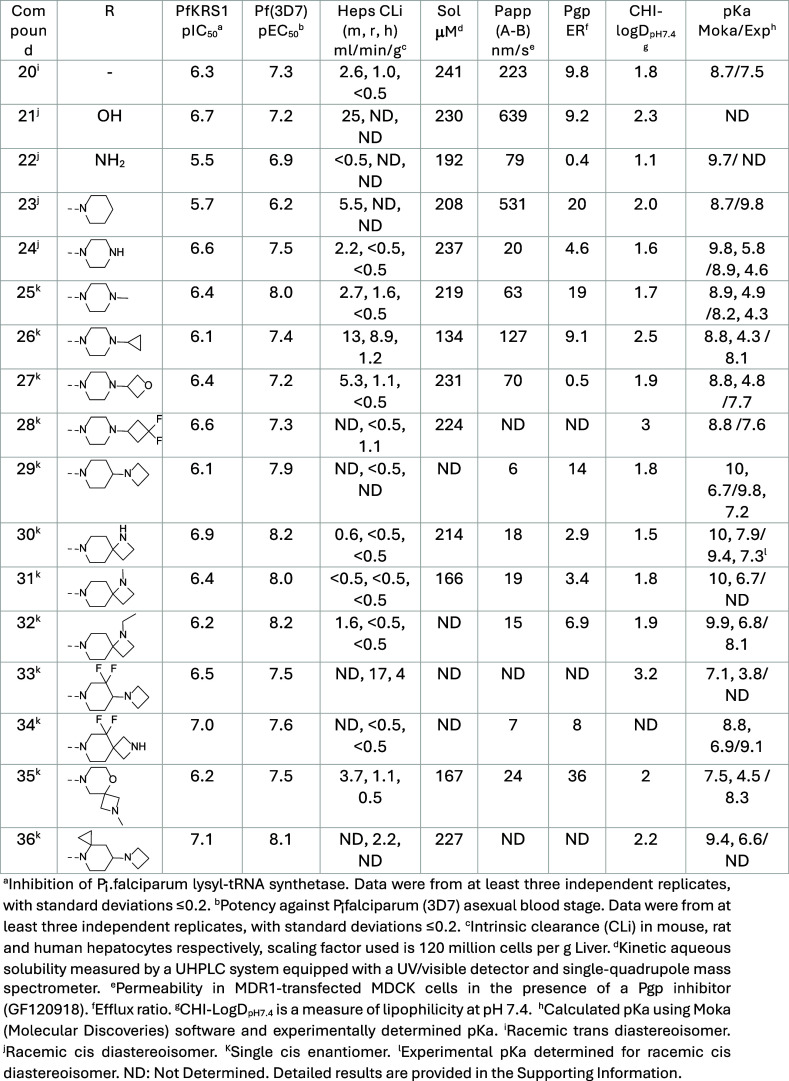
Basic Analogues

The introduction of an amino group at C1 (**20)** resulted in a 20-fold drop in phenotypic potency compared with alcohol **11.** The next set of compounds was designed with a two-carbon linker between C1 and a basic group. The two-carbon linker was the minimum length necessary to avoid a clash of the basic center with R295, a residue that interacts with the substrate lysine and closes the adenine binding pocket (Table 6). Compound design was assisted by docking, predictions of p*K*_a_ and binding affinity. Considering that the target product profile for new antimalarials includes a desirable price of ≤1USD per treatment, we selected commercially available achiral amines to control the cost of goods. Alcohol **21** (racemic), an intermediate in the synthesis of this set of compounds, showed that the linker and the polar group were tolerated, with less than 10-fold drop in potency compared to **11** (racemic). In contrast, primary amine **22** and piperidine **23** resulted in lower biochemical inhibition and phenotypic potency. Piperazine **24,** with an ionizable nitrogen further away from the adenine binding site and closer to negatively charged residues (E464 and E457), was better tolerated. In addition, **24** was soluble and metabolically stable in hepatocytes across species. However, it suffered from limited passive permeability, likely due to a combination of low lipophilicity and high percentage of ionized species at physiological pH (experimental p*K*_a_ 8.9, 4.6) resulting in negligible oral bioavailability in rats (Table 7). The introduction of a basic piperazine in **24** resulted in a substantial increase in volume of distribution (6.5 L/kg) but, unexpectedly, higher blood clearance than anticipated considering the excellent stability in *in vitro* rat hepatocytes. In fact, a large proportion of **24** was detected in the stools after iv dosing suggesting a hepatobiliary clearance mechanism.

**Table 7:** Rat Pharmacokinetic Data for Piperazines **24–28**

Compound	24^a^		25^b^		26^c^		27^d^		28^e^	
Route	IV	PO	IV	PO	IV	PO	IV	PO	IV	PO
Dose (mg/kg)	1	3	1	3	1	3	1	3	1	3
Dose in feces_(0–24 h)_ (%)	25	30	8	11	0.3	0.3	1.9	2.1	ND	ND
C_max_ (ng/mL)		BLQ		38		52		118		326
T_max_ (h)				1		1.3		1		2
T_1/2_ (h)	2.3		1.5		1.3		1.9		4.3	
AUC_0–8 h_ (ng-min/mL)	22427	0	19192	10944	22454	14234	22744	26303	212650	89801
Vdss (L/kg)	6.5		5	-	3.5	-	2.7	-	0.4	
Vdss,u (L/kg)	9.6		8.0		9.1		4.5		1.2	
Clb (mL/min/kg)	48		52		45		44		5	
Clu (mL/min/kg)^f^	70		83		117		71		15	
F (%)		0		19		21		39		14
Fabs (%)		0		57		50		89	-	15

a Rat PPB: Compound **24** fu = 0.678.

b Compound **25** fu = 0.624.

c Compound **26** fu = 0.384.

d Compound **27** fu = 0.602.

e Compound **28** fu = 0.326.

f For the purpose of calculating unbound plasma clearances a blood to plasma (B/P) ratio = 1 was assumed.

Subsequently, our efforts were directed toward improving permeability and reducing hepatobiliary transport. Common strategies reported to mitigate active transport are reducing H-bond donors and TPSA and increasing branching.^22^ In addition, we designed analogues of **24** with a modest reduction in basicity to increase passive permeability. Piperazine analogues **25–28** were tolerated with improved passive permeability. In rat pharmacokinetic studies, these four piperazine analogues had low to moderate oral bioavailability (Table 7). Compound **27** displayed the best bioavailability and fraction absorbed, correlating well with improved passive permeability and low Pgp efflux ratio. Encouragingly, low levels of parent compound were detected in the stools following intravenous administration suggesting that hepatobiliary clearance was not a major clearance mechanism for compounds **25–27**. Finally, unbound volume of distribution decreased with the introduction of substitution on the piperazine (cPr < Me < oxetane < di-F,F-cyclobutyl) while clearance was moderate for **25–27** and low for **28**. This resulted in compound **28** displaying the longest half-life driven by low clearance instead of a boost in volume of distribution. In summary, compound **27** displayed moderate rat bioavailability (F% 39) but without the desired boost in half-life and compound **28** displayed a boost in half-life (4.3 h) but unfortunately, given that metabolic stability in human hepatocytes was lower than in rats, it was anticipated that its half-life in humans would be reduced. These pharmacokinetic profiles coupled with moderate phenotypic potency (pEC_50_ ≤ 8) precluded further progression for compounds **27** and **28**.

We then focused on improving potency by strengthening the electrostatic interactions with the negatively charged residues at the opening of the ATP binding site. We prepared dibasic compounds **29** and **30** with a second ionizable nitrogen further from the core. As predicted by binding mode studies, a crystal structure of *CpPf*KRS bound to lysine and **30** showed the azetidine ring interacting through water mediated interactions with D415, E457, E423, and E500 (D450, E493, E458, and E500 in *Pf*KRS1) and the piperazine moiety interacting with the carboxylic acid of the lysine substrate (Figure 5). This network of electrostatic interactions led to an improvement in potency compared with the piperazine monobasic analogues. Compound **30** was potent (pEC_50_ 8.2) and soluble and displayed low clearance in hepatocytes across species. However, permeability was low due to high polarity (shake flask experimental logD = 0.2). We thus decided to cap the azetidine NH with methyl **31** and ethyl **32** to reduce the number of H-bond donors, increase lipophilicity, and reduce basicity. Encouragingly, **31** and **32** maintained potency and metabolic stability but permeability remained low for both compounds. We then prepared a set of analogues with commercially available diamines (**33–36**) to modulate basicity and reduced polarity to improve permeability. However, none of these analogues combined low clearance and improved permeability.

**Figure 5:**
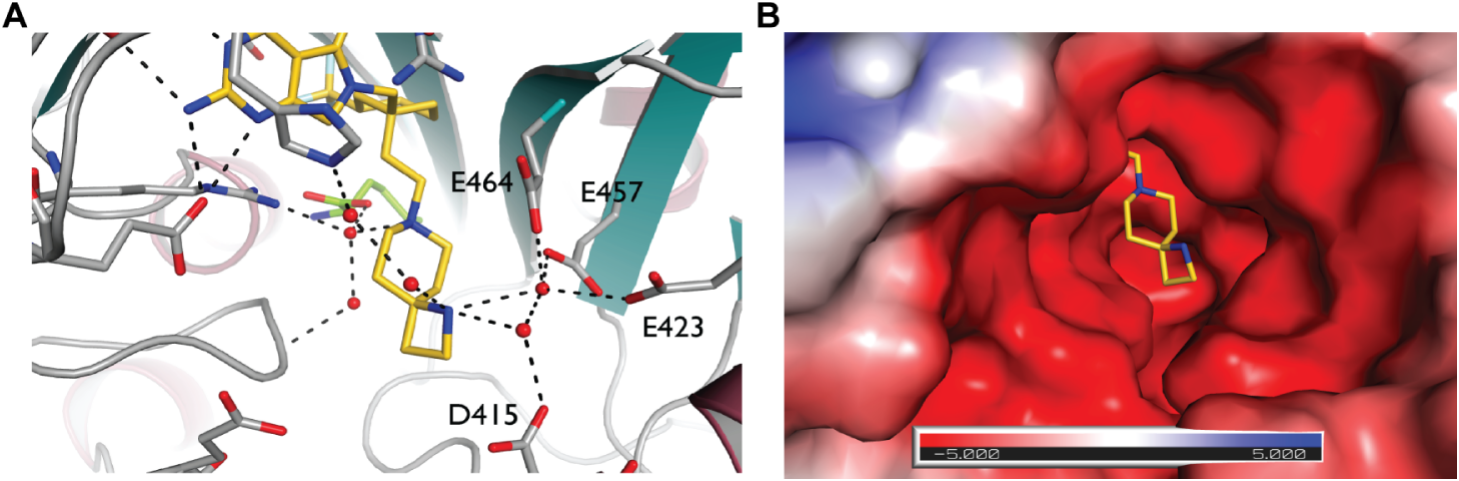
(a) Interactions of **30** basic side chain in cocrystal structure with *Cp-Pf*KRS chimeric protein with **30** (yellow) and lysine (green) (PDB ID code 9r3g). (b) Protein surface electrostatics. Figures were prepared using PyMol: The PyMOL Molecular Graphics System, Version 2.5.5 Schrödinger, LLC.

In pharmacokinetic studies, compound **30** showed high volume of distribution across species, moderate clearance in mice and low in rats and dogs resulting in long half-lives across species (Table 8). Encouragingly, we predicted a long human half-life (35 h) using MMV Sola^23^ showing potential for single oral dose malaria treatment. However, low passive permeability limited absorption resulting in negligible rat oral bioavailability. Ethyl analogue **32** retained low clearance, high volume of distribution and long half-life but bioavailability increased only marginally.

**Table 8:** Pharmacokinetic Data for Compounds **30** and **32**

Compound	30^a^			32^b^	
Species	Mouse	Rat	Dog	Rat	
Route	IV	IV	IV	IV	PO
Dose (mg/kg)	3	1	0.3	1	2
C_max_					12
T_max_ (h)					4
T_1/2_ (h)	7	21	37	14	
AUC_0–8 h_ (ng-min/mL)	43648	134488	15900	54995	4051
Vdss (L/kg)	12	7	14	13	
Vdss,u (L/kg)	20	12	77	19	
Clb (mL/min/kg)^c^	67	5	19	18	
Clu (mL/min/kg)	114	8	105	26	
F (%)					4

a Compound **30:** mouse/rat/dog PPB fu = 0.585/0.591/0.179.

b Compound **32:** rat PPB fu = 0.695.

c For the purpose of calculating unbound plasma clearances a blood to plasma (B/P) ratio = 1 was assumed.

All the basic analogues progressed to *in vivo* pharmacokinetic studies were evaluated in terms of selectivity, potential for DDI and hERG inhibition (Table 9). These compounds displayed high levels of biochemical and cellular selectivity with negligible inhibition of human KRS and HepG2 cell growth. Compounds **27** and **30** exhibited low micromolar inhibition of CYP2D6 and CYP3A4 respectively, while other basic compounds inhibited at least one of these two enzymes with IC_50_ below 1 μM. We were pleased to see that **24**, **30** and **32** did not inhibit hERG despite the introduction of basicity and compound **30** was negative in an Ames assay (5 strains ± S9).^24,25^

**Table 9:** Summary of Key Selectivity and Safety Data for Basic Leads^a^ ^b^

Assays	24^a^	25	26	27	30	32
*Hs*KRS pIC_50_	<4	<4	<4	<4	<4	<4
HepG2 pEC_50_	<4	4	<4	<4	<4	<4
CYP (2D6, 3A4)^b^ pIC_50_	5.4, 6.0	6.2, 5.6	6.3, 6.2	5.3, 4.8	4.9, 5.6	ND
hERG Qpatch IC_50_ (μM)	>30	ND	0.9	8.1^a^	>30	>30

a Racemic trans.

b CYP1A2, CYP2C9, and CYP2C19 pIC_50_ < 5.

### Prodrugs to Increase Oral Bioavailability

At this point, compound **30** was the series lead, a potent inhibitor of parasite growth displaying low clearance and the desired long half-life across preclinical species, but **30** suffered from a lack of bioavailability due to poor permeability. Its safety profile was favorable: Ames negative, no significant hERG inhibition, low potential for drug–drug interactions, and good biochemical and cellular selectivity. Taken together, **30** had a promising profile for progression as a single dose cure antimalarial if it could be delivered systemically.

At this final stage of the project, we prioritized a prodrug approach to improve absorption and mitigate the low bioavailability of **30**. We prepared a small set of prodrugs to optimize permeability and the rate of conversion to the active molecule in cross-species hepatocytes (Table 10). It was encouraging to see that our prodrugs, **37–40**, showed rapid conversion to **30** in mouse, rat, and human hepatocytes. Among these four analogues, we selected **39** for an *in vivo* pharmacokinetic study. Prodrug **39** retained rapid conversion to **30** in hepatocyte cross-species and displayed moderate FaSSIF solubility (221 μM) despite its lower kinetic solubility (27 μM) and improved permeability in MDCK cells. Disappointingly, while **39** was rapidly converted into **30** systemically when dose intravenously, it failed to improve bioavailability when dosed orally. In fact, no **39** or **30** were detected systemically after dosing **39** orally. To investigate this lack of oral bioavailability, we measured permeability in MDR1-transfected MDCK cells in the presence and absence of a P-gp inhibitor. Interestingly, all three prodrugs exhibited high efflux ratios, which likely compromise absorption.

**Table 10:**
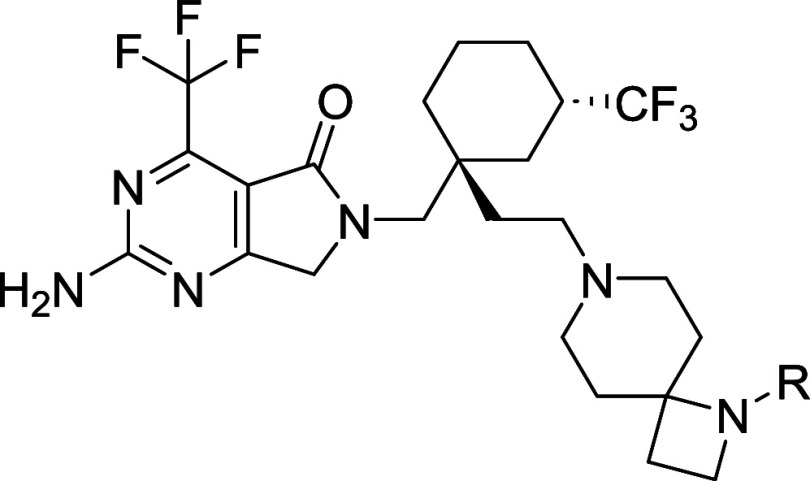

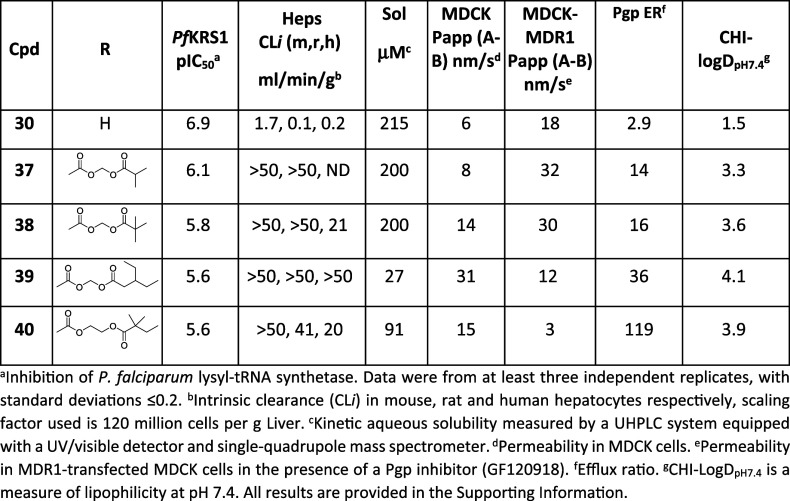
Prodrugs of Compound **30**

In summary, we carried out extensive studies to identify a potent basic orally bioavailable KRS inhibitor with a long half-life and potential for single dose cure of malaria. When this initial strategy was deemed exhausted, we explored a pro-drug approach to deliver **30** systemically. Disappointingly the prodrug approach failed to increase absorption.

### Activity Predictive Model for Compound Prioritization

A total of 27 *Pf*KRS1 inhibitors were included to generate a FMO–DFTB (Fragment Molecular Orbital- density-functional tight-binding) activity model for *Pf*KRS1 inhibition (Figure S4). The experimental pIC_50_ of these compounds range from 4.6 to 7.1. The compounds were docked into a homology model of *Pf*KRS1, which was generated from the *Cp*KRS cocrystal structure with compound **22**. Docked poses were filtered by RMSD analysis to identify those with an alignment consistent with the template crystal structure. The complexes were then minimized briefly with Protein Preparation Wizard in Maestro, Schrödinger (version 2021–2) with convergence of heavy atoms set to 0.5 Å RMSD. We applied a regression model incorporating both FMO–DFTB calculation and calculated logP from StarDrop (eq 1). The theory and procedures have been detailed in separate publications.^26,27^


1 $$pIC_{50}=\alpha \;EFMO-DFTB+\beta logPStarDrop+\gamma$$

The α, β, and γ in eq 1 were optimized to be −0.0096, 0.88, and 1.88, respectively. The correlation of training data points gives a R^2^ of 0.73 and a RMSE (root-mean-square error) of 0.301 (Figure 6). This model was applied to prioritize novel designs at the lead optimization stage of the project to evaluate the possibility to introduce a basic center with the aim of improving half-life.

**Figure 6:**
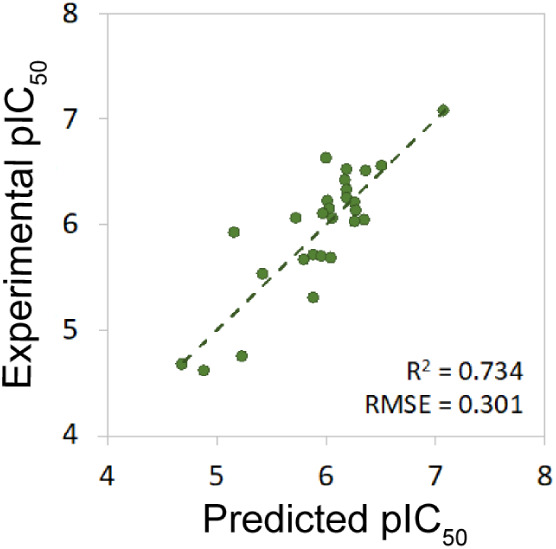
Correlation between FMO–DFTB (Fragment Molecular Orbital- density-functional tight-binding) activity prediction and experimental inhibition of *Pf* KRS1.

### Pharmacological Profile

Lysyl-tRNA synthetase inhibitors **12** and **30** were profiled in asexual blood stage assays with a range of drug-resistant *P. falciparum* strains as well as *P. falciparum*, *P. vivax*, *P. ovale* *and* *P. malariae* clinical isolates from America and Africa (Table 11). Both compounds were potent (<10 nM) against laboratory strains resistant to clinical antimalarials and compounds in development. Potency was maintained against field isolates of *P. falciparum* and *P. vivax*, the two species responsible for most of the malaria disease burden.^28^ Interestingly, compound **30** also retained activity against *P. ovale* and *P. malariae* isolated from patients in Ghana and Mali.^29,29^ To assess the potential of KRS inhibitors for chemoprotection, prophylaxis and transmission-blocking activity, **12** and **30** were tested in liver, sexual and mosquito stages (Table 11). Compound **12** retained activity against *P. falciparum* and *P. vivax* liver schizonts, in contrast to compound **30** that was significantly less potent. We hypothesized that low permeability limited access of **30** to liver schizonts inside hepatocytes, explaining its lack of potency. Compounds **12** and **30** were inactive against *P. vivax* hypnozoites.^30,31^ Compound **30** retained activity within 5-fold of the asexual blood stage activity in several assays for sexual and mosquito stages, indicative of potential for blocking malaria transmission.^32,33^

**Table 11:** *In Vitro* Activity Profile against *P. falciparum* Strains, Field Isolates, and Life Cycle Stages (nM)

		**Compound**	
Life cycle stage	Assays	12	30
*Plasmodium* asexual blood stage (treatment of symptoms)	*Pf*(3D7) EC_50_ (SYBR green)	2	6
	*Pf*(NF54) EC_50_ (3H hypoxanthine)	6	4
	*Pf (*7G8, Dd2, Dd2CARL, Dd2cyBC1, Dd2DHODH, Dd2eEF2, Dd2PI4K, K1, RF12, TM90) EC_50_ (3H hypoxanthine)^34^	ND^a^	3–5
	Brazilian isolates^28^ *Pf* EC_50_	1	2
	Brazilian isolates^28^ *Pv* EC_50_	3	6
	Field isolates *Pf* EC_50_ (Mali, Ghana)	ND, ND	5, 2
	Mali isolates^29^ *Pm* EC_50_	ND	5
	Ghana isolates^35^ *Po* EC_50_	ND	3
*Plasmodium* liver stage (chemoprotection)	*Pv* liver schizonts^31^ EC_50_	22	>10,000
	*Pv* liver hypnozoites^31^ EC_50_	>50,000	>10,000
	*Pf* liver schizonts EC_50_	4	734
*Plasmodium* sexual and mosquito stages (transmission blocking)	*Pf* Gametocytes (early/late)^32^ EC_50_	11, 50	3, 3
	*Pf* DGFA (male/female)^33^ EC_50_	50, 73	14, 13
	*Pf* oocyst intensity EC_50_	30	15

a ND: Not Determined.

As expected for two compounds with the same mode of action, **12** and **30** displayed a similar parasite rate of kill comparable with pyrimethamine. This was determined in a *P. falciparum* viability assay using serial limiting dilution to quantify the parasites that remain viable after drug treatment.^36^ The *in vitro* log parasite reduction ratio for **12** and **30** was 4.2 and 3.4 at 10 × *Pf*(3D7) EC_50_ and the parasite clearance time (PCT 99.9%) was 53 and 56 h, respectively. Both compounds showed a 24 h lag phase similar to pyrimethamine (Figure 7).

**Figure 7:**
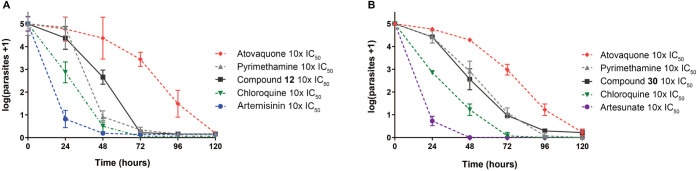
*In vitro* parasite reduction ratio (PRR) assay showing kill kinetics for **12** (A) and **30** (B). Results previously reported for standard antimalarials using the same conditions are shown for comparison.

### Evaluation of Resistance Risk and On-Target Activity

Emergence of resistance is one of the major concerns in antimalarial drug development, and therefore evaluation of resistance potential for target/compound series pairs is essential. To assess the resistance risk for this series, we determined the minimum parasite inoculum needed to obtain a single event of resistance (minimum inoculum of resistance, MIR).^37^ Compounds requiring a minimum of 10^8^ parasites (log_10_ MIR > 8) to generate resistance are preferable while those with log_10_ MIR ≤ 6 and a EC_50_ shift greater than 10-fold compared to parent Dd2-B2 parasites are considered high risk. We determined the frequency of resistance for compound **11** by exposing different parasite inocula to high compound concentrations (4.5 × EC_50_). Experiments were carried out in triplicate. Recrudescence was observed on day 16 of the study in one of three flasks with 10^8^ parasites while the other two flasks remained parasite-negative for 60 days. The recrudescent parasites exhibited a 300-fold shift in EC_50_ compared to parent Dd2-B2 parasites. Whole-genome sequencing (WGS) of three resistant clones unveiled a S344L mutation in the gene coding for cytosolic lysyl-tRNA synthetase (KRS1, PF3D7_1350100) thus confirming on-target activity of **11** (Table S7). The mutation of serine 344, in the ATP binding pocket, to leucine, a larger lipophilic amino acid, is predicted to cause a steric clash with the cyclohexyl substituent. Interestingly, we previously reported the same mutation for a different chemical series of inhibitors of *Pf*KRS1.^20^ No other consistent SNPs were identified elsewhere in the genome. Moreover, the S344L variant was not present in the >7,000 *P. falciparum* genomes in the MalariaGen database.^38^ In fact, the naturally occurring PF3D7_1350100 gene variants were mostly synonymous (change of base-pair without changing amino-acid) with no nonsynonymous changes in structural features at the binding site.

During lead optimization, the advanced lead **30** was assessed in single step selections with the *P. falciparum* Dd2-B2 clone pressured at 3 × EC_90_, with two starting inocula of 1.2 × 10^6^ parasites per well × 12 wells and 1 × 10^7^ parasites per well × 3 wells (for a total of 4.4 × 10^7^ parasites). Parasite cultures were monitored for recrudescence three times weekly by flow cytometry and microscopy. No recrudescence was observed. We further tested **30** at higher inoculum where we seeded 6 wells each with 7 × 10^7^ Dd2-B2 parasites and subjected them to continuous drug pressure equivalent to 3 × EC_90_. Recrudescence was observed in only one of the six wells after 22 days, thus corresponding to a log_10_ MIR = 8.6 (Table S8). The recrudescent parasites exhibited a mean 25-fold shift in EC_50_ compared to parent Dd2-B2 parasites and, upon WGS analysis, were found to harbor two mutations. The first was F342Y in KRS1 (PF3D7_1350100) while the second was a S1281* stop codon mutation on ATP-dependent RNA helicase DBP10 (PF3D7_0827000) (Table S9). DSM265 was included as a reference, with all wells recrudescing following selection with 3 × EC_90_ (MIR < 1.2 × 10^6^ parasites).

Taken together, the resistance risk for this series of *Pf*KRS1 inhibitors was deemed manageable and did not preclude further progression. Furthermore, we identified *Pf*KRS1 as a key mediator of resistance for both **11** and **30** strongly suggestive of on-target activity.

### Chemistry

Compounds **2**, **3** and **4** were synthesized from intermediate bromide **42**, which was formed from the bromination of methyl pyrimidine **41**, as outlined in Scheme 1. Treatment of this benzylic bromide with a series of amines effectively furnished the desired lactams through a single-pot alkylation/amidation reaction.

**Scheme 1:**
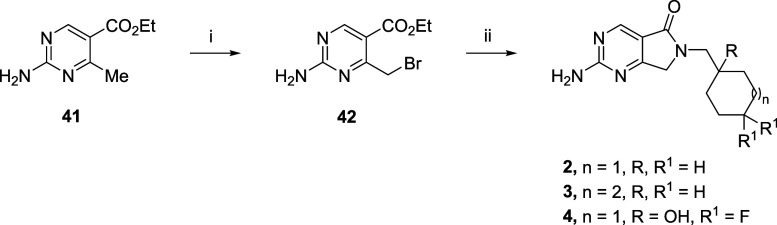
Synthesis of Compounds 2, 3 and 4; (i) Br_2_, AcOH, 70 °C, 62%; (ii) Amine, LiOH, DMF rt; or Amine, NMP, 130 °C, 34–49%

As shown in Scheme 2, the doubly protected hydrazine **43** was methylated using methyl iodide, then the benzyl carbamate (Cbz) was removed via a hydrogenation reaction. A nucleophilic aromatic substitution (SNAr) was performed using this hydrazine, followed by a second SNAr with dimethoxybenzylamine. Cleavage of the remaining *tert*-butyl carbamate (Boc) group facilitated a cyclization to afford compound **5**.

**Scheme 2:**
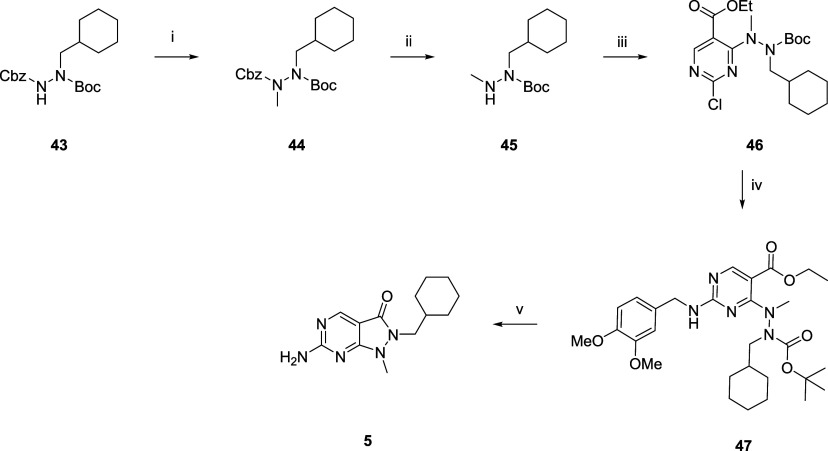
Synthesis of Compound 5; (i) NaH, THF, MeI, rt, 76%; (ii) Pd/C, H_2_, MeOH, rt, 76%; (iii) Ethyl 2,4-Dichloropyrimidine-5-carboxylate, DIPEA, MeCN, rt, 79%; (iv) (2,4-Dimethoxyphenyl) Methanamine, DIPEA, DMF, rt, 45%; (v) TFA, rt, 48%

The pyridine analogues **6–9** were all formed through the same general route (Scheme 3); beginning with formylation at the 4-position of a functionalized pyridine ring, followed by a single-pot reductive-amination/amidation. In the case of compound **6**, the amino group was installed onto the pyridine ring via a Buchwald-Hartwig amination of *tert*-butyl carbamate, which was subsequently deprotected using acid. Similarly, the amino groups on pyridines **8** and **9** were installed through a nucleophilic aromatic substitution using 2,4-dimethoxy benzenylamine, which again was deprotected using acid.

**Scheme 3:**
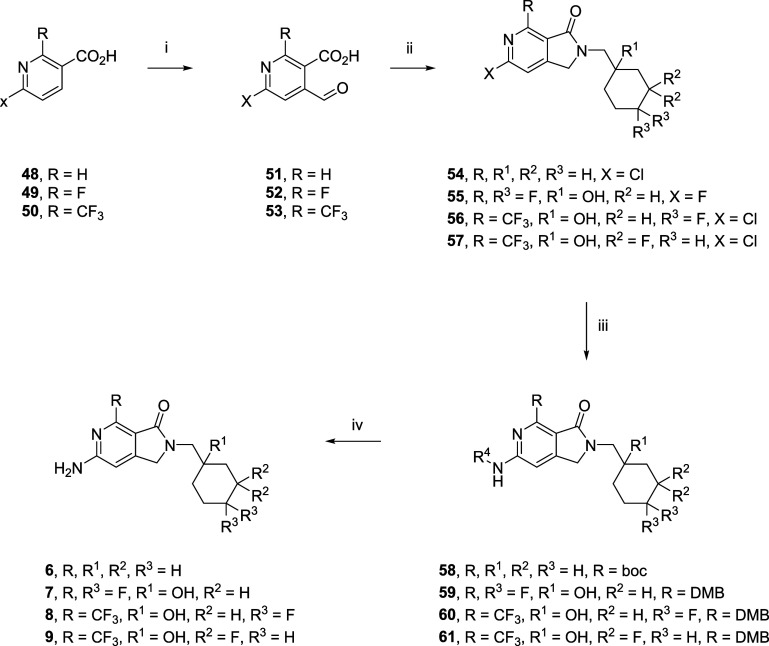
Synthesis of Compounds **6–9**; (i) TMP, *n*-BuLi, THF, −78 °C, then DMF; (ii) Amine, AcOH, DCM, rt, then STAB, 26–44%; (iii) Amine, Pd­(dba)_2_, XantPhos, Cs_2_CO_3_, THF, 65 °C, or Amine, DIPEA, DMF/DMA/DMSO, 40 °C–120 °C, 13–40%; (iv) TFA, DCM, rt, 14–29%

The route toward compounds **10** and **11** began with an ambient light promoted three-component reaction (Scheme 4). Based on chemistry reported by Bi et al., guanidine hydrochloride, 3-oxobutanoate, and pentafluoroethyl iodide were employed in a formal [2 + 1 + 3] annulation to elegantly and efficiently afford the highly functionalized amino pyrimidine **62**.^39^ As before, bromination followed by a single-pot alkylation/amidation furnished the desired final compounds. For compound **11**, the amine portion was first synthesized through a Corey-Chaykovsky epoxidation of 3-trifluoromethyl cyclohexanone in a highly diastereoselective manner. Treatment with methanolic ammonia opened the epoxide to afford primary amine **66** with a dr of 20:1. Compound **11** was separated into its four enantiomeric components (**12–15**) using supercritical fluid chromatography (SFC).

**Scheme 4:**
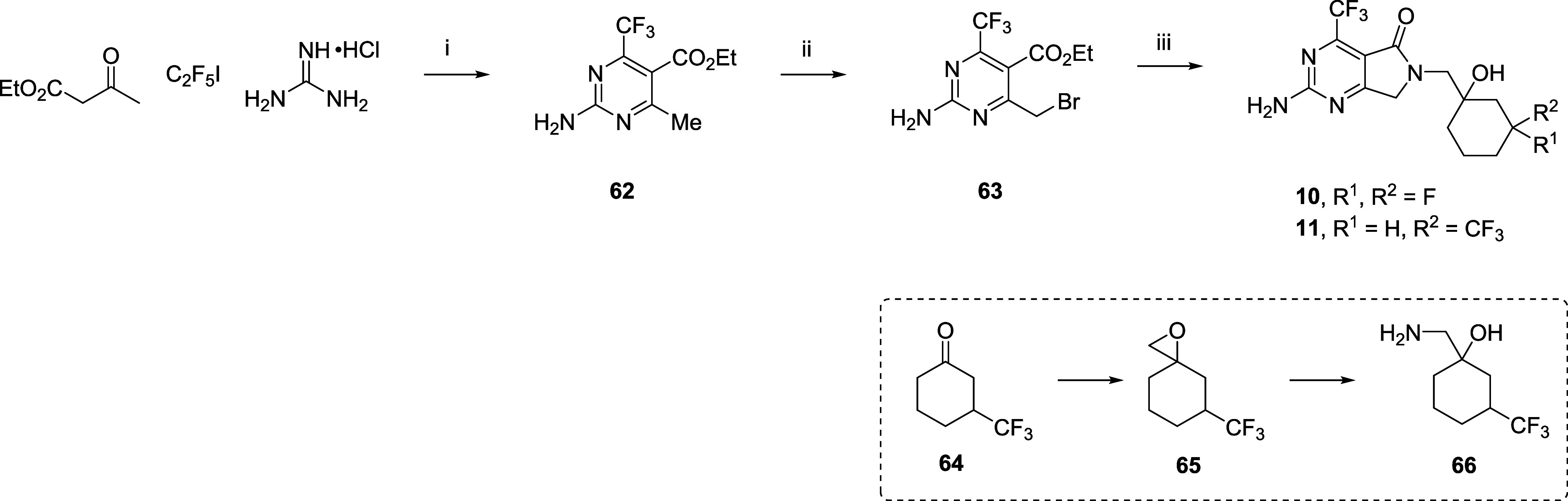
Synthesis of Compounds **10** and **11** (**11** Separated into **12–15**); (i) NaOH, MeCN, Blue Light, rt, 30%; (ii) Br_2_, AcOH, CHCl_3_, 60 °C, 51%; (iii) Amine, Et_3_N, MeCN, 60 °C or rt, 78%

Diversification of the 4-position required unique syntheses for each analogue. Scheme 5 outlines the synthesis of compound **16**. Pyrimidine **67** was bis-Boc protected, and an ester group was installed with ethyl chloroformate. A Suzuki-Miyaura coupling was used to attach a vinyl group, which was then oxidatively cleaved and difluorinated using DAST. The coupling/oxidative cleavage protocol was repeated, and a reductive amination/lactamisation was performed to afford compound **16**.

**Scheme 5:**
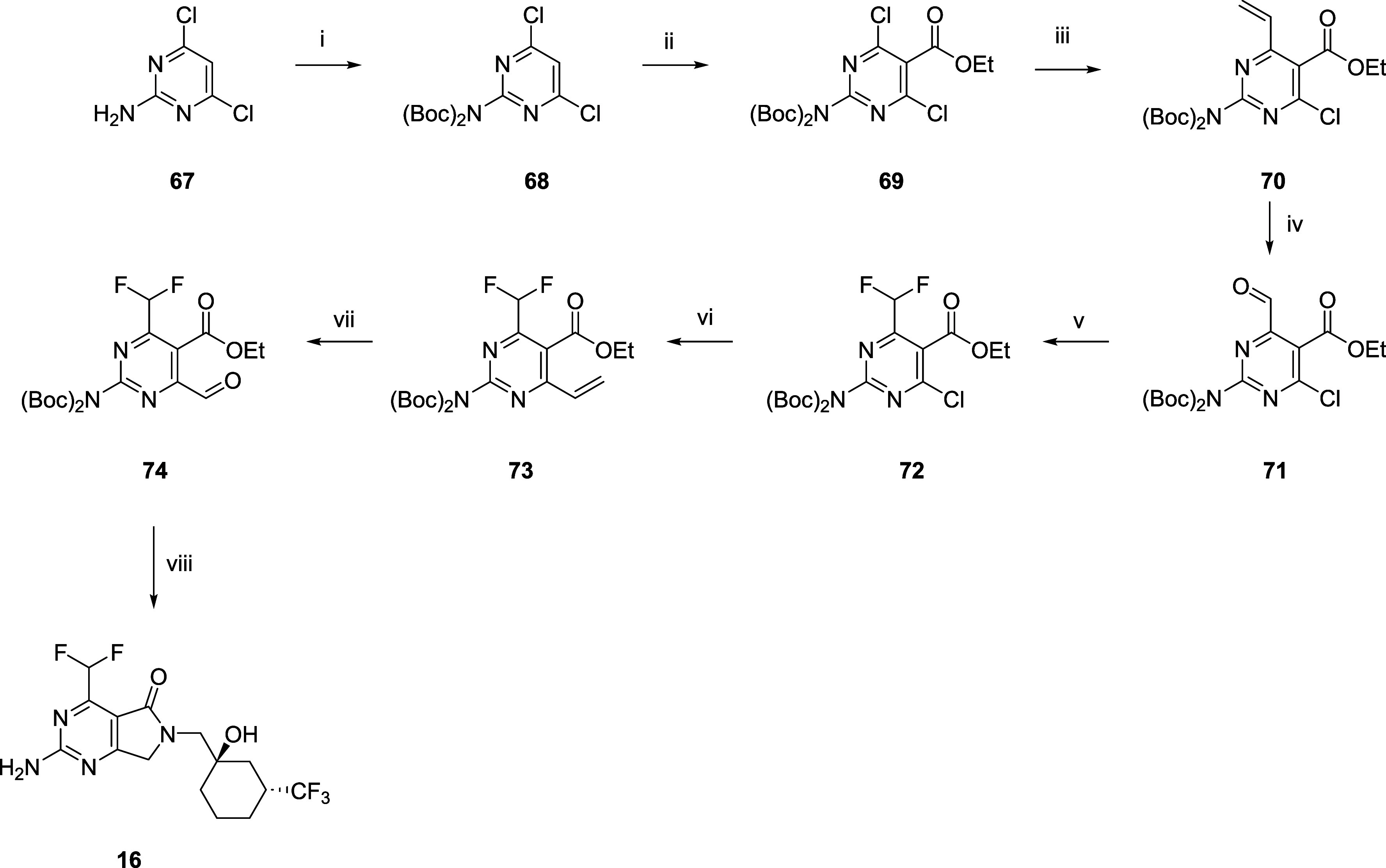
Synthesis of Compound **16**; (i) Boc_2_O, DMAP, THF, 10 °C, 77%; (ii) LDA, Ethyl Chloroformate, THF, −78 °C, 24%; (iii) Vinyl BF_3_K, Pd­(dppf)­Cl_2_, Cs_2_CO_3_, Dioxane, Water, 90 °C, 28%; (iv) OsO_4_, NaIO_4_, Dioxane, Water, rt; (v) DAST, DCM, 0 °C, 81%; vi) Vinyl BF_3_K, Pd­(dppf)­Cl_2_, Cs_2_CO_3_, Dioxane, Water, 100 °C, 68%; vii) OsO_4_, NaIO_4_, Dioxane, Water, rt, 50%; viii) Amine, STAB, DCM, rt, then HCl/diaxane, rt, 11%

For compound **17**, the route began with a Suzuki-Miyaura employing vinyl potassium trifluoroborate to afford the styrenyl compound **76** (Scheme 6). The newly installed alkene was oxidatively cleaved using dipotassium dioxido­(dioxo)osmium dihydrate, providing an intermediate on which to perform the reductive-amination/amidation process. Finally, the thioether was oxidized using oxone and displaced with a solution of ammonia in dioxane.

**Scheme 6:**
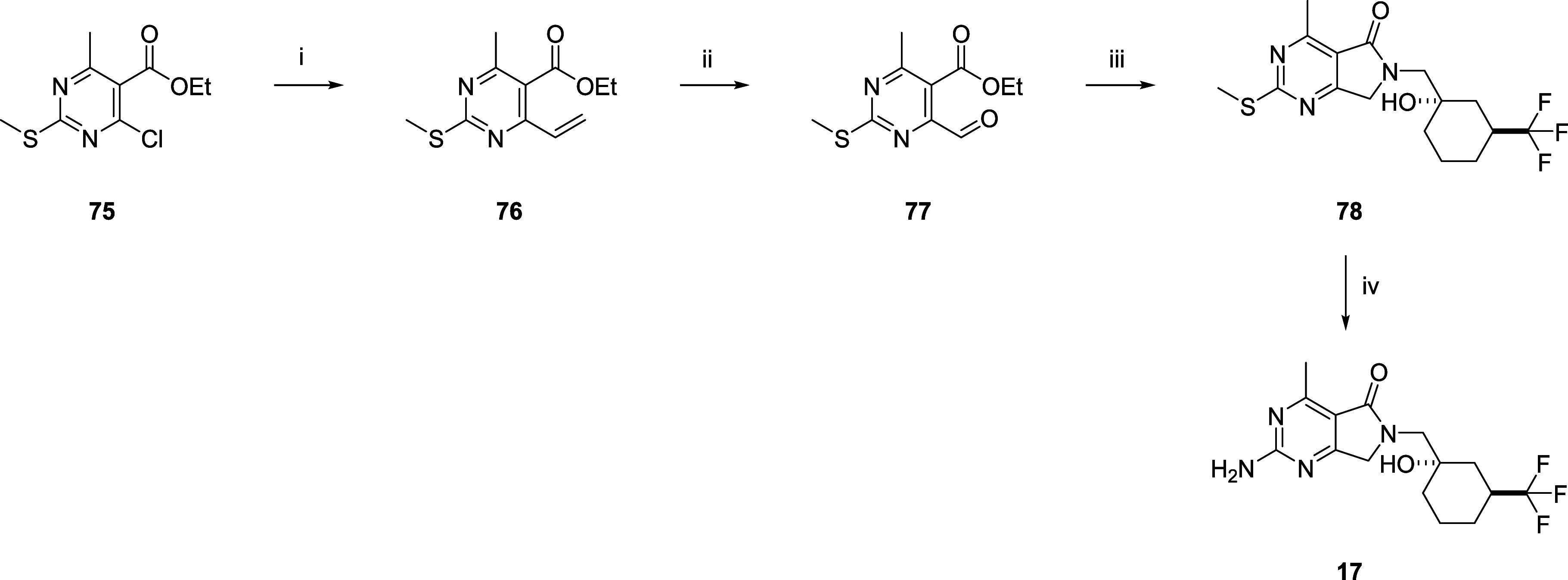
Synthesis of Compound **17**; (i) Vinyl BF_3_K, Et_3_N, Pd­(dppf)­Cl_2_, EtOH, 85 °C, 79%; (ii) K_2_OsO_4_·2H_2_O, THF, Water, rt, Then Sodium Periodate, 44%; (iii) Amine, STAB, DCM, 40 °C, 91%; (iv) Oxone, MeCN, rt, Then NH_3_ in Dioxane, Dioxane, rt, 7%

The route toward compound **18** (Scheme 7) started with the double Boc protection of amino pyrimidine **79** to allow for an acylation with LDA and ethyl chloroformate. As with compound **75**, a vinyl group was installed via a Suzuki-Miyaura reaction and converted to the aldehyde this time using osmium tetroxide and sodium periodate. Once again, the right-hand ring was formed through a reductive amination/amidation and the amino group was exposed upon treatment with acid.

**Scheme 7:**
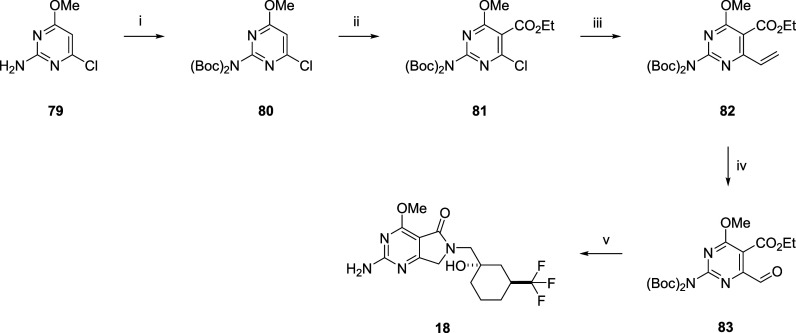
Synthesis of Compound **18**; (i) Boc_2_O, DMAP, THF, rt, 94%; (ii) LDA, THF, Ethyl Chloroformate, 86%; (iii) Vinyl BF_3_K, Et_3_N, Pd­(dppf)­Cl_2_, EtOH, 120 °C, 26%; (iv) OsO_4_, Sodium Periodate, 1,4-Dioxane, Water, rt; (v) Amine, Stab, DCM, rt, then TFA, DCM, rt, 44%

The difluoromethoxy group in compound **19** was installed by treating pyrimidinone **85** with sodium chlorodifluoroacetate (Scheme 8). This intermediate was reacted with ethyl chloroformate to install the ester moiety, then subjected to a similar route as above to ultimately afford compound **19**.

**Scheme 8:**
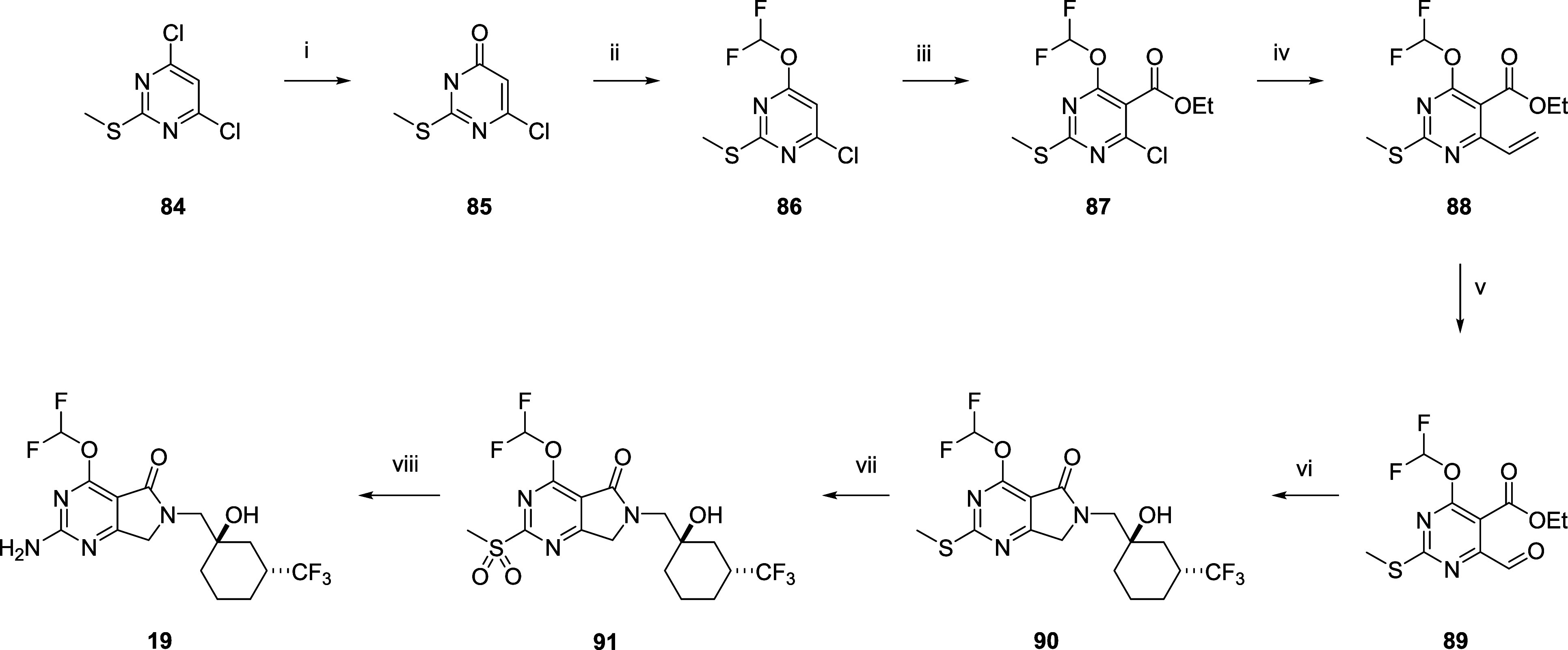
Synthesis of Compound **19**; (i) NaOH (aq.), 74%; (ii) Sodium Chlorodifluoroacetate, Na_2_CO_3_, MeCN, DMF, 90 °C, 82%; (iii) TMP, *n*-BuLi, Ethyl Chloroformate, THF, −78 °C, 20%; (iv) Vinyl BF_3_K, Pd­(dppf)­Cl_2_·DCM, DIPEA, Ethanol, 90 °C, 91%; (v) OsO_4_, NaIO_4_, THF, Water, rt, 59%; vi) Amine, Sodium Cyanoborohydride, MeOH, rt, 39%; vii) mCPBA, DCM, rt, 96%; viii) NH_3_, THF, rt

The amine intermediate required for compound **20** was prepared by transiently forming the imine of 3-trifluoromethyl cyclohexanone with ammonium hydroxide, before adding potassium cyanide and trapping the resultant amine with Cbz chloride. The nitrile group was reduced with borane and this intermediate was subjected to the alkylation/amidation procedure discussed earlier in this section (Scheme 9).

**Scheme 9:**
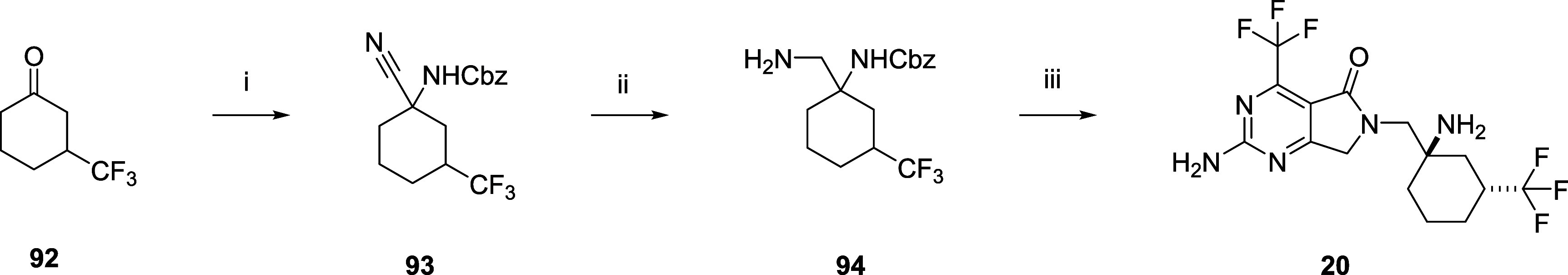
Synthesis of Compound **20**; (i) NH_4_OH, NH_4_Cl, KCN, MeOH, rt, 65%; (ii) BH_3_·DMS, THF, rt, 57%; (iii) **63**, DIPEA, MeCN, 80 °C, then Pd/C, MeOH, H_2_, rt, 13%

The routes towards compounds 21 and 22 began with a Horner–Wadsworth–Emmons reaction on 3-(trifluoromethyl)­cyclohexan-1-one to afford the corresponding α,β-unsaturated ester and nitrile derivatives 96a and 96b, respectively. A diastereoselective conjugate addition of nitromethane was then performed, followed by reduction of the ester or nitrile group using DIBAL or borane to furnish the corresponding intermediates. For compound 21, the nitro group was directly reduced with iron and HCl, and the resulting amino alcohol was subjected to an alkylation/amidation sequence with intermediate 63 to afford the target compound. At this stage, 21 was resolved into single enantiomers using SFC, and the desired enantiomer was subsequently oxidized using Dess–Martin periodinane (DMP). The resulting aldehyde (100) served as a key intermediate for the synthesis of compounds 23–36. For the synthesis of 22, the primary amine in 98b was first Boc-protected prior to reduction of the nitro group by hydrogenation. The resulting amine was then subjected to an alkylation/amidation sequence with 63, followed by Boc deprotection to afford 22.(Scheme 10).

**Scheme 10:**
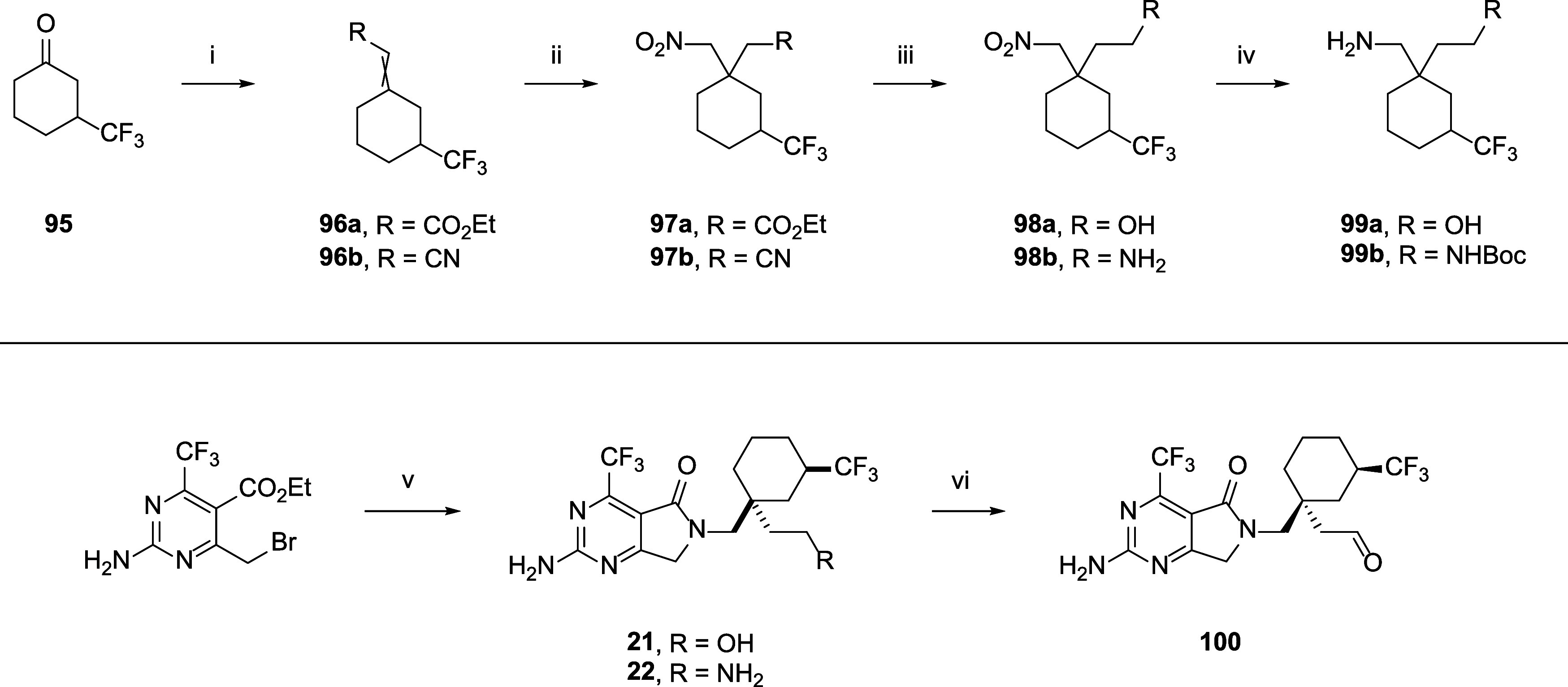
Synthesis of 21, 22 and 100; (i) LiCl, DBU, Ethyl 2-(diethoxyphosphoryl)­acetate, rt, 96%, or NaH, diethyl (cyanomethyl)­phosphonate, rt, 47%; (ii) MeNO2, TBAF, THF, 70 °C, 87% or 58%; (iii) DIBAL, toluene, −78 °C, 67%, or BH3.DCM, toluene; (iv) Fe, NH4Cl, EtOH, water, 80 °C, 89%, or Boc2O, Et3N, DCM, rt, 31% then Pd/C, H2, MeOH, 36%; (v) 99a, DIPEA, EtOH, rt, 67%, or 99b, Et3N, MeCN, rt, 42% then HCl, ether, rt, 94%; vi) DMP, DCM, CHCl3, 0 °C

The amines for this set of compounds were commercial, with the exception of compound **36**. The amine building block for this target was made through a reductive amination of piperidinone **101** with azetidine, and subsequent deprotection using trifluoroacetic acid (Scheme 11).

**Scheme 11:**
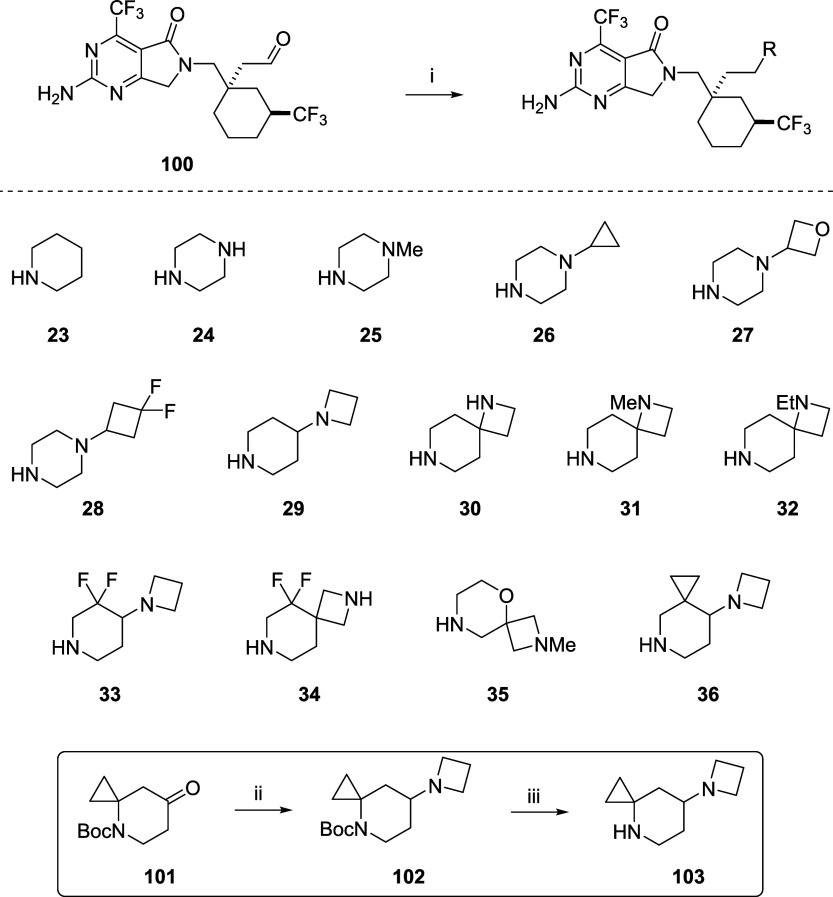
Synthesis of Compounds **23–36**; (i) Amine, STAB, CHCl_3_, rt; (ii) Azetidine, STAB, THF, MeOH, 65 °C; (iii) HCl in Ether, rt

The prodrugs in Table **10** were all synthesized from compound **30** (Scheme 12 and Scheme 13). Compound **37** was made by the amidation of **30** using chloromethyl chloroformate, followed by an alkylation using isobutyric acid. Compound **38** began with the treatment of chloromethyl chloroformate with thioethane to afford **105**, before undergoing the alkylation of pivalic acid, and subsequent activation. Intermediate **107** was then used to couple to compound **30**. Compound **39** underwent a similar synthesis but required a Finkelstein reaction to improve the electrophilicity before undergoing the acylation.

**Scheme 12:**
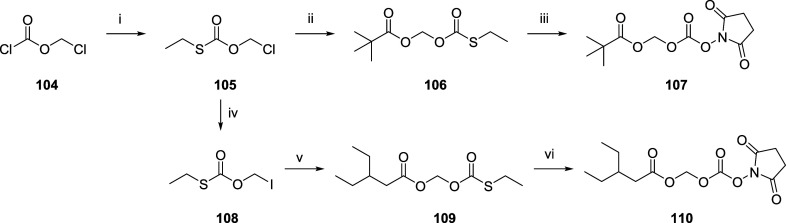
Synthesis of Intermediates **107** and **110**, towards Compounds **38** and **39**; (i) EtSH, Et_3_N, Et_2_O, 0 °C, 99%; (ii) Pivalic Acid, DIPEA, 60 °C; (iii) m-CPBA, 1-Hydroxypyrrolidine-2,5-dione, DCM, rt; (iv) NaHCO_3_, NaI, Acetone, 40 °C; (v) 3-Ethylpentanoic Acid, Tetrabutylammonium Hydrogensulfate, NaHSO_3_, DCM, Water; vi) m-CPBA, 1-Hydroxypyrrolidine-2,5-dione, CHCl_3_, 0 °C

**Scheme 13:**
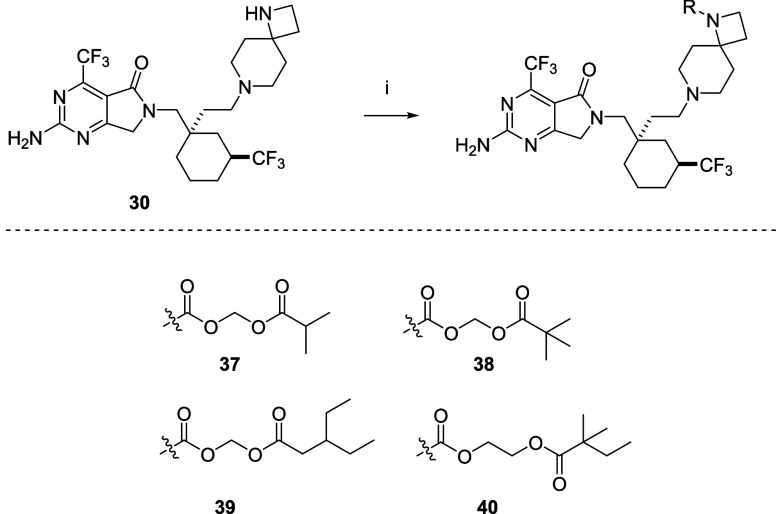
Synthesis of Compounds **37–40**; (i) Acid/Activated Acid; DIPEA, rt −110 °C, 6–31%

## Conclusions

In summary, we described here the hit identification, hit-to-lead and lead optimization phases for a new series of inhibitors of *Pf*KRS1. Structure-driven optimization of a micromolar hit with good ligand efficiency yielded potent leads with single digit nanomolar parasite growth inhibition and excellent biochemical and cellular selectivity (>1,000 fold). Neutral analogues from this series were efficacious in the *P. falciparum* SCID mouse model after oral dosing. We optimized selectivity, bioavailability and mitigated a CYP inhibition liability. However, neutral analogues suffered from a short half-life and were not suitable treatment of malaria infections with a single dose. With the aim of extending half-life by increasing volume of distribution, we introduced basic substituents and optimized their potency to the same levels as the neutral analogues (<10 nM). This optimization was supported by computational methods and structural information and led to compound **30,** a potent and selective lead with a long half-life across preclinical species. In addition, **30** was Ames negative and did not inhibit hERG significantly. However, compound **30** lacked oral bioavailability due to limited permeability, leading us to explore more lipophilic analogues and, at a later stage in the project, a prodrug approach to deliver this lead systemically after oral dosing.

Representatives from this series displayed activity across *Plasmodium* species, drug-resistant strains and field isolates. Compounds were active in asexual and sexual stages showing potential for treatment and transmission blocking. The risk of resistance for this series was manageable (log_10_ MIR = 8.6) and resistant clones confirmed on-target activity. Unfortunately, further development of this series was hampered by challenges in combining long half-life and bioavailability in a lead with potential for single low dose treatment of malaria.

Taken together, our work underscores the potential of inhibitors of lysyl-tRNA synthetase for treatment, chemoprotection and transmission-blocking of malaria infections. We envisage that learnings from this work will assist future drug discovery programs on new KRS1 inhibitors toward meeting the challenging requirements of the malaria target candidate profiles and deliver a clinical candidate with this novel mode of action.

## Experimental Section

### General Chemistry Methods

Solvents and reagents were purchased from commercial suppliers and used without further purification. Dry solvents were purchased in sure sealed bottles stored over molecular sieves. Unless otherwise stated herein, reactions have not been optimized. Yields refer to chromatographically and spectroscopically pure compounds. Column chromatography was performed using Combiflash Companion Rf (commercially available from Teledyne ISCO) and prepacked silica gel columns purchased from Teledyne ISCO. Mass-directed preparative HPLC separations were performed using a Waters HPLC (2545 binary gradient pumps, 515 HPLC make up pump, 2767 sample manager) connected to a Waters 2998 photodiode array and a Waters 3100 mass detector. Preparative HPLC separations were performed with a Gilson HPLC (321 pumps, 819 injection module, 215 liquid handler/injector) connected to a Gilson 155 UV/vis detector. On both instruments, HPLC chromatographic separations were conducted using Waters XBridge C18 columns, 19 × 100 mm, 5 um particle size; using 0.1% ammonia in water (solvent A) and acetonitrile (solvent B) or 0.1% formic acid in water (solvent A) and acetonitrile (solvent B) as mobile phase. Supercritical fluid chromatography (SFC) chiral separation was performed on a Waters SFC 350 using a Daicel Chiralpak column. ^1^H, ^13^C, and ^19^F NMR spectra were recorded on a Bruker Avance DPX 500 spectrometer (^1^H at 500.1 MHz, ^13^C at 125.8 MHz, ^19^F at 470.5 MHz), or a Bruker Avance DPX 400 (^1^H at 400 MHz, ^13^C at 100.6 MHz). Chemical shifts (δ) are expressed in parts per million (ppm), recorded using the residual solvent as the internal reference in all cases. Signal splitting patterns are described as singlet (s), doublet (d), triplet (t), quartet (q), multiplet (m), broad (br), or a combination thereof. Coupling constants (J) are quoted to the nearest 0.1 Hz. Low resolution electrospray (ES) mass spectra were recorded on an Agilent HPLC 1100 series connected to a Bruker Daltonics MicrOTOF; or an Agilent Technologies 1200 series HPLC connected to an Agilent Technologies 6130 quadrupole LC/MS, where both instruments were connected to an Agilent diode array detector; or a Shimadzu LCMS 2020 with dual ion source (ESI and APCI) conducted using a Thermo Fisher Hypersil Gold C18 column, 2.1 × 50 mm, 1.9 μm particle size; mobile phase, water/acetonitrile + 0.1% formic acid. All final compounds showed chemical purity of ≥95% as determined by the UV chromatogram (190–450 nm) obtained by LC–MS analysis.

#### Synthesis of 2-Amino-6-(cyclohexylmethyl)-6,7-dihydro-5*H*-pyrrolo­[3,4-*d*]­pyrimidin-5-one (**2**)

##### Step 1: Synthesis of Ethyl 2-Amino-4-(bromomethyl)­pyrimidine-5-carboxylate (**42**)

To a solution of ethyl 2-amino-4-methyl-pyrimidine-5-carboxylate (2370 mg, 13.08 mmol, 1.1 eq) in acetic acid (50 mL) was added bromine (1882 mg, 11.77 mmol, 1.0 equiv) dropwise at room temperature under nitrogen. The reaction mixture was stirred at 70 °C for 4 hours, then at room temperature for 16 hours. The mixture was filtered and rinsed with DCM followed by water to afford compound **42** (2109 mg, 62% yield) as a white solid, which was used in the subsequent step without further purification. LC-MS: *m*/*z* 262 (M+H)^+^

##### Step 2: Synthesis of 2-Amino-6-(cyclohexylmethyl)-6,7-dihydro-5*H*-pyrrolo­[3,4-*d*]­pyrimidin-5-one (**2**)

To a solution of cyclohexylmethanamine (131 mg, 1.15 mmol, 3 eq) in DMF (2 mL) was added ethyl 2-amino-4-(bromomethyl)­pyrimidine-5-carboxylate (100 mg, 0.38 mmol, 1 eq) in DMF (2 mL) dropwise at room temperature. The reaction was stirred at room temperature for 30 minutes, then lithium hydroxide monohydrate (32 mg, 0.77 mmol, 2 eq) in water (0.5 mL) was added and the reaction was stirred at room temperature for 16 hours. The reaction mixture was concentrated *in vacuo* and purified by column chromatography (0–10% MeOH/DCM) to afford compound **2** (34 mg, 34% yield) as a white solid. ^1^H NMR (400 MHz, DMSO-*d*_6_): δ 8.47 (s, 1H), 7.34 (s, 1H), 4.28 (s, 2H), 3.25 (d, J = 7.4 Hz, 2H), 1.74–1.52 (m, 6H), 1.26–1.09 (m, 3H), 0.99–0.84 (m, 2H). LC-MS: *m*/*z* 247 (M+H)^+^

#### Synthesis of 2-Amino-6-(cycloheptylmethyl)-7*H*-pyrrolo­[3,4-*d*]­pyrimidin-5-one (**3**)

To a solution of cycloheptylmethanamine (196 mg, 1.54 mmol, 2 equiv) and Et_3_N (0.64 mL, 4.613 mmol, 6 equiv) in NMP (2.6 mL) was added ethyl 2-amino-4-(bromomethyl)­pyrimidine-5-carboxylate (200 mg, 0.769 mmol, 1 equiv) portion-wise at room temperature, and the reaction was stirred at room temperature for 30 min. The temperature was increased to 130 °C for and stirring was continued for 2 h. The reaction mixture was concentrated *in vacuo* and was purified by reverse-phase chromatography (20–95% water/MeCN, acidic method) to afford compound **3** as a light-yellow solid (103 mg, 49% yield). ^1^H NMR (500 MHz, DMSO-*d*_6_): δ 8.48 (s, 1H), 7.32 (s, 2H), 4.27 (s, 2H), 3.25 (d, *J* = 7.6 Hz, 2H), 1.93–1.80 (m, 1H), 1.66–1.32 (m, 10H), 1.19–1.08 (m, 2H). LC-MS: *m*/*z* 261 (M+H)^+^

#### Synthesis of 2-Amino-6-[(4,4-difluoro-1-hydroxy-cyclohexyl)­methyl]-7*H*-pyrrolo­[3,4-*d*]­pyrimidin-5-one (**4**)

To a suspension of 1-(aminomethyl)-4,4-difluoro-cyclohexanol hydrochloride (1551 mg, 7.69 mmol, 2 equiv) and Et_3_N (2330 mg, 23.07 mmol, 6 equiv) in NMP (7 mL), was added ethyl 2-amino-4-(bromomethyl)­pyrimidine-5-carboxylate (1000 mg, 3.84 mmol, 1 equiv) portionwise. The reaction mixture was stirred at room temperature for 30 min, then was stirred under microwave irradiation at 130 °C for 1.5 h. The reaction mixture was concentrated *in vacuo* to afford a precipitate which was filtered and washed with DCM, water and methanol to afford compound **4** (440 mg, 38% yield) as a light pink solid. ^1^H NMR (500 MHz, DMSO-*d*_6_): δ 8.51 (s, 1H), 7.36 (s, 2H), 4.82 (s, 1H), 4.47 (s, 2H), 3.42 (s, 2H), 2.06–1.83 (m, 4H), 1.66–1.47 (m, 4H). LC-MS: *m*/*z* 299 (M+H)^+^

#### Synthesis of 6-Amino-2-(cyclohexylmethyl)-1-methyl-1,2-dihydro-3*H*-pyrazolo­[3,4-*d*]­pyrimidin-3-one (**5**)

##### Step 1: Synthesis of 1-Benzyl 2-(*tert*-butyl) 2-(Cyclohexylmethyl)-1-methylhydrazine-1,2-dicarboxylate (**44**)

To a solution of compound 2-benzyl 1-(*tert*-butyl) 1-(cyclohexylmethyl)­hydrazine-1,2-dicarboxylate (5 g, 13.79 mmol, 1 equiv) in THF (50 mL) was added NaH (662 mg, 16.55 mmol, 60% purity, 1.2 equiv) and MeI (43.81 g, 308.65 mmol, 19.21 mL, 22.38 equiv). After addition, the mixture was stirred at 20 °C for 2 h. The mixture was diluted with water (80 mL) and extracted with ethyl acetate (80 mL × 3). The combined organic layers were washed with washed with brine (60 × 2 mL), dried with anhydrous Na_2_SO_4_, filtered and concentrated *in vacuo*. Compound **44** (4.5 g, 10.52 mmol, 76.25% yield, 88% purity) was obtained as a colorless oil. ^1^H NMR (400 MHz, DMSO-*d*_6_): δ 7.41–7.26 (m, 5H), 5.32–4.93 (m, 1H), 5.32–4.93 (m, 1H), 3.32–3.04 (m, 3H), 3.02–2.96 (m, 2H), 1.64 (br d, *J* = 8.9 Hz, 5H), 1.42–1.36 (m, 4H), 1.28 (s, 5H), 1.21–0.75 (m, 6H). LC-MS: *m*/*z* 277 (M+H)^+^

##### Step 2: Synthesis of *tert*-Butyl 1-(Cyclohexylmethyl)-2-methylhydrazine-1-carboxylate (**45**, crude)

To a mixture of compound 44 (4.5 g, 11.95 mmol, 1 equiv) in MeOH (45 mL) was added Pd/C (0.5 g). The reaction was stirred at 20 °C for 12 h under H_2_ (50 psi). The reaction mixture was filtered and the filtrate was concentrated *in vacuo*. Compound **45** (2.2 g, 9.08 mmol, 76% yield) was used for next step directly.

##### Step 3: Synthesis of Ethyl 4-(2-(*tert*-Butoxycarbonyl)-2-(cyclohexylmethyl)-1-methylhydrazineyl)-2-chloropyrimidine-5-carboxylate (**46**, crude)

To a solution of ethyl 2,4-dichloropyrimidine-5-carboxylate (0.5 g, 2.06 mmol, 1 equiv) in MeCN (5 mL) was added compound 45 (684.03 mg, 3.09 mmol, 1.5 equiv) and DIPEA (533 mg, 4.13 mmol, 718.70 μL, 2 equiv). The mixture was stirred at room temperature for 12 h. The mixture was diluted with water (50 mL) and extracted with ethyl acetate (40 mL × 3). The combined organic layers were washed with washed with brine (50 × 2 mL), dried with anhydrous Na_2_SO_4_, filtered and concentrated *in vacuo*. Compound **46** (0.7 g, 1.64 mmol, 79% yield, colorless oil) was used for next step directly.

##### Step 4: Synthesis of Ethyl 4-(2-(*tert*-Butoxycarbonyl)-2-(cyclohexylmethyl)-1-methylhydrazineyl)-2-((3,4-dimethoxybenzyl)­amino)­pyrimidine-5-carboxylate (**47**)

To a solution of compound 46 (0.5 g, 1.17 mmol, 1 equiv) and (2,4-dimethoxyphenyl)­methanamine (215 mg, 1.29 mmol, 193.87 μL, 1.1 equiv) in DMF (5 mL) was added DIPEA (151 mg, 1.17 mmol, 204 μL, 1 equiv). The mixture was stirred at room temperature for 12 h. The mixture was diluted with water (40 mL) and extracted with ethyl acetate (40 mL × 3). The combined organic layers were washed with washed with brine (40 × 2 mL), dried with anhydrous Na_2_SO_4_, filtered and concentrated *in vacuo*. The residue was purified by column chromatography (5–50% EtOAc/heptane) to obtain compound **47** (0.31 g, 45% yield, 95% purity) as a colorless oil. ^1^H NMR (400 MHz, DMSO-*d*_6_): δ 8.37 (br s, 1H), 7.75 (br s, 1H), 7.10–6.79 (m, 1H), 6.52 (br s, 1H), 6.41 (br s, 1H), 4.17 (br s, 2H), 4.03 (q, *J* = 7.2 Hz, 1H), 3.78 (s, 3H), 3.70 (s, 3H), 3.04 (br s, 2H), 1.99 (s, 2H), 1.62 (br d, *J* = 15.9 Hz, 4H), 1.39 (br s, 6H), 1.25 (br s, 9H), 1.18–1.05 (m, 3H), 0.81 (br s, 2H). LC-MS: *m*/*z* 558 (M+H)^+^

##### Step 5: Synthesis of 6-Amino-2-(cyclohexylmethyl)-1-methyl-1,2-dihydro-3*H*-pyrazolo­[3,4-*d*]­pyrimidin-3-one (**5**)

A solution of compound 47 (0.31 g, 556 μmol, 1 equiv) in TFA (3 mL) was stirred at room temperature for 12 h. The reaction mixture was concentrated *in vacuo*. The residue was dissolved in MeOH, filtered and the filtrate was adjusted to pH 7 with saturated NaHCO_3_ solution. The mixture was filtered and the filter cake was concentrated *in vacuo*. Compound **5** (70 mg, 48% yield) was obtained as a white solid. ^1^H NMR (400 MHz, DMSO-*d*_6_): δ = 8.51 (s, 1H), 7.27 (br s, 2H), 3.59 (br d, *J* = 7.4 Hz, 2H), 3.27 (s, 3H), 1.67–1.41 (m, 6H), 1.17–1.05 (m, 3H), 0.96–0.83 (m, 2H). LC-MS: *m*/*z* 262 (M+H)^+^

#### Synthesis of 6-Amino-2-(cyclohexylmethyl)-1*H*-pyrrolo­[3,4-*c*]­pyridin-3-one (**6**)

##### Step 1: Synthesis of 6-Chloro-4-formyl-pyridine-3-carboxylic Acid (**51**)

To a solution of 2,2,6,6-tetramethylpiperidine (8.478 g, 60.02 mmol, 3 equiv) in THF (60 mL) was added *n*-BuLi in hexanes (1.6 M, 50 mL, 80 mmol, 4 equiv) at −78 °C. The reaction was allowed to warm to room temperature over 1 h, then cooled to −78 °C. A solution of 6-chloropyridine-3-carboxylic acid (3.151 g, 20 mmol, 1 equiv) in THF (20 mL) was added dropwise. The reaction was stirred at −78 °C for 1 h, then −50 °C for 30 min. The reaction was cooled to −78 °C and *N,N*-dimethylformamide (4 mL, 51.62 mmol, 2.6 equiv) was added dropwise. The reaction was stirred at −78 °C for 30 min, before being quenched with 2 M HCl and extracted with ethyl acetate (2 × 150 mL). The organic layer was dried with magnesium sulfate and concentrated *in vacuo* to afford the compound **51** (3.30 g), to be used as crude in the next step.

##### Step 2: Synthesis of 6-Chloro-2-(cyclohexylmethyl)-1*H*-pyrrolo­[3,4-*c*]­pyridin-3-one (**54**)

To a mixture of crude 6-chloro-4-formyl-pyridine-3-carboxylic acid (1.5 g, 8.08 mmol, 1 equiv), cyclohexylmethanamine (0.915 g, 8.08 mmol, 1 eq.) and acetic acid (2 mL) in DCM (5 mL), was added sodium triacetoxyborohydride (2.570 g, 12.13 mmol, 1.5 equiv) at room temperature. The reaction was stirred at room temperature for 3 days before being diluted with DCM and washed with 2 M aqueous NaOH. The organic layer was dried with magnesium sulfate and concentrated *in vacuo*. The crude material was purified by column chromatography (0–100% EtOAc/heptane) to afford compound **54** (580 mg, 26% yield) as a white solid. ^1^H NMR (500 MHz, DMSO-*d*_6_): δ 8.70 (d, *J* = 0.9 Hz, 1H), 7.81 (d, *J* = 0.9 Hz, 1H), 4.55 (s, 2H), 3.34 (d, *J* = 7.4 Hz, 2H), 1.74–1.56 (m, 6H), 1.24–1.10 (m, 3H), 0.99–0.88 (m, 2H). LC-MS: *m*/*z* 265 (M+H)^+^

##### Step 3: Synthesis of *tert*-Butyl *N*-[2-(Cyclohexylmethyl)-3-oxo-1*H*-pyrrolo­[3,4-*c*]­pyridin-6-yl]­carbamate (**58**)

A solution of 6-chloro-2-(cyclohexylmethyl)-1*H*-pyrrolo­[3,4-*c*]­pyridin-3-one (50 mg, 0.19 mmol, 1 equiv), *tert*-butyl carbamate (111 mg, 0.94 mmol, 5 equiv), Pd­(dba)_2_ (1 mg, 0.002 mmol, 0.01 equiv), Xantphos (2.2 mg, 0.004 mmol, 0.02 eq.) and Cs_2_CO_3_ (308 mg, 0.944 mmol, 5 equiv) in THF (3 mL) was stirred at reflux for 6 h. The reaction was allowed to cool to room temperature, filtered through Celite and washed with ethyl acetate. The solution was washed with saturated sodium bicarbonate, dried with magnesium sulfate, and purified by column chromatography (0–100% ethyl acetate/heptane) to afford compound **58** (10 mg, 14% yield) as an off-white solid. ^1^H NMR (500 MHz, DMSO-*d*_6_): δ 10.16 (s, 1H), 8.52 (d, *J* = 1.0 Hz, 1H), 8.01 (d, *J* = 0.9 Hz, 1H), 4.49 (s, 2H), 1.72–1.55 (m, 6H), 1.49 (s, 9H), 1.24–1.10 (m, 3H), 0.99–0.88 (m, 2H). LC-MS: *m*/*z* 346 (M+H)^+^

##### Step 4: Synthesis of 6-Amino-2-(cyclohexylmethyl)-1*H*-pyrrolo­[3,4-*c*]­pyridin-3-one (**6**)

To a solution of *tert*-butyl *N*-[2-(cyclohexylmethyl)-3-oxo-1*H*-pyrrolo­[3,4-*c*]­pyridin-6-yl]­carbamate (70 mg, 0.19 mmol, 1 equiv) in DCM (3 mL) was added trifluoroacetic acid (2 mL, 26.14 mmol, 139 equiv) at 0 °C. The reaction was allowed to warm to room temperature and stirred for 1 h. The reaction was concentrated *in vacuo*, and purified by reverse phase column chromatography (5–95% MeCN/water with 0.1% ammonia buffer) to afford compound **6** (20 mg, 42% yield) as a white solid. ^1^H NMR (500 MHz, DMSO-*d*_6_): δ 8.20 (s, 1H), 6.54–6.38 (m, 3H), 4.31 (s, 2H), 3.24 (d, *J* = 7.3 Hz, 2H), 1.69–1.54 (m, 6H), 1.24–1.11 (m, 3H), 0.96–0.85 (m, 2H). LC-MS: *m*/*z* 246 (M+H)^+^

#### Synthesis of 2-Amino-6-((4,4-difluorocyclohexyl)­methyl)-4-fluoro-6,7-dihydro-5*H*-pyrrolo­[3,4-*d*]­pyrimidin-5-one (**7**)

##### Step 1: Synthesis of 4,6-Difluoro-1-hydroxyfuro­[3,4-*c*]­pyridin-3­(1*H*)-one (52)

To a solution of tetramethylpiperidine (61.72 g, 437 mmol, 3.16 equiv) in THF (374 mL) was added *n*-BuLi (2.5 M, 166 mL, 3 equiv) dropwise under N_2_ at −50 °C, the mixture was stirred at −50 °C for 0.5 h and then was added a solution of 2,6-difluoronicotinic acid (22 g, 138.29 mmol, 1 equiv) in THF (110 mL). The mixture was stirred at −50 °C for another 0.5 h, treated with DMF (50.54 g, 691.43 mmol, 53.20 mL, 5 equiv) at −50 °C for 0.5 h and warmed to −20 °C for 0.5 h. The mixture was warmed to 0 °C for 0.5 h and further up to 25 °C for another 0.5 h. The mixture was poured into ice-H_2_O (1 L) then added 4 N HCl (80 mL) and extracted with ethyl acetate (800 mL × 3). The combined organic layers were washed with washed with brine (800 × 2 mL), dried with anhydrous Na_2_SO_4_, filtered and concentrated *in vacuo*. The reaction was purified by flash chromatography (0–100% EtOAc/heptane) to afford compound **52** (16 g, 76.96 mmol, 56% yield, 90% purity) as a yellow oil. LC-MS: *m*/*z* 188 (M+H)^+^

##### Step 2: Synthesis of 6-((4,4-Difluorocyclohexyl)­methyl)-2,4-difluoro-6,7-dihydro-5*H*-pyrrolo­[3,4-*d*]­pyrimidin-5-one (**55**)

To a solution of compound 52 (1 g, 5.34 mmol, 1 equiv) and 1-(aminomethyl)-4,4-difluorocyclohexanol (1.06 g, 6.41 mmol, 1.20 equiv) in DCM (10 mL) was added AcOH (2.14 mL, 37.41 mmol, 7 equiv) and NaBH­(OAc)_3_ (2.83 g, 13.36 mmol, 2.5 equiv). After addition, the mixture was stirred at 40 °C for 16 h. The mixture was diluted with water (80 mL) and extracted with ethyl acetate (80 mL × 3). The combined organic layers were washed with washed with brine (60 × 2 mL), dried with anhydrous Na_2_SO_4_, filtered and concentrated in *vacuo*. The residue was purified by flash chromatography (0–100% EtOAc/heptane) to afford compound **55** (0.75 g, 2.36 mmol, 44% yield) as a yellow solid. LC-MS: *m*/*z* 319 (M+H)^+^

##### Step 3: Synthesis of 6-((4,4-Difluorocyclohexyl)­methyl)-2-((3,4-dimethylbenzyl)­amino)-4-fluoro-6,7-dihydro-5*H*-pyrrolo­[3,4-*d*]­pyrimidin-5-one (**59**)

To a solution of compound 55 (1.4 g, 4.40 mmol,1 equiv) in DMF (13 mL) was added (2,4-dimethoxyphenyl)­methanamine (662 μL, 4.40 mmol, 1 equiv) and DIPEA (1.53 mL, 8.80 mmol, 2 equiv). The mixture was stirred at 40 °C for 2 h. The mixture was diluted with water (80 mL) and extracted with ethyl acetate (80 mL × 3). The combined organic layers were washed with washed with brine (60 × 2 mL), dried with anhydrous Na_2_SO_4_, filtered and concentrated *in vacuo.* The residue was purified by flash chromatography (0–100% EtOAc/heptane) to afford compound **59** (0.110 g, 2.7% yield, 62% purity) was obtained as a yellow solid. LC-MS: *m*/*z* 466 (M+H)^+^

##### Step 4: Synthesis of 2-Amino-6-((4,4-difluorocyclohexyl)­methyl)-4-fluoro-6,7-dihydro-5*H*-pyrrolo­[3,4-*d*]­pyrimidin-5-one (**7**)

A solution of compound 59 (0.110 g, 236 μmol, 1 eq.) in TFA (2 mL) was stirred at room temperature for 12 h. The reaction mixture was concentrated *in vacuo*. The residue was purified by reverse phase chromatography (0–42% ammonia/water with ammonia buffer) to afford compound **7** (11 mg, 14% yield) as a white solid. ^1^H NMR 400 MHz DMSO-*d*_6_: δ 6.94 (s, 2H), 6.38 (d, *J* = 2.5 Hz, 1H), 4.83 (br s, 1H), 4.54 (s, 2H), 2.52 (d, *J* = 1.8 Hz, 2H), 2.09–1.81 (m, 4H), 1.64–1.45 (m, 4H). LC-MS: *m*/*z* 316 (M+H)^+^

#### Synthesis of 6-Amino-2-[(4,4-difluoro-1-hydroxy-cyclohexyl)­methyl]-4-(trifluoromethyl)-1*H*-pyrrolo­[3,4-*c*]­pyridin-3-one (**8**)

##### Step 1: Synthesis of 6-Chloro-1-hydroxy-4-(trifluoromethyl)-1*H*-furo­[3,4-*c*]­pyridin-3-one (**53**)

To a stirred solution of 2,2,6,6-tetramethylpiperidine (2505 mg, 17.73 mmol, 4 equiv) in THF (10 mL), *n*-BuLi in hexanes (11.1 mL, 17.73 mmol, 4 equiv) was added dropwise at −78 °C. The reaction was warmed to 0 °C for 15 min, then cooled at −78 °C. A solution of 6-chloro-2-(trifluoromethyl)­pyridine-3-carboxylic acid (1 g, 4.43 mmol, 1 equiv) in THF (15 mL) was added dropwise at −78 °C and the reaction was stirred for 1.5 h (warming to −50 °C). *N,N*-Dimethylformamide (1.7 mL, 22.17 mmol) was added dropwise at −78 °C and the reaction was stirred for 30 min at −78 °C and then stirred at −50 °C for 1 h. The reaction mixture was quenched with 2 M HCl and extracted with ethyl acetate. The organic layer was dried with MgSO_4_ and concentrated *in vacuo* to afford compound **53** as a yellow gum, to be used directly in a subsequent reaction. LC-MS: *m*/*z* 254 (M+H)^+^

##### Step 2: Synthesis of 6-Chloro-2-[(4,4-difluoro-1-hydroxy-cyclohexyl)­methyl]-4-(trifluoromethyl)-1*H*-pyrrolo­[3,4-*c*]­pyridin-3-one (56)

A suspension of crude 6-chloro-1-hydroxy-4-(trifluoromethyl)-1*H*-furo­[3,4-*c*]­pyridin-3-one (4.5 g, 17.75 mmol, 1 equiv) in DCM (20 mL) and a suspension of 1-(aminomethyl)-4,4-difluoro-cyclohexanol hydrochloride (3579 mg, 17.75 mmol, 1 equiv) and *N,N*-diethylethanamine (2.47 mL, 17.75 mmol, 3 equiv) in DCM (20 mL) were mixed. Sodium triacetoxyborohydride (11284 mg, 53.24 mmol, 3 equiv) and acetic acid (8 mL) were added to the reaction mixture in an ice bath. The reaction was allowed to reach room temperature and stirred at room temperature for 40 h. The reaction was diluted with DCM and basified with 2 M NaOH to pH 8. The organic layer was separated, dried with MgSO_4_, and concentrated *in vacuo*. The crude material was purified using column chromatography (0–100% EtOAc/heptane) to afford compound **56** (1866 mg, 26% yield) as a gray solid. ^1^H NMR (400 MHz, DMSO-*d*_6_): δ 8.21 (s, 1H), 4.92 (s, 1H), 4.80 (s, 2H), 3.52 (s, 2H), 2.12–1.80 (m, 4H), 1.75–1.45 (m, 4H). LC-MS: *m*/*z* 385 (M+H)^+^

##### Step 3: Synthesis of 2-[(4,4-Difluoro-1-hydroxy-cyclohexyl)­methyl]-6-[(2,4-dimethoxyphenyl)­methylamino]-4-(trifluoromethyl)-1*H*-pyrrolo­[3,4-*c*]­pyridin-3-one (**60**)

To a solution of 6-chloro-2-[(4,4-difluoro-1-hydroxy-cyclohexyl)­methyl]-4-(trifluoromethyl)-1*H*-pyrrolo­[3,4-*c*]­pyridin-3-one (300 mg, 0.78 mmol, 1 equiv) in DMA (3 mL), (2,4-dimethoxyphenyl)­methanamine (266 mg, 1.56 mmol, 2 equiv) and DIPEA (0.28 mL, 1.62 mmol, 2 equiv) were added and the reaction was stirred at 120 °C overnight. The reaction mixture was partitioned between a 10% aqueous solution of LiCl and ethyl acetate. The organic layer was dried with Na_2_SO_4_ concentrated *in vacuo* and purified by column chromatography (0–100% EtOAc/heptane) to afford compound **60** (200 mg, 40% yield) as a yellow wax. ^1^H NMR (400 MHz, DMSO-*d*_6_): δ 7.81 (t, *J* = 5.5 Hz, 1H), 7.16 (d, *J* = 8.4 Hz, 1H), 6.79 (s, 1H), 6.56 (d, *J* = 2.4 Hz, 1H), 6.46 (dd, *J* = 8.4, 2.4 Hz, 1H), 4.82 (s, 1H), 4.56 (s, 2H), 4.41 (d, *J* = 5.3 Hz, 2H), 3.80 (s, 3H), 3.73 (s, 3H), 3.41 (s, 2H), 2.07–1.85 (m, 4H), 1.64–1.48 (m, 4H). LC-MS: *m*/*z* 516 (M+H)^+^

##### Step 4: Synthesis of 6-Amino-2-[(4,4-difluoro-1-hydroxy-cyclohexyl)­methyl]-4-(trifluoromethyl)-1*H*-pyrrolo­[3,4-*c*]­pyridin-3-one (**8**)

To a suspension of 2-[(4,4-difluoro-1-hydroxy-cyclohexyl)­methyl]-6-[(2,4-dimethoxyphenyl)­methylamino]-4-(trifluoromethyl)-1*H*-pyrrolo­[3,4-*c*]­pyridin-3-one (820 mg, 1.59 mmol, 1 equiv) in DCM (25 mL) was added trifluoroacetic acid (4.38 mL, 57 mmol, 36 equiv) at 0 °C and the reaction was stirred at room temperature for 3 h. The reaction mixture was concentrated *in vacuo*, and the crude material was dissolved in methanol (30 mL) and 7 M NH_3_ in methanol (5 mL) was added. The suspension was stirred for 10 min at room temperature, then was concentrated *in vacuo*. The crude material was dissolved in DCM and washed with water, and the organic layer was dried with magnesium sulfate and concentrated *in vacuo*. The resulting material was purified by reverse phase column chromatography (5–60% MeCN/water with 0.1% formic acid buffer) to afford compound **8** (343 mg, 58% yield) as a white solid. ^1^H NMR (500 MHz, DMSO-*d*_6_): δ 7.02 (s, 2H), 6.73 (s, 1H), 4.82 (s, 1H), 4.57 (s, 2H), 3.41 (s, 2H), 2.06–1.85 (m, 4H), 1.65–1.48 (m, 4H). LC-MS: *m*/*z* 366 (M+H)^+^

#### Synthesis of 6-Amino-2-[(3,3-difluoro-1-hydroxy-cyclohexyl)­methyl]-4-(trifluoromethyl)-1*H*-pyrrolo­[3,4-*c*]­pyridin-3-one (**9**)

##### Step 1: Synthesis of 6-Chloro-4-formyl-2-(trifluoromethyl)­nicotinic Acid (**57**, crude)

To a suspension of 1-(aminomethyl)-3,3-difluoro-cyclohexanol hydrochloride (3777 mg, 18.73 mmol, 1 equiv) and *N,N*-diethylethanamine (0.137 mL, 18.73 mmol, 1 equiv) in DCM (25 mL) was added compound 53 (4750 mg, 18.73 mmol, 1 equiv) in DCM (25 mL) at room temperature. The reaction was cooled to 0 °C and sodium triacetoxyborohydride (14889 mg, 56.2 mmol, 3 equiv) and acetic acid (10 mL) were added. The reaction mixture was subsequently allowed to warm to room temperature and stirred for 30 min at room temperature. The reaction mixture was concentrated *in vacuo,* and taken up in MeCN (50 mL), and the reaction was stirred at 75 °C for 30 min. The reaction mixture was concentrated *in vacuo* and used in the subsequent reaction as crude material.

##### Step 2: Synthesis of 2-[(3,3-Difluoro-1-hydroxy-cyclohexyl)­methyl]-6-[(2,4-dimethoxyphenyl)­methylamino]-4-(trifluoromethyl)-1*H*-pyrrolo­[3,4-*c*]­pyridin-3-one**(61)**

A solution of crude 6-chloro-2-[(3,3-difluoro-1-hydroxy-cyclohexyl)­methyl]-4-(trifluoromethyl)-1*H*-pyrrolo­[3,4-*c*]­pyridin-3-one (3000 mg, 7.79 mmol, 1 equiv), DIPEA (2.85 mL, 16.38 mmol, 2.1 equiv), (2,4-dimethoxyphenyl)­methanamine (2.44 mL, 15.595 mmol, 2 eq.) and potassium fluoride (1359 mg, 23.39 mmol, 3 equiv) in DMSO (30 mL) was stirred at 80 °C for 3 h. The reaction was diluted with DCM and washed with brine, and the organic layer was dried with magnesium sulfate and concentrated *in vacuo*. The crude material was purified by column chromatography (55% EtOAc/heptane, isocratic) to afford compound **61** as an off-white solid (509 mg, 13% yield). LC-MS: *m*/*z* 516 (M+H)^+^

##### Step 3: Synthesis of 6-Amino-2-[(3,3-difluoro-1-hydroxy-cyclohexyl)­methyl]-4-(trifluoromethyl)-1*H*-pyrrolo­[3,4-*c*]­pyridin-3-one (**9**)

To a solution of 2-[(3,3-difluoro-1-hydroxy-cyclohexyl)­methyl]-6-[(2,4-dimethoxyphenyl)­methylamino]-4-(trifluoromethyl)-1*H*-pyrrolo­[3,4-*c*]­pyridin-3-one (500 mg, 0.97 mmol, 1 eq.) in DCM (6 mL) was added trifluoroacetic acid (3 mL, 38.8 mmol, 40 eq.) and the reaction was stirred for 1 h at room temperature. The reaction was concentrated *in vacuo* and the crude material was dissolved in methanol and applied to an SCX cartridge. The cartridge was rinsed with methanol and the product eluted with 7 M NH_3_ in methanol. The mixture was concentrated *in vacuo* and further purified using reverse phase chromatography (5–95% MeCN/water with 0.1% formic acid buffer) to afford compound **9** as a white solid (103 mg, 29% yield). ^1^H NMR (500 MHz, DMSO-*d*_6_): δ 6.99 (s, 2H), 6.72 (s, 1H), 4.73 (s, 1H), 4.61 (d, *J* = 18.4 Hz, 1H), 4.51 (d, *J* = 18.4 Hz, 1H), 3.60 (d, *J* = 14.0 Hz, 1H), 3.24 (d, *J* = 14.1 Hz, 1H), 2.01–1.67 (m, 5H), 1.67–1.58 (m, 1H), 1.51–1.41 (m, 2H). LC-MS: *m*/*z* 366 (M+H)^+^

#### Synthesis of 2-Amino-6-[(3,3-difluoro-1-hydroxy-cyclohexyl)­methyl]-4-(trifluoromethyl)-7*H*-pyrrolo­[3,4-*d*]­pyrimidin-5-one (**10**)

##### Step 1: Synthesis of Ethyl 2-Amino-4-methyl-6-(trifluoromethyl)­pyrimidine-5-carboxylate (**62**)

To a stirred solution of guanidine hydrochloride (40.0 g, 418.78 mmol, 1.1 equiv) in acetonitrile (350 mL), was added sodium hydroxide (63 g, 158 mmol, 4.1 equiv) and the reaction mixture was stirred at room temperature for 16 h. After that, ethyl 3-oxobutanoate (50 g, 384.2 mmol, 1 equiv) was added, then pentafluoroethyl iodide (103 g, 418.7 mmol, 1.1 equiv) gas was purged slowly with temperature control to keep the internal temperature below 5 °C. A blue light was used to initiate the reaction for 40 min and the reaction was stirred at room temperature for 16 h. After completion, the reaction mixture was diluted with water and extracted with ethyl acetate, the combined organic layer was dried over sodium sulfate, filtered and concentrated *in vacuo*. The crude product was purified by column chromatography (20% EtOAc in hexane) to afford compound **62** (29 g, 30% yield) as yellow sticky gum. ^1^H NMR (400 MHz, CDCl_3_) δ 5.54 (s, 2H), 4.38 (q, *J* = 7.2 Hz, 2H), 2.45 (s, 3H), 1.33 (t, *J* = 7.2 Hz, 3H) ppm. LCMS: *m*/*z* = 250 [M+H]^+^.

##### Step 2: Synthesis of Ethyl 2-Amino-4-(bromomethyl)-6-(trifluoromethyl)­pyrimidine-5-carboxylate (**63**)

To a solution of ethyl 2-amino-4-methyl-6-(trifluoromethyl)­pyrimidine-5-carboxylate (1704 mg, 6.84 mmol, 1 equiv) in chloroform (50 mL) and acetic acid (0.001 mL), bromine (1421 mg, 8.89 mmol, 1.3 equiv) was added dropwise. The mixture was stirred at 60 °C overnight then washed with 10% Na_2_S_2_O_3_ aqueous solution before drying the organic layer with sodium sulfate. The crude material was concentrated *in vacuo* and purified by column chromatography (0–20% EtOAc/heptane) to afford compound **63** (1155 mg, 51% yield) as a white solid. LC-MS: *m*/*z* 329 (M+H)^+^

##### Step 3: Synthesis of 2-Amino-6-[(3,3-difluoro-1-hydroxy-cyclohexyl)­methyl]-4-(trifluoromethyl)-7*H*-pyrrolo­[3,4-*d*]­pyrimidin-5-one (10)

To a solution of 1-(aminomethyl)-3,3-difluoro-cyclohexanol hydrochloride (492 mg, 2.44 mmol, 2 equiv) and Et_3_N (1.02 mL, 7.32 mmol, 6 equiv) in MeCN (2 mL), ethyl 2-amino-4-(bromomethyl)-6-(trifluoromethyl)­pyrimidine-5-carboxylate (400 mg, 1.22 mmol, 1 equiv) was added portionwise and the solution stirred for 30 min at room temperature. The temperature was increased to 60 °C and stirring was continued for 2 h. The reaction mixture was concentrated *in vacuo*, DCM was added, and the suspension was washed with water. The organic later was concentrated *in vacuo*, DCM was added, and the precipitate was washed with DCM, followed by methanol, to afford compound **10** (137 mg, 30 mg) as a white solid. ^1^H NMR (500 MHz, DMSO-*d*_6_): δ 7.93 (d, *J* = 23.4 Hz, 2H), 4.73 (s, 1H), 4.53 (q, *J* = 18.9 Hz, 2H), 3.61 (d, *J* = 14.0 Hz, 1H), 3.26 (d, *J* = 14.1 Hz, 1H), 2.01–1.56 (m, 6H), 1.54–1.41 (m, 2H). LC-MS: *m*/*z* 367 (M+H)^+^

#### Synthesis of *trans*-2-Amino-6-((1-hydroxy-3-(trifluoromethyl)­cyclohexyl)­methyl)-4-(trifluoromethyl)-6,7-dihydro-5*H*-pyrrolo­[3,4-*d*]­pyrimidin-5-one (**11**)

##### Step 1: Synthesis of 1-(Aminomethyl)-3-(trifluoromethyl)­cyclohexanol (**66**, via **65**)

A mixture of trimethylsulfoxonium iodide (620 mg, 2.82 mmol, 1.3 equiv) and sodium hydride (113 mg, 2.82 mmol, 1.3 equiv) was prepared at room temperature and cooled to 0 °C under nitrogen. DMSO (20 mL) was added dropwise over 5 min and the mixture allowed to warm to room temperature. After stirring for 1 h at room temperature, 3-(trifluoromethyl)­cyclohexan-1-one (360 mg, 2.167 mmol, 1 equiv) in DMSO was added dropwise and the reaction mixture stirred overnight at room temperature. The reaction was quenched with water and extracted with Et_2_O. The combined organics were then washed with brine, before being dried with sodium sulfate and concentrated *in vacuo* to afford a yellow oil. The crude material was diluted with ammonia in methanol (7.13 mL, 49.90 mmol, 23 equiv) and heated in a microwave at 110 °C for 30 min. The material was concentrated *in vacuo* and applied to an SCX cartridge and rinsed with methanol. Next, the cartridge was washed with 7 M ammonia in methanol and the resulting fraction was concentrated *in vacuo* to afford compound **66** (350 mg, 78% yield) as a yellow oil. ^1^H NMR (500 MHz, CDCl_3_) δ 2.61–2.44 (m, 2H), 2.03–1.90 (m, 2H), 1.87–1.68 (m, 2H), 1.66–1.53 (m, 1H), 1.36–1.06 (m, 4H), 0.94–0.76 (m, 1H).

##### Step 2: Synthesis of *trans*-2-Amino-6-((1-hydroxy-3-(trifluoromethyl)­cyclohexyl)­methyl)-4-(trifluoromethyl)-6,7-dihydro-5*H*-pyrrolo­[3,4-*d*]­pyrimidin-5-one (11)

To a solution of compound 66 (214 mg, 0.91 mmol, 1.5 equiv) and triethylamine (0.51 mL, 3.66 mmol, 6 equiv) in MeCN (2 mL) was added compound 63 (200 mg, 0.61 mmol, 1 equiv) portion wise. The reaction was stirred at room temperature for 12 h then the reaction was concentrated *in vacuo*. The residue was dissolved in DCM and washed with water and sat. aqueous NH_4_Cl, then dried with sodium sulfate and concentrated *in vacuo.* The crude material was purified by column chromatography to (0–100% EtOAc/heptane) to afford compound **11** (77 mg, 31% yield) as a white solid. ^1^H NMR (500 MHz, DMSO-*d*_6_): δ 7.92 (d, *J* = 24.6 Hz, 2H), 4.69 (s, 1H), 4.53 (s, 2H), 3.40 (s, 2H), 1.80 (d, *J* = 10.9 Hz, 1H), 1.72 (d, *J* = 12.9 Hz, 1H), 1.64–1.49 (m, 3H), 1.33–1.11 (m, 4H). LC-MS: *m*/*z* 399 (M+H)^+^

#### Synthesis of *trans*-6-Amino-2-((1-hydroxy-3-(trifluoromethyl)­cyclohexyl)­methyl)-4-(trifluoromethyl)-1,2-dihydro-3*H*-pyrrolo­[3,4-*c*]­pyridin-3-one (**12**)

Single enantiomer separated from compound **11** using supercritical fluid chromatography. Unknown absolute configuration. >99% ee. ^1^H NMR (400 MHz, DMSO-*d*_6_): δ 7.92 (s, 2H), 4.71 (s, 1H), 4.53 (s, 2H), 3.40 (s, 2H), 2.47–2.35 (m, 1H), 1.80 (d, *J* = 11.7 Hz, 1H), 1.72 (d, *J* = 12.5 Hz, 1H), 1.65–1.50 (m, 3H), 1.35–1.22 (m, 2H), 1.22–1.10 (m, 1H). LC-MS: *m*/*z* 399 (M+H)^+^

#### Synthesis of *trans*-6-Amino-2-((1-hydroxy-3-(trifluoromethyl)­cyclohexyl)­methyl)-4-(trifluoromethyl)-1,2-dihydro-3*H*-pyrrolo­[3,4-*c*]­pyridin-3-one (**13**)

Single enantiomer separated from compound **11** using supercritical fluid chromatography. Unknown absolute configuration. >99% ee. ^1^H NMR (400 MHz, DMSO-*d*_6_) δ 7.92 (s, 2H), 4.70 (s, 1H), 4.53 (s, 2H), 3.40 (s, 2H), 2.48–2.40 (m, 1H), 1.80 (d, *J* = 12.0 Hz, 1H), 1.72 (d, *J* = 12.8 Hz, 1H), 1.66–1.48 (m, 3H), 1.29 (t, *J* = 12.9 Hz, 2H), 1.17 (ddd, *J* = 23.8, 14.8, 9.3 Hz, 1H). LC-MS: *m*/*z* 399 (M+H)^+^

#### Synthesis of *cis*-6-amino-2-((1-hydroxy-3-(trifluoromethyl)­cyclohexyl)­methyl)-4-(trifluoromethyl)-1,2-dihydro-3*H*-pyrrolo­[3,4-*c*]­pyridin-3-one (**14**)

Single enantiomer separated from compound **11** using supercritical fluid chromatography. Unknown absolute configuration. >99% ee. ^1^H NMR (400 MHz, DMSO-*d*_6_): δ 7.93 (s, 2H), 4.91 (s, 1H), 4.61 (d, *J* = 19.1 Hz, 1H), 4.49 (d, *J* = 19.2 Hz, 1H), 3.70 (d, *J* = 14.3 Hz, 1H), 3.31 (d, *J* = 17.6 Hz, 1H), 2.74–2.57 (m, 1H), 1.85–1.65 (m, 4H), 1.61–1.44 (m, 1H), 1.34–1.04 (m, 3H). LC-MS: *m*/*z* 399 (M+H)^+^

#### Synthesis of *cis*-6-amino-2-((1-hydroxy-3-(trifluoromethyl)­cyclohexyl)­methyl)-4-(trifluoromethyl)-1,2-dihydro-3*H*-pyrrolo­[3,4-*c*]­pyridin-3-one (**15**)

Single enantiomer separated from compound **11** using supercritical fluid chromatography. Unknown absolute configuration. >99% ee. ^1^H NMR (400 MHz, DMSO-*d*_6_): δ 7.93 (s, 2H), 4.91 (s, 1H), 4.61 (d, *J* = 19.2 Hz, 1H), 4.49 (d, *J* = 19.2 Hz, 1H), 3.70 (d, *J* = 14.3 Hz, 1H), 3.30 (d, *J* = 26.9 Hz, 1H), 2.72–2.55 (m, 1H), 1.89–1.65 (m, 4H), 1.61–1.45 (m, 1H), 1.38–1.05 (m, 3H). LC-MS: *m*/*z* 399 (M+H)^+^

#### Synthesis of *trans*-2-Amino-4-(difluoromethyl)-6-1-hydroxy-3-(trifluoromethyl)­cyclohexyl)­methyl)-6,7-dihydro-5*H*-pyrrolo­[3,4-*d*]­pyrimidin-5-one (**16**)

##### Step 1: Synthesis of *tert*-Butyl­(*tert*-butoxycarbonyl)­(4,6-dichloropyrimidin-2-yl)­carbamate (**68**)

A mixture of 4,6-dichloropyrimidin-2-amine (5 g, 30.49 mmol, 1 equiv), (Boc)_2_O (13.31 g, 60.98 mmol, 2 equiv) and DMAP (745 mg, 6.10 mmol, 0.2 equiv) in THF (25 mL) was stirred at 10 °C for 0.5 h. The mixture was diluted with water, then the aqueous layer was extracted with ethyl acetate (3×), and the combined organic layer was washed with brine (25 mL), dried over Na_2_SO_4_, filtered and concentrated *in vacuo* to afford the crude product which was purified by flash chromatography (ethyl acetate/petroleum ether 5–25%) to afford compound **68** (8.5 g, 77% yield) as a white solid.

##### Step 2: Synthesis of Ethyl 2-(bis­(*tert*-Butoxycarbonyl)­amino)-4,6-dichloropyrimidine-5-carboxylate (**69**)

To a solution of Compound 68 (8.5 g, 23.34 mmol, 1 equiv) in THF (90 mL) was added LDA (2 M, 23.34 mL, 2 equiv) at −78 °C under N_2_, and the mixture was stirred at −78 °C for 0.5 h before ethyl carbonochloridate (3.33 mL, 35.01 mmol, 1.5 equiv) was added dropwise, and the mixture was stirred at −78 °C for 0.5 h, then warmed to 10 °C for 1 h. The reaction was quenched with sat. NH_4_Cl (50 mL), then extracted with ethyl acetate (50 mL × 3), the combined organic layer was washed with brine (50 mL), dried over Na_2_SO_4_, filtered and concentrated *in vacuo*. The crude product was purified by flash chromatography chromatography (ethyl acetate/petroleum ether 9–17%) to afford compound **69** (3.2 g, 24% yield, 75% purity) was obtained as a yellow solid. ^1^H NMR: 400 MHz, CDCl_3_: δ = 4.49 (q, J = 7.2 Hz, 2H), 1.55–1.49 (m, 18H), 1.43 (t, J = 7.2 Hz, 3H)

##### Step 3: Synthesis of Ethyl 2-(bis­(*tert*-Butoxycarbonyl)­amino)-4-chloro-6-vinylpyrimidine-5-carboxylate (**70**)

To a solution of Compound 69 (30 g, 68.76 mmol, 1 equiv), Pd­(dppf)­Cl_2_ (5.03 g, 6.88 mmol, 0.1 equiv), Cs_2_CO_3_ (44.81 g, 138 mmol, 2 equiv) and potassium vinyltrifluoroborate (11.97 g, 89 mmol, 1.3 equiv) in dioxane (300 mL) and Water (30 mL), then the mixture was stirred at 90 °C for 3 h. The mixture was diluted with water (100 mL), then extracted with EtOAc (100 mL × 3), and the combined organic layers were washed with brine (100 mL), dried over Na_2_SO_4_, filtered and concentrated *in vacuo*. The crude product was purified by column chromatography (20% ethyl acetate in petroleum ether, isocratic) to afford Compound **70** (9 g, 28% yield, 90% purity) as a yellow solid. LCMS: *m*/*z* 272 (M+H)^+^

##### Step 4: Synthesis of Ethyl 2-(bis­(*tert*-Butoxycarbonyl)­amino)-4-chloro-6-formylpyrimidine-5-carboxylate (**71**)

NaIO_4_(6.00 g, 28.05 mmol, 3 equiv) was added to a solution of compound 70 (4.00 g, 9.35 mmol, 1 equiv) in dioxane (120 mL) and H_2_O (40 mL) at 20 °C, followed by the addition of OsO_4_ (12.13 μL, 234 μmol, 0.025 equiv). The mixture was stirred at room temperature for 1 h. Water (50 mL) was added, and the mixture was extracted with ethyl acetate (3 × 50 mL). The combined organic layers were washed with brine (100 mL), dried over Na_2_SO_4_ filtered, and concentrated to afford crude compound **71** (4.00 g) as a brown oil.

##### Step 5: Synthesis of Ethyl 2-(bis­(*tert*-Butoxycarbonyl)­amino)-4-chloro-6-(difluoromethyl)­pyrimidine-5-carboxylate (**72**)

A solution of Compound 71 (25 g, 58.16 mmol, 1 equiv) in dichloromethane (250 mL) was treated with DAST (37.50 g, 232.64 mmol, 30.74 mL, 4 equiv) at 0 °C. The reaction mixture was stirred at room temperature for 15 h, then quenched with water (100 mL) and extracted with dichloromethane (3 × 100 mL). The combined organic layers were washed with brine (100 mL), dried over Na_2_SO_4_ filtered, and concentrated *in vacuo*. The crude product was purified by flash chromatography (5–20% ethyl acetate in petroleum ether) to afford Compound **72** (24 g, 47.27 mmol, 81.3% yield, 89% purity) as a brown oil. ^1^H NMR (400 MHz, CDCl_3_) δ 6.84–6.45 (m, 1H), 4.55–4.41 (m, 2H), 1.51 (s, 16H), 1.42 (t, J = 7.2 Hz, 3H).

##### Step 6: Synthesis of Ethyl 2-(bis­(*tert*-Butoxycarbonyl)­amino)-4-(difluoromethyl)-6-vinylpyrimidine-5-carboxylate (**73**)

To a solution of Compound 72 (24 g, 53.12 mmol, 1 equiv), Pd­(dppf)­Cl_2_ (3.11 g, 4.25 mmol, 0.08 equiv), Cs_2_CO_3_ (34.61 g, 106.23 mmol, 2 equiv) and potassium trifluoro­(vinyl)­boranuide (14.23 g, 106.23 mmol, 2 equiv) in dioxane (240 mL) and water (24 mL), the mixture was stirred at 100 °C for 3 h. The reaction mixture was then diluted with water (50 mL) and extracted with EtOAc (3 × 100 mL). The combined organic layers were washed with brine (100 mL), dried over Na_2_SO_4_ filtered, and concentrated *in vacuo* to obtain the crude product. Purification by column chromatography (0–20% ethyl acetate/petroleum ether) afforded compound **73** (16 g, 36.08 mmol, 67.93% yield) as a colorless oil. ^1^H NMR: 400 MHz, CDCl_3_ δ = 7.00–6.53 (m, 3H), 5.88–5.78 (m, 1H), 4.53–4.41 (m, 2H), 1.48 (s, 15H), 1.47–1.42 (m, 5H)

##### Step 7: Synthesis of Ethyl 2-(bis­(*tert*-Butoxycarbonyl)­amino)-4-(difluoromethyl)-6-formylpyrimidine-5-carboxylate (**74**)

NaIO_4_ (17.36 g, 81.18 mmol, 3 equiv) was added to a solution of Compound 73 (12 g, 27.06 mmol, 1 equiv) in dioxane (150 mL) and H_2_O (50 mL) at 20 °C, then OsO_4_ (35 μL, 677 μmol, 0.025 equiv) was added, the mixture was stirred at 20 °C for 1 h. The mixture was added water (50 mL), then extracted with DCM (50 mL × 3), the combined organic layers were washed with brine (100 mL), dried over Na_2_SO_4_, filtered and concentrated *in vacuo*. The crude material was purified by flash chromatography (10–30% EtOAc/heptane) to afford Compound **74** (8.6 g, 50% yield, 70% purity) was obtained as a brown oil. ^1^H NMR 400 MHz, CDCl_3_ δ = 10.14–9.86 (m, 1H), 6.90–6.56 (m, 1H), 4.55–4.47 (m, 2H), 1.52 (s, 17H), 1.43–1.39 (m, 3H)

##### Step 8: Synthesis of *trans*-2-Amino-4-(difluoromethyl)-6-1-hydroxy-3-(trifluoromethyl)­cyclohexyl)­methyl)-6,7-dihydro-5*H*-pyrrolo­[3,4-*d*]­pyrimidin-5-one (**16**)

To a solution of compound **74** (345 mg, 0.77 mmol, 1 equiv.) in THF (2.5 mL) was added a suspension of compound 66 (362 mg, 1.55 mmol, 2 equiv.) and triethylamine (0.22 mL, 1.55 mmol, 2 equiv.) in DCM (2.5 mL). Sodium triacetoxyborohydride (492 mg, 2.32 mmol, 3 equiv.) and acetic acid (0.30 mL) were added and the reaction mixture was stirred at room temperature overnight. LCMS analysis showed remaining intermediate, therefore the reaction mixture was heated to 60 °C for 2 h. The solvent was evaporated in vacuo and the residue was dissolved in DCM (50 mL), washed with water and saturated aqueous NaHCO3, dried over MgSO4, filtered, and concentrated in vacuo. The crude material was purified by column chromatography (0–35% EtOAc/heptane) to afford tert-butyl N-tert-butoxycarbonyl-N-[4-(difluoromethyl)-6-[[1-hydroxy-3-(trifluoromethyl)­cyclohexyl]­methyl]-5-oxo-7H-pyrrolo­[3,4-d]­pyrimidin-2-yl]­carbamate (140 mg) as an oil. The material was dissolved in 4 M HCl in dioxane (1 mL) and stirred at room temperature for 2 h. The reaction mixture was concentrated in vacuo and the residue was dissolved in MeOH and treated with NH3 (7 N in MeOH, 1 mL) to afford the free base. After stirring for 10 min, the solvent was evaporated in vacuo and the residue was dissolved in DCM and washed with water. The organic layer was dried, filtered, and concentrated in vacuo to afford a crude material, which was purified by column chromatography (0–60% EtOAc/heptane) to afford compound **16** (36 mg, 11% yield over two steps) as a white solid.^1^H NMR (500 MHz, DMSO-*d*_6_) δ 7.73 (s, 2H), 7.28 (t, *J* = 53.8 Hz, 1H), 4.70 (s, 1H), 4.54 (s, 2H), 3.44–3.36 (m, 2H), 2.49–2.38 (m, 1H), 1.76 (dd, *J* = 37.6, 12.8 Hz, 2H), 1.66–1.48 (m, 3H), 1.29 (t, *J* = 12.8 Hz, 2H), 1.17 (ddd, *J* = 17.3, 12.3, 5.6 Hz, 1H). LC-MS: *m*/*z* 381 (M+H)^+^

#### Synthesis of 2-Amino-6-[[1-hydroxy-3-(trifluoromethyl)­cyclohexyl]­methyl]-4-methyl-7*H*-pyrrolo­[3,4-*d*]­pyrimidin-5-one (**17**)

##### Step 1: Synthesis of Ethyl 4-Methyl-2-methylsulfanyl-6-vinyl-pyrimidine-5-carboxylate (**76**)

To a stirred solution of ethyl 4-chloro-6-methyl-2-(methylthio)­pyrimidine-5-carboxylate (7.7 g, 31.21 mmol, 1.1 equiv) in ethanol (70 mL) in a flask with nitrogen bubbling through were added potassium vinyltrifluoroborate (5.017 g, 37.45 mmol, 1.2 equiv), Et_3_N (5.645 mL, 40.57 mmol, 1.3 equiv) and Pd­(dppf)­Cl_2_ (256 mg, 0.31 mmol, 0.01 equiv). The reaction flask was sealed under a nitrogen balloon and the reaction mixture was heated to 85 °C and stirred overnight. The reaction mixture was then concentrated *in vacuo* then EtOAc and water were added, and the two-phase system was shaken and separated. The aq. liquors were extracted with EtOAc and the organic extracts were combined, washed with brine, dried with MgSO_4_ and concentrated *in vacuo* to give the crude product as an orange-red thick oil. The crude product was dry-loaded and purified by column chromatography (0–30% EtOAc/heptane) to afford compound **76** (6.15 g, 79% yield) as a colorless oil. ^1^H NMR (400 MHz, CDCl_3_) δ 6.82 (dd, J = 16.8, 10.3 Hz, 1H), 6.70 (dd, J = 16.8, 2.1 Hz, 1H), 5.67 (dd, J = 10.3, 2.1 Hz, 1H), 4.40 (q, J = 7.1 Hz, 2H), 2.58 (s, J = 9.7 Hz, 3H), 2.49 (s, 3H), 1.39 (t, J = 7.1 Hz, 3H). LCMS: *m*/*z* 239 (M+H)^+^

##### Step 2: Synthesis of Ethyl 4-Formyl-6-methyl-2-methylsulfanyl-pyrimidine-5-carboxylate (**77**)

To a solution of ethyl 4-methyl-2-methylsulfanyl-6-vinyl-pyrimidine-5-carboxylate (1 g, 4.20 mmol, 1 equiv) in THF (20 mL) and water (5 mL) was added a solution of dipotassium dioxido­(dioxo)osmium dihydrate (54 mg, 0.15 mmol, 0.04 equiv) in water (2 mL). The reaction mixture was stirred at room temperature for 15 min, then a suspension of sodium (meta)­periodate (3590 mg, 16.79 mmol, 4 equiv) in water (5 mL) was added dropwise over 5 min, followed by further THF (5 mL), and the reaction was stirred at room temperature for 2 h. The reaction mixture was diluted with ethyl acetate and washed with water and brine. The organic layer was dried using a phase separator, to which corn oil was added. This mixture was concentrated *in vacuo and* purified by column chromatography (0–60% EtOAc/heptane) to afford compound **77** (445 mg, 44% yield) as a pale brown oil. ^1^H NMR (400 MHz, CDCl_3_) δ 9.92 (s, 1H), 4.43 (q, *J* = 7.2 Hz, 2H), 2.63 (s, 3H), 2.56 (s, 3H), 1.38 (t, *J* = 7.2 Hz, 3H). LC-MS: *m*/*z* 241 (M+H)^+^

##### Step 3: Synthesis of 6-[[1-Hydroxy-3-(trifluoromethyl)­cyclohexyl]­methyl]-4-methyl-2-methylsulfanyl-7*H*-pyrrolo­[3,4-*d*]­pyrimidin-5-one (**78**)

To a solution of ethyl 4-formyl-6-methyl-2-methylsulfanyl-pyrimidine-5-carboxylate (100 mg, 0.42 mmol, 1 equiv) in DCM (3 mL) was added 1-(aminomethyl)-3-(trifluoromethyl)­cyclohexanol (98 mg, 0.50 mmol, 1.2 equiv) and a spatula tip of magnesium sulfate. To this suspension, sodium triacetoxyborohydride (265 mg, 1.25 mmol, 3 equiv) was added and the reaction was stirred overnight at 40 °C. The reaction was diluted with DCM and water and passed through a phase separator. The organics were concentrated *in vacuo* and purified by column chromatography (isocratic, 50% EtOAc/heptane) to afford compound **78** (150 mg, 91% yield) as an off-white solid. ^1^H NMR (500 MHz, CDCl_3_): δ 4.48 (s, 2H), 3.65–3.54 (m, 2H), 3.27 (s, 1H), 2.78 (s, *J* = 8.7 Hz, 3H), 2.62 (s, 3H), 1.96 (d, *J* = 13.5 Hz, 1H), 1.88 (d, *J* = 10.6 Hz, 1H), 1.72 (t, *J* = 8.7 Hz, 3H), 1.40–1.21 (m, 4H). LC-MS: *m*/*z* 376 (M+H)^+^

##### Step 4: Synthesis of 2-Amino-6-[[1-hydroxy-3-(trifluoromethyl)­cyclohexyl]­methyl]-4-methyl-7*H*-pyrrolo­[3,4-*d*]­pyrimidin-5-one (**17**)

To a solution of 6-[[1-hydroxy-3-(trifluoromethyl)­cyclohexyl]­methyl]-4-methyl-2-methylsulfanyl-7*H*-pyrrolo­[3,4-*d*]­pyrimidin-5-one (150 mg, 0.40 mmol, 1 equiv) in MeCN (2 mL) was added a solution of oxone (319 mg, 0.52 mmol, 2 equiv) in water (1 mL). The mixture was allowed to stir at room temperature for 1 h. Water and DCM were added, and the mixture was passed through a phase separator. The organics were concentrated *in vacuo* and dissolved in 1,4-dioxane (2 mL) before ammonia in 1,4-dioxane (1.598 mL, 0.799 mmol, 1.3 equiv) was added and the mixture was allowed to stir overnight at room temperature. Water was added and the mixture was extracted with DCM. The organics were washed with brine, dried with MgSO_4_ and concentrated *in vacuo.* The crude material was purified by reverse phase chromatography (5–95% MeCN/water with 0.1% formic acid buffer) to afford compound **17** (10 mg, 7% yield) as a white solid. ^1^H NMR (400 MHz, DMSO-*d*_6_): 7.19 (s, 2H), 4.69 (s, 1H), 4.39 (s, 2H), 3.39 (m, 2H), 2.49 (s, 3H), 1.81 (d, 1H), 1.71 (d, 1H), 1.64–1.49 (m, 4H), 1.33–1.2 (m, 2H), 1.22–1.11 (m, 1H). LC-MS: *m*/*z* 345 (M+H)^+^

#### Synthesis of Synthesis of 2-Amino-6-[[1-hydroxy-3-(trifluoromethyl)­cyclohexyl]­methyl]-4-methoxy-7*H*-pyrrolo­[3,4-*d*]­pyrimidin-5-one (**18**)

##### Step 1: Synthesis of *tert*-Butyl *N*-*tert*-Butoxycarbonyl-*N*-(4-chloro-6-methoxy-pyrimidin-2-yl)­carbamate (**80**)

To a suspension of 4-chloro-6-methoxypyrimidin-2-amine (5 g, 31.33 mmol, 1 equiv) in THF (70 mL) was added di-*tert*-butyl dicarbonate (13.677 g, 62.67 mmol, 2 equiv) and DMAP (383 mg, 3.13 mmol, 0.1 equiv) at 0 °C. The reaction was concentrated *in vacuo* and was purified by column chromatography (0–50% EtOAc/heptane) to afford compound **80** (11.274 g, 94% yield) as a clear, colorless oil. ^1^H NMR (500 MHz, CDCl_3_) δ 6.62 (s, 1H), 3.97 (s, 3H), 1.49 (d, *J* = 15.6 Hz, 18H). LC-MS: *m*/*z* 160 (M-CO_2_^t^Bu-CO_2_^t^Bu+3H)^+^

##### Step 2: Synthesis of Ethyl 2-[bis­(*tert*-Butoxycarbonyl)­amino]-4-chloro-6-methoxy-pyrimidine-5-carboxylate (**81**)

To a solution of *tert*-butyl *N*-tert-butoxycarbonyl-*N*-(4-chloro-6-methoxy-pyrimidin-2-yl)­carbamate (10.6 g, 29.46 mmol, 1 equiv) in anhydrous THF (147 mL) at −50 °C was added (diisopropylamino)lithium (2 M in THF, 17.7 mL, 35.35 mmol, 1.2 equiv). After 2 h chloroformic acid ethyl ester (2.8 mL, 29.46 mmol, 1 equiv) was added and the reaction left to warm to room temperature and stirred overnight. The reaction mixture was poured into brine then extracted with ethyl acetate. The organic layers were dried with magnesium sulfate and concentrated *in vacuo*. The crude material was purified by column chromatography (0–50% EtOAc/heptane) to afford compound **81** (11 g, 86% yield) as a clear, yellow oil. ^1^H NMR (500 MHz, CDCl_3_) δ 7.29 (s, *J* = 7.2 Hz, 1H), 4.44 (q, *J* = 7.1 Hz, 2H), 4.04 (s, *J* = 6.4 Hz, 3H), 1.53 (s, 18H), 0.91 (t, *J* = 6.9 Hz, 3H). LC-MS: *m*/*z* 248 (M-CO_2_^t^Bu-^t^Bu+OEt+4H)^+^

##### Step 3: Synthesis of Ethyl 2-[bis­(*tert*-Butoxycarbonyl)­amino]-4-methoxy-6-vinyl-pyrimidine-5-carboxylate (**82**)

To a 10–20 mL microwave vial was added methyl 2-[bis­(tert-butoxycarbonyl)­amino]-4-chloro-6-ethoxy-pyrimidine-5-carboxylate (2 g, 4.79 mmol, 1 equiv), potassium trifluoro­(vinyl)boron (962 mg, 7.18 mmol, 1.5 equiv), Et_3_N (556 mg, 5.51 mmol, 1.15 equiv) and Pd­(dppf)­Cl_2_ (78 mg, 0.10 mmol, 0.02 equiv). The vial was capped and purged with nitrogen before ethanol (20 mL) was added. The reaction was heated in a microwave for 1.5 h at 120 °C. The reaction mixture was filtered through Celite and rinsed with ethanol. After concentration *in vacuo*, the crude material was purified by column chromatography (0–25% EtOAc/heptane) to afford compound **82** (380 mg, 26% yield) as a clear gum.

##### Step 4: Synthesis of Ethyl 2-[bis­(*tert*-butoxycarbonyl)­amino]-4-formyl-6-methoxy-pyrimidine-5-carboxylate (**83**)

To a solution of ethyl 2-[bis­(tert-butoxycarbonyl)­amino]-4-methoxy-6-vinyl-pyrimidine-5-carboxylate (244 mg, 0.58 mmol, 1 equiv) in 1,4-dioxane (3 mL) and Water (0.5 mL) was added osmium tetroxide (0.092 mL, 0.01 mmol, 0.025 equiv) and sodium periodate (370 mg, 1.73 mmol, 3 equiv). The reaction was stirred at room temperature overnight. The reaction mixture was diluted with water and extracted with ethyl acetate. To the organic layer was added 0.5 mL of corn oil and the solution became slightly darker. This was concentrated *in vacuo* to afford compound **83** (160 mg) as a brown oil. The crude material was used in subsequent reactions without further purification.

##### Step 4: Synthesis of 2-Amino-6-[[1-hydroxy-3-(trifluoromethyl)­cyclohexyl]­methyl]-4-methoxy-7*H*-pyrrolo­[3,4-*d*]­pyrimidin-5-one (**18**)

A suspension of ethyl 2-[bis­(*tert*-butoxycarbonyl)­amino]-4-formyl-6-methoxy-pyrimidine-5-carboxylate (96 mg, 0.23 mmol, 1 equiv), 1-(aminomethyl)-3-(trifluoromethyl)­cyclohexanol (66 mg, 0.34 mmol, 1.5 equiv) and a spatula of sodium sulfate in DCM (1 mL) was stirred for 5 min at room temperature. Sodium triacetoxyborohydride (62 mg, 0.25 mmol, 1.1 equiv) was added and the reaction was stirred at room temperature overnight. The reaction mixture was concentrated *in vacuo* and purified directly by column chromatography (0–80% EtOAc/heptane) to a white solid. This was dissolved in DCM (1 mL) and TFA (0.17 mL, 2.25 mmol, 10 equiv) was added and the mixture was stirred for 1 h at room temperature. The reaction mixture was concentrated *in vacuo* to afford compound **18** (55 mg, 44% yield) as a white solid. ^1^H NMR (400 MHz, DMSO-*d*_6_) δ 7.25 (s, 2H), 4.66 (s, 1H), 4.45–4.29 (m, 2H), 3.91 (s, 3H), 3.44–3.32 (m, 2H), 2.47–2.36 (m, 1H), 1.80 (d, *J* = 11.9 Hz, 1H), 1.74–1.46 (m, 4H), 1.37–1.07 (m, 3H). LC-MS: *m*/*z* 361 (M+H)^+^

#### Synthesis of 2-Amino-4-(difluoromethoxy)-6-1-hydroxy-3-(trifluoromethyl)­cyclohexyl)­methyl)-6,7-dihydro-5*H*-pyrrolo­[3,4-*d*]­pyrimidin-5-one (19)

##### Step 1: Synthesis of 6-Chloro-2-(methylthio)­pyrimidin-4­(3*H*)-one (**85**)

4,6-Dichloro-2-(methylthio)­pyrimidine (1) (15 g, 76.5 mmol) was taken in aqueous solution of NaOH (2 M, 375 mL) and stirred at 120 °C for 5 h. The reaction mixture was cooled to room temperature and acetic acid was added until pH 6 was reached. A white precipitate formed was filtered and washed with water. The solid product was collected, triturated with Et_2_O and concentrated *in vacuo* to afford compound **85** (10 g, 74%) as white solid. ^1^H NMR (400 MHz, DMSO-*d*_6_): δ 13.12 (br, 1H), 6.29 (s, 1H), 2.50 (s, 3H)

##### Step 2: Synthesis of 4-Chloro-6-(difluoromethoxy)-2-(methylthio)­pyrimidine (**86**)

To the stirred solution of 6-chloro-2-(methylthio)­pyrimidin-4­(3H)-one (9 g, 50.95 mmol, 1 equiv) in acetonitrile-DMF (500 mL, 5:1), ClCF_2_CO_2_Na (11.6 g, 76.43 mmol, 1.5 equiv) and Na_2_CO_3_ (10.8 g, 102 mmol, 2 equiv) were added. The mixture was heated at 90 °C for 16 h. The reaction mixture was diluted with water (200 mL), extracted with ethyl acetate (200 mL × 3), and the combined organic layers were washed with brine (50 mL), dried over anhydrous Na_2_SO_4_ filtered, and concentrated. The crude product was purified by column chromatography (10% ethyl acetate in hexane) to afford compound **86** (9.5 g, 82%). ^1^H NMR (400 MHz, CDCl_3_): δ 7.44 (t, 2JHF = 69.8 Hz, 1H), 6.56 (s, 1H), 2.53 (s, 3H)

##### Step 3: Synthesis of Ethyl 4-Chloro-6-(difluoromethoxy)-2-(methylthio)­pyrimidine-5-carboxylate (**87**)

To the stirred solution of 2,2,6,6-tetramethylpiperidine (12.84 mL, 75.45 mmol, 1 equiv) in THF (325 mL), *n*-BuLi (32 mL, 75.45 mmol, 2.5 M in THF, 2 equiv) was added dropwise at −78 °C, and stirring was continued for 1 h at the same temperature. Then, 4-chloro-6-(difluoromethoxy)-2-(methylthio)­pyrimidine (9.5 g, 41.92 mmol, 1 equiv) in THF (25 mL) was added, and stirring was continued at −78 °C for 1.5 h. Ethyl carbonochloridate (5.85 mL, 75.45 mmol, 2 equiv) was added, and the mixture was stirred at room temperature for 16 h. The reaction mixture was quenched with water (100 mL) and extracted with ethyl acetate (150 mL × 3). The combined organic layers were washed with brine (50 mL), dried over anhydrous Na_2_SO_4_, filtered, and concentrated. The crude product was purified by column chromatography (10% ethyl acetate in hexane) to afford compound **87** (2.5 g, 20%). 1H NMR (400 MHz, CDCl_3_) δ 7.42 (t, 2JHF = 70.8 Hz, 1H), 4.42 (q, J= 7.1 Hz, 2H), 2.55 (s, 3H), 1.38 (t, J = 7.1 Hz, 3H). LCMS: RT 1.87 min; *m*/*z* 299.0 [M+H]^+^

##### Step 4: Synthesis of Ethyl 4-(Difluoromethoxy)-2-(methylthio)-6-vinylpyrimidine-5-carboxylate (**88**)

To the stirred solution of ethyl 4-chloro-6-(difluoromethoxy)-2-(methylthio)­pyrimidine-5-carboxylate (2.5 g, 8.37 mmol, 1 equiv) in ethanol (80 mL), DIPEA (2.6 mL, 15.06 mmol, 1.8 equiv) was added and the mixture was degassed with nitrogen for 15 min. Potassium vinyl trifluoroborate (1.68 g, 12.55 mmol, 1.5 equiv) and Pd­(dppf)­Cl_2_·DCM (0.342 g, 0.41 mmol, 0.05 equiv) were then added. The reaction mixture was refluxed at 90 °C for 16 h under a nitrogen atmosphere. The reaction was concentrated, diluted with DCM (75 mL), washed with saturated NaHCO_3_ solution (15 mL), dried over anhydrous Na_2_SO_4_, filtered, and concentrated. The crude product was purified by column chromatography (10% ethyl acetate in hexane) to afford compound **88** (2.2 g, 91%). LC-MS: *m*/*z* 291.3 (M+H)^+^

##### Step 5: Synthesis of Ethyl 4-(Difluoromethoxy)-6-formyl-2-(methylthio)­pyrimidine-5-carboxylate (**89**)

To the stirred solution of ethyl 4-(difluoromethoxy)-2-(methylthio)-6-vinylpyrimidine-5-carboxylate (2.2 g, 7.58 mmol, 1 equiv) in THF–water (6:1, 50 mL), 2,6-lutidine (1.76 mL, 15.16 mmol, 2 equiv), 4% aqueous solution of OsO_4_ (2.5 mL, 0.38 mmol, 0.05 equiv) and NaIO_4_(6.5 g, 30.32 mmol, 4 equiv) were added and the mixture was stirred at room temperature for 16 h. The reaction mixture was concentrated, extracted with ethyl acetate (60 mL × 3), and washed with saturated Na_2_S_2_O_3_ solution (20 mL). The organic layer was dried over anhydrous Na_2_SO_4_, filtered and concentrated. The crude product was purified by column chromatography (10% ethyl acetate in hexane) to afford compound **89** (1.3 g, 59%). LC-MS: *m*/*z* 293.2 (M+H)^+^

##### Step 6: Synthesis of *trans*-4-(Difluoromethoxy)-6-1-hydroxy-3-(trifluoromethyl)­cyclohexyl)­methyl)-2-(methylthio)-6,7-dihydro-5*H*-pyrrolo­[3,4-*d*]­pyrimidin-5-one (**90**)

To a stirred solution of ethyl 4-(difluoromethoxy)-6-formyl-2-(methylthio)­pyrimidine-5-carboxylate (600 mg, 2.05 mmol, 1 equiv) in methanol (20 mL) were added ((1*S*,3*S*)-1-(aminomethyl)-3-(trifluoromethyl)­cyclohexan-1-ol (608 mg, 3.08 mmol, 1.5 equiv) and 1–2 drops of acetic acid. The mixture was stirred for 30 min at room temperature. Sodium cyanoborohydride (388 mg, 6.16 mmol, 3 equiv) was then added at 0 °C, and the reaction mixture was stirred at room temperature for 15 h. The mixture was concentrated, extracted with ethyl acetate (60 mL × 3), washed with saturated NaHCO_3_ solution (20 mL), dried over anhydrous Na_2_SO_4_, filtered, and concentrated. The crude product was purified by column chromatography (20% ethyl acetate in hexane) to afford compound **90** (340 mg, 38.75% yield). ^1^H NMR (400 MHz, CDCl_3_) δ 7.58 (t,^2^J_HF = 70.7 Hz, 1H), 4.53 (s, 2H), 3.61–3.53 (m, 2H), 2.58 (s, 3H), 2.58–2.46 (m, 1H), 1.96–1.17 (m, 8H). LC-MS: *m*/*z* 428.3 (M+H)^+^

##### Step 7: Synthesis of 4-(Difluoromethoxy)-6-(((1*r*,3*r*)-1-hydroxy-3-(trifluoromethyl)­cyclohexyl)­methyl)-2-(methylsulfonyl)-6,7-dihydro-5*H*-pyrrolo­[3,4-*d*]­pyrimidin-5-one (**91**)

To the stirred solution of *trans*-rac-4-(difluoromethoxy)-6-(((1*S*,3*S*)-1-hydroxy-3-(trifluoromethyl)­cyclohexyl)­methyl)-2-(methylsulfinyl)-6,7-dihydro-5H-pyrrolo­[3,4-*d*]­pyrimidin-5-one (9) (340 mg, 0.80 mmol, 1 equiv) in dichloromethane (30 mL) was added mCPBA (686 mg, 3.98 mmol, 5 equiv) at 0 °C, and the reaction was stirred at room temperature for 16 h. The reaction mixture was concentrated and extracted with ethyl acetate (30 mL × 2). The combined organic layer was washed with 10% Na_2_S_2_O_3_ solution (10 mL), saturated NaHCO_3_ solution (10 mL), dried over anhydrous Na_2_SO_4_, filtered, and concentrated to afford compound **91** (350 mg, 96% yield). This was taken to the next step without further purification. LC-MS: *m*/*z* 460.2 (M+H)^+^

##### Step 8: Synthesis of 2-Amino-4-(difluoromethoxy)-6-1-hydroxy-3-(trifluoromethyl)­cyclohexyl)­methyl)-6,7-dihydro-5*H*-pyrrolo­[3,4-*d*]­pyrimidin-5-one (**19**)

*trans*-rac-4-(Difluoromethoxy)-6-(((1*S*,3*S*)-1-hydroxy-3-(trifluoromethyl)­cyclohexyl)­methyl)-2-(methylsulfonyl)-6,7-dihydro-5H-pyrrolo­[3,4-*d*]­pyrimidin-5-one (10) (340 mg, 0.74 mmol, 1 equiv) was taken in THF (20 mL) in a sealed tube, and ammonia was passed through the reaction mixture for 15 min. The tube was sealed and stirred at room temperature for 16 h. The reaction mixture was concentrated, extracted with ethyl acetate (30 mL × 2), and washed with saturated NaHCO_3_ solution (10 mL). The organic layer was dried over anhydrous Na_2_SO_4_, filtered, and concentrated. The crude product was purified by preparative HPLC to afford compound **19**.

^1^H NMR (400 MHz, CDCl_3_) δ 7.49 (t,^2^J_HF = 70.8 Hz, 1H), 5.42 (s, 2H), 4.38 (s, 2H), 3.57–3.45 (m, 2H), 2.55–2.45 (m, 1H), 1.95–1.18 (m, 8H). LC-MS: *m*/*z* 397.4 (M+H)^+^

#### Synthesis of 2-Amino-6-[[1-amino-3-(trifluoromethyl)­cyclohexyl]­methyl]-4-(trifluoromethyl)-7*H*-pyrrolo­[3,4-*d*]­pyrimidin-5-one (**20**)

##### Step 1: Synthesis of Benzyl *N*-[1-Cyano-3-(trifluoromethyl)­cyclohexyl]­carbamate (93)

A mixture of ammonium hydroxide (15 mL, 385.16 mmol, 105 equiv), ammonium chloride (390 mg, 7.30 mmol, 2 equiv) and cyanopotassium (475 mg, 7.30 mmol, 2 equiv) was prepared at room temperature, and 3-(trifluoromethyl)­cyclohexan-1-one (606 mg, 3.65 mmol, 1 equiv) in methanol (5 mL) was added. The mixture and stirred at room temperature overnight. DCM was added and the extracted organics were dried with sodium sulfate and concentrated *in vacuo*. The crude material was diluted in DCM (20 mL) and potassium carbonate (655 mg, 4.74 mmol, 1.3 equiv) was added, followed by the dropwise addition of benzyl chlorofomate (0.67 mL, 4.74 mmol, 1.3 equiv) and the reaction was stirred at room temperature overnight. DCM was added and the mixture was washed with sat. aq. NaHCO_3_. The organics were dried through a phase separator and concentrated *in vacuo*, before being purified by column chromatography (0–50% EtOAc/heptane) to afford compound 93 (820 mg, 65% yield) as a white solid. ^1^H NMR (500 MHz, DMSO-*d*_6_) δ 7.42–7.31 (m, 5H), 5.13–5.08 (m, 2H), 2.69–2.61 (m, 1H), 2.46–2.37 (m, 1H), 2.35–2.27 (m, 1H), 1.96–1.86 (m, 2H), 1.83–1.73 (m, 1H), 1.58–1.51 (m, 2H), 1.37–1.23 (m, 1H).

##### Step 2: Synthesis of *N*-[1-(Aminomethyl)-3-(trifluoromethyl)­cyclohexyl]­carbamate (**94**)

To a solution of benzyl *N*-[1-cyano-3-(trifluoromethyl)­cyclohexyl]­carbamate (400 mg, 1.23 mmol, 1 equiv) in THF (3 mL) was added borane-DMS (2 M in THF, 1.8 mL, 3.6 mmol, 3 equiv) dropwise at room temperature. The reaction was stirred in a sealed vessel at room temperature for 5 h. The reaction was quenched with water and extracted with ethyl acetate, before being dried with magnesium sulfate and concentrated *in vacuo*. The resulting crude was applied to an SCX column and was washed with methanol, followed by ammonia in methanol (3.5 M) to afford compound 94 (232 mg, 57% yield) as a colorless oil. ^1^H NMR (500 MHz, DMSO-*d*_6_) δ 7.51–7.15 (m, 5H), 6.86 (s, 1H), 5.03–4.89 (m, 2H), 2.82–2.72 (m, 2H), 2.43–2.29 (m, 1H), 2.15–2.00 (m, 1H), 1.80 (dd, *J* = 20.7, 12.9 Hz, 2H), 1.73–1.55 (m, 3H), 1.46–1.29 (m, 1H), 1.20–1.07 (m, 1H).

##### Step 3: Synthesis of 2-Amino-6-[[1-amino-3-(trifluoromethyl)­cyclohexyl]­methyl]-4-(trifluoromethyl)-7*h*-pyrrolo­[3,4-*d*]­pyrimidin-5-one (**20**)

To a solution of benzyl *N*-[1-(aminomethyl)-3-(trifluoromethyl)­cyclohexyl]­carbamate (333 mg, 1.01 mmol, 2 equiv) and diisopropylethylamine (0.27 mL, 1.55 mmol, 3 equiv) in MeCN (8 mL) was added ethyl 2-amino-4-(bromomethyl)-6-(trifluoromethyl)­pyrimidine-5-carboxylate (165 mg, 0.50 mmol, 1 equiv) portionwise, and the mixture was stirred at room temperature for 3.5 h, then stirred at 80 °C overnight. The reaction mixture was concentrated *in vacuo* then applied to an SCX cartridge and rinsed with methanol. The resulting solution was concentrated *in vacuo* and purified by reverse phase chromatography (5–95% water/MeCN, with 0.1% formic acid buffer). The resulting material was dissolved in methanol (12 mL) and added to a flask containing Pd/C (10%, 22 mg) under nitrogen. The suspension was stirred under an atmosphere of hydrogen (balloon) overnight at room temperature. The mixture was filtered through a pad of Celite and washed with methanol, before being concentrated *in vacuo* and purified by column chromatography (0–10% MeOH/DCM) to afford compound **20** (28 mg, 13% yield) as a white solid. ^1^H NMR (400 MHz, CDCl_3_) δ 5.63 (s, 2H), 4.71 (d, *J* = 19.2 Hz, 1H), 4.59 (d, *J* = 19.2 Hz, 1H), 3.65 (d, *J* = 14.3 Hz, 1H), 3.42 (d, *J* = 14.3 Hz, 1H), 2.65–2.45 (m, 1H), 1.97 (d, *J* = 13.2 Hz, 1H), 1.89–1.60 (m, 3H), 1.29–1.08 (m, 3H). LC-MS: *m*/*z* 398 (M+H)^+^

#### Synthesis of Common Intermediate (Compound 100) for the Preparation of Compounds 23–36

##### Step 1: Synthesis of Ethyl 2-(3-(Trifluoromethyl)­cyclohexylidene)­acetate (**96a**)

In a round-bottom flask, LiCl (10.7 g, 252.8 mmol, 1.4 equiv) was suspended in MeCN (230 mL) and cooled in an ice bath. To this ice cooled solution, DBU (32.4 mL, 216.7 mmol, 1.2 equiv) was added dropwise followed by ethyl 2-(diethoxyphosphoryl)­acetate (46.8 mL, 234.7 mmol, 1.3 equiv). The reaction mixture was stirred at 0 °C for 30 min. After 30 min, 3-(trifluoromethyl)­cyclohexan-1-one (30.0 g, 180.6 mmol, 1 equiv) was added dropwise over a period of 30 min. The reaction mixture was stirred for another 30 min at 0 °C, then at room temperature overnight. The reaction mixture was cooled to 0 °C and quenched with saturated NaHCO_3_ solution. The crude reaction mixture was extracted with EtOAc and purified by column chromatography (0–5% EtOAc/heptane) to afford compound **96a** (mixture of E & Z isomer, 41.0 g, 96% yield) as colorless oil. ^1^H NMR (400 MHz, CDCl_3_) δ 5.70 (s, 1H), 4.17–3.80 (m, 3H), 2.46–2.27 (m, 1H), 2.19–2.12 (m, 2H), 2.08–1.95 (m, 2H), 1.91–1.81 (m, 1H), 1.55–1.45 (m, 2H), 1.42–1.39 (m, 3H) ppm. LC-MS: *m*/*z* = 237 [M+H]^+^

##### Step 2: Synthesis of *cis*-2-((1*r*,3*s*) or (1*s*,3*r*)-1-(Nitromethyl)-3-(trifluoromethyl)­cyclohexyl)­acetate (**97a**)

Ethyl 2-(3-(trifluoromethyl)­cyclohexylidene)­acetate (E & Z mixture 54.0 g, 228.7 mmol, 1 equiv) was dissolved in THF (200 mL) and to this stirring solution nitromethane (24.5 mL, 457.4 mmol, 2 equiv) followed by 1.0 M TBAF in THF (228.7 mL, 228.7 mmol, 1 equiv) was added. The reaction mixture was heated at 70 °C for 16 h. The reaction mixture was diluted with EtOAc and washed with 2 M HCl. The crude reaction mixture was extracted in EtOAc and purified by column chromatography (0–5% EtOAc/heptane) to afford compound **97a** (59.2 g, 87% yield) as yellow oil. ^1^H NMR (400 MHz, CDCl_3_) δ 4.62–4.57 (m, 2H), 4.19–4.10 (m, 2H), 2.62–2.48 (m, 2H), 2.29–2.20 (m, 1H), 1.99 (d, *J* = 12.7 Hz, 2H), 1.80 (d, *J* = 9.6 Hz, 2H), 1.56–1.45 (m, 2H), 1.34–1.28 (m, 5H) ppm. LC-MS: *m*/*z* = 298 [M+H]^+^

##### Step 3: Synthesis of *cis*-2-((1*r*,3*s*) or (1*s*,3*r*)-1-(Nitromethyl)-3-(trifluoromethyl)­cyclohexyl)­ethan-1-ol (**98a**)

*Cis*-2-((1*R*,3*S*) or (1*S*,3*R*)-1-(nitromethyl)-3-(trifluoromethyl)­cyclohexyl)­acetate (3.8 g, 12.8 mmol, 1 equiv) was dissolved in toluene (50 mL) and cooled to −78 °C. DIBAL solution (1.5 M in toluene, 13.0 mL, 1.5 equiv) was added dropwise over 35 min, producing an exotherm. The reaction mixture stirred 1 h before removing the cooling bath. Once the reaction mixture reached to room temperature, the solution was colorless. The reaction mixture was cooled to 0 °C and quenched by dropwise addition of saturated NH_4_Cl solution. Two M HCl was added dropwise and crude reaction mixture was extracted with EtOAc, dried with sodium sulfate, and purified by column chromatography (0–20% EtOAc/heptane) to afford compound **98a** (2.2 g, 67% yield) as pale oil. ^1^H NMR (400 MHz, CDCl_3_) δ 4.45 (s, 2H), 3.81 (d, *J* = 2.9 Hz, 2H), 2.27–2.21 (m, 1H), 1.95–1.92 (m, 2H), 1.84–1.75 (m, 4H), 1.52–1.40 (m, 2H), 1.33–1.14 (m, 3H) ppm.

##### Step 4: Synthesis of *cis*-2-((1*r*,3*s*) or (1*s*,3*r*)-1-(Aminomethyl)-3-(trifluoromethyl)­cyclohexyl)­ethan-1-ol (**99a**)

*Cis*-2-((1*R*,3*S*) or (1*S*,3*R*)-1-(nitromethyl)-3-(trifluoromethyl)­cyclohexyl)­ethan-1-ol (6.5 g, 25.5 mmol, 1 equiv) was dissolved in EtOH (157 mL) and Fe powder (14.2 g, 255.0 mmol, 10 equiv) followed by NH_4_Cl (13.6 g, 255.0 mmol, 10 equiv) in water (51 mL) were added. The reaction mixture was stirred at 80 °C for 4 h. The reaction mixture was filtered through a pad of Celite and washed with EtOH. Combined volatiles were removed *in vacuo*, and the crude product was dissolved in DCM and passed through a pad of Celite and washed with DCM. The organics were concentrated *in vacuo* to get the crude amine as sticky solid, which was further crystallized from hot CH_3_CN to afford compound **99a** (5.1 g, 89% yield) as white solid. ^1^H NMR (400 MHz, CDCl_3_) δ 8.14 (s, 2H), 3.73 (d, *J* = 4.2 Hz, 2H), 3.35 (s, 2H), 2.95–2.75 (m, 2H), 2.11 (brs, 1H), 1.95 (d, *J* = 12.5 Hz, 2H), 1.83–1.65 (m, 3H), 1.42–1.32 (m, 1H), 1.25–1.15 (m, 3H) ppm. LCMS: *m*/*z* = 226 [M+H]^+^

##### Step 5: Synthesis of *cis*-2-Amino-6-(((1*r*,3*s*) or (1*s*,3*r*)-1-(2-Hydroxyethyl)-3-(trifluoromethyl)­cyclohexyl)­methyl)-4-(trifluoromethyl)-6,7-dihydro-5h-pyrrolo­[3,4-*d*]­pyrimidin-5-one (**21**)

To a stirred solution ethyl 2-amino-4-(bromomethyl)-6-(trifluoromethyl) pyrimidine-5-carboxylate (22 g, 67 mmol, 1 equiv) in ethanol (100 mL), was added *cis*-2-(1-(aminomethyl)-3-(trifluoromethyl) cyclohexyl) ethan-1-ol (22.6 g, 100.5 mmol, 1.5 equiv) followed by DIPEA (34.8 mL, 201.1 mmol, 3 equiv). The reaction mixture was stirred at room temperature for 48 h. The reaction mixture was concentrated *in vacuo* and purified by column chromatography (0–50% EtOAc/heptane) to afford compound **21** (19.1 g, 67% yield) as an off-white solid. The enantiomers were separated by chiral prep-HPLC to afford **21a** (9 g) as first eluting enantiomer.

^1^H NMR (400 MHz, CDCl_3_) δ 5.68 (s, 2H), 4.42 (s, 2H), 3.83–3.79 (m, 2H), 3.57–3.47­(m, 2H), 3.35 (t, *J* = 5.4 Hz, 1H), 2.21–2.15 (m, 1H), 1.95–1.87 (m, 2H), 1.76–1.71 (m, 2H), 1.68–1.59 (m, 2H), 1.48–1.38 (m, 1H), 1.23–1.11 (m, 3H) ppm. LCMS RT 4.99 min; *m*/*z* = 427.26 [M+H]^+^.

##### Step 5: Synthesis of *cis*-2-((1*r*,3*s*) or (1*s*,3*r*)-1-((2-Amino-5-oxo-4-(trifluoromethyl)-5,7-dihydro-6h-pyrrolo­[3,4-*d*]­pyrimidin-6-yl)­methyl)-3-(trifluoromethyl)­cyclohexyl)­acetaldehyde (100)

To a stirred solution of **Compound 21a** (100 mg, 0.24 mmol, 1 equiv) in DCM–CHCl_3_ (1:1, 10 mL) was added DMP (795.6 mg, 1.88 mmol, 7.83 equiv) at 0 °C, and the reaction mixture was stirred at the same temperature for 30 min. After completion, the reaction was quenched with saturated NaHCO_3_ solution and extracted with ethyl acetate (50 mL × 2). The combined organic layers were dried over anhydrous Na_2_SO_4_, filtered, and concentrated *in vacuo* to afford compound **100** (110 mg, crude) as a white solid. This was taken to the next step without further purification. LCMS: *m*/*z* = 425 [M+H]^+^.

#### Synthesis of *cis*-Racemic 2-Amino-6-((1-(2-aminoethyl)-3-(trifluoromethyl)­cyclohexyl)­methyl)-4-(trifluoromethyl)-6,7-dihydro-5h-pyrrolo­[3,4-*d*]­pyrimidin-5-one (**22**)

##### Step 1: Synthesis of 2-(3-(Trifluoromethyl)­cyclohexylidene)­acetonitrile (**96b**)

To a stirred solution of NaH (451 mg, 18.78 mmol, 1.3 equiv) in THF (55 mL), diethyl (cyanomethyl)­phosphonate (3.07 g, 17.33 mmol, 1.2 equiv) was added at 0 °C and stirred for 15 min under a nitrogen atmosphere. Then, 3-(trifluoromethyl)­cyclohexan-1-one (2.40 g, 14.45 mmol, 1.0 equiv) in THF (55 mL) was added, and the reaction was stirred overnight at room temperature. The liquor was decanted, the residue washed with ether, and the combined organic layers were washed with 1.5 N HCl and water, dried over anhydrous Na_2_SO_4_, and concentrated *in vacuo*. The crude product was purified by column chromatography (20% ethyl acetate in hexane) to afford compound **96b** (1.30 g, 47%). ^1^H NMR (400 MHz, CDCl_3_) δ 5.19–5.17 (m, 1H), 3.13–1.92 (m, 5H), 1.49–1.37 (m, 4H) ppm.

#### Step 2: Synthesis of cis 2-(1-(Nitromethyl)-3-(trifluoromethyl)­cyclohexyl)­acetonitrile (97b)

To a stirred solution of 2-(3-(trifluoromethyl)­cyclohexylidene)­acetonitrile (1.30 g, 6.87 mmol, 1.0 equiv) in THF (22 mL), nitromethane (0.74 mL, 13.74 mmol, 2.0 equiv) and TBAF (10.7 mL, 1 M in THF, 10.7 mmol, 1.6 equiv) were added, and the reaction mixture was refluxed at 70 °C for 16 h. The mixture was extracted with ethyl acetate and washed with dilute HCl followed by water. The organic layer was dried over anhydrous Na_2_SO_4_ and concentrated. The crude product was purified by column chromatography (15–20% ethyl acetate in hexane) to afford compound 97b (1.00 g, 58%). ^1^H NMR (400 MHz, CDCl_3_) δ 4.45 (dd, J = 11.6, 7 Hz, 2H), 2.71 (s, 2H), 2.18–2.11 (m, 1H), 2.08–2.01 (m, 1H), 1.95–1.85 (m, 2H), 1.83–1.75 (m, 1H), 1.48–1.35 (m, 3H), 1.33–1.20 (m, 1H) ppm.

#### Step 3: Synthesis of cis-2-(1-(Nitromethyl)-3-(trifluoromethyl)­cyclohexyl)­ethan-1-amine (98b**)**

To a stirred solution of 2-(1-(nitromethyl)-3-(trifluoromethyl)­cyclohexyl)­acetonitrile (1.00 g, 3.99 mmol, 1.0 equiv) in toluene (20 mL), borane–dimethyl sulfide complex (BH_3_·DMS) (0.45 mL, 4.80 mmol, 1.2 equiv) was added at 0 °C and the mixture was stirred at room temperature for 16 h. Methanol was then added dropwise under cooling, followed by 4 M dioxane–HCl, and the mixture was refluxed for 1 h. The reaction mixture was concentrated, neutralized with aqueous NaOH solution, and extracted with ethyl acetate. The combined organic layers were washed with water and brine, dried over anhydrous Na_2_SO_4_, and concentrated to afford compound **98b** (1.20 g), which was used in the next step without further purification. LC-MS: *m*/*z* 255 (M+H)^+^

#### Step 4: Synthesis of cis-tert-Butyl (2-(1-(Nitromethyl)-3-(trifluoromethyl)­cyclohexyl)­ethyl)­carbamate ()

To a stirred solution of 2-(1-(nitromethyl)-3-(trifluoromethyl)­cyclohexyl)­ethan-1-amine (1.10 g, 4.23 mmol, 1.0 equiv) in DCM (50 mL), triethylamine (TEA) (0.90 mL, 6.49 mmol, 1.5 equiv) followed by di*tert*-butyl dicarbonate (Boc_2_O) (1.10 mL, 4.76 mmol, 1.1 equiv) were added and the mixture was stirred at room temperature for 16 h under a nitrogen atmosphere. The reaction was diluted with DCM, washed with water, dried over anhydrous Na_2_SO_4_, and concentrated. The crude product was purified by column chromatography (5–10% ethyl acetate in hexane) to afford (480 mg, 31%). ^1^H NMR (400 MHz, DMSO-*d*_6_) δ 6.79 (s, 1H), 4.51 (s, 2H), 3.43–3.38 (m, 1H), 3.00–2.95 (m, 2H), 2.50–2.47 (m, 1H), 1.63–1.11 (m, 19H) ppm. LC-MS: *m*/*z* 355 (M+H)^+^

#### Step 5: Synthesis of cis-tert-Butyl (2-(1-(Aminomethyl)-3-(trifluoromethyl)­cyclohexyl)­ethyl)­carbamate (99b)

cis-*tert*-Butyl (2-(1-(nitromethyl)-3-(trifluoromethyl)­cyclohexyl)­ethyl)­carbamate (480 mg, 1.35 mmol, 1.0 equiv) was dissolved in MeOH (10 mL) in a Parr shaker vessel, and Pd/C (250 mg, 5%) was added. The mixture was hydrogenated under 50 psi of hydrogen for 2 h. The reaction mixture was filtered through a Celite bed, washed with methanol, and concentrated. The crude product was purified by column chromatography (2–3% MeOH in dichloromethane) to afford compound **99b** (160 mg, 36%). ^1^H NMR (400 MHz, DMSO-*d*_6_) δ 6.72 (s, 1H), 2.86–2.81 (m, 2H), 2.38–2.30 (m, 3H), 1.82–1.79 (m, 1H), 1.59–1.56 (m, 2H), 1.49–1.40 (m, 4H), 1.36 (s, 9H), 1.12–0.94 (m, 3H) ppm. LC-MS: *m*/*z* 325 (M+H)^+^

#### Step 6: Synthesis of cis-Ethyl 2-Amino-4-((((1-(2-((tert-Butoxycarbonyl)­amino)­ethyl)-3-(trifluoromethyl)­cyclohexyl)­methyl)­amino)­methyl)-6-(trifluoromethyl)­pyrimidine-5-carboxylate

To a solution of cis-ethyl 2-amino-4-(bromomethyl)-6-(trifluoromethyl)­pyrimidine-5-carboxylate (150 mg, 0.46 mmol, 1.0 equiv) in acetonitrile (6 mL) were added triethylamine (0.10 mL, 0.68 mmol, 1.5 equiv) followed by 2-(3-(trifluoromethyl)­cyclohexylidene)­acetonitrile (148 mg, 0.46 mmol, 1.0 equiv). The reaction mixture was stirred for 6 h, then diluted with ethyl acetate, washed with water, dried over anhydrous Na_2_SO_4_, and concentrated under reduced pressure to afford 210 mg, which was used in the next step without further purification. This material was dissolved in toluene and heated at reflux for 6 h. The reaction mixture was concentrated and purified by flash chromatography (30–35% ethyl acetate in hexane) to afford (70 mg, 42%). ^1^H NMR (400 MHz, DMSO-*d*_6_) δ 8.10–7.92 (m, 2H), 6.75 (s, 1H), 4.50 (s, 2H), 3.31–3.29 (m, 2H), 3.00–2.99 (m, 2H), 1.81–1.78 (m, 1H), 1.65–1.59 (m, 2H), 1.53–1.42 (m, 4H), 1.37 (s, 9H), 1.23–1.10 (m, 4H) ppm. LC-MS: *m*/*z* 526 (M+H)^+^

#### Step 7: Synthesis of cis-Racemic 2-Amino-6-((1-(2-aminoethyl)-3-(trifluoromethyl)­cyclohexyl)­methyl)-4-(trifluoromethyl)-6,7-dihydro-5H-pyrrolo­[3,4-d]­pyrimidin-5-one (**22**)

To a stirred solution of cis-*tert*-butyl (2-(1-((2-amino-5-oxo-4-(trifluoromethyl)-5,7-dihydro-6H-pyrrolo­[3,4-*d*]­pyrimidin-6-yl)­methyl)-3-(trifluoromethyl)­cyclohexyl)­ethyl)­carbamate (10) (70 mg, 0.13 mmol, 1.0 equiv) in ether, HCl in ether (5 mL, 2M) was added at 0 °C and the mixture stirred at room temperature for 16 h. The reaction mixture was concentrated, titrated with pentane, and the solid was filtered and dried to afford compound **22** as the HCl salt (63 mg, 94%) as a yellow solid. ^1^H NMR (400 MHz, DMSO-*d*_6_) δ 8.03–7.81 (m, 5H), 4.51 (s, 2H), 3.29 (dd, J = 19.6, 14.2 Hz, 2H), 2.93–2.79 (m, 2H), 2.40–2.32 (m, 1H), 1.84–1.38 (m, 6H), 1.28–1.11 (m, 4H) ppm. LC-MS: *m*/*z* 426 (M+H)^+^

#### Synthesis of 2-Amino-6-[[1-[2-(1-piperidyl)­ethyl]-3-(trifluoromethyl)­cyclohexyl]­methyl]-4-(trifluoromethyl)-7H-pyrrolo­[3,4-*d*]­pyrimidin-5-one;formic acid (**23**)

Piperidine (21 mg, 0.25 mmol, 1.1 equiv) was dissolved in chloroform (4 mL) and compound 100 (95 mg, 0.22 mmol, 1 equiv) was added. The reaction was stirred for 5 min at room temperature before sodium triacetoxyborohydride (71.173 mg, 0.34 mmol, 1.5 equiv) was added at 0 °C. The reaction was stirred at room temperature for 2 h before being concentrated *in vacuo* and purified by column chromatography (0–10% MeOH/DCM) to afford compound **23** (60 mg, 47% yield) as a white solid. ^1^H NMR (500 MHz, DMSO-*d*_6_) δ 8.24 (s, 1H), 7.96 (s, 2H), 4.49 (s, 2H), 3.33 (dd, *J* = 24.0, 14.1 Hz, 2H), 2.49–2.29 (m, 6H), 1.81 (d, *J* = 10.8 Hz, 1H), 1.69 (d, *J* = 12.7 Hz, 1H), 1.66–1.43 (m, 10H), 1.43–1.34 (m, 2H), 1.27–1.05 (m, 4H). LCMS: *m*/*z* = 494 [M+H]^+^

#### Synthesis of 2-Amino-6-(((1*r*,3*s*)-1-(2-(piperazin-1-yl)­ethyl)-3-(trifluoromethyl)­cyclohexyl)­methyl)-4-(trifluoromethyl)-6,7-dihydro-5H-pyrrolo­[3,4-*d*]­pyrimidin-5-one (**24**)

1-(tert-butoxycarbonyl)-piperazine (88 mg, 0.47 mmol, 1.1 equiv) was dissolved in chloroform (4 mL) and 2-[1-[[2-amino-5-oxo-4-(trifluoromethyl)-7H-pyrrolo­[3,4-*d*]­pyrimidin-6-yl]­methyl]-3-(trifluoromethyl)­cyclohexyl]­acetaldehyde (182 mg, 0.43 mmol, 1 equiv) was added. The reaction was stirred for 5 min at room temperature before sodium triacetoxyborohydride (136 mg, 0.64 mmol, 1.5 equiv) was added at 0 °C. The reaction was stirred at room temperature for 2 h before being concentrated *in vacuo* and purified by column chromatography (0–10% MeOH/DCM) to afford the crude material (145 mg, 56% yield) as a white solid. This was dissolved in DCM (6 mL) HCl (4 M in dioxane, 2.3 mL, 9.25 mmol, 20 equiv) was added and the reaction was stirred for 48 h at room temperature. The reaction mixture was concentrated *in vacuo* and purified by reverse phase chromatography (5–95% MeCN/water with 0.1% formic acid buffer) to afford **24** (120 mg, 46% yield) as a white solid. ^1^H NMR (500 MHz, DMSO-*d*_6_) δ 11.88 (s, 1H), 9.68 (s, 2H), 7.98 (s, 2H), 4.52 (q, *J* = 18.4 Hz, 2H), 3.85–3.64 (m, 2H), 3.64–3.44 (m, 3H), 3.38–3.17 (m, 8H), 1.98–1.76 (m, 3H), 1.76–1.60 (m, 2H), 1.60–1.44 (m, 2H), 1.38–1.09 (m, 3H). LC-MS: *m*/*z* 495 (M+H)^+^

#### Synthesis of *cis*-2-Amino-6-(((1*r*,3*s*) or (1*s*,3*r*)-1-(2-(4-Methylpiperazin-1-yl)­ethyl)-3-(trifluoromethyl)­cyclohexyl)­methyl)-4-(trifluoromethyl)-6,7-dihydro-5H-pyrrolo­[3,4-*d*]­pyrimidin-5-one (**25**)

To a stirred solution of *cis*-2-((1*R*,3*S*) or (1*S*,3*R*)-1-((2-amino-5-oxo-4-(trifluoromethyl)-5,7-dihydro-6*H*-pyrrolo­[3,4-*d*]­pyrimidin-6-yl)­methyl)-3-(trifluoromethyl)­cyclohexyl)­acetaldehyde (390 mg, 0.92 mmol, 1 equiv) and 1-methylpiperazine (110 mg, 1.10 mmol, 1.2 equiv) in CHCl_3_ (7 mL), NaBH­(OAc)_3_ (584 mg, 2.76 mmol, 3 equiv) was added and the reaction mixture was stirred at room temperature for 2 h. After completion, the reaction mixture was extracted with 10% methanol in dichloromethane, washed with aqueous saturated NaHCO_3_ solution, brine, dried over anhydrous Na_2_SO_4_ and concentrated *in vacuo*. The crude product was purified by reverse-phase preparative-HPLC to afford compound **25** (85 mg, 17% yield) as an off-white solid formic acid salt (1:1). ^1^H NMR (400 MHz, DMSO-*d*_6_) δ 8.20 (s, 1 H), 7.99–7.91 (m, 2 H), 4.47 (s, 2 H), 3.34 (d, *J* = 14.2 Hz, 1 H), 3.28 (d, *J* = 14.2 Hz, 1 H), 2.50–2.20 (m, 10 H), 2.16 (s, 3 H), 1.79 (d, *J* = 11.3 Hz, 1 H), 1.67 (d, *J* = 12.4 Hz, 1 H), 1.61–1.58 (m, 1 H), 1.51–1.40 (m, 4 H), 1.23–1.10 (m, 4 H) ppm. LCMS: *m*/*z* = 509 [M+H]^+^

#### Synthesis of *cis*-2-Amino-6-(((1*r*,3*s*) or (1*s*,3*r*)-1-(2-(4-Cyclopropylpiperazin-1-yl)­ethyl)-3-(trifluoromethyl)­cyclohexyl)­methyl)-4-(trifluoromethyl)-6,7-dihydro-5H-pyrrolo­[3,4-*d*]­pyrimidin-5-one (**26**)

To a stirred solution of *cis*-2-((1*R*,3*S*) or (1*S*,3*R*)-1-((2-amino-5-oxo-4-(trifluoromethyl)-5,7-dihydro-6*H*-pyrrolo­[3,4-*d*]­pyrimidin-6-yl)­methyl)-3-(trifluoromethyl)­cyclohexyl)­acetaldehyde (100 mg, 0.24 mmol, 1 equiv) and 1-cyclopropylpiperazine (36 mg, 0.28 mmol, 1.2 equiv) in CHCl_3_ (3 mL), NaBH­(OAc)_3_ (75 mg, 0.35 mmol, 1.5 equiv) was added and the reaction mixture was stirred at room temperature for 2 h. After completion, the reaction mixture was concentrated *in vacuo* and purified by reverse phase preparative-HPLC to afford compound **26** (50 mg, 40% yield) as an off-white solid. ^1^H NMR (400 MHz, DMSO-*d*_6_) δ 7.99–7.91 (m, 2 H), 4.47 (s, 2 H), 3.35–3.26 (m, 2 H), 2.50–2.20 (m, 10 H), 1.79 (d, *J* = 11.7 Hz, 1 H), 1.67 (d, *J* = 12.7 Hz, 1 H), 1.61–1.45 (m, 6 H), 1.23–1.09 (m, 4 H), 0.40–0.36 (m, 2 H), 0.27–0.25 (m, 2 H) ppm. LC MS: *m*/*z* = 535 [M+H]^+^

#### Synthesis of_ *cis*-2-Amino-6-(((1*r*,3*s*) or (1*s*,3*r*)-1-(2-(4-(Oxetan-3-yl)­piperazin-1-yl)­ethyl)-3-(trifluoromethyl)­cyclohexyl)­methyl)-4-(trifluoromethyl)-6,7-dihydro-5H-pyrrolo­[3,4-*d*]­pyrimidin-5-one (**27**)

To a stirred solution of *cis*-2-((1*R*,3*S*) or (1*S*,3*R*)-1-((2-amino-5-oxo-4-(trifluoromethyl)-5,7-dihydro-6*H*-pyrrolo­[3,4-*d*]­pyrimidin-6-yl)­methyl)-3-(trifluoromethyl)­cyclohexyl)­acetaldehyde (200 mg, 0.47 mmol, 1 equiv) and 1-(oxetan-3-yl)­piperazine (80 mg, 0.57 mmol, 1.2 equiv) in CHCl_3_ (5 mL), NaBH­(OAc)_3_ (300 mg, 1.41 mmol, 3 equiv) was added and the reaction mixture was stirred at room temperature for 2 h. After completion, the reaction mixture was concentrated *in vacuo* and purified by reverse-phase preparative-HPLC to afford compound **27** (40 mg, 15% yield) as an off-white solid. ^1^H NMR (400 MHz, DMSO-*d*_6_) δ 7.99–7.91 (m, 2 H), 4.51 (t, *J* = 6.4 Hz, 2 H), 4.48 (s, 2 H), 4.40 (t, *J* = 6.0 Hz, 2 H), 3.36–3.27 (m, 3 H), 2.50–2.20 (m, 10 H), 1.79 (d, *J* = 11.8 Hz, 1 H), 1.67 (d, *J* = 12.0 Hz, 1 H), 1.61–1.58 (m, 1 H), 1.51–1.45 (m, 4 H), 1.23–1.09 (m, 4 H) ppm. LC-MS: *m*/*z* = 551.38 [M+H]^+^

#### Synthesis of 2-Amino-6-(((1*r*,3*s*)-1-(2-(4-(3,3-difluorocyclobutyl)­piperazin-1-yl)­ethyl)-3-(trifluoromethyl)­cyclohexyl)­methyl)-4-(trifluoromethyl)-6,7-dihydro-5H-pyrrolo­[3,4-*d*]­pyrimidin-5-one (**28**)

To a stirred solution of cis-2-[(1*R*,3*S*) or (1*S*,3*R*)-1-[[2-amino-5-oxo-4-(trifluoromethyl)-7*H*-pyrrolo [3,4-*d*]­pyrimidin-6-yl]­methyl]-3-(trifluoromethyl)­cyclohexyl]­acetaldehyde (100 mg, 0.24 mmol, 1 equiv) in chloroform (10 mL) was added 1-(3,3-difluorocyclobutyl)-4,2-piperazine dihydrochloride (70 mg, 0.28 mmol, 1.2 equiv) and stirred at room temperature for 30 min. STAB (100 mg, 0.47 mmol, 2 equiv) was added and the reaction mixture was stirred at room temperature for 14 h. After completion, the reaction mixture was quenched with aqueous NaHCO_3_ solution, extracted with 20% IPA in dichloromethane, washed with brine, dried over anhydrous Na_2_SO_4_ and concentrated *in vacuo*. The crude product was purified by reverse phase preperative HPLC to afford to afford compound **28** (60 mg, 43% yield) as white solid. ^1^H NMR (400 MHz, MeOD): δ 8.44 (s, 1H), 4.48 (d, *J* = 3.2 Hz, 2H), 3.42–3.40 (m, 2H), 3.01–2.99 (m, 5H), 2.74–2.70 (m, 4H), 2.57 (br s, 2H), 2.47–2.36 (m, 4H), 1.96–1.93 (m, 1H), 1.81–1.73 (m, 4H), 1.66–1.63 (m, 1H), 1.61–1.59 (m, H), 1.34–1.28 (m, 4H) ppm. LC MS: *m*/*z* 585.39 [M+H]^+^

#### Synthesis of_ *cis*-2-Amino-6-(((1*r*,3*s*) or (1*s*,3*r*)-1-(2-(4-(Azetidin-1-yl)­piperidin-1-yl)­ethyl)-3-(trifluoromethyl)­cyclohexyl)­methyl)-4-(trifluoromethyl)-6,7-dihydro-5H-pyrrolo­[3,4-*d*]­pyrimidin-5-one (**29**)

To a stirred solution of *cis*-2-((1*R*,3*S*) or (1*S*,3*R*)-1-((2-amino-5-oxo-4-(trifluoromethyl)-5,7-dihydro-6*H*-pyrrolo­[3,4-*d*]­pyrimidin-6-yl)­methyl)-3-(trifluoromethyl)­cyclohexyl)­acetaldehyde (130 mg, 0.31 mmol, 1 equiv) and 4-(azetidin-1-yl)­piperidine hydrochloride (64 mg, 0.37 mmol, 1.2 equiv) in CHCl_3_ (3 mL), NaBH­(OAc)_3_ (97 mg, 0.46 mmol, 1.5 equiv) was added and the reaction mixture was stirred at room temperature for 2 h. After completion, the reaction mixture was concentrated *in vacuo* and purified by reverse-phase preparative-HPLC to afford compound **29** (84 mg, 50% yield) as an off-white solid. ^1^H NMR (400 MHz, DMSO-*d*_6_) δ 7.99–7.91 (m, 2 H), 4.47 (s, 2 H), 3.35–3.26 (m, 2 H), 3.01 (t, *J* = 6.8 Hz, 4 H), 2.78–2.71 (m, 2 H), 2.50–2.36 (m, 1 H), 2.33–2.22 (m, 2 H), 1.96–1.84 (m, 5 H), 1.79 (d, *J* = 11.3 Hz, 1 H), 1.66 (d, *J* = 12.4 Hz, 1 H), 1.60–1.40 (m, 7 H), 1.22–1.09 (m, 5 H) ppm. LC-MS: *m*/*z* = 549.41 [M+H]^+^

#### Synthesis of *cis*-6-((1*r*,3*s*) or (1*s*,3*r*)-1-(2-(1,7-Diazaspiro­[3.5]­nonan-7-yl)­ethyl)-3-(Trifluoromethyl)­cyclohexyl)­methyl)-2-amino-4-(trifluoromethyl)-6,7-dihydro-5H-pyrrolo­[3,4-*d*]­pyrimidin-5-one (**30**)

To a stirred solution of Cis-2-((1*R*,3*S*) or (1*S*,3*R*)-1-((2-amino-5-oxo-4-(trifluoromethyl)-5,7-dihydro-6*H*-pyrrolo­[3,4-*d*]­pyrimidin-6-yl)­methyl)-3-(trifluoromethyl)­cyclohexyl)­acetaldehyde (650 mg, 1.53 mmol, 1 equiv) in CHCl_3_ (60 mL), *tert*-butyl 1,7-diazaspiro[3.5]­nonane-1-carboxylate (416 mg, 1.84 mmol, 1.2 equiv) was added and stirred for 30 min at room temperature. To this stirring solution, STAB (650 mg, 3.06 mmol, 2 equiv) was added and the reaction mixture was stirred at room temperature for 16 h. After the reaction was complete solvent was evaporated and the crude mixture was purified by column chromatography (0–3% MeOH/DCM) to afford compound **30** (750 mg, 77% yield) as white solid. ^1^H NMR (400 MHz, DMSO-*d*_6_) δ 7.99–7.93 (m, 2H), 4.47 (s, 2H), 4.03 (q, *J* = 7.1 Hz, 1H), 3.69 (s, 1H), 3.61 (t, *J* = 6.5 Hz, 1H), 2.84 (s, 2H), 2.49–2.38 (m, 1H), 2.36–2.22 (m, 2H), 2.21–2.09 (m, 1H), 2.07–1.99 (m, 3H), 1.94 (t, *J* = 7.4 Hz, 2H), 1.88–1.78 (m, 2H), 1.69–1.58 (m, 4H), 1.56–1.42 (m, 4H), 1.35 (s, 9H), 1.23–1.09 (m, 3H) ppm. LC-MS: *m*/*z* 635.3 [M+H]^+^. This material was dissolved in diethyl ether (5 mL), 2 M HCl in diethyl ether (50 mL) was added and stirred for 2 h at room temperature. After the reaction was complete solvent was evaporated and the crude mixture was purified by reverse-phase preparative HPLC to afford compound **30** (100 mg, 16% yield) as white solid. ^1^H NMR (400 MHz, DMSO-*d*_6_) δ 11.07 (s, 1H), 9.49–9.45 (m, 1H), 9.32 (s, 1H), 8.02–7.94 (m, 2H), 4.52 (d, *J* = 3.3 Hz, 2H), 3.84 (d, *J* = 6.4 Hz, 2H), 3.54 (t, *J* = 12.2 Hz, 2H), 3.35–3.24 (m, 2H), 3.14 (s, 3H), 2.95 (t, *J* = 9.1 Hz, 1H), 2.67–2.58 (m, 2H), 2.41–2.31 (m, 4H), 2.20–2.14 (m, 1H), 1.93–1.80 (m, 3H), 1.65 (t, *J* = 11.9 Hz, 2H), 1.58–1.47 (m, 2H), 1.29–1.14 (m, 3H) ppm. LC-MS: *m*/*z* 535.2 [M+H]^+^

#### Synthesis of 2-Amino-6-[[1-[2-(1-Methyl-1,7-diazaspiro[3.5]­nonan-7-yl)­ethyl]-3-(trifluoromethyl)­cyclohexyl]­methyl]-4-(trifluoromethyl)-7H-pyrrolo­[3,4-*d*]­pyrimidin-5-one;formic acid (**31**)

1-Methyl-1,7-diazaspiro­[3.5]­nonane dihydrochloride (88 mg, 0.45 mmol, 1.1 equiv) was dissolved in chloroform (4 mL) and 2-[1-[[2-amino-5-oxo-4-(trifluoromethyl)-7H-pyrrolo­[3,4-*d*]­pyrimidin-6-yl]­methyl]-3-(trifluoromethyl)­cyclohexyl]­acetaldehyde (160 mg, 0.38 mmol, 1 equiv) was added. The reaction was stirred for 5 min at room temperature before sodium triacetoxyborohydride (120 mg, 0.57 mmol, 1.5 equiv) was added at 0 °C. The reaction was stirred at room temperature for 2 h before being concentrated *in vacuo* and purified by reverse-phase column chromatography (5–95% MeCN/water with 0.1% formic acid buffer) to afford compound **31** (32 mg, 13% yield) as a white solid. ^1^H NMR (500 MHz, DMSO-*d*_6_) δ 8.25 (s, 2H), 8.06–7.82 (m, 2H), 4.48 (s, 2H), 3.41 (t, *J* = 7.4 Hz, 2H), 3.32 (dd, *J* = 24.8, 14.2 Hz, 2H), 2.92–2.80 (m, 2H), 2.43–2.34 (m, 2H), 2.31 (s, 3H), 2.07–1.87 (m, 4H), 1.89–1.74 (m, 3H), 1.76–1.56 (m, 4H), 1.58–1.39 (m, 4H), 1.28–1.02 (m, 5H). LC-MS: *m*/*z* 549 [M+H]^+^

#### Synthesis of 2-Amino-6-[[1-[2-(1-Ethyl-1,7-diazaspiro[3.5]­nonan-7-yl)­ethyl]-3-(trifluoromethyl)­cyclohexyl]­methyl]-4-(trifluoromethyl)-7H-pyrrolo­[3,4-*d*]­pyrimidin-5-one;formic acid (**32**)

1-Ethyl-1,7-diazaspiro­[3.5]­nonane (24 mg, 0.16 mmol, 1.1 equiv) was dissolved in chloroform (4 mL) and 2-[1-[[2-amino-5-oxo-4-(trifluoromethyl)-7H-pyrrolo­[3,4-*d*]­pyrimidin-6-yl]­methyl]-3-(trifluoromethyl)­cyclohexyl]­acetaldehyde (60 mg, 0.14 mmol, 1 equiv), was added. The reaction was stirred for 5 min at room temperature before sodium triacetoxyborohydride (445 mg, 0.21 mmol, 1.5 equiv) was added at 0 °C. The reaction was stirred at room temperature for 2 h before being concentrated *in vacuo* and purified by reverse-phase column chromatography (5–95% MeCN/water with 0.1% formic acid buffer) to afford compound **32** (21 mg, 21% yield) as a white solid. ^1^H NMR (500 MHz, DMSO-*d*_6_): δ 8.25 (s, 1H), 7.96 (s, 2H), 4.48 (s, 2H), 3.44–3.23 (m, 4H), 2.87 (dd, *J* = 19.6, 11.5 Hz, 2H), 2.64–2.55 (m, 2H), 2.49–2.24 (m, 4H), 2.02–1.85 (m, 4H), 1.81 (d, *J* = 12.1 Hz, 3H), 1.74–1.38 (m, 8H), 1.29–1.06 (m, 3H), 0.92 (t, *J* = 7.1 Hz, 3H). LC-MS: *m*/*z* 563 (M+H)^+^

#### Synthesis of *cis*-2-Amino-6-(((1*r*,3*s*) or (1*s*,3*r*)-1-(2-(4-(Azetidin-1-yl)-3,3-difluoropiperidin-1-yl)­ethyl)-3-(trifluoromethyl)­cyclohexyl)­methyl)-4-(trifluoromethyl)-6,7-dihydro-5H-pyrrolo­[3,4-*d*]­pyrimidin-5-one (**33**)

To a stirred solution of cis-2-[(1*R*,3*S*) or (1*S*,3*R*)-1-[[2-amino-5-oxo-4-(trifluoromethyl)-7*H*-pyrrolo [3,4-*d*]­pyrimidin-6-yl]­methyl]-3-(trifluoromethyl)­cyclohexyl]­acetaldehyde (100 mg, 0.24 mmol, 1 equiv) in chloroform (10 mL) was added 4-(azetidin-1-yl)-3,3-difluoropiperidine dihydrochloride (**10**) (70.4 mg, 0.28 mmol, 1.2 equiv) and stirred at room temperature for 15 min. STAB (100 mg, 0.47 mmol, 2 equiv) was added and the reaction mixture was stirred at room temperature for 14 h. After completion, the reaction mixture was quenched with aqueous NaHCO_3_ solution, extracted with 20% IPA in dichloromethane, washed with brine, dried over anhydrous Na_2_SO_4_ and concentrated. The crude product was purified by reverse phase preperative HPLC to afford **compound 33** (50 mg, 34% yield) as white solid. ^1^H NMR (400 MHz, DMSO-*d*_6_): δ 8.18 (s, 1H), 7.99–7.92 (m, 2H), 4.47 (s, 2H), 3.19–3.13 (m, 6H), 2.75–2.75 (m, 1H), 2.51–2.29 (m, 6H), 1.96–1.92 (m, 2H), 1.80–1.78 (m, 1H), 1.66–1.63 (m, 3H), 1.49 (br s, 5H), 1.24–1.09 (m, 4H) ppm. LC-MS: *m*/*z* 583 [M-H]^−^.

#### Synthesis of *cis*-2-Amino-6-(((1*r*,3*s*) or (1*s*,3*r*)-1-(2-(3-Morpholinoazetidin-1-yl)­ethyl)-3-(trifluoromethyl)­cyclohexyl)­methyl)-4-(trifluoromethyl)-6,7-dihydro-5H-pyrrolo­[3,4-*d*]­pyrimidin-5-one (**34**)

To a stirred solution of *cis*-2-[(1*R*,3*S*) or (1*S*,3*R*)-1-{[2-amino-5-oxo-4-(trifluoromethyl)-5*H*,6*H*,7*H*-pyrrolo­[3,4-*d*]­pyrimidin-6-yl]­methyl}-3-(trifluoromethyl)­cyclohexyl]­acetaldehyde (150 mg, 0.35 mmol, 1 equiv) in CHCl_3_ (15 mL) was added *tert*-butyl 5,5-difluoro-2,7-diazaspiro[3.5]­nonane-2-carboxylate (111 mg, 0.43 mmol, 1.2 equiv) and the mixture was stirred for 30 min at room temperature. Na­(OAc)_3_BH (300 mg, 1.41 mmol, 4 equiv) was added and the reaction mixture was stirred at room temperature for 14 h. After completion, the reaction mixture was quenched with aqueous NaHCO_3_ solution, extracted with dichloromethane, washed with brine, dried over anhydrous Na_2_SO_4_ and concentrated to afford the crude compound (180 mg) as white solid. LCMS: *m*/*z* 671 [M+H]^+^ This was dissolved in dichloromethane (15 mL) and TFA (3 mL) was added dropwise at 0 °C. Reaction mixture was stirred at room temperature for 10 h. Reaction mixture was concentrated and crude product was purified by reverse-phase preparative HPLC to afford compound **34** (60 mg, 36% yield) as an off-white solid. ^1^H NMR (400 MHz, DMSO-*d*_6_) δ 8.25 (s, 1H), 7.91–7.99 (br, 2H), 4.46 (s, 2H), 3.70–3.68 (m, 2H), 3.69–3.24 (m, 6H), 2.49–2.38 (m, 5H), 1.90–1.40 (m, 9H), 1.20–1.08 (m, 3H). LC MS: *m*/*z* 569 [M-H]^+^

#### Synthesis of *cis*-2-Amino-6-{[((1*r*,3*s*) or (1*s*,3*r*)-1-(2-{2-Methyl-5-oxa-2,8-diazaspiro­[3.5]­nonan-8-yl}­ethyl)-3-(trifluoromethyl)­cyclohexyl]­methyl}-4-(trifluoromethyl)-5h,6h,7h-pyrrolo­[3,4-*d*]­pyrimidin-5-one (**35**)

To a stirred solution of Cis-2-[(1*R*,3*S*) or (1*S*,3*R*)-1-{[2-amino-5-oxo-4-(trifluoromethyl)-5*H*,6*H*,7*H*-pyrrolo­[3,4-*d*]­pyrimidin-6-yl]­methyl}-3-(trifluoromethyl)­cyclohexyl]­acetaldehyde (100 mg, 0.24 mmol, 1 equiv) in chloroform (5 mL) 2-methyl-5-oxa-2,8-diazaspiro[3.5]­nonane (**10**) (34 mg, 0.24 mmol, 1 equiv) was added and stirred at rt for 30 min. Then, Na­(OAc)_3_BH (75 mg, 0.35 mmol, 1.5 equiv) was added and the reaction mixture was stirred at room temperature for 14 h. After completion, the reaction mixture was quenched with aqueous NaHCO_3_ solution, extracted with dichloromethane, washed with brine, dried over anhydrous Na_2_SO_4_ and concentrated *in vacuo*. The crude product was purified by reverse-phase preparative HPLC to afford compound **35** (45 mg, 35% yield) as an off-white solid. ^1^H NMR (400 MHz, CDCl_3_): δ 5.70 (br s, 2H), 4.39 (s, 2H), 3.65–3.59 (m, 2H), 3.47–3.32 (m, 4H), 2.89 (d, *J* = 7.8 Hz, 2H), 2.64–2.51 (m, 2H), 2.45–2.33 (m, 6H), 2.26–2.22 (m, 1H), 1.92 (d, *J* = 12.2 Hz, 1H), 1.75–1.70 (m, 2H), 1.58–1.42 (m, 5H), 1.27–1.11 (m, 3H) ppm. LCMS: *m*/*z* 551.34 [M+H]^+^

#### Synthesis of *cis*-2-Amino-6-(((1*r*,3*s*) or (1*s*,3*r*)-1-(2-(7-(Azetidin-1-yl)-4-azaspiro­[2.5]­octan-4-yl)­ethyl)-3-(trifluoro methyl)­cyclohexyl)­methyl)-4-(trifluoromethyl)-6,7-dihydro-5H-pyrrolo­[3,4-*d*]­pyrimidin-5-one (**36**)

##### Step 1: Synthesis of *tert*-Butyl 7-(Azetidin-1-yl)-4-azaspiro[2.5]­octane-4-carboxylate (102)

To a solution of *tert*-butyl 7-oxo-4-azaspiro[2.5]­octane-4-carboxylate (400 mg, 1.78 mmol, 1 equiv) in THF:MeOH (9:1, 10.0 mL) was added 4Å molecular sieves (1.0 g), azetidine (358 mg, 5.33 mmol, 3 equiv), acetic acid (1.2 mL) and sodium cyanoborohydride (335 mg, 5.33 mmol, 3 equiv) at room temperature. The reaction was heated at 65 °C for 16 h. The suspension was filtered and concentrated *in vacuo*. The crude was diluted with sodium bicarbonate solution and extracted with dichloromethane. The organic layer was dried over anhydrous Na_2_SO_4_, filtered and concentrated *in vacuo* to obtain crude compound 102 (400 mg, 84% yield) as colorless liquid, which was used for next step without further purification. LCMS: *m*/*z* 267 [M+H]^+^

##### Step 2: Synthesis of 7-(Azetidin-1-yl)-4-Azaspiro[2.5]­octane (103)

2M HCl in ether (20.0 mL) was added to *tert*-butyl 7-(azetidin-1-yl)-4-azaspiro[2.5]­octane-4-carboxylate (400.0 mg, 1.5 mmol, 1 equiv) and stirred at room temperature for 16 h. The reaction mixture was concentrated *in vacuo* to afford Compound 103 (240 mg, 96% yield) as off white solid as hydrochloride salt, which was used for next step without further purification. LCMS: *m*/*z* 167 [M+H]^+^.

##### Step 3: Synthesis of *cis*-2-Amino-6-(((1*r*,3*s*) or (1*s*,3*r*)-1-(2-(7-(azetidin-1-yl)-4-azaspiro­[2.5]­octan-4-yl)­ethyl)-3-(trifluoro methyl)­cyclohexyl)­methyl)-4-(trifluoromethyl)-6,7-dihydro-5h-pyrrolo­[3,4-*d*]­pyrimidin-5-one (**36**)

A solution of Cis-2-((1*R*,3*S*) or (1*S*,3*R*)-1-((2-amino-5-oxo-4-(trifluoromethyl)-5,7-dihydro-6H-pyrrolo­[3,4-*d*]­pyrimidin-6-yl)­methyl)-3-(trifluoromethyl)­cyclohexyl) acetaldehyde (100 mg, 0.236 mmol, 1 equiv) and 7-(azetidin-1-yl)-4-azaspiro[2.5]­octane (57.33 mg, 0.28 mmol, 1.2 equiv) in chloroform (5 mL) was stirred at room temperature for 5 min. After that NaBH­(OAc)_3_ (100 mg, 0.471 mmol, 2 equiv) was added and the reaction mixture was stirred at room temperature for 12 h. The reaction mixture was diluted with 20% IPA in DCM, washed with saturated sodium bicarbonate solution, dried over anhydrous Na_2_SO_4_, concentrated *in vacuo*, and purified by reverse phase preparative HPLC to afford compound **36** (40 mg, 27% yield) as off white solid as formic acid salt. ^1^H NMR (400 MHz, DMSO-*d*_6_) 8.21 (s, 1H), 8.01 (br s, 1H), 7.93 (br s, 1H), 4.46 (s, 2H), 3.33–2.27 (m, 12H), 1.95–1.91 (m, 2H), 1.78–1.09 (m, 14H), 0.80–0.27 (m, 4H) ppm. LC-MS: *m*/*z* 573 [M-H]

#### Synthesis of (Isobutyryloxy)­methyl *cis*-7-(2-((1*r*,3*s*) or (1*s*,3*r*)-1-((2-Amino-5-oxo-4-(trifluoromethyl)-5,7-dihydro-6*h*-pyrrolo­[3,4-*d*]­pyrimidin-6-yl)­methyl)-3-(trifluoromethyl)­cyclohexyl)­ethyl)-1,7-diazaspiro­[3.5]­nonane-1-carboxylate (Formate salt) (**37**)

##### Step 1: Synthesis of Chloromethyl 7-(2-*cis*-((1*r*,3*s*) or (1*s*,3*r*)-1-((2-Amino-5-oxo-4-(trifluoromethyl)-5,7-dihydro-6*h*-pyrrolo­[3,4-*d*]­pyrimidin-6-yl)­methyl)-3-(trifluoromethyl)­cyclohexyl)­ethyl)-1,7-diazaspiro­[3.5]­nonane-1-carboxylate

To a stirred solution of 6-((cis-1-(2-((1*R*,3*S*) or (1*S*,3*R*)-(1,7-diazaspiro­[3.5]­nonan-7-yl)­ethyl)-3-(trifluoromethyl)­cyclohexyl)­methyl)-2-amino-4-(trifluoromethyl)-6,7-dihydro-5H-pyrrolo­[3,4-*d*]­pyrimidin-5-one (42 mg, 0.08 mmol, 1.0 equiv) in dichloromethane (5 mL) at 0 °C, DIPEA (0.002 mL, 0.01 mmol, 1.5 equiv) followed by chloromethyl chloroformate (0.007 mL, 0.08 mmol, 1.0 equiv) was slowly added. The reaction mixture was stirred at 0 °C for 1 h. After the reaction was complete, DCM was evaporated *in vacuo* to afford the crude compound, which was used for the next step without further purification. LC-MS: *m*/*z* 627 [M+H]^+^

##### Step 2: Synthesis of (Isobutyryloxy)­methyl *cis*-7-(2-((1*r*,3*s*) or (1*s*,3*r*)-1-((2-Amino-5-oxo-4-(trifluoromethyl)-5,7-dihydro-6*H*-pyrrolo­[3,4-*d*]­pyrimidin-6-yl)­methyl)-3-(trifluoromethyl)­cyclohexyl)­ethyl)-1,7-diazaspiro­[3.5]­nonane-1-carboxylate (formate salt) (**37**)

To a stirred solution of crude chloromethyl cis-7-(2-((1*R*,3*S*) or (1*S*,3*R*)-1-((2-amino-5-oxo-4-(trifluoromethyl)-5,7-dihydro-6H-pyrrolo­[3,4-*d*]­pyrimidin-6-yl)­methyl)-3-(trifluoromethyl)­cyclohexyl)­ethyl)-1,7-diazaspiro­[3.5]­nonane-1-carboxylate (50 mg, 0.08 mmol, 1.0 equiv) in chloroform (5 mL), DIPEA (0.011 mL, 0.06 mmol, 0.75 equiv) was added, followed by isobutyric acid (0.011 mL, 0.12 mmol, 1.5 equiv). The reaction mixture was heated in a sealed tube at 80 °C for 16 h. After the reaction was complete, all volatiles were removed *in vacuo* and the crude mixture was purified by reverse-phase preparative HPLC to afford compound 37 (17 mg, 31% yield) as a white solid. ^1^H NMR (400 MHz, DMSO-*d*_6_) δ 8.24 (s, 1H), 8.00–7.93 (m, 2H), 5.63 (d, *J* = 12.4 Hz, 2H), 4.47 (s, 2H), 3.81 (t, *J* = 7.4 Hz, 1H), 3.73 (t, *J* = 7.4 Hz, 1H), 2.88–2.50 (m, 3H), 2.32–2.29 (m, 2H), 2.13–2.07 (m, 1H), 1.99–1.94 (m, 3H), 1.90–1.78 (m, 4H), 1.68–1.58 (m, 4H), 1.51–1.49 (m, 4H), 1.21–1.12 (m, 3H), 1.08–1.04 (m, 6H) ppm. LC-MS: *m*/*z* 677 [M-H]^+^

#### Synthesis of 2,2-Dimethylpropanoyloxymethyl 7-[2-[(1*r*,3*s*) or (1s, 3r)-1-[[2-amino-5-oxo-4-(trifluoromethyl)-7*H*-pyrrolo­[3,4-*d*]­pyrimidin-6-yl]­methyl]-3-(trifluoromethyl)­cyclohexyl]­ethyl]-1,7-diazaspiro­[3.5]­nonane-1-carboxylate (**38**)

##### Step 1: Synthesis of Chloromethyl Ethylsulfanylformate (105)

To a stirred solution of chloromethyl carbonochloridate (5 g, 39.8 mmol, 1.0 equiv) in diethyl ether (30 mL), a mixture of thioethane (9 mL, 119.5 mmol, 3.0 equiv) and triethyl amine (17 mL, 119.5 mmol, 3.0 equiv) was added dropwise at 0 °C. The mixture was stirred at room temperature overnight, then the solid was separated out from the reaction mixture, which was filtered through a sintered funnel and discarded. The filtrate was collected and concentrated *in vacuo* to afford compound **105** (6 g, 99% yield) which was used for the next step without further purification. ^1^H NMR (400 MHz, CDCl_3_) δ 5.75 (s, 2H), 2.93 (q, *J* = 7.6 Hz, 2H), 1.33 (t, *J* = 7.6 Hz, 3H) ppm.

##### Step 2: Synthesis of Ethylsulfanylcarbonyloxymethyl 2,2-dimethylpropanoate (106)

To a stirred solution of pivalic acid (940 mg, 6.14 mmol, 1 equiv) in DIPEA (10 mL) O-(chloromethyl) S-ethyl carbonothioate (627 mg, 6.14 mmol, 1 equiv) was added and stirred the reaction mixture for 2 h at 60 °C. The reaction mixture was diluted with water and was extracted with ethyl acetate. Organic fractions were combined, dried over anhydrous sodium sulfate and concentrated *in vacuo* to afford compound **106** which was used for the next step without further purification. ^1^H NMR (400 MHz, CDCl_3_) δ 5.79 (s, 2H), 2.90 (q, *J* = 7.6 Hz, 2H), 1.33 (t, *J* = 7.6 Hz, 3H) (m, 3H), 1.21 (s, 9H) ppm.

##### Step 3: Synthesis of (2,5-Dioxopyrrolidin-1-yl)­oxycarbonyloxymethyl 2,2-dimethylpropanoate (107)

To a stirred solution of ethylsulfanylcarbonyloxymethyl 2,2-dimethylpropanoate (400 mg, 1.6 mmol, 1 equiv) in dichloromethane (20 mL) was added m-CPBA (254 mg, 3.3 mmol, 2 equiv) under ice-cold condition. Thereafter 1-hydroxypyrrolidine-2,5-dione (383 mg, 3.3 mmol, 2 equiv) was added to the reaction mixture and stirred at room temperature for overnight. The mixture was washed with saturated NaHCO_3_ solution (aq.) and extracted with DCM. Organic fractions were combined, dried over anhydrous sodium sulfate and concentrated *in vacuo* to afford compound **107** which was used for the next step without further purification.

##### Step 3: Synthesis of 2,2-Dimethylpropanoyloxymethyl 7-[2-[(1*r*,3*s*) or (1s, 3r)-1-[[2-amino-5-oxo-4-(trifluoromethyl)-7h-pyrrolo­[3,4-*d*]­pyrimidin-6-yl]­methyl]-3-(trifluoromethyl)­cyclohexyl]­ethyl]-1,7-diazaspiro­[3.5]­nonane-1-carboxylate (**38**)

To a stirred solution of 2-amino-6-[[(1*S*,3*R*) or (1*S*,3*R*)-1-[2-(1,7-diazaspiro­[3.5]­nonan-7-yl)­ethyl]-3-(trifluoromethyl)­cyclohexyl]­methyl]-4-(trifluoromethyl)-7H-pyrrolo­[3,4-*d*]­pyrimidin-5-one (200 mg, 0.38 mmol, 1 equiv) in chloroform (5 mL) was added triethyl amine (0.1 mL, 0.75 mmol, 2 equiv) and stirred for 5 min. Thereafter a solution of (2,5-dioxopyrrolidin-1-yl)­oxycarbonyloxymethyl 2,2-dimethylpropanoate (154 mg, 0.56 mmol, 1.5 equiv) in chloroform (3 mL) was added to the reaction mixture and stirred at room temperature overnight. The reaction mixture was concentrated *in vacuo* and purified by reverse phase preparative HPLC to afford compound **38** (15 mg, 6% yield) as white solid. ^1^H NMR (400 MHz, DMSO-*d*_6_, 100 °C) δ 8.12 (s, 1H), 7.61 (brs, 2H), 5.65 (s, 2H, rotameric pattern observed even at 100 °C), 4.47 (s, 2H), 3.79 (t, *J* = 6.8 Hz, 2H), 3.36 (m, 2H), 2.93–2.88 (m, 2H), 2.67 (s, 2H), 2.48–2.35 (m, 1H), 2.25–2.18 (m, 3H), 2.06–2.02 (m, 2H), 1.88–1.47 (m, 10H), 1.30–1.21 (m, 2H), 1.16 (s, 9H) ppm. LC-MS: *m*/*z* = 693 [M+H]^+^

#### Synthesis of 3-Ethylpentanoyloxymethyl 7-[2-[(1*r*,3*s*) or (1*s*,3*r*)-1-[[2-Amino-5-oxo-4-(trifluoromethyl)-7h-pyrrolo­[3,4-*d*]­pyrimidin-6-yl]­methyl]-3-(trifluoromethyl)­cyclohexyl]­ethyl]-1,7-diazaspiro­[3.5]­nonane-1-carboxylate (**39**)

##### Step 1: Synthesis of Iodomethyl Ethylsulfanylformate (108)

To a stirred solution of **105** (200 mg, 1.29 mmol, 1 equiv) in acetone (10 mL) were added NaHCO_3_ (11 mg, 0.13 mmol, 0.1 equiv) then sodium iodide (390 mg, 2.59 mmol, 2 equiv) and the resulting mixture was stirred at 40 °C for 4 h. Reaction mixture was cooled to room temperature, filtered and the residue was washed with acetone and diethyl ether. Filtrate fractions were combined and concentrated *in vacuo* to get a crude residue which was partitioned between cold pentane (20 mL) and cold water (10 mL). The organic phase was washed successively with a cold solution of 5% aqueous NaHCO_3_, 10% aqueous Na_2_SO_3_, water, then dried (Na_2_SO_4_), filtered and concentrated *in* vacuo to afford compound **108** as yellow oil which was used for the next step without further purification. ^1^H NMR (400 MHz, CDCl_3_) δ 5.98 (s, 2H), 2.93 (q, *J* = 7.6 Hz, 2H), 1.33 (t, *J* = 7.6 Hz, 3H) ppm.

##### Step 2: Synthesis of Ethylsulfanylcarbonyloxymethyl 3-ethylpentanoate (109)

To a stirred solution of 3-ethylpentanoic acid (496 mg, 3.81 mmol, 1 equiv), in DCM and water was added sodium bicarbonate (641 mg, 7.62 mmol, 2 equiv), tetrabutyl ammonium hydrogen sulfate (1.3 g, 3.81 mmol, 1 equiv) successively and stirred for 1 h at room temperature. Thereafter, a solution of iodomethyl ethylsulfanylformate (700 mg, 2.84 mmol, 0.75 equiv) in dichloromethane (1 mL) was added dropwise to the reaction mixture and stirred overnight. The phase was separated, and the aqueous layer was extracted with DCM. Organic fractions were combined, washed with water, dried over anhydrous sodium sulfate and concentrated *in vacuo*. Then diethyl ether was added to the residue and the mixture was stirred for 1 h. The residue was filtered, and filtrate was concentrated *in vacuo* to afford compound **109**, which was used for the next step without further purification. ^1^H NMR (400 MHz, CDCl_3_) δ 5.79 (s, 2H), 2.89 (q, *J* = 7.6 Hz, 2H), 2.28 (d, *J* = 7.2 Hz, 2H), 1.78–1.72 (m, 1H), 1.39–1.17 (m, 7H), 0.87–0.84 (t, *J* = 7.2 Hz, 6H) ppm.

##### Step 3: Synthesis of (2,5-Dioxopyrrolidin-1-yl)­oxycarbonyloxymethyl 3-ethylpentanoate (110)

To a stirred solution of ethylsulfanylcarbonyloxymethyl 3-ethylpentanoate (200 mg, 0.81 mmol, 1 equiv) in chloroform (10 mL) were added *m*-CPBA (123 mg, 1.61 mmol, 2 equiv) followed by 1-hydroxypyrrolidine-2,5-dione (185 mg, 0.16 mmol, 2 equiv) at 0 °C. The reaction mixture was warmed to room temperature and then refluxed for 2 days. The mixture was then cooled to room temperature, washed with saturated NaHCO_3_ (aq.) solution and extracted with dichloromethane. Organic fractions were combined, dried over anhydrous sodium sulfate and concentrated *in vacuo* to afford compound **110** as sticky gum which was used for the next step without further purification.

##### Step 4: Synthesis of 3-Ethylpentanoyloxymethyl 7-[2-[(1*r*,3*s*) or (1*s*,3*r*)-1-[[2-amino-5-oxo-4-(trifluoromethyl)-7h-pyrrolo­[3,4-*d*]­pyrimidin-6-yl]­methyl]-3-(trifluoromethyl)­cyclohexyl]­ethyl]-1,7-diazaspiro­[3.5]­nonane-1-carboxylate (**39**)

To a stirred solution of 2-amino-6-[[(1*S*,3*R*) or (1*R*,3*S*)-1-[2-(1,7-diazaspiro­[3.5]­nonan-7-yl)­ethyl]-3-(trifluoromethyl)­cyclohexyl]­methyl]-4-(trifluoromethyl)-7H-pyrrolo­[3,4-*d*]­pyrimidin-5-one (240 mg, 0.44 mmol, 1 equiv) in chloroform (10 mL) was added triethylamine (0.20 mL, 1.34 mmol, 3 equiv) and stirred for 5 min. Thereafter, a solution of (2,5-dioxopyrrolidin-1-yl)­oxycarbonyloxymethyl 3-ethylpentanoate (271 mg, 0.89 mmol, 2 equiv) in chloroform (3 mL) was added and stirred at room temperature overnight. On completion, the reaction mixture was concentrated *in vacuo* and purified by reverse-phase preparative HPLC. After purification by HPLC, the pure fraction was treated with 0.1% formic acid aqueous buffer at 0 °C and dried under lyopholization to afford compound **39** as formic acid salt (20 mg, 6% yield, white solid). ^1^H NMR (400 MHz, DMSO-*d*_6_, 100 °C) δ 8.19 (s, 1H), 7.60 (m, 2H), 5.63 (s, 2H), 4.46 (s, 2H), 3.75 (t, *J* = 7.2 Hz, 2H), 3.36 (s, 2H), 2.40–2.32 (m, 2H), 2.28–2.22 (m, 2H), 2.20–2.07 (m, 1H), 2.06–2.01 (m, 2H), 2.02–1.93 (m, 2H), 1.94–1.80 (m, 1H), 1.77–1.60 (m, 6H), 1.59–1.41 (m, 5H), 1.38–1.10 (m, 9H), 0.6–0.8 (t, *J* = 7.2 Hz, 6H) ppm. LC-MS: *m*/*z* = 721 [M+H]^+^

#### Synthesis of 2-(2,2-Dimethylbutanoyloxy)­ethyl-cis-7-[2-((1*r*,3*s*) or (1*s*,3*r*)-1-[[2-amino-5-oxo-4-(trifluoromethyl)-7*h*-pyrrolo­[3,4-*d*]­pyrimidin-6-yl]­methyl]-3-(trifluoromethyl)­cyclohexyl]­ethyl]-1,7-diazaspiro­[3.5]­nonane-1-carboxylate (Formate salt) (**40**)

To a stirred solution of crude 2-chloroethyl 7-(2-cis-1-((1*R*,3*S*) or (1*S*,3*R*)-(2-amino-5-oxo-4-(trifluoromethyl)-5,7-dihydro-6H-pyrrolo­[3,4-*d*]­pyrimidin-6-yl)­methyl)-3-(trifluoromethyl)­cyclohexyl)­ethyl)-1,7-diazaspiro­[3.5]­nonane-1-carboxylate (350 mg, 0.55 mmol, 1 equiv) in CHCl_3_ (10 mL) was added DIPEA (0.24 mL, 1.36 mmol, 2.5 equiv) and 2,2 dimethylbutanoic acid (0.2 mL, 0.82 mmol, 1.5 equiv) and the resulting reaction mixture was stirred for 72 h at 110 °C. The reaction mixture was concentrated *in vacuo* and purified by reverse-phase preparative HPLC to afford compound **40** (formate salt) (60 mg, 15% yield) as light yellowish solid. ^1^H NMR (400 MHz, DMSO-*d*_6_): δ 8.15 (s, 1H), 8.05–7.96 (m, 2H), 4.48 (s, 2H), 4.15 (s, 4H), 3.76–3.67 (m, 2H), 3.04–2.74 (m, 3H), 2.47–2.35 (m, 3H), 2.24–2.07 (m, 3H), 2.04–1.98 (m, 2H), 1.96–1.85 (m, 2H), 1.81–1.74 (m, 1H), 1.69–1.64 (m, 2H), 1.63–1.42 (m, 8H), 1.20–1.14 (m, 2H), 1.09 (s, 3H), 1.05 (s, 3H), 0.80–0.75 (m, 3H). LCMS: 721 [M+H]^+^

### *Pf* KRS1,*Cp*KRS,*Cp/Pf*KRS (Triple Mutant) and *Hs*KRS Expression and Purification

The plasmid construct designs, protein expression and purification were carried out as described previously.^17,20^

### Crystallization and Ligand Soaking

Crystals of WT *Cp*KRS and *CpPf*KRS were grown as reported previously. Briefly, protein at 30 mg/mL in storage buffer (25 mM HEPES, 0.5 M NaCl, 5% w/v glycerol, 2 mM TCEP, pH 7.0) was incubated with 5 mM lysine at 4 °C for 1 h prior to setting up crystallsation drops. Crystals were grown by hanging drop vapor diffusion with reservoirs consisting of 25% PEG 3350, 0.2 M lithium sulfate and 0.1 M tris pH 7.8, and drops of 1 μL reservoir and 1 μL protein solution. Crystals were soaked in drops consisting of 1 ul reservoir solution and 1 μL of storage buffer containing 10 mM ligand for 1 h, cryoprotected using reservoir with 33% glycerol, and flash frozen in liquid nitrogen.

### X-ray Data Collection and Processing

Data were collected at various sources as summarized in Table S1. Each data set was integrated using the DIALS automated pipeline and scaled and merged using Aimless. Structures were solved by molecular replacement using the structure of WT *Cp*KRS (PDB 5elo) as the search model in Phaser. Model building was done using Coot, and structures refined using Refmac5 incorporated into the CCP4 suite. Ligand dictionaries were generated using AceDRG. Data collection and refinement statistics are summarized in Table S1. Figures were prepared using PyMOL.

### *Pf*KRS1 and *hs*krs *in vitro* enzyme Assays

These were performed as described previously.^20^

### *P. falciparum* Asexual Blood Stage Assay

Assays against *P. falciparum* were conducted as previously described.^17^ Human erythrocytes were obtained with ethical approval from national blood transfusion services. Cultures of the widely used malaria reference strain of chloroquine-sensitive *P. falciparum* strain 3D7 were maintained in a 5% suspension of human red blood cells cultured in RPMI 1640 medium supplemented with 0.5% Albumax II (available from Gibco Life Technologies, San Diego, CA, cat.no. 11021–037), 12 mM sodium bicarbonate, 0.2 mM hypoxanthine, (pH 7.3), and 20 mg/Liter gentamicin at 37 °C, in a humified atmosphere of 1% O2, 3% CO2 with a gas balance of nitrogen. Growth inhibition of the *Plasmodium falciparum* cultures was quantified in a 10-point dose response curve with a 1 in 3 dilution series from a top assay concentration of 50 μM. This 384 well plate-based fluorescence assay utilizes the binding of SYBRgreen I (Thermo Fisher Scientific/Invitrogen cat.no. S7585) to double stranded DNA, which greatly increases the fluorescent signal at 528 nm after excitation at 485 nm. Mefloquine was used as a drug control to monitor the quality of the assay (Z’ = 0.6 to 0.8, where Z’ is a measure of the discrimination between the positive and negative controls on a screen plate). Dose–response curves were determined from a minimum of 3 independent experiments. Compound bioactivity was expressed as EC50, the effective concentration of compound causing 50% parasite death.

### *In Vitro* Cytotoxicity Studies

HepG2 (Human Caucasian hepatocyte carcinoma cell) cytotoxicity assays were conducted, to estimate potency/potential toxicity in mammalian cell lines. Cells were provided by ECACC with certificate of analysis confirming STR profiling and were mycoplasma tested every time new stabilates were made. Cells were screened utilizing a resazurin-based fluorescence assay. (λex = 528 nm, λem = 590 nm), in a 384 well plate format. HepG2 cells (1 × 10^5^ cells/mL) were incubated with compound in a 10-point, 1 in 3 dilution series from a top concentration of 100 μM, for 72 h. Doxorubicin was used as a drug control to monitor the quality of the assay. After the 72-h incubation period resazurin (0.27 mM in cell culture medium) was added and the plates were incubated for a further 3–4 h before the fluorescent signal was measured using a PHERAstar (BMG Labtech, GER). Compound bioactivity was expressed as EC_50_, the effective concentration of compound causing 50% cell death.

### Microsomal and Hepatocyte Intrinsic Clearance (CLi), Aqueous Solubility Determination by Laser Nephelometry, Parallel Artificial Membrane Permeability (PAMPA), CHI-logD and CYP Inhibition Experiments

These *in vitro* ADME experiments were performed exactly as previously reported.^17^

### Plasma Protein Binding Determination

This assay was performed exactly as previously described.^40^

### Aqueous Solubility Determination by a UHPLC System Equipped with UV/vis Detector and Single-Quadrupole Mass Spectrometer

The aqueous solubility of the test compounds was measured by this method exactly as previously described.^41^

### MDCK-Mdr1 Permeability Assay and Ppg Efflux Ratio Determination

This assay was performed exactly as previously described.^41^

### Human Ether-À-Go-Go Related Gene (hERG) K+ Assay (Eurofins)

Compounds were tested for inhibition of the human ether-à-go-go-related gene (hERG) K+ channel using automated whole-cell (CHO) patch clamp electrophysiology.^42^ Five-point concentration–response curves were generated on 2 occasions using 10-fold serial dilutions from the maximum final assay concentration. The degree of inhibition (%) was obtained by measuring the tail current amplitude, which is induced by a one second test pulse to −40 mV after a two second pulse to +20 mV, before and after drug incubation (the difference current was normalized to control and multiplied by 100 to obtain the percent of inhibition). Concentration (log) response curves were fitted to a logistic equation (three parameters assuming complete block of the current at very high concentrations of the test compound to generate estimates of the 50% inhibitory concentration (IC_50_). The concentration–response relationship of each compound was constructed from the percentage reductions of current amplitude by sequential concentrations.

### *In Vitro* Safety Pharmacology Profiling (Eurofins)

Compounds were tested at Eurofins against Eurofins SafetyScreen44 Panels at a single concentration (10 μM).

### Kinase Selectivity Panel

Compounds were tested at the MRC Protein Phosphorylation and Ubiquitylation Unit (MRC PPU, University of Dundee) in their premier screen^43^ at a single concentration (10 μM).

### Mouse and Rat Pharmacokinetics

Female balb/c mice and female Han Wistar rats were obtained from Charles River Laboratoris, UK. Animals were maintained under a 12-h light/12-h dark cycle. Temperature and relative humidity were maintained between 20 and 24 °C and 45–65% respectively. Food and water were supplied *ad-libitum* throughout. All animals received a minimum of 7 days acclimatization and were randomized prior to the start of study. Animals were not blinded as all animals in each pharmacokinetic study received the same dose, and results are based on objective blood concentration measurements. Dose formulations were prepared on the day of dosing. Intravenous formulations were dosed at a volume of 5 mL/kg in a vehicle of 5–10% DMSO and up to 60% PEG400 (depending on the solubility of individual compounds tested) in water. Oral formulations were dosed at a volume of 10 mL/kg for mice, and 5 mL/kg for rats, in a vehicle of 0.5% hydroxypropylmethylcellulose, 0.4% Tween 80, and 0.5% benzyl alcohol. Dose formulations were dosed to 3 animals per administration route, per compound. Serial blood samples (10 μL for mice, 20 μL for rats) were collected from the lateral tail vein at 5, 15, and 30 min, 1, 2, 4, 6, 8, and 24 h postdose. Mouse samples were diluted in 10 volumes of distilled water, while rat samples were diluted in 3 volumes of distilled water. All samples were stored at -20 °C until analysis. The concentration of each compound in mouse or rat blood was determined by UPLC-MS/MS. Pharmacokinetic parameters were derived from the blood concentration time curve using PKsolutions software v 2.0 (Summit Research Services, USA) or Phoenix WinNonLin software (Certara, USA).

### *In Vivo Antimalarial* Efficacy Studies in *P. falciparum*

Compounds were tested in the murine *P. falciparum* SCID model essentially as described.^17^

### Dog Pharmacokinetics and Bioanalytical Methods

Compound **30** was administered intravenously to three male marshall beagle dogs at a dose of 0.3 mg/kg. Blood samples were collected serially from the cephalic vein over a period of up to 48 h postdose using heparinized syringes. Plasma was obtained by centrifugation and aliquots of plasma (20 μL) were stored at – 15 °C or below until PK bioanalysis.

Plasma samples were deproteinized with 30% methanol/70% acetonitrile containing 0.03 μg/mL of propranolol as an internal standard (IS) followed by centrifugation. The obtained supernatant was filtrated, and then the filtrate was analyzed by high-performance liquid chromatography with tandem mass spectrometry (LC-MS/MS).

The concentration of compound **30** were measured by the LC-MS/MS system consisting of Shimadzu 20A HPLC system (Shimadzu, Kyoto, Japan) and Triple Quad 6500 (Sciex, Framingham, MA, USA). The ionization mode was positive electrospray ionization. Chromatography was performed using an L-column IDS (5 μm, 2.1 mm i.d. × 150 mm; Chemicals Evaluation and Research Institute, Tokyo, Japan) at a column temperature of 40 °C with a flow rate of 0.5 mL/min. The mobile phases were water containing 0.02% formic acid (A) and acetonitrile containing 0.02% formic acid (B). The following gradient elution of the mobile phases was employed: 0 min, 2% (B); 0.5 min, 2% (B); 3 min, 99% (B); 5 min, 99% (B); 5.01 min, 2% (B); 6 min, 2% (B). The assay run time was 6 min.

### Ames^24,25^ ^,^

The exploratory reverse mutation assay of compound **30** was conducted with preincubation at 37 °C for 20 min, using *S. typhimurium* TA100, TA1535, TA98, TA1537, and *E. coli* WP2*uvrA* in the presence or absence of S9 mix. The S9 mix included cofactors and S9 fraction from liver homogenate of male Sprague–Dawley rats treated with phenobarbital and 5,6-benzoflavone. The 7 doses from 6.86 to 5000 μg/plate with a common ratio of 3 were tested in triplicate. The concurrent negative and positive controls were set for the respective bacterial strains. Dimethyl sulfoxide was used as the vehicle and negative control.

### *Ex Vivo* Studies against Field Isolates of *P. falciparum* and *P. vivax*

This study received ethical approval from the Ethics Committee of the Centro de Pesquisa em Medicina Tropical (CEPEM) in Rondônia, Brazil (CAAE 58738416.1.0000.0011). Prior to blood collection, all participants provided written informed consent, and a trained nurse conducted the sample collection. Clinical isolates of *P. falciparum* were obtained in March and April 2022 from patients recruited at CEPEM, located in Porto Velho, Rondônia, within the Brazilian Western Amazon region. Only individuals presenting monoinfections with *P. falciparum*, parasitemia levels between 2,000 and 80,000 parasites/μL, and at least 70% of parasites in the ring stage were included in the study. Patients who had taken antimalarial medications within the past month or exhibited severe malaria symptoms were excluded. The study enrolled a total of 31 patients from this malaria-endemic region, including 20 with *P. vivax* infections and 11 with *P. falciparum* infections. Peripheral venous blood samples (5 mL) were collected from each participant using heparin-containing tubes and were immediately utilized for *ex vivo* drug susceptibility assays. The assay plates, preloaded with diluted antimalarial compounds, were prepared using test compounds (**12** and **30**) and control drugs (artesunate and chloroquine). Stock solutions of these compounds were initially prepared at 2 mM in DMSO (diluted from a primary stock solution). Subsequent dilutions in assay medium were performed at a 20,000-fold and 40-fold ratio, yielding an initial drug concentration of 0.1 μM for artesunate and 50 μM for chloroquine as well as compounds **12** and **30**. A 2-fold serial dilution was then performed, followed by the transfer of 20 μL of each dilution into the *ex vivo* assay plate, where the compounds were further diluted 10-fold in the final assay medium containing the parasites. For infected blood preparation, whole blood samples (5 mL) were centrifuged at 800 g for 10 min, after which plasma and the buffy coat were removed. The remaining red blood cell (RBC) pellet was washed with RPMI 1640 medium and diluted at a 50% hematocrit before passing through a CF11 cellulose column for leukocyte depletion.^44^ Following this step, the blood was centrifuged, and the resulting packed RBCs containing infected parasites (iRBCs) were diluted to a 2% hematocrit using complete RPMI 1640 medium supplemented with 20% human serum. Parallel control assays using the *P. falciparum* 3D7 laboratory strain were conducted in the same medium. For drug exposure, 180 μL of iRBCs was distributed into each well of the drug-preloaded plates. Parasite maturation from the ring to schizont stage was conducted in a hypoxia-controlled incubator (5% O_2_, 5% CO_2_, 90% N_2_) at 37 °C for 40 to 47 h. Drug-free control wells containing iRBCs were incubated in complete medium under identical conditions. The incubation period ended when at least 40% of the ring-stage parasites in the control wells developed into schizonts (defined as having at least three distinct nuclei). To assess parasite growth, thick blood films were prepared from each well, air-dried, stained with 5% Giemsa solution for 30 min, and analyzed microscopically. The proportion of schizonts among 200 asexual-stage parasites was determined for each drug concentration and normalized relative to the drug-free control wells, which represented 100% growth.^45^ The half-maximal effective concentration (EC_50_) values were calculated using curve-fitting software (OriginLab Corporation, Northampton, MA, USA) by comparing parasite growth across drug concentrations to the drug-free controls.

### *P. vivax* Liver Stage Assays

Compound activity against *P. vivax* liver stages was performed using the version 2 protocol as previously described using human hepatocytes (BioIVT lot BGW).^31^

### *P. falciparum* Immature and Mature Gametocyte Viability Assays

The gametocytocidal activity was determined on immature gametocytes (>80% stage II/III) and mature gametocytes (>90% stage V) using *P. falciparum* NF54-pfs16-GFP-Luc parasites, for 48 h drug pressure for three independent repeats each in technical triplicates as previously described.^46^

### *P. falciparum* Dual Gamete Formation Assay (*Pf*DGFA)

This assay was performed essentially as described previously described.^33^ Briefly, mature stage V gametocytes were incubated in 384 well plates with test compounds at 37 °C for 48 h before gametogenesis was triggered by adding xanthurenic acid and lowering the temperature to 4 °C for 4 min. Plates were then warmed for 5 min to 28 °C and then male gametogenesis (exflagellation) was recorded by brightfield timelapse microscopy around 15 min postactivation. Plates were then incubated at room temperature for a further 24 h and female gametogenesis recorded using an anti-Pfs25 Cy3-labeled antibody and fluorescence microscopy. Male and female gametes were detected by custom automated algorithms and percentage inhibition calculated with respect to positive (Gentian Violet) and negative (DMSO) controls.

### Parasite Reduction Ratio (PRR) Studies

The *in vitro* PRR assay was used to determine onset of action and rate of killing as previously described.^36^ *P. falciparum* was exposed to **12** and **30** at a concentration corresponding to 10 × EC_50_. The number of viable parasites at each time point was determined as described.^36^ Four independent serial dilutions were done with each sample to correct for experimental variation and the error bars shown are the standard deviation. Previous results reported on standard antimalarials tested at 10 × EC_50_ using the same conditions are shown for comparison.^36^ The following reagent was obtained through BEI Resources, NIAID, NIH: *Plasmodium falciparum*, Strain 3D7A, MRA-151, contributed by David Walliker. The human biological samples were sourced ethically, and their research use was in accordance with the terms of the informed consents under an IRB/EC-approved protocol.

### Minimum Inoculum of Resistance (MIR) Studies

Resistance selection studies were performed as described, including methods of whole-genome sequence analysis.^47^

## Supplementary Material





## Data Availability

PDB codes for wild type *C. parvum* KRS with bound compounds are **1** (9R2C), **8** (9R3R), **12** (9R32) and **22** (9R3F). PDB codes for *C. parvum* *“Pf”* KRS with bound racemic compound **30** (9R3G). Authors will release the atomic coordinates upon article publication.

## Notes

Manuscript key *in vitro* data table with PAINS alert analysis and SMILES strings (CSV)
X-ray crystallographic data collection and refinement statistics; oral mouse pharmacokinetic data for compound **4**; *in vitro* safety panel results for compounds **7** and **8**; kinase panel results for compound **8**; compound **12** electron density; oral mouse pharmacokinetics for compounds **10** and **12**; compounds included in the generation of the FMO–DFTB *Pf*KRS1 pIC_50_ predictive model; summary of resistant selections for compounds **11** and **30**; list of mutations from whole-genome sequencing of compound **30** resistant clone; LCMS and ^1^HNMR for final compounds (PDF)

## □

Recursion Pharmaceuticals Inc., Salt Lake City, Utah 84101, United States

## ■

School of Biomedical Sciences, Faculty of Medicine & Health, University of New South Wales, Sydney 2052, Australia.

## Abbreviations

DAST: diethylaminosulfur trifloride
DBU: 1,8-diazabicyclo­[5.4.0]­undec-7-ene
DIBAL: diisobutylaluminum hydride
DIPEA: N,N-diisopropylethylamine
DCM: dichloromethane
DMA: dimethylacetamide
DMAP: 4-(dimethylamino)­pyridine
DMF: dimethylformamide
DMSO: dimethyl sulfoxide
HEPES: 2-[4-(2-hydroxyethyl)­piperazin-1-yl]­ethanesulfonic acid
LDA: lithium disopropylamide
mCPBA: meta-chloroperbenzoic acid
NMP: *N*-methyl-2-pyrrodione
PEG: polyethylene glycol
STAB: sodium triacetoxyborohydride
TBAF: tetrabutylammonium fluoride
TCEP: tris­(2-carboxyethyl)­phosphine
TFA: trifluoroacetic acid
THF: tetrahydrofuran
TRIS: tris­(hydroxymethyl)­aminomethane
UPLS-MS/MS: ultraperformance liquid chromatography with tandem mass spectrometry.
