# Supplementary material for: Structure-Guided Optimization of Novel Inhibitors of Plasmodium Lysyl-tRNA Synthetase with Multistage Activity against Malaria Parasites
Source: J Med Chem. 2026 Jun 2;69(11):13820–55. doi: 10.1021/acs.jmedchem.6c00823 (PMC13266985; doi:10.1021/acs.jmedchem.6c00823)
Supplement: Supplementary file 2 [file jm6c00823_si_002.pdf]

**Supporting information**

# Structure guided optimization of novel inhibitors of *Plasmodium* lysyl-tRNA synthetase with multistage activity against malaria parasites

Barbara Forte[1,25], Fiona Bellany[1,25], Peter S. Campbell[1,25], Giulia Chemi[1,26], Alice Dawson[1], Mark Anderson[1], Yaw Aniweh[2], Anna Y. Burkhard[3,4], Anna Caroline Campos Aguiar[5] , Alisje Churchyard[6] , Caitlin A Cooper[7], Amália dos Santos Ferreira[8], Mufuliat Toyin Famodimu[6,9] , Francis G. Fang[10], Xiao Hu[1], Tonnie Huijs[11], Delphine Baud[12], Chimed Jansen[1], María Belén Jiménez Díaz[13], Roger Bonnert[12], Susan Boyd[12], Benigno Crespo-Fernandez[14], Branko Mitasev[10], Simone Montagna[1], Sachel Mok[3,4,15], Dinakaran Murugesan[1], Sunil K. Narwal[3,4], Neil R. Norcross[1], John Okombo[3.4], Heekuk Park[4,15], Caroline Peet[1], Dhelio B. Pereira[16], John M. Post[1], Janette Reader[17], Jennifer Riley[1], David A. Robinson[1], Raku Shinkyo[10], Frederick R. C. Simeons,[1] Laura Simpson[1], Alasdair Smith[1], Dennis Smith[12], Josefine Striepen[3,4], Carolina B. G. Teles[8], Rianne van der Laak[11], Anne-Catrin Uhlemann[4,15], Amélie Vantaux[18], Caroline Wilson[1], Benoît Witkowski[19], Gavin Wood[1], Tomas Yeo[3,4], Fabio Zuccotto[1], Iñigo Angulo-Barturen[13], Jake Baum[6,27], Judith M. Bolscher[11], Rafael Victorio Carvalho Guido[19], Lyn-Marié Birkholtz[17,20], Michael J. Delves[6,9], Laurent Dembele[21], David A. Fidock[3,4,15], Francisco Javier Gamo[14], Dennis E Kyle[7], Steven P. Maher[7], Jean Popovici[22,23], Chris Walpole[24], Fabian Gusovsky[10], Paul A. Willis[12], Kevin D. Read[*,1], Ian H. Gilbert[*,1], Beatriz Baragaña[*,1]

[1]Drug Discovery Unit, Division of Biological Chemistry and Drug Discovery, Faculty of Life Sciences, University of Dundee, Dundee, DD1 5EH, UK

[2]West African Centre for Cell Biology of Infectious Pathogens (WACCBIP), College of Basic and Applied Sciences, University of Ghana, LG54, Accra, Ghana

[3]Department of Microbiology & Immunology, Columbia University Irving Medical Center, New York, NY, 10032, USA

[4]Center for Malaria Therapeutics and Antimicrobial Resistance, Division of Infectious Diseases, Department of Medicine, Columbia University Irving Medical Center, New York, NY, 10032, USA

[5]Department of Microbiology, Immunology, and Parasitology, Federal University of São Paulo, CEP 04023-062, São Paulo, SP, Brazil

[6]Department of Life Sciences, Imperial College, London, SW7 2AZ, UK

[7]Center for Tropical and Emerging Global Diseases, University of Georgia, Athens, GA, United States

[8]Oswaldo Cruz Foundation, Leishmaniasis and Malaria Bioassay Platform, CEP 76812-245, Porto Velho, Rondônia, Brazil

[9] Department of Infection Biology, London School of Hygiene and Tropical Medicine, London, WC1E 7HT, UK

[10]Eisai, Inc. 35 Cambridge Park Drive, Suite 200, Cambridge, MA, 02140, USA

[11]TropIQ Health Sciences, Nijmegen, 6534 AT, The Netherlands

[12]MMV Medicines for Malaria Venture, ICC, 1215 Geneva, Switzerland

[13]The Art of Discovery, Derio, 48160, Spain

[14]Global Health Medicines R&D, GSK, Tres Cantos, Madrid, 28760, Spain

[15]Division of Infectious Diseases, Department of Medicine, Columbia University Irving Medical Center, New York, NY, 10032, USA

[16]Research Center in Tropical Medicine of Rondônia, CEP 76812-329, Porto Velho, RO, Brazil

[17]Department of Biochemistry, Genetics and Microbiology, Institute for Sustainable Malaria Control University of Pretoria, Hatfield, Pretoria, 0028, South Africa

[18]Malaria Molecular Epidemiology Unit, Institut Pasteur du Cambodge, Phnom Penh, 120210, Cambodia

[19]São Carlos Institute of Physics, University of São Paulo, CEP 13563-120, São Carlos, SP, Brazil

[20]Department of Biochemistry, Stellenbosch University, Stellenbosch, Matieland 7602, South Africa
[21]Univerité des Sciences, des Techniques et des Technologies de Bamako (USTTB), Parasite and Microbe Research and Training Centre (P-MRTC), Faculty of Pharmacy, Point G, BP:1805, Bamako, Mali
[22] Malaria Research Unit, Institut Pasteur du Cambodge, Phnom Penh, 120210, Cambodia
[23] Infectious Disease Epidemiology and Analytics G5 Unit, Institut Pasteur, Université Paris Cité, 75015, Paris, France
[24]Structural Genomics Consortium, Research Institute of the McGill University Health Centre, Montreal, QC H4A 3J1, Canada
[25]These authors contributed equally
Present address:
[26]Recursion Pharmaceuticals Inc, Salt Lake City, Utah 84101, United States.
[27]School of Biomedical Sciences, Faculty of Medicine & Health, University of New South Wales, Sydney 2052, Australia.

**Corresponding authors information**
Beatriz Baragaña (b.baragana@dundee.ac.uk)
Ian H. Gilbert (ihgilbert@dundee.ac.uk)
Kevin D. Read (kread@dundee.ac.uk)

**Table of Contents**


| | |
|---|---|
| **Table S1:** Data collection and refinement statistics | S4 |
| **Figure S1 and Table S2:** Oral mouse pharmacokinetics for compound 4 | S5 |
| **Table S3:** *In vitro* safety panel results for compound 7 at 10 μM | S6 |
| **Table S4:** *In vitro* safety panel results for compound 8 at 10 μM | S8 |
| **Table S5:** Dundee kinase panel results for compound 8 at 10 μM | S10 |
| **Figure S2:** Compound 12 electron density | S12 |
| **Figure S3 and Table S6:** Oral mouse pharmacokinetics for compound 10 and 12 | S13 |
| **Figure S4:** Compounds included to generate the FMO-DFTB *Pf*KRS1 pIC50 predictive model | S14 |
| **Table S7:** Summary of resistant selection results for compound 11 | S15 |
| **Table S8:** Summary of resistant selection results for compound 30 | S15 |
| **Table S9:** List of mutations from whole-genome sequencing of compound 30 resistant clone | S15 |
| LCMS for final compounds | S16 |
| 1HNMR for key compounds | S62 |

**Table S1: Data collection and refinement statistics**

| | **1** | **8** | **12** | **30** (racemic) | **22** |
|---|---|---|---|---|---|
| Protein | WT *Cp*KRS | WT *Cp*KRS | WT *Cp*KRS | *CpPf*KRS | WT *Cp*KRS |
| X-ray source | Diamond I03 | ESRF ID30-A | Rigaku Micromax-007 HF | Diamond I03 | Rigaku Micromax-007 HF |
| Detector | DECTRIS PILATUS 6M | DECTRIS PILATUS 2M | RIGAKU SATURN 944+ | DECTRIS PILATUS 6M | RIGAKU SATURN 944+ |
| Wavelength (Å) | 0.97623 | 0.966 | 1.5418 | 0.97625 | 1.5418 |
| Space group | $P\ 2\ 2_1\ 2_1$ | $P\ 2\ 2_1\ 2_1$ | $P\ 2_1$ | $P\ 2\ 2_1\ 2_1$ | $P\ 2\ 2_1\ 2_1$ |
| Unit cell lengths (*a, b, c* Å) | 72.595, 116.129, 142.036 | 72.76, 116.26, 142.09 | 73.15, 119.43, 142.76 | 72.808, 116.579, 142.991 | 73.152, 116.852, 143.262 |
| Unit cell angles ($\alpha,\beta,\gamma$ °) | 90, 90, 90 | 90, 90, 90 | 90, 90.13, 90 | 90, 90, 90 | 90, 90, 90 |
| Resolution range (Å) | 116.16-1.84 (1.85-1.84) | 47.36 – 1.50 (1.53-1.50) | 142.76 – 1.90 (1.93- 1.90) | 61.83 – 1.90 (1.949-1.90) | 46.88 – 2.30 (2.37 – 2.30) |
| Total no. of reflections | 656934 (4721) | 906596 (45313) | 1298362(41638) | 1321557 (65932) | 737840 (39471) |
| Total unique reflections | 104930 (1069) | 191914 (9494) | 187306 (8580) | 96515 (4715) | 54785 (4335) |
| Redundancy | 6.3 (4.4) | 4.7 (4.8) | 6.9 (4.9) | 13.7 (14.0) | 13.5 (9.1) |
| Completeness | 100 (97) | 99.7 (100) | 97.3 (90.0) | 100 (99.7) | 99.1 (96.9) |
| $R_{sym}$ | 0.088 (0.807) | 0.057 (0.851) | 0.093 (0.658) | 0.162 (1.668) | 0.174 (0.414) |
| $R_{pim}$ | 0.053 (0.580) | 0.044 (0.655) | 0.055(0.514) | 0.065 (0.662) | 0.066 (0.208) |
| $\langle I\rangle/\langle\sigma\rangle$ | 14.4 (2.0) | 11.4 (1.5) | 11.8 (1.9) | 12.8 (2.0) | 10.1 (3.7) |
| $CC_{1/2}$ | 0.999 (0.653) | 0.999 (0.542) | 0.997 (0.706) | 0.998 (0.685) | 0.985 (0.957) |
| $R_{work}$ / $R_{free}$ | 16.22 / 19.33 | 15/76 / 18.06 | 18.02 / 20.64 | 16.00 / 18.97 | 21.37 / 26.12 |
| B-factors | | | | | |
| Protein (A/B/C/D) | 31.1 / 30.9 /-/ - | 23.8 / 23.9 /-/- | 20.5 / 22.5 / 23.6 / 25.9 | 25.4 / 26.5 /-/- | 15.4 / 16.9 /-/- |
| Ligand (A/B/C/D) | 23.0 / 23.3 /-/ - | 17.0 / 16.6 /-/- | 15.2 / 15.8 / 16.5 / 18.6 | 19.3 / 19.4 /-/- | 10.6 / 10.3 /-/- |
| Lysine (A/B/C/D) | 26.6 / 26.7 / - / - | 15.4 / 16.2 /-/- | 12.1 / 15.7 / 15.5 / 15.9 | 17.1 / 18.3 /-/- | 6.4 / 9.3 /- / - |
| Waters | 38.6 | 33.8 | 25.8 | 30.8 | 17.2 |
| R.m.s. deviations | | | | | |
| Bond lengths (Å) | 0.0087 | 0.116 | 0.0074 | 0.0083 | 0.0091 |
| Bond angles (°) | 1.6883 | 1.9696 | 1.5428 | 1.6776 | 1.6729 |
| Ramachandran favoured / allowed / disallowed (%, all chains) | 99 / 1/ 0 | 98 / 2 / 0 | 98 / 2 / 0 | 98 / 2 / 0 | 98 / 2 / 0 |
| | | | | | |
| PDB code | 9r2c | 9r3r | 9r32 | 9r3g | 9r3f |

**Figure S1 and Table S2: Oral mouse pharmacokinetics for compound 4 following a 10 mg/kg single oral administration to the female Balb/c mouse (n=3). Mean total blood concentration (blue), mean free blood concentration (green) and *P. falciparum* (3D7) asexual blood stage $EC_{90}$ (red dashed line).**

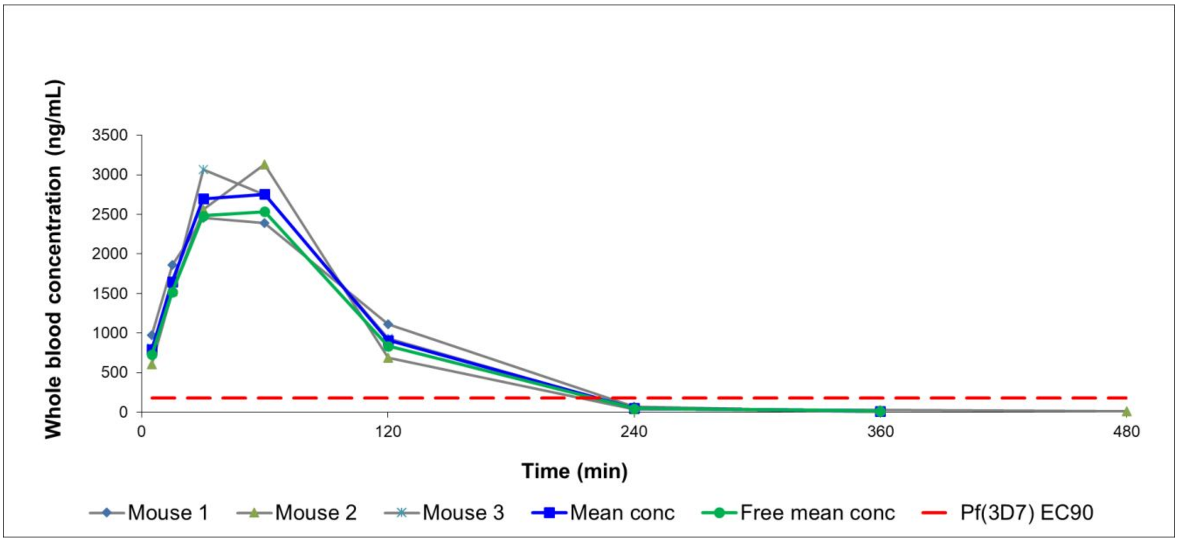


| **Compound** | **4** |
|---|---|
| Route | PO |
| Dose (mg/kg) | 10 |
| $C_{max}$ (ng/mL) | 2756 |
| $T_{max}$ (h) | 1 |
| $AUC_{0\text{-}8h}$ (ng-min/mL) | 300389 |
| Mouse PPB Fu | 0.92 |
| *Pf*(3D7) $EC_{90}$ (ng/mL) | 175.7 |

## Table S3: *In vitro* safety panel results for compound 7 at 10 μM

| Binding Assay | % Inhibition of Control Specific Binding | % of Control Specific Binding | Reference Compound | $IC_{50}$ Ref (M) | Ki Ref (M) |
|---|---|---|---|---|---|
| A2A (h) (agonist radioligand) | 10 | 90.2 | NECA | 2.8E-08 | 2.3E-08 |
| alpha 1A (h) (antagonist radioligand) | 0 | 99.5 | WB 4101 | 4.1E-10 | 2.1E-10 |
| alpha 2A (h) (antagonist radioligand) | -10 | 109.9 | yohimbine | 6.8E-09 | 3.0E-09 |
| beta 1 (h) (agonist radioligand) | 6 | 94.2 | atenolol | 6.4E-07 | 3.6E-07 |
| beta 2 (h) (antagonist radioligand) | 7 | 92.6 | ICI 118551 | 3.5E-10 | 1.2E-10 |
| BZD (central) (agonist radioligand) | -5 | 105.4 | diazepam | 1.4E-08 | 1.1E-08 |
| CB1 (h) (agonist radioligand) | -12 | 111.5 | CP 55940 | 1.1E-09 | 9.6E-10 |
| CB2 (h) (agonist radioligand) | 15 | 85.2 | WIN 55212-2 | 1.7E-09 | 1.1E-09 |
| CCK1 (CCKA) (h) (agonist radioligand) | -7 | 107.4 | CCK-8s | 1.2E-10 | 9.1E-11 |
| D1 (h) (antagonist radioligand) | -5 | 105.1 | SCH 23390 | 4.5E-10 | 1.8E-10 |
| D2S (h) (agonist radioligand) | 9 | 90.6 | 7-OH-DPAT | 2.7E-09 | 1.1E-09 |
| ETA (h) (agonist radioligand) | 1 | 99.1 | endothelin-1 | 1.1E-10 | 5.4E-11 |
| NMDA (antagonist radioligand) | 8 | 91.8 | CGS 19755 | 1.9E-07 | 1.5E-07 |
| H1 (h) (antagonist radioligand) | -7 | 106.8 | pyrilamine | 2.2E-09 | 1.4E-09 |
| H2 (h) (antagonist radioligand) | 1 | 99.0 | cimetidine | 4.4E-07 | 4.2E-07 |
| MAO-A (antagonist radioligand) | 14 | 85.9 | clorgyline | 2.2E-09 | 1.3E-09 |
| M1 (h) (antagonist radioligand) | -4 | 103.9 | pirenzepine | 3.2E-08 | 2.7E-08 |
| M2 (h) (antagonist radioligand) | -23 | 122.9 | methoctramine | 3.1E-08 | 2.2E-08 |
| M3 (h) (antagonist radioligand) | 3 | 96.7 | 4-DAMP | 9.6E-10 | 6.9E-10 |
| N neuronal alpha 4beta 2 (h) (agonist radioligand) | -1 | 101.5 | nicotine | 6.0E-09 | 2.0E-09 |
| delta (DOP) (h) (agonist radioligand) | -3 | 103.4 | DPDPE | 3.7E-09 | 2.2E-09 |
| kappa (KOP) (agonist radioligand) | 3 | 96.6 | U 50488 | 8.8E-10 | 5.9E-10 |
| mu (MOP) (h) (agonist radioligand) | 11 | 88.7 | DAMGO | 1.2E-09 | 5.0E-10 |
| 5-HT1A (h) (agonist radioligand) | -8 | 107.7 | 8-OH-DPAT | 6.2E-10 | 3.1E-10 |
| 5-HT1B (antagonist radioligand) | -5 | 105.3 | serotonin | 1.8E-08 | 1.1E-08 |
| 5-HT2A (h) (agonist radioligand) | 5 | 94.8 | (±)DOI | 2.4E-10 | 1.8E-10 |
| 5-HT2B (h) (agonist radioligand) | -11 | 111.2 | (±)DOI | 6.8E-09 | 3.4E-09 |
| 5-HT3 (h) (antagonist radioligand) | -4 | 103.8 | MDL 72222 | 1.4E-08 | 9.4E-09 |
| GR (h) (agonist radioligand) | -8 | 107.6 | dexamethasone | 3.1E-09 | 1.6E-09 |
| AR (h) (agonist radioligand) | 2 | 97.5 | testosterone | 4.3E-09 | 1.9E-09 |
| V1a (h) (agonist radioligand) | 7 | 92.8 | [d(CH2)51,Tyr(Me)2]-AVP | 2.2E-09 | 1.4E-09 |
| Ca2+ channel (L, dihydropyridine site) (antagonist radioligand) | 0 | 99.5 | nitrendipine | 2.8E-10 | 1.8E-10 |
| Potassium Channel hERG (human)- [3H] Dofetilide | 1 | 98.6 | Terfenadine | 4.5E-08 | 3.1E-08 |
| KV channel (antagonist radioligand) | -8 | 108.3 | alpha -dendrotoxin | 1.2E-10 | 1.0E-10 |
| Na+ channel (site 2) (antagonist radioligand) | 14 | 85.9 | veratridine | 1.1E-05 | 9.6E-06 |
| norepinephrine transporter (h) (antagonist radioligand) | -4 | 103.9 | protriptyline | 3.8E-09 | 2.8E-09 |
| dopamine transporter (h) (antagonist radioligand) | 6 | 93.6 | BTCP | 1.2E-08 | 6.4E-09 |
| 5-HT transporter (h) (antagonist radioligand) | -2 | 102.4 | imipramine | 3.7E-09 | 1.7E-09 |

| Enzyme Assay | % Inhibition of Control Values | % of Control Values | Reference Compound | $IC_{50}$ Ref (M) |
|---|---|---|---|---|
| COX1(h) | 13 | 87.4 | Diclofenac | 1.1E-08 |
| COX2(h) | -3 | 103.0 | NS398 | 1.1E-07 |

| | | | | |
|---|---|---|---|---|
| PDE3A (h) | -27 | 127.2 | milrinone | 3.2E-07 |
| PDE4D2 (h) | -8 | 108.2 | Ro 20-1724 | 2.3E-07 |
| Lck kinase (h) | 1 | 98.9 | staurosporine | 2.8E-08 |
| acetylcholinesterase (h) | -2 | 101.6 | galanthamine | 8.6E-07 |

## Table S4: *In vitro* safety panel results for compound 8 at 10 μM

| Binding Assay | % Inhibition of Control Specific Binding | % of Control Specific Binding | Reference Compound | $IC_{50}$ Ref (M) | Ki Ref (M) |
|---|---|---|---|---|---|
| A2A (h) (agonist radioligand) | 15 | 85.4 | NECA | 2.8E-08 | 2.3E-08 |
| alpha 1A (h) (antagonist radioligand) | 9 | 91.2 | WB 4101 | 4.1E-10 | 2.1E-10 |
| alpha 2A (h) (antagonist radioligand) | -9 | 108.9 | yohimbine | 6.8E-09 | 3.0E-09 |
| beta 1 (h) (agonist radioligand) | 4 | 96.2 | atenolol | 6.4E-07 | 3.6E-07 |
| beta 2 (h) (antagonist radioligand) | 3 | 97.4 | ICI 118551 | 3.5E-10 | 1.2E-10 |
| BZD (central) (agonist radioligand) | 0 | 100.1 | diazepam | 1.5E-08 | 1.3E-08 |
| CB1 (h) (agonist radioligand) | -8 | 108.3 | CP 55940 | 1.1E-09 | 9.6E-10 |
| CB2 (h) (agonist radioligand) | 11 | 89.1 | WIN 55212-2 | 1.7E-09 | 1.1E-09 |
| CCK1 (CCKA) (h) (agonist radioligand) | -3 | 103.3 | CCK-8s | 1.2E-10 | 9.1E-11 |
| D1 (h) (antagonist radioligand) | 2 | 97.9 | SCH 23390 | 4.5E-10 | 1.8E-10 |
| D2S (h) (agonist radioligand) | 13 | 86.8 | 7-OH-DPAT | 2.7E-09 | 1.1E-09 |
| ETA (h) (agonist radioligand) | -3 | 103.1 | endothelin-1 | 1.1E-10 | 5.4E-11 |
| NMDA (antagonist radioligand) | 6 | 94.2 | CGS 19755 | 1.9E-07 | 1.5E-07 |
| H1 (h) (antagonist radioligand) | -5 | 105.3 | pyrilamine | 2.2E-09 | 1.4E-09 |
| H2 (h) (antagonist radioligand) | -7 | 107.4 | cimetidine | 4.4E-07 | 4.2E-07 |
| MAO-A (antagonist radioligand) | 11 | 89.2 | clorgyline | 2.2E-09 | 1.3E-09 |
| M1 (h) (antagonist radioligand) | -8 | 107.8 | pirenzepine | 3.2E-08 | 2.7E-08 |
| M2 (h) (antagonist radioligand) | -31 | 130.7 | methoctramine | 3.1E-08 | 2.2E-08 |
| M3 (h) (antagonist radioligand) | 5 | 94.8 | 4-DAMP | 9.6E-10 | 6.9E-10 |
| N neuronal alpha 4beta 2 (h) (agonist radioligand) | -3 | 102.6 | nicotine | 6.0E-09 | 2.0E-09 |
| delta (DOP) (h) (agonist radioligand) | -6 | 106.5 | DPDPE | 3.7E-09 | 2.2E-09 |
| kappa (KOP) (agonist radioligand) | -2 | 102.0 | U 50488 | 8.8E-10 | 5.9E-10 |
| mu (MOP) (h) (agonist radioligand) | 6 | 93.6 | DAMGO | 1.2E-09 | 5.0E-10 |
| 5-HT1A (h) (agonist radioligand) | -3 | 103.4 | 8-OH-DPAT | 6.2E-10 | 3.1E-10 |
| 5-HT1B (antagonist radioligand) | 31 | 69.5 | serotonin | 1.8E-08 | 1.1E-08 |
| 5-HT2A (h) (agonist radioligand) | 9 | 90.8 | (±)DOI | 2.4E-10 | 1.8E-10 |
| 5-HT2B (h) (agonist radioligand) | 26 | 73.6 | (±)DOI | 6.8E-09 | 3.4E-09 |
| 5-HT3 (h) (antagonist radioligand) | -6 | 105.5 | MDL 72222 | 1.4E-08 | 9.4E-09 |
| GR (h) (agonist radioligand) | -2 | 102.1 | dexamethasone | 3.1E-09 | 1.6E-09 |
| AR (h) (agonist radioligand) | -4 | 104.4 | testosterone | 4.3E-09 | 1.9E-09 |
| V1a (h) (agonist radioligand) | 2 | 98.4 | [d(CH2)51,Tyr(Me)2]-AVP | 2.2E-09 | 1.4E-09 |
| Ca2+ channel (L, dihydropyridine site) (antagonist radioligand) | -12 | 111.7 | nitrendipine | 1.8E-10 | 1.1E-10 |
| Potassium Channel hERG (human)-[3H] Dofetilide | 0 | 100.0 | Terfenadine | 4.5E-08 | 3.1E-08 |
| KV channel (antagonist radioligand) | 2 | 98.3 | alpha -dendrotoxin | 1.2E-10 | 1.0E-10 |
| Na+ channel (site 2) (antagonist radioligand) | -7 | 106.6 | veratridine | 1.1E-05 | 9.6E-06 |
| norepinephrine transporter (h) (antagonist radioligand) | -16 | 115.9 | protriptyline | 3.8E-09 | 2.8E-09 |
| dopamine transporter (h) (antagonist radioligand) | 0 | 100.0 | BTCP | 1.2E-08 | 6.4E-09 |

| | | | | | |
|---|---|---|---|---|---|
| 5-HT transporter (h) (antagonist radioligand) | -3 | 103.1 | imipramine | 3.3E-09 | 1.5E-09 |

| **Enzyme Assay** | **% Inhibition of Control Values** | **% of Control Values** | **Reference Compound** | **$IC_{50}$ Ref (M)** |
|---|---|---|---|---|
| COX1(h) | 9 | 90.9 | Diclofenac | 1.1E-08 |
| COX2(h) | 1 | 98.7 | NS398 | 1.1E-07 |
| PDE3A (h) | -21 | 121.2 | milrinone | 3.2E-07 |
| PDE4D2 (h) | 37 | 63.4 | Ro 20-1724 | 2.3E-07 |
| Lck kinase (h) | 8 | 92.2 | staurosporine | 2.8E-08 |
| acetylcholinesterase (h) | 1 | 99.0 | galanthamine | 8.6E-07 |

**Table S5: Dundee kinase panel results for compound 8 at 10 μM**

| Kinase | % activity remaining | Kinase | % activity remaining |
|---|---|---|---|
| MKK1 | 91 | NEK2a | 111 |
| MKK2 | 86 | NEK6 | 92 |
| MKK6 | 99 | IKKb | 71 |
| ERK1 | 98 | IKKe | 92 |
| ERK2 | 87 | TBK1 | 95 |
| ERK5 | 93 | PIM1 | 92 |
| JNK1 | 108 | PIM2 | 105 |
| JNK2 | 99 | PIM3 | 91 |
| JNK3 | 101 | SRPK1 | 96 |
| p38a MAPK | 111 | EF2K | 95 |
| p38b MAPK | 108 | EIF2AK3 | 104 |
| p38g MAPK | 99 | HIPK1 | 108 |
| p38d MAPK | 106 | HIPK2 | 89 |
| ERK8 | 74 | HIPK3 | 108 |
| RSK1 | 92 | CLK2 | 73 |
| RSK2 | 92 | PAK2 | 98 |
| PDK1 | 91 | PAK4 | 89 |
| PKBa | 98 | PAK5 | 105 |
| PKBb | 107 | PAK6 | 88 |
| SGK1 | 101 | MST2 | 84 |
| S6K1 | 89 | MST3 | 94 |
| PKA | 101 | MST4 | 95 |
| ROCK 2 | 90 | GCK | 111 |
| PRK2 | 112 | MAP4K3 | 96 |
| PKCa | 93 | MAP4K5 | 110 |
| PKCγ | 95 | MINK1 | 109 |
| PKCz | 108 | MEKK1 | 85 |
| PKD1 | 87 | MLK1 | 111 |
| STK33 | 91 | MLK3 | 96 |
| MSK1 | 90 | TESK1 | 83 |
| MNK1 | 86 | TAO1 | 85 |
| MNK2 | 66 | ASK1 | 98 |
| MAPKAP-K2 | 103 | TAK1 | 97 |
| MAPKAP-K3 | 98 | IRAK1 | 110 |
| PRAK | 98 | IRAK4 | 92 |
| CAMKKb | 98 | RIPK2 | 105 |
| CAMK1 | 109 | OSR1 | 105 |
| SmMLCK | 83 | TTK | 93 |
| PHK | 103 | MPSK1 | 112 |
| DAPK1 | 91 | WNK1 | 91 |
| CHK1 | 96 | ULK1 | 99 |
| CHK2 | 98 | ULK2 | 113 |
| GSK3b | 103 | TGFBR1 | 93 |
| CDK2-Cyclin A | 97 | Src | 89 |
| CDK9-Cyclin T1 | 89 | Lck | 84 |
| PLK1 | 93 | CSK | 89 |
| Aurora A | 97 | YES1 | 104 |
| Aurora B | 92 | ABL | 101 |
| TLK1 | 105 | BTK | 101 |
| LKB1 | 88 | JAK3 | 101 |
| AMPK (hum) | 94 | SYK | 93 |
| MARK1 | 96 | ZAP70 | 103 |
| MARK2 | 91 | TIE2 | 102 |
| MARK3 | 87 | BRK | 89 |
| MARK4 | 98 | EPH-A2 | 105 |
| BRSK1 | 93 | EPH-A4 | 108 |
| BRSK2 | 94 | EPH-B1 | 123 |

| | | | |
|---|---|---|---|
| **MELK** | 89 | **EPH-B2** | 108 |
| **NUAK1** | 91 | **EPH-B3** | 99 |
| **SIK2** | 91 | **EPH-B4** | 99 |
| **SIK3** | 87 | **FGF-R1** | 104 |
| **TSSK1** | 97 | **HER4** | 89 |
| **CK1γ2** | 99 | **IGF-1R** | 111 |
| **CK1δ** | 99 | **IR** | 86 |
| **CK2** | 94 | **IRR** | 91 |
| **TTBK1** | 106 | **TrkA** | 110 |
| **TTBK2** | 111 | **DDR2** | 116 |
| **DYRK1A** | 70 | **VEG-FR** | 85 |
| **DYRK2** | 58 | **PDGFRA** | 91 |
| **DYRK3** | 63 | **PINK** | 105 |

**Figure S2: Compound 12 electron density**. Initial difference density (contoured at 3 sigma) for compound **12** with the final coordinates overlaid showing unambiguous assignation of the absolute stereochemistry for this compound. (PDB9r32)

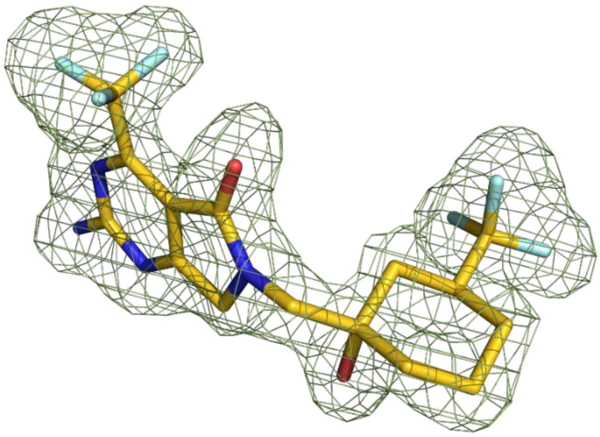

**Figure S3 and Table S6: Oral mouse pharmacokinetics for compound 10 and 12 following a 10 mg/kg single oral administration to the female Balb/c mouse (n=3). Mean total blood concentration (blue), mean free blood concentration (green) and *P. falciparum* (3D7) asexual blood stage $EC_{90}$ (red dashed line).**

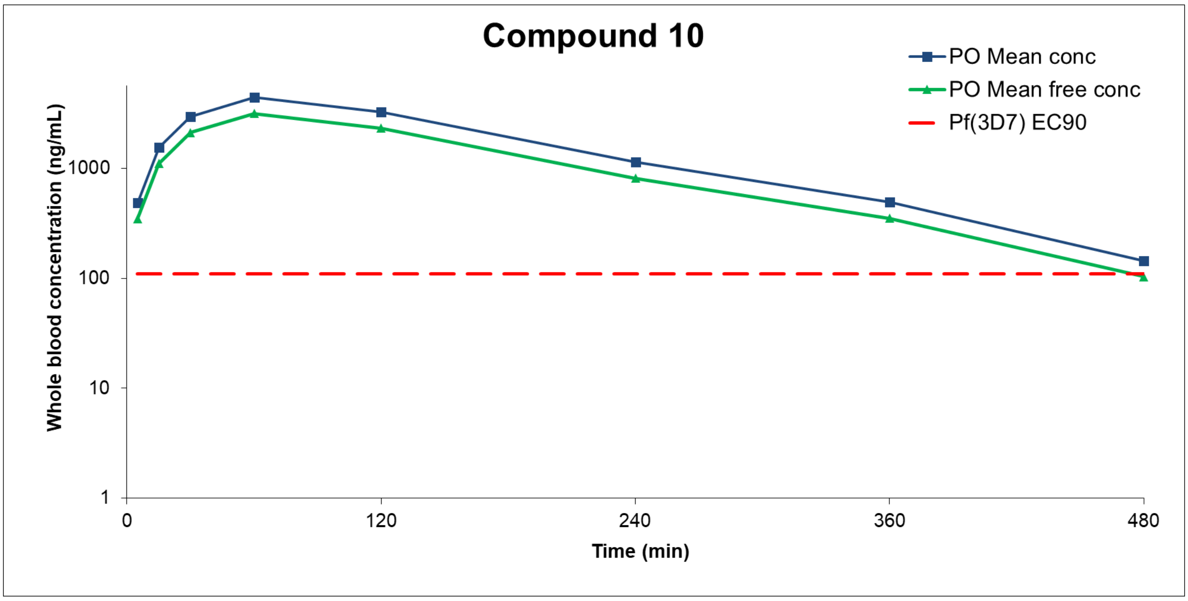


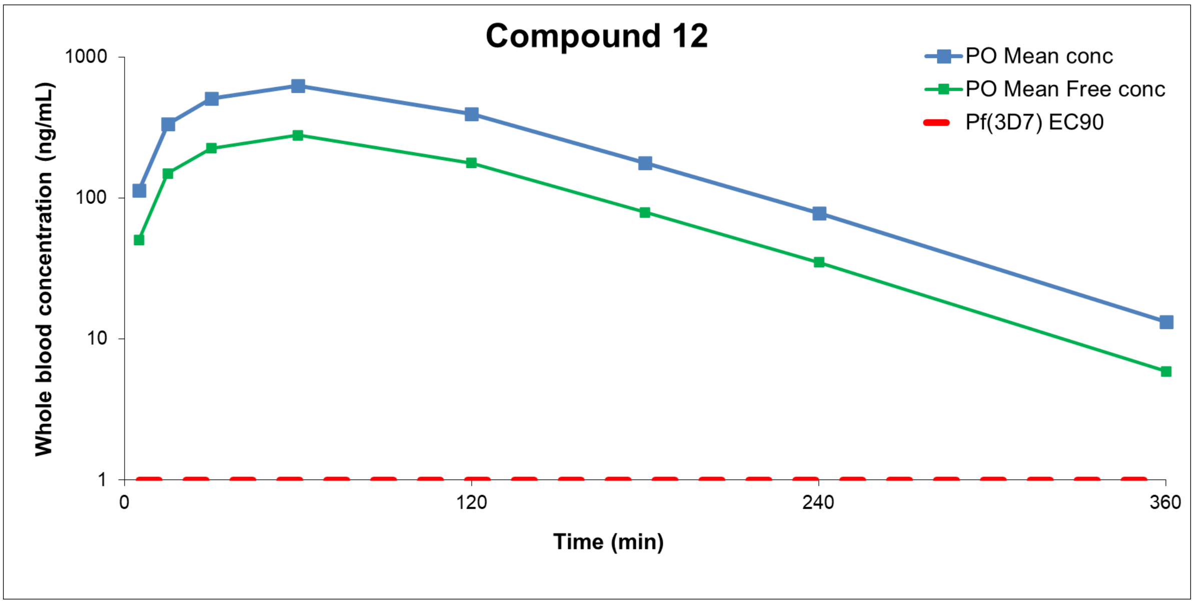


| **Compound** | **10** | **12** |
|---|---|---|
| Route | PO | PO |
| Dose (mg/kg) | 10 | 10 |
| $C_{max}$ (ng/mL) | 4397 | 628 |
| $T_{max}$ (h) | 1.6 | 0.9 |
| $AUC_{0\text{-}8h}$ (ng-min/mL) | 781845 | 87015 |
| Mouse PPB Fu | 0.715 | 0.446 |
| *Pf*(3D7) $EC_{90}$ (ng/mL) | 110 | 1 |

**Figure S4: Compounds included to generate the FMO-DFTB *Pf*KRS1 pIC50 predictive model**

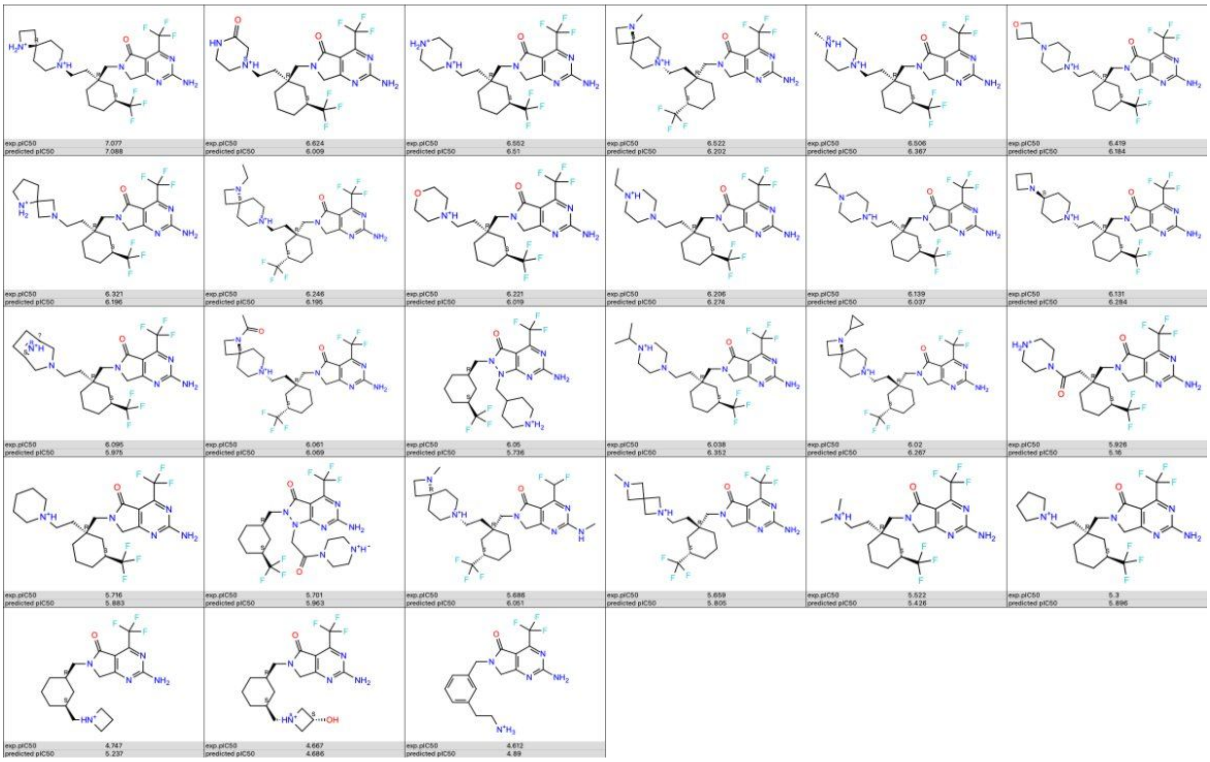

**Table S7: Summary of resistant selection results for compound 11**

| Parental Line | Parasite inoculum | Day parasites recrudesced | $IC_{50}$ fold change | Gene ID | Gene Name | Codon change | Amino acid change |
|---|---|---|---|---|---|---|---|
| Dd2B2 | $1x10^8$ | Day 16: 1/3 Flasks | 300 | PF3D7_1350100 | Lysyl-tRNA synthetase | tCg/tTg | S344L |

**Table S8: Summary of resistant selection results for compound 30**

| Sample | N | $IC_{50}$ ± SEM (nM) | $IC_{90}$ ± SEM (nM) | $IC_{90}/IC_{50}$ ± SEM (nM) | $IC_{50}$ (FC) | $IC_{90}$ (FC) | $IC_{90}/IC_{50}$ (FC) |
|---|---|---|---|---|---|---|---|
| Dd2-B2 | 5 | 1.5 ± 0.1 | 3.7 ± 0.9 | 2.6 ± 0.4 | 1.0 | 1.0 | 1.0 |
| Dd2-B2W4 | 5 | 37 ± 0.6 | 54 ± 1.3 | 1.5 ± 0.05 | 25 | 15 | 1.7 |

**Table S9: List of mutations from whole-genome sequencing of compound 30 resistant clone**

| Sample | Gene ID | Gene name | Amino acid change | Codon change | Effect | W1.alt_AB |
|---|---|---|---|---|---|---|
| Dd2-B2W4 | PF3D7_0827000 | ATP-dependent RNA helicase DBP10, putative | S1281* | tCa/tAa | Stop gained | 0.96 |
| Dd2-B2W4 | PF3D7_1350100 | Lysyl-tRNA synthetase | F342Y | tTt/tAt | Non synonymous coding | 1 |

# LCMS for final compounds

## Compound 2

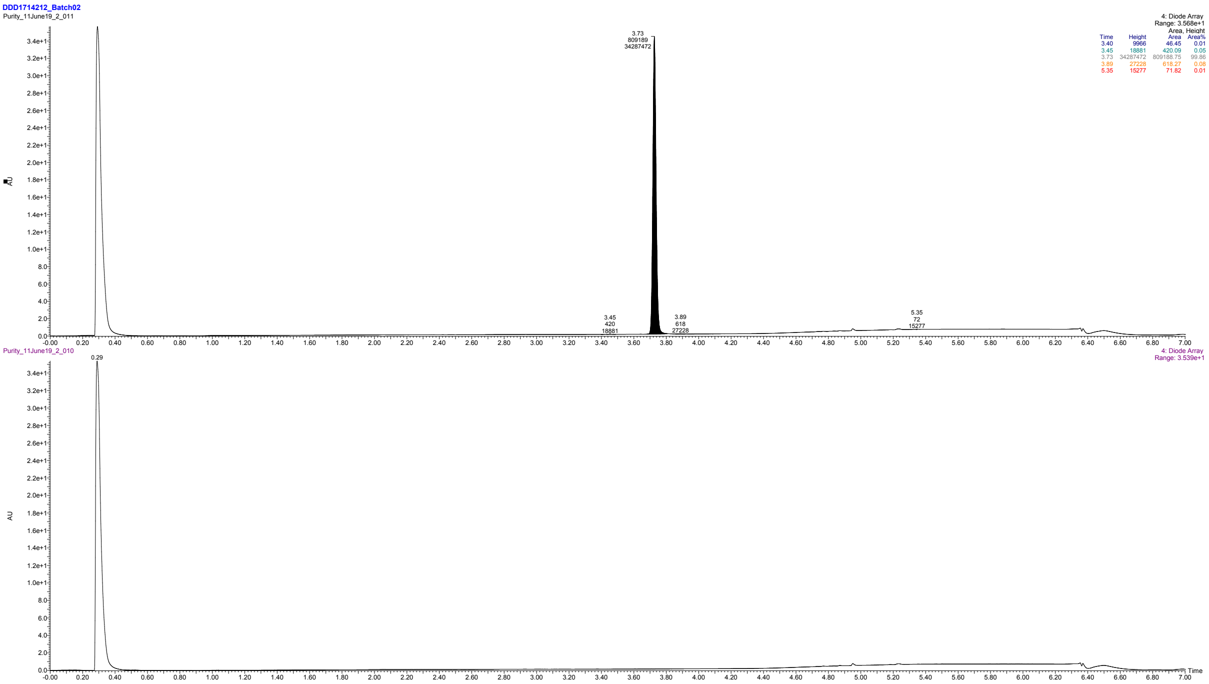


## b)

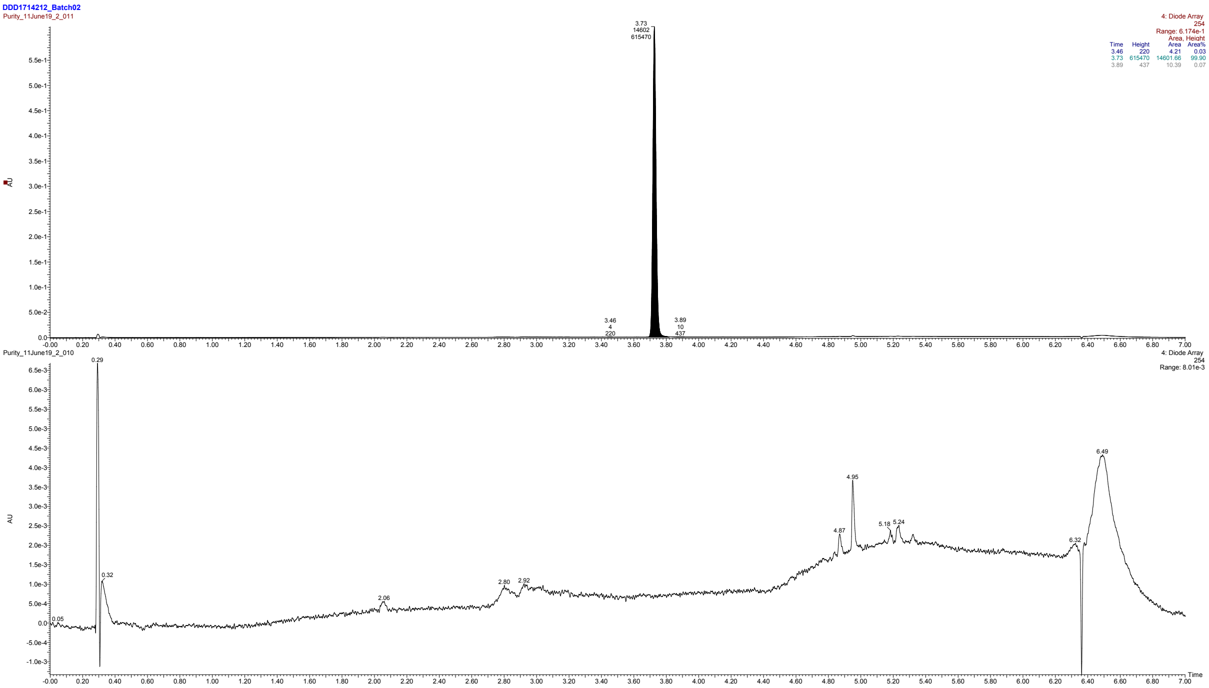

**c)**

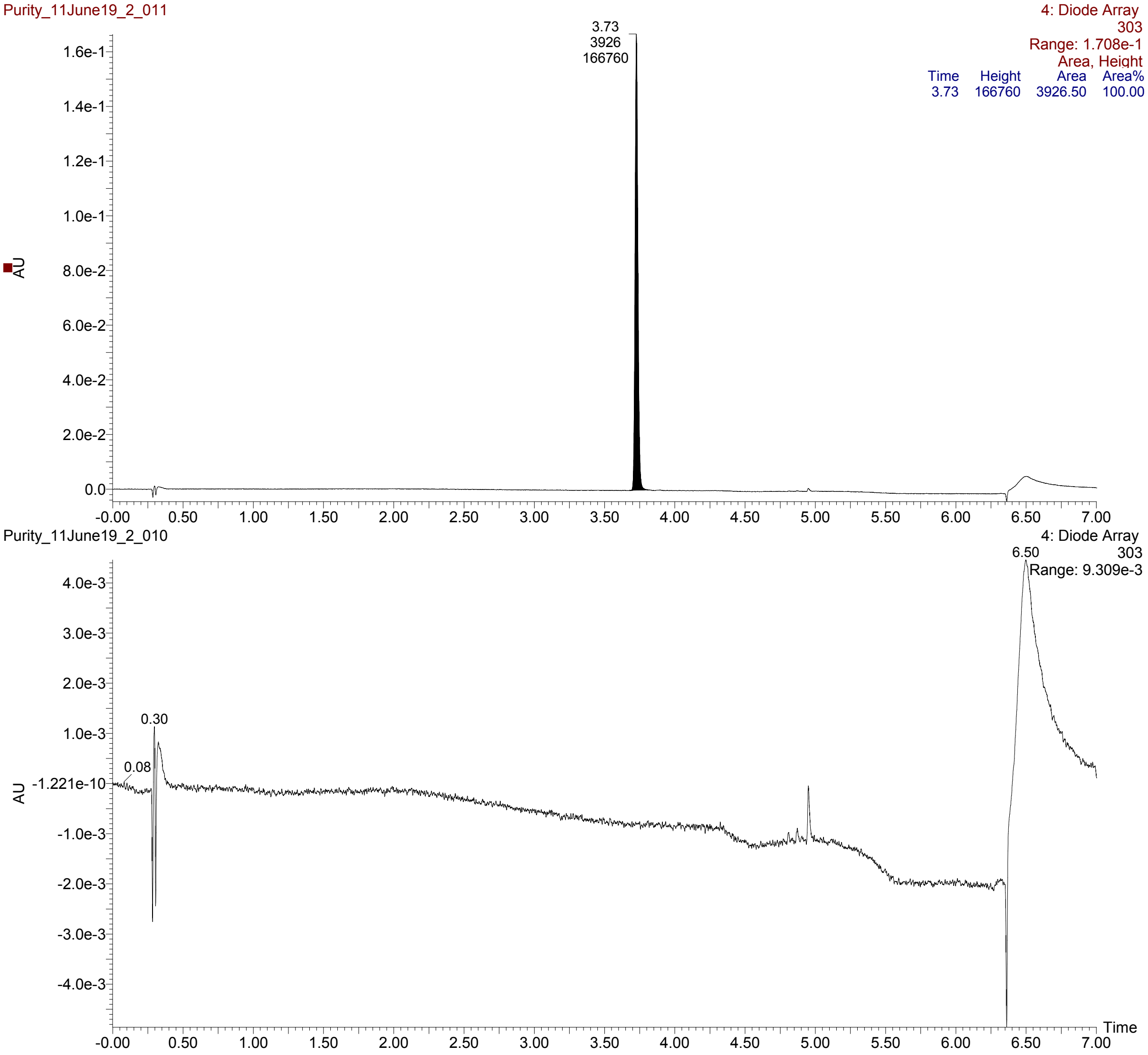

DDD1714212_Batch02
Purity_11June19_2_011
4: Diode Array
303
Range: 1.708e-1
Area, Height
Time Height Area Area%
3.73 166760 3926.50 100.00
3.73
3926
166760
AU
Purity_11June19_2_010
4: Diode Array
303
Range: 9.309e-3
6.50
0.30
0.08
AU
Time


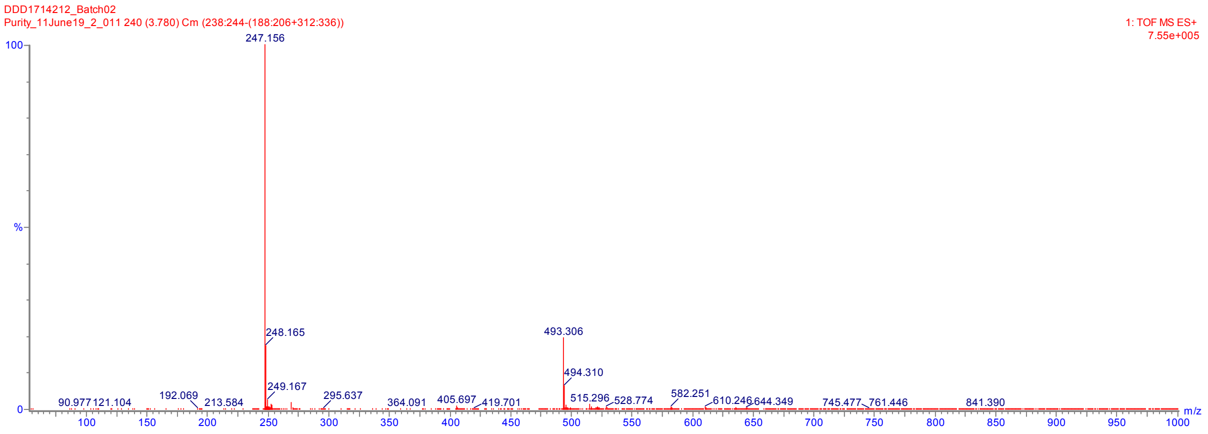

DDD1714212_Batch02
Purity_11June19_2_011 240 (3.780) Cm (238:244-(188:206+312:336))
1: TOF MS ES+
7.55e+005
247.156
248.165
249.167
493.306
494.310
%
m/z

## Compound 3

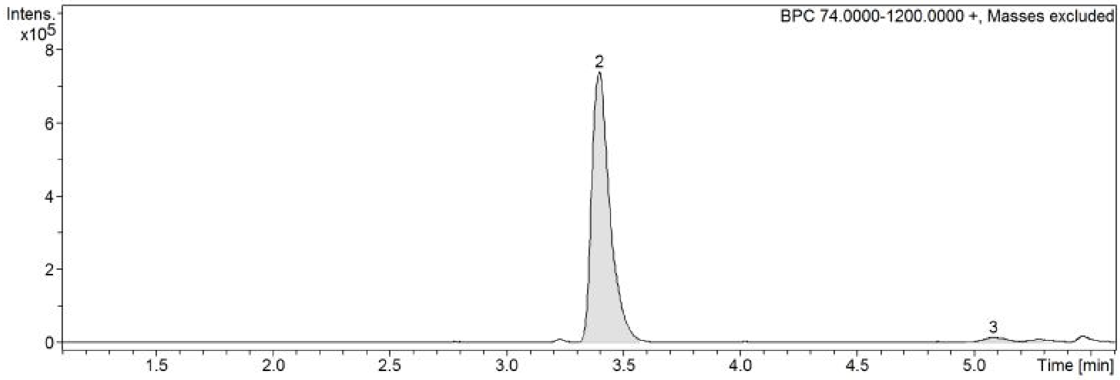


Sample Description

Method 1-microtof-2 Identify Compounds LCMS Pos 5-95.m

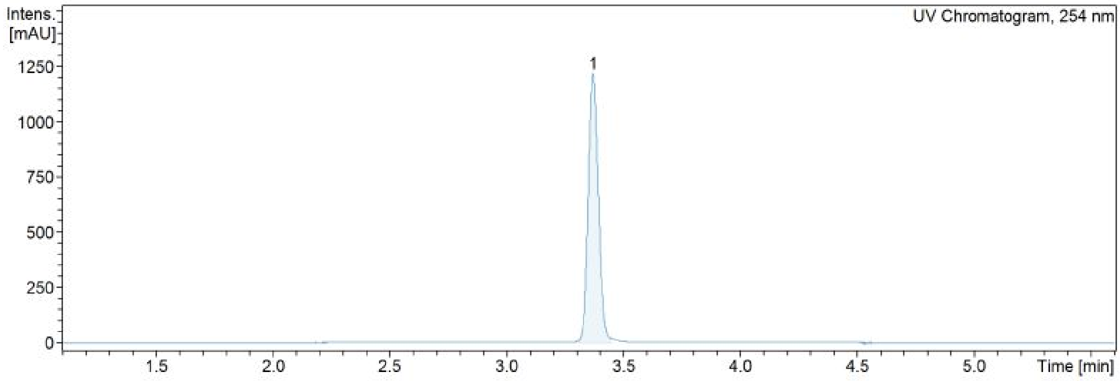


**Cmpd 1, 3.4 min**

Undefined context.
Component not placed in a spectrum iterator.

**Cmpd 2, 3.4 min**

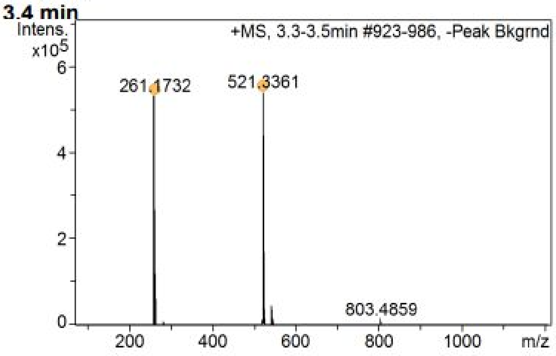

**Compound 4**

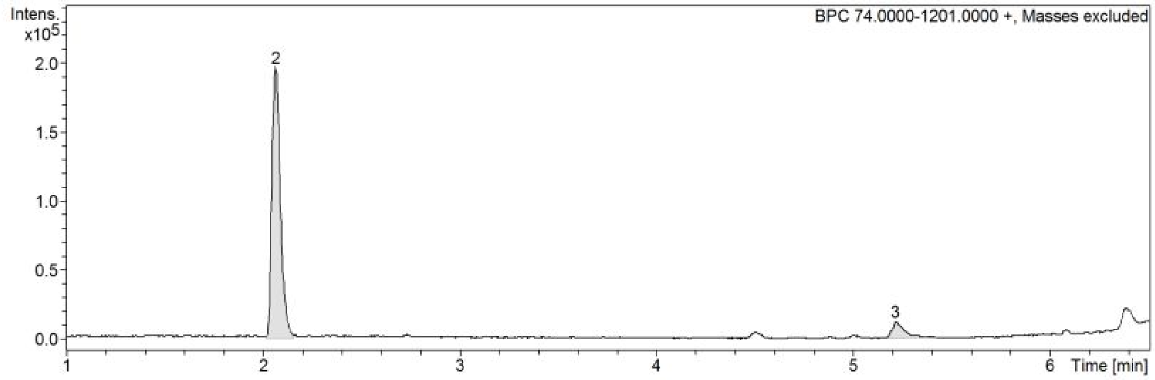


Sample Description

Method 1-microtof-2 Identify Compounds LCMS Pos 5-95.m

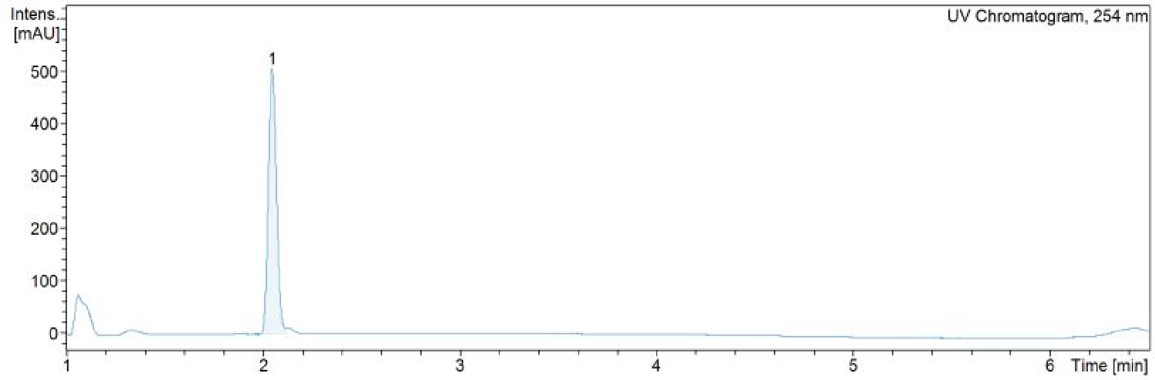


**Cmpd 1, 2.0 min**

Undefined context.
Component not placed in a spectrum iterator.

**Cmpd 2, 2.1 min**

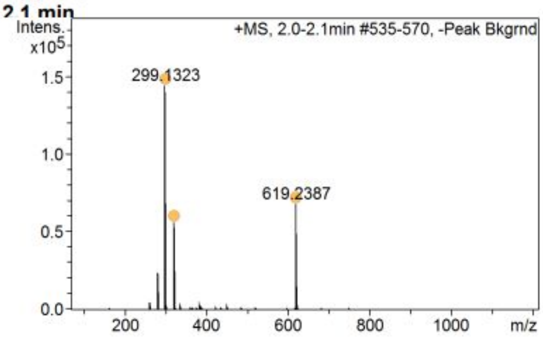

# Compound 5

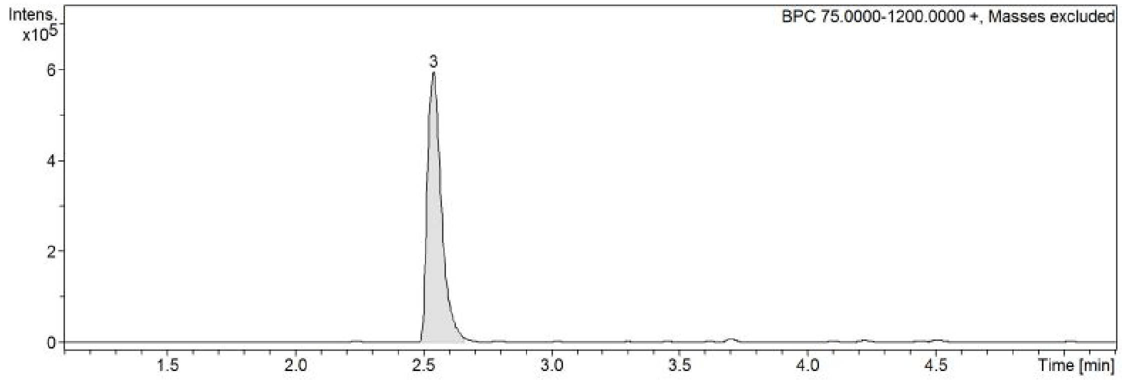


Sample Description

Method 1-microtof-2 Identify Compounds LCMS Pos 5-95.m

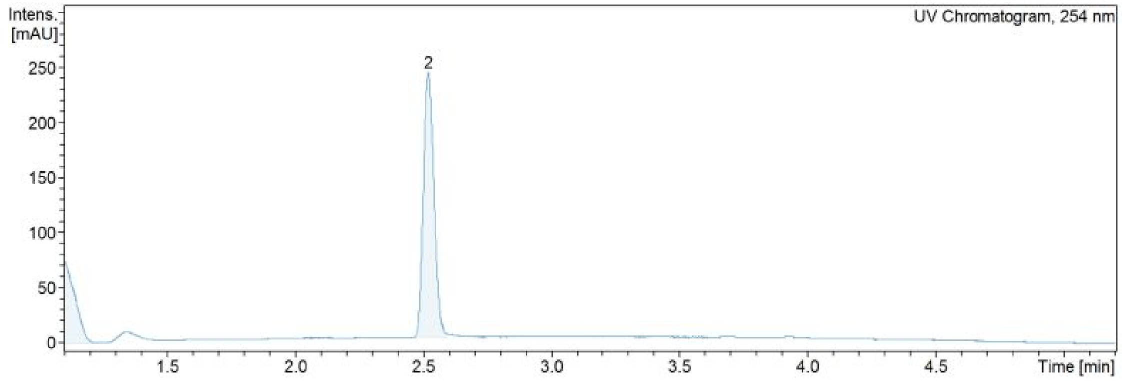


**Cmpd 1, 1.1 min**

Undefined context.
Component not placed in a spectrum iterator.

**Cmpd 2, 2.5 min**

Undefined context.
Component not placed in a spectrum iterator.

**Cmpd 3, 2.5 min**

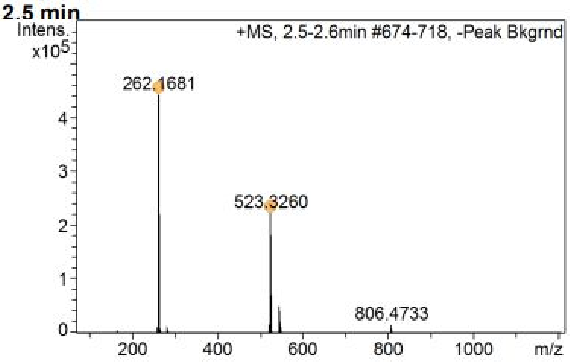

**Compound 6**

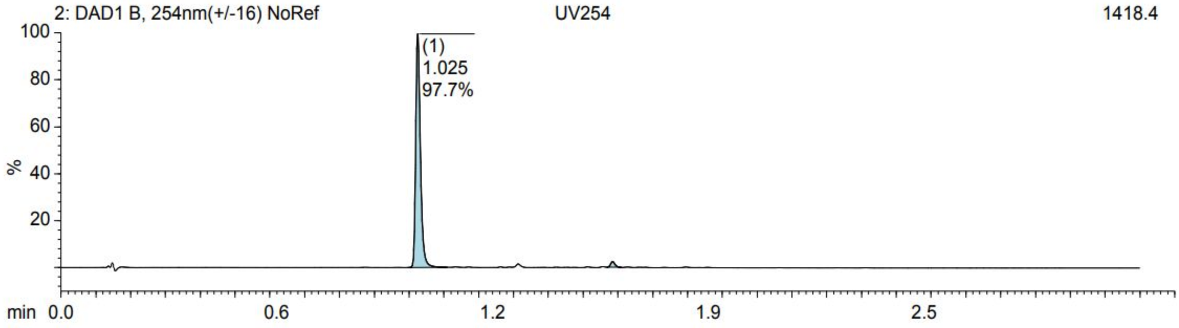

2: DAD1 B, 254nm(+/-16) NoRef
UV254
1418.4
(1)
1.025
97.7%
%
min


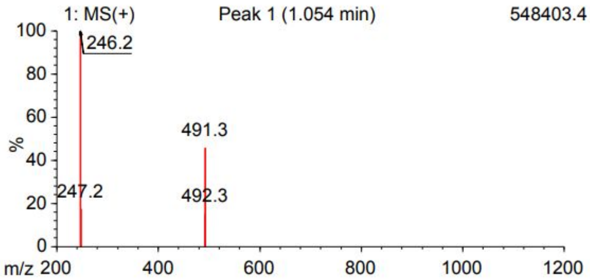

1: MS(+)
Peak 1 (1.054 min)
548403.4
246.2
491.3
247.2
492.3
%
m/z


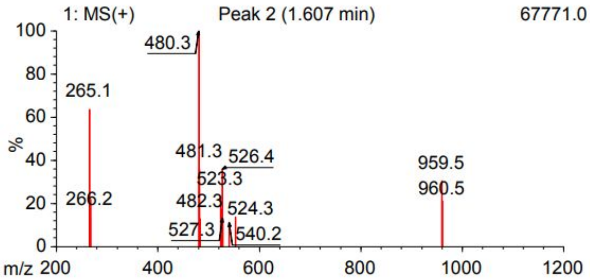

1: MS(+)
Peak 2 (1.607 min)
67771.0
480.3
265.1
481.3
526.4
523.3
959.5
960.5
266.2
482.3
524.3
527.3
540.2
%
m/z


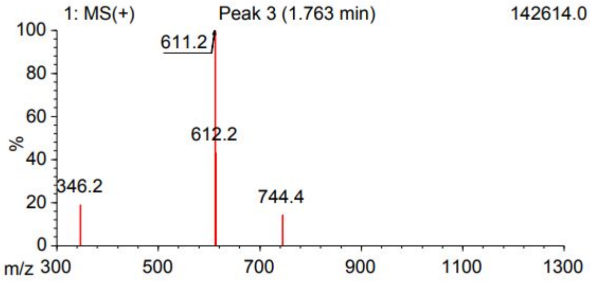

1: MS(+)
Peak 3 (1.763 min)
142614.0
611.2
612.2
346.2
744.4
%
m/z

## Compound 7

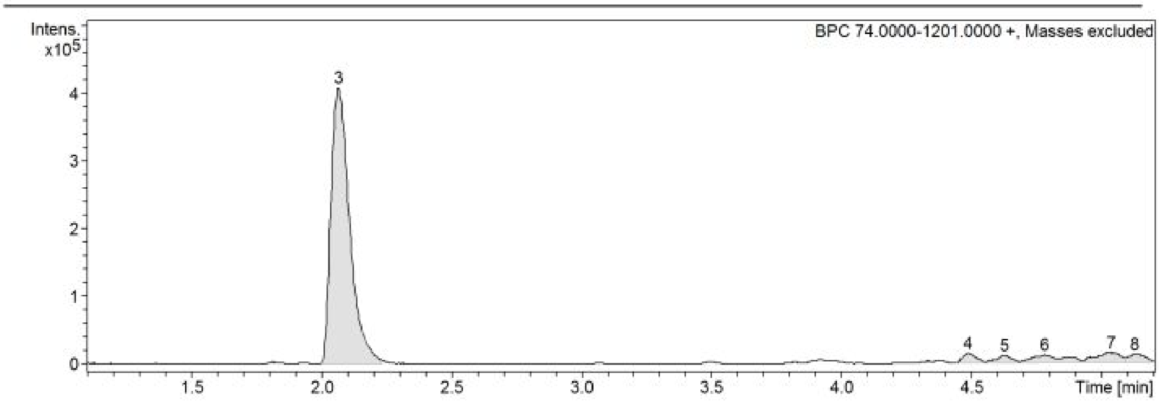


Sample Description

Method 1-microtof-2 Identify
Compounds LCMS Pos 5-95.m

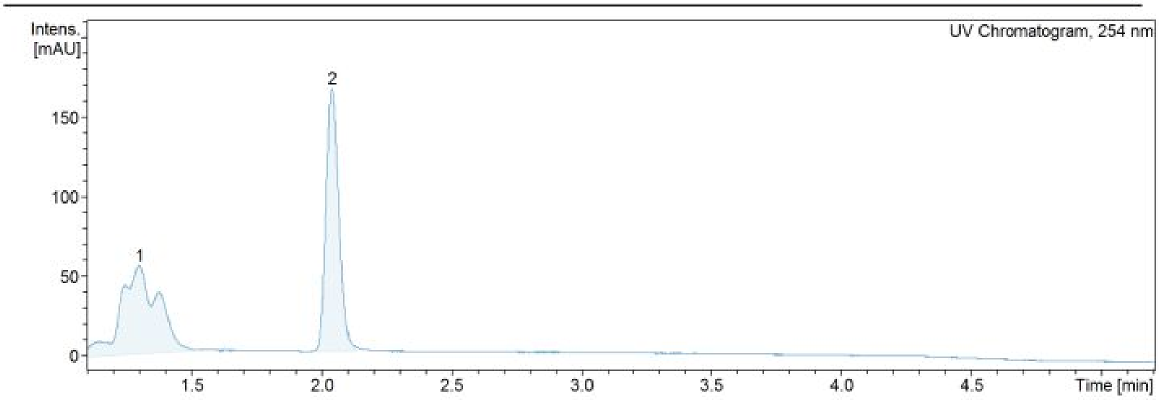


**Cmpd 3,**
**2.1 min**

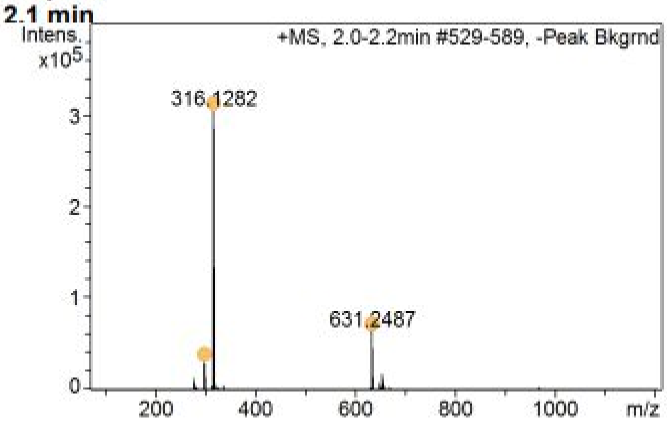

**Compound 8**

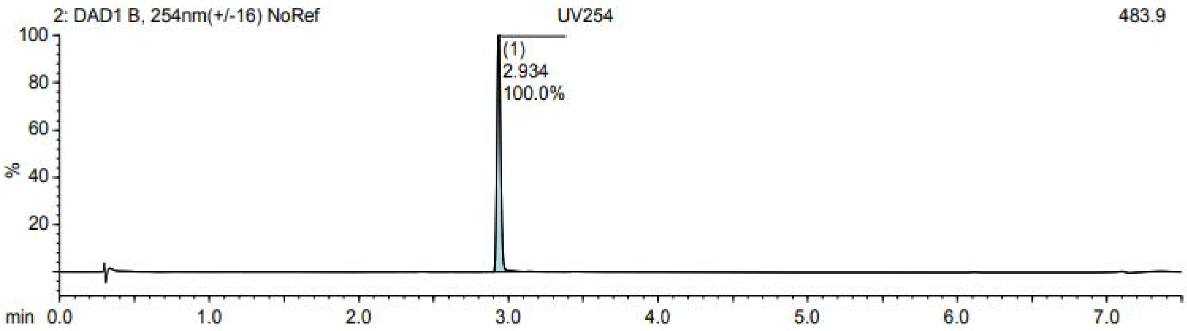
2: DAD1 B, 254nm(+/-16) NoRef
UV254
483.9
(1)
2.934
100.0%
%
min


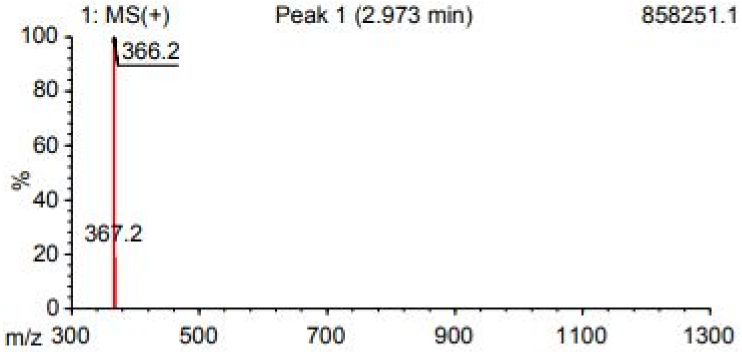
1: MS(+)
Peak 1 (2.973 min)
858251.1
366.2
367.2
%
m/z

## Compound 9

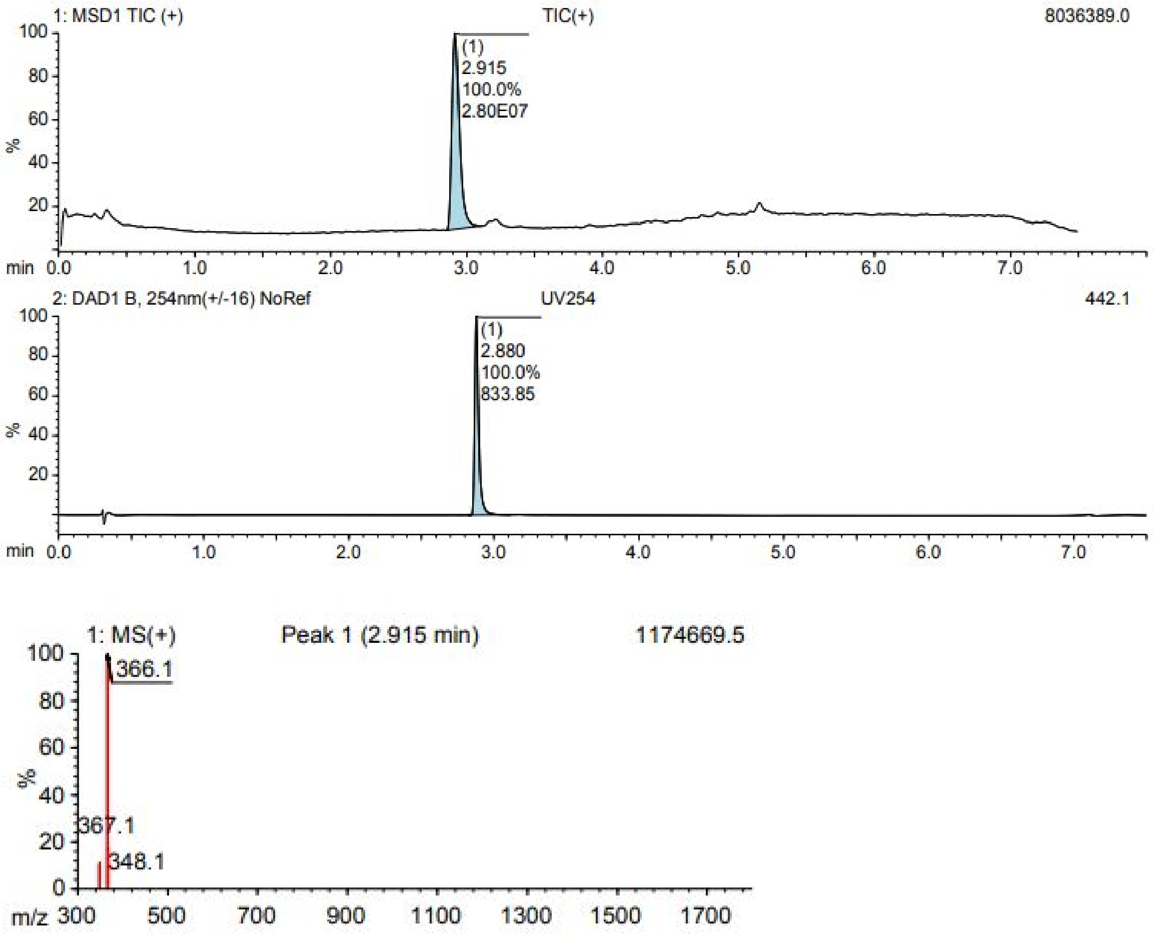
1: MSD1 TIC (+)
TIC(+)
8036389.0
(1)
2.915
100.0%
2.80E07
2: DAD1 B, 254nm(+/-16) NoRef
UV254
442.1
(1)
2.880
100.0%
833.85
1: MS(+)
Peak 1 (2.915 min)
1174669.5
366.1
367.1
348.1

**Compound 10**

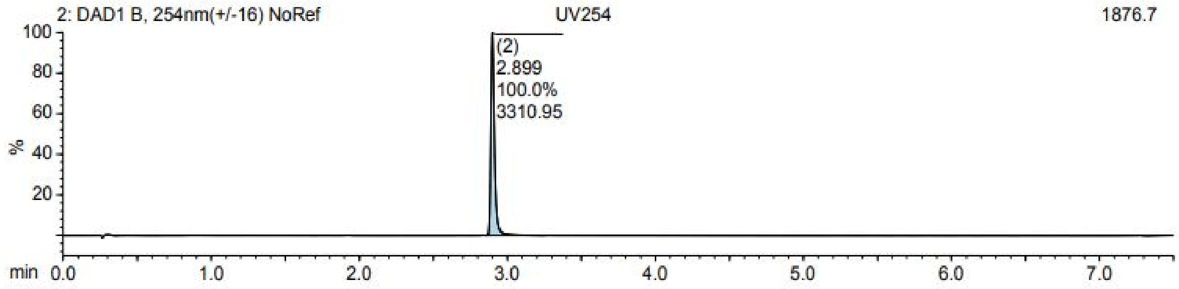

2: DAD1 B, 254nm(+/-16) NoRef
UV254
1876.7
(2)
2.899
100.0%
3310.95
%
min


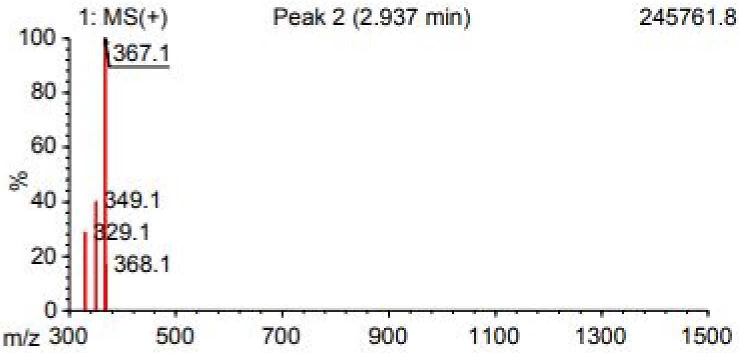

1: MS(+)
Peak 2 (2.937 min)
245761.8
367.1
349.1
329.1
368.1
%
m/z

**Compound 11**

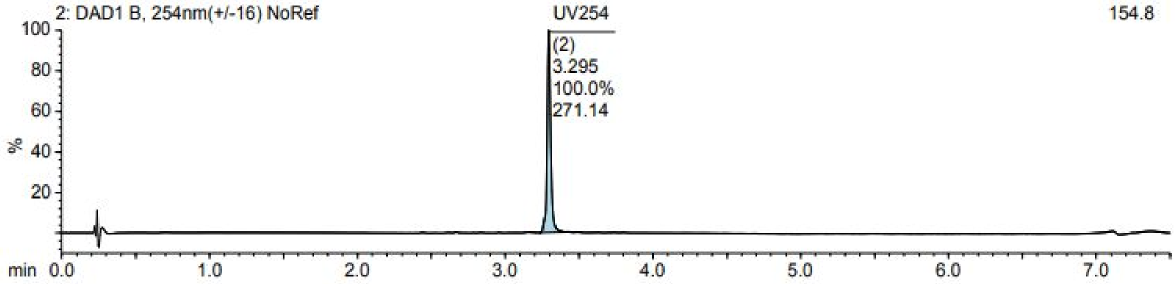

2: DAD1 B, 254nm(+/-16) NoRef
UV254
154.8
(2)
3.295
100.0%
271.14
%
min
0.0
1.0
2.0
3.0
4.0
5.0
6.0
7.0


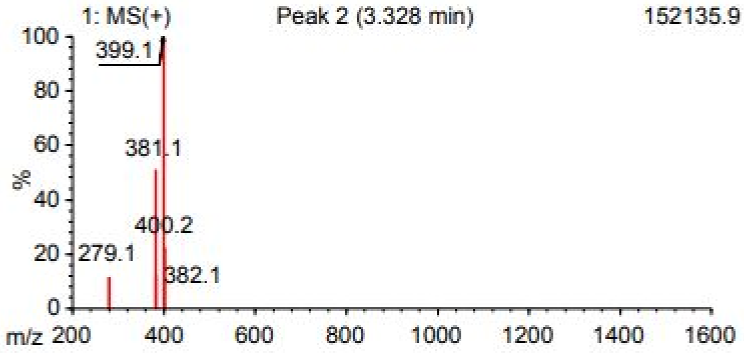

1: MS(+)
Peak 2 (3.328 min)
152135.9
399.1
381.1
400.2
279.1
382.1
%
m/z
200
400
600
800
1000
1200
1400
1600

**Compound 12**

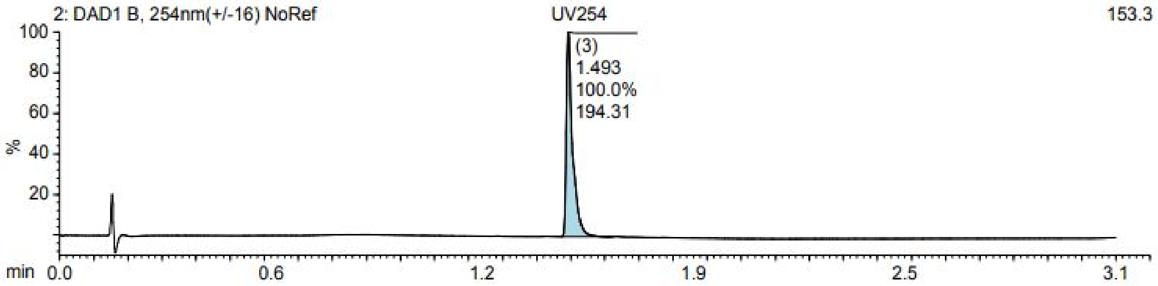
2: DAD1 B, 254nm(+/-16) NoRef
UV254
153.3
(3)
1.493
100.0%
194.31
%
min 0.0 0.6 1.2 1.9 2.5 3.1
100 80 60 40 20

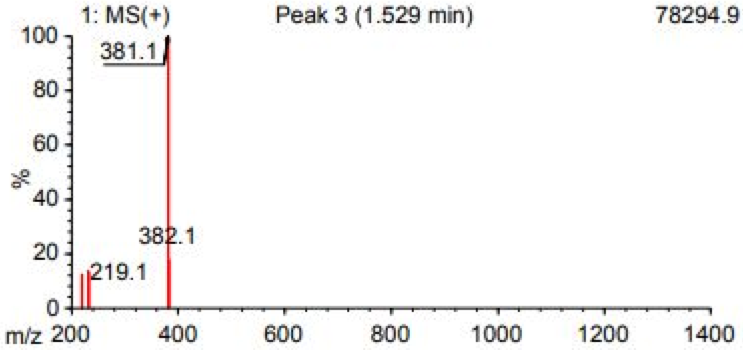
1: MS(+)
Peak 3 (1.529 min)
78294.9
381.1
382.1
219.1
%
m/z 200 400 600 800 1000 1200 1400
100 80 60 40 20 0

## Compound 13

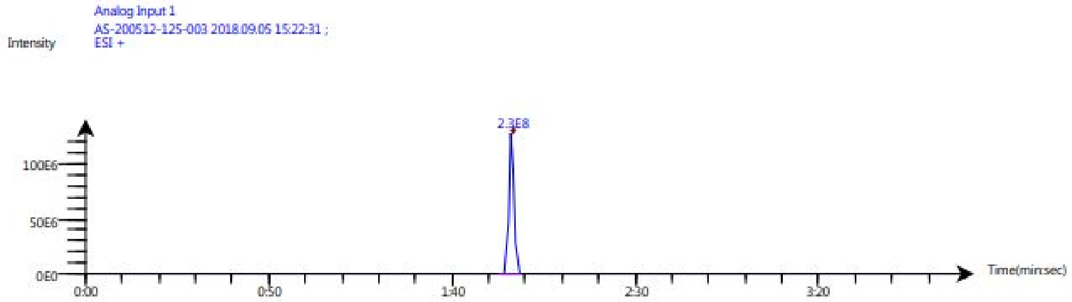


| Time (Peak Maximum M:S/Minutes) | Maximum Intensity (c/s) | Time (Peak Centroid M:S/Minutes) | Peak Area | % Peak Area | Peak Resolution | Label |
|---|---|---|---|---|---|---|
| 1:56.3 | 1.3E8 | 1:56.6 | 2.3E8 | 100.0 | 1.7 | |

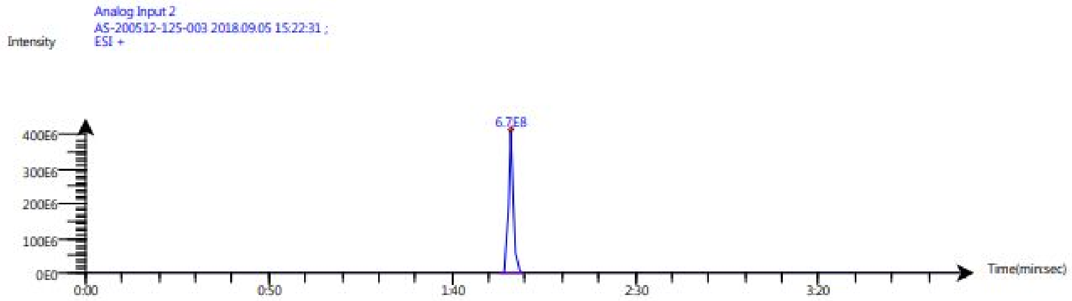


| Time (Peak Maximum M:S/Minutes) | Maximum Intensity (c/s) | Time (Peak Centroid M:S/Minutes) | Peak Area | % Peak Area | Peak Resolution | Label |
|---|---|---|---|---|---|---|
| 1:56.3 | 4.1E8 | 1:56.3 | 6.7E8 | 100.0 | 1.5 | |

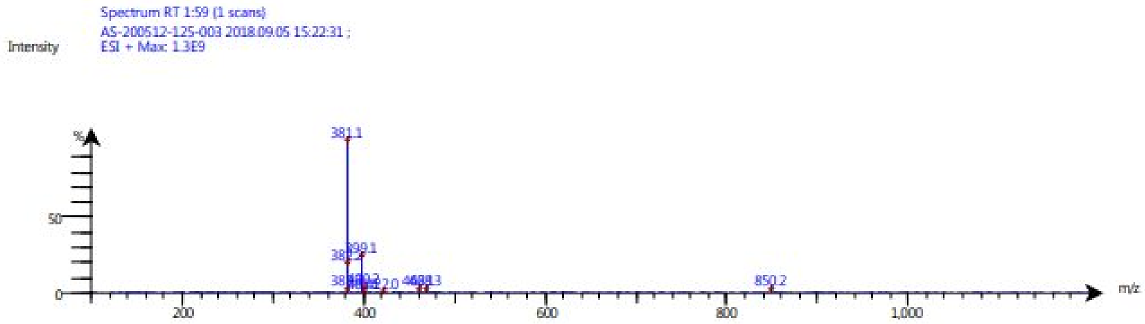

## Compound 14

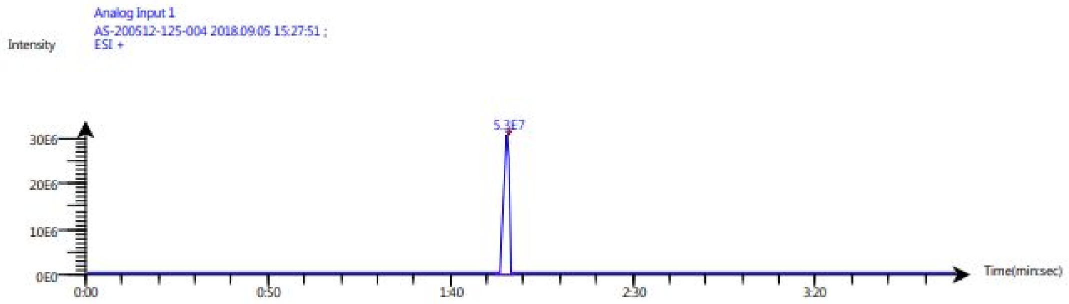


| Time (Peak Maximum M:S/Minutes) | Maximum Intensity (c/s) | Time (Peak Centroid M:S/Minutes) | Peak Area | % Peak Area | Peak Resolution | Label |
|---|---|---|---|---|---|---|
| 1:55.5 | 3.1E7 | 1:55.8 | 5.3E7 | 100.0 | 1.7 | |

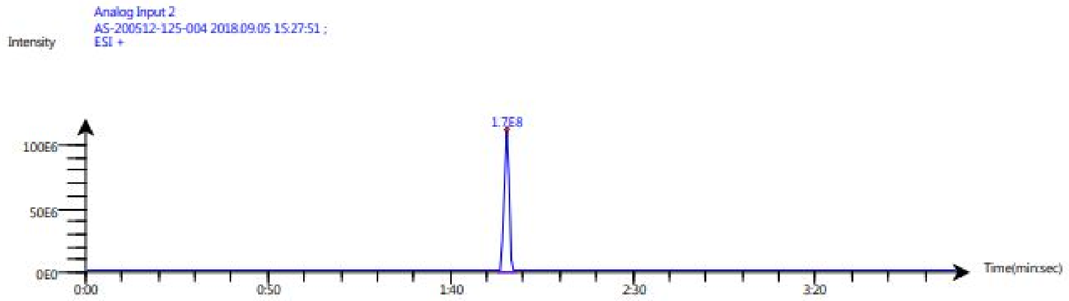


| Time (Peak Maximum M:S/Minutes) | Maximum Intensity (c/s) | Time (Peak Centroid M:S/Minutes) | Peak Area | % Peak Area | Peak Resolution | Label |
|---|---|---|---|---|---|---|
| 1:55.5 | 1.1E8 | 1:55.7 | 1.7E8 | 100.0 | 1.5 | |

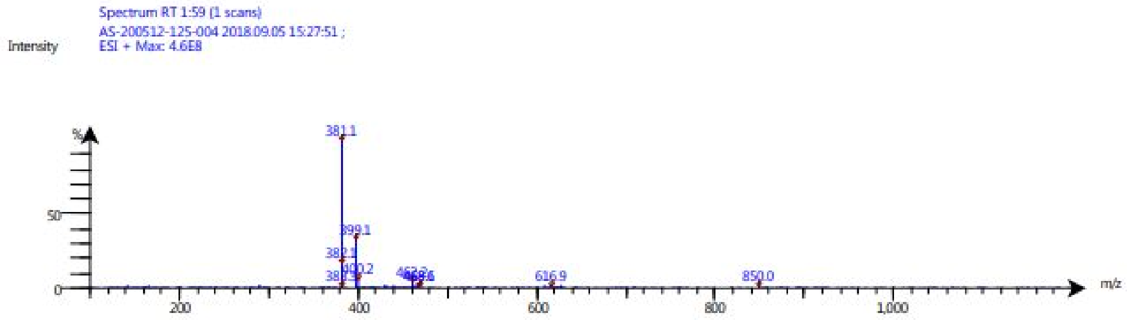

**Compound 15**

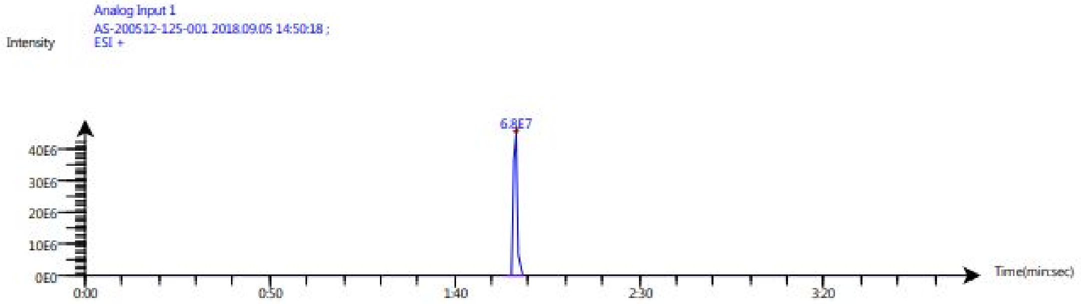


| Time (Peak Maximum M:S/Minutes) | Maximum Intensity (c/s) | Time (Peak Centroid M:S/Minutes) | Peak Area | % Peak Area | Peak Resolution | Label |
|---|---|---|---|---|---|---|
| 1:57.1 | 4.5E7 | 1:56.8 | 6.8E7 | 100.0 | 1.5 | |

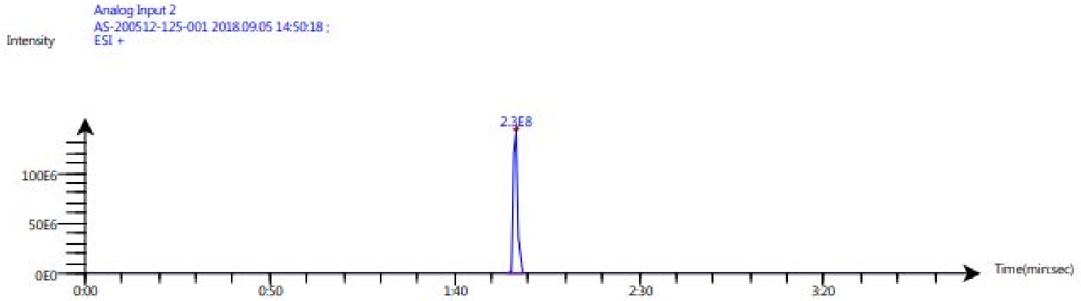


| Time (Peak Maximum M:S/Minutes) | Maximum Intensity (c/s) | Time (Peak Centroid M:S/Minutes) | Peak Area | % Peak Area | Peak Resolution | Label |
|---|---|---|---|---|---|---|
| 1:57.1 | 1.4E8 | 1:56.8 | 2.3E8 | 100.0 | 1.6 | |

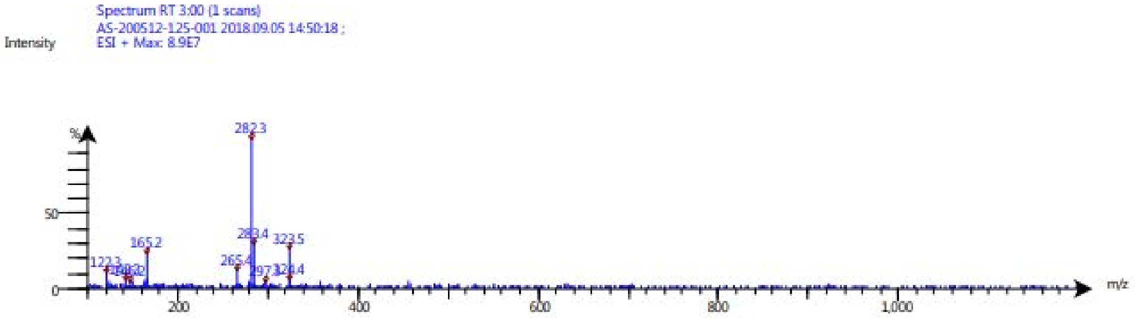

**Compound 16**

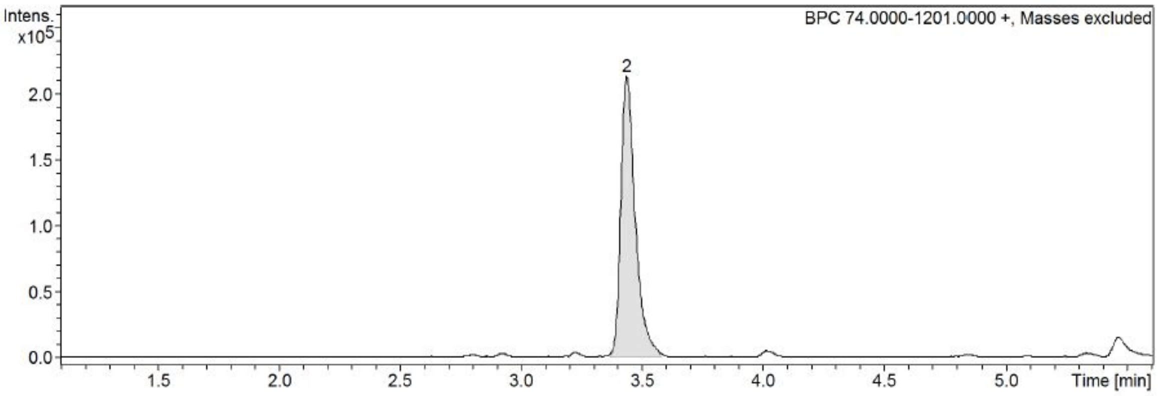


Cmpd 1, 3.4 min

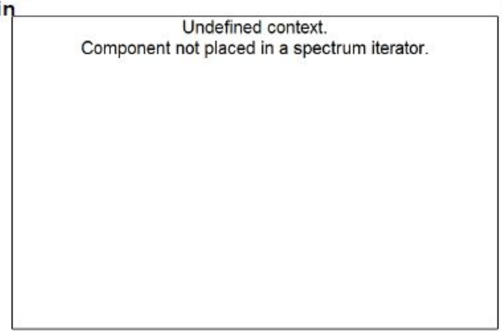


Cmpd 2, 3.4 min

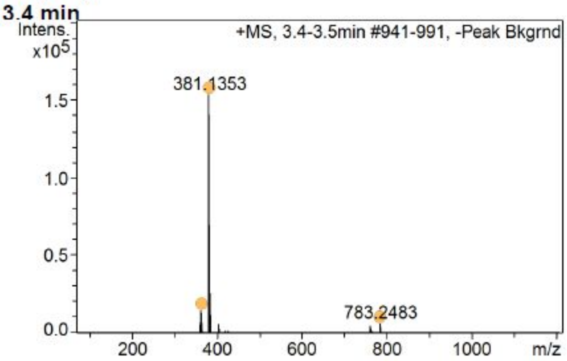

**Compound 17**

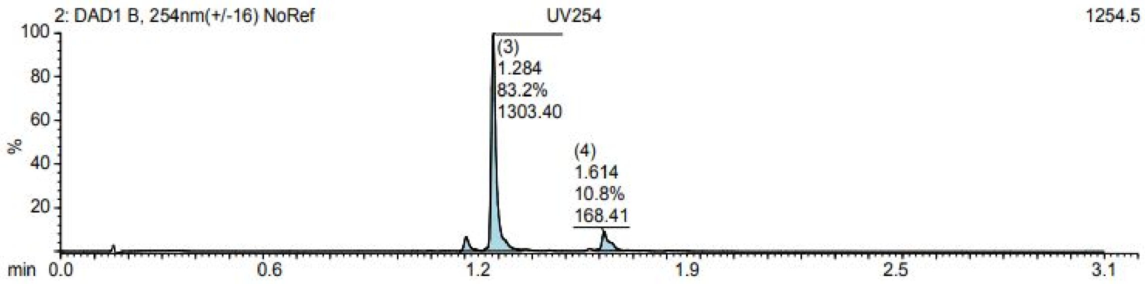
2: DAD1 B, 254nm(+/-16) NoRef
UV254
1254.5
(3)
1.284
83.2%
1303.40
(4)
1.614
10.8%
168.41
%
min
0.0
0.6
1.2
1.9
2.5
3.1
100
80
60
40
20

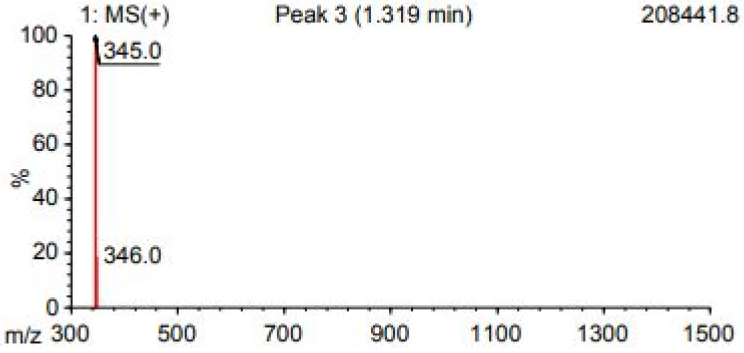
1: MS(+)
Peak 3 (1.319 min)
208441.8
345.0
346.0
%
m/z
300
500
700
900
1100
1300
1500
100
80
60
40
20
0

**Compound 18**

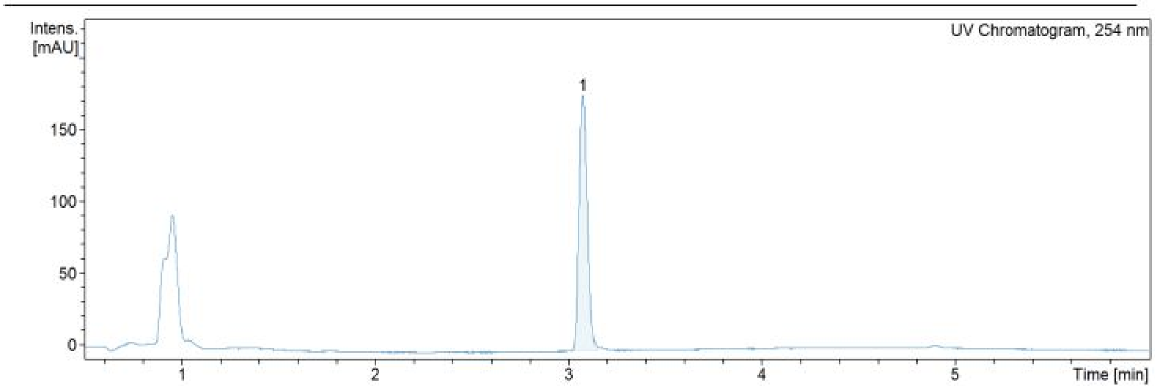


**Cmpd 2, 3.1 min**

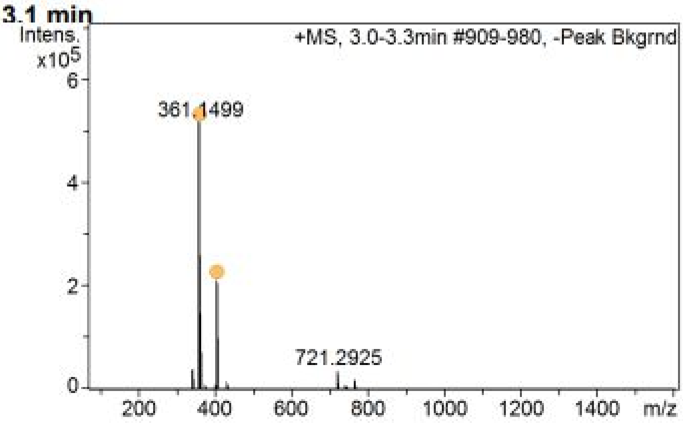

**Compound 19**

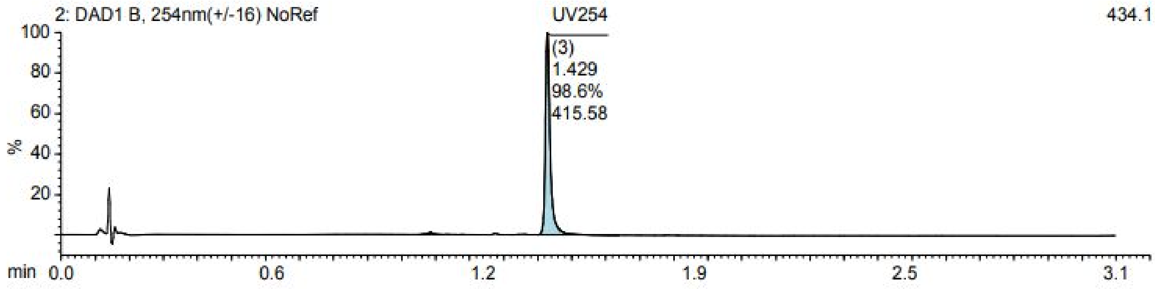

2: DAD1 B, 254nm(+/-16) NoRef
UV254
434.1
(3)
1.429
98.6%
415.58
%
min
0.0
0.6
1.2
1.9
2.5
3.1


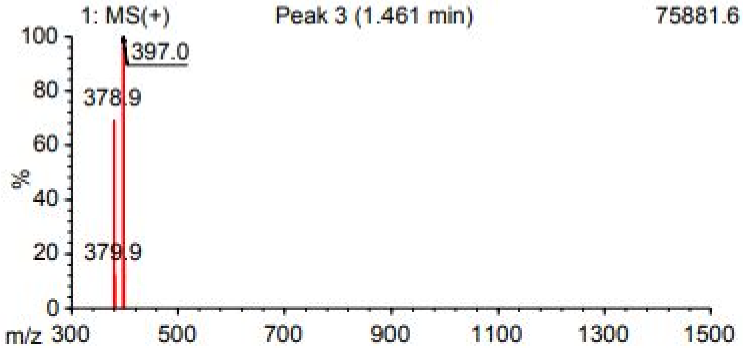

1: MS(+)
Peak 3 (1.461 min)
75881.6
397.0
378.9
379.9
%
m/z
300
500
700
900
1100
1300
1500

**Compound 20**

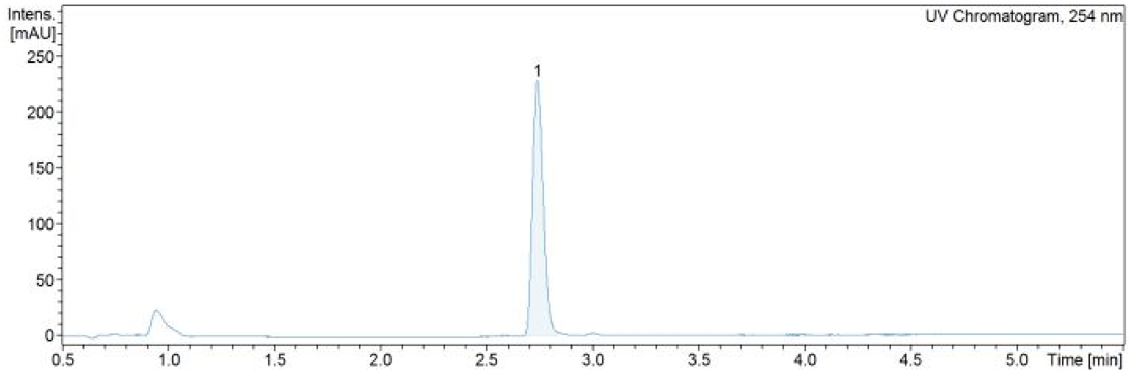

Intens.
[mAU]
UV Chromatogram, 254 nm
1
250
200
150
100
50
0
0.5
1.0
1.5
2.0
2.5
3.0
3.5
4.0
4.5
5.0
Time [min]


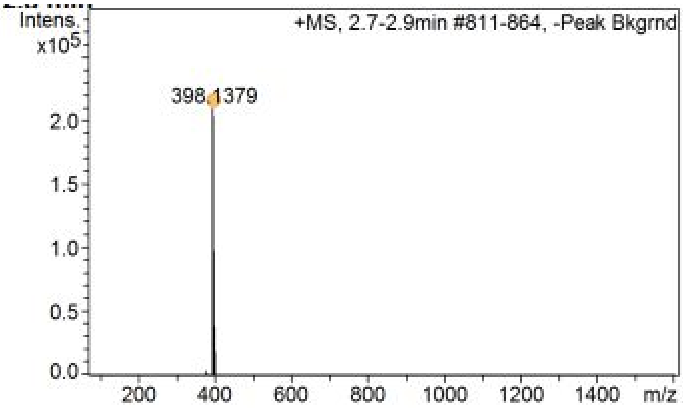

Intens.
x10^5
+MS, 2.7-2.9min #811-864, -Peak Bkgrnd
398.1379
2.0
1.5
1.0
0.5
0.0
200
400
600
800
1000
1200
1400
m/z

**Compound 21**

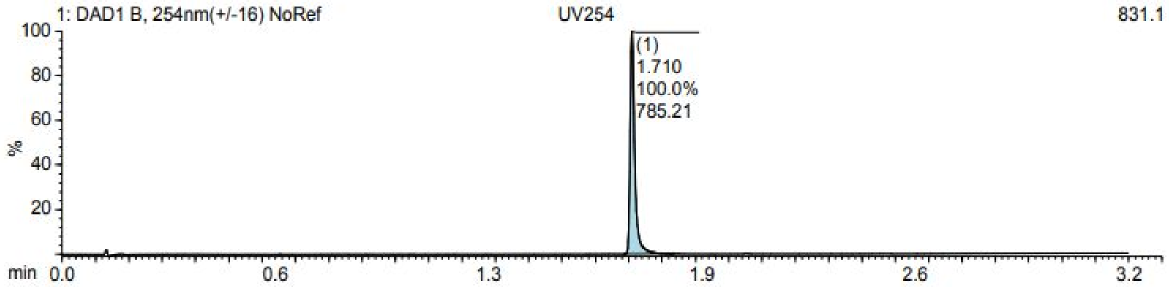

1: DAD1 B, 254nm(+/-16) NoRef
UV254
831.1
(1)
1.710
100.0%
785.21
%
min
0.0
0.6
1.3
1.9
2.6
3.2


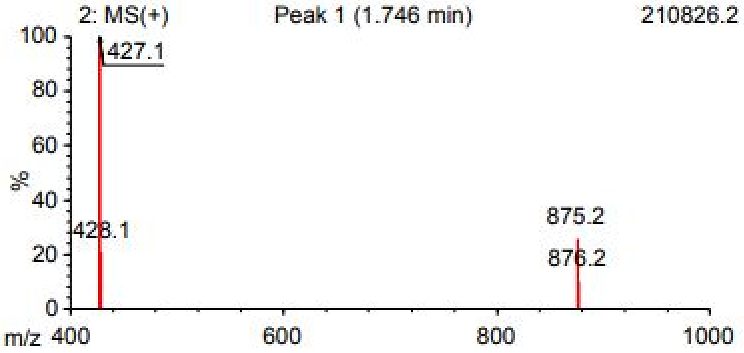

2: MS(+)
Peak 1 (1.746 min)
210826.2
427.1
428.1
875.2
876.2
%
m/z
400
600
800
1000

**Compound 22**

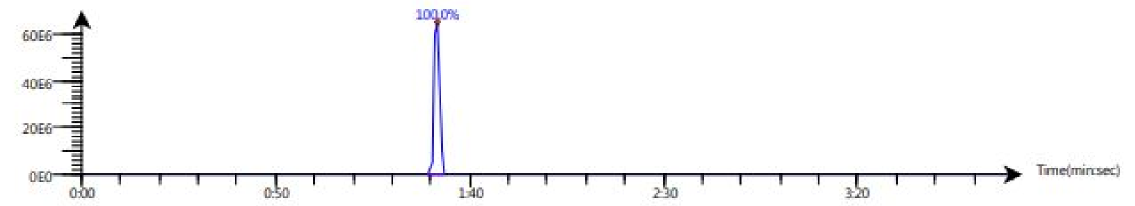
100.0%
60E6
40E6
20E6
0E0
0:00
0:50
1:40
2:30
3:20
Time(min:sec)

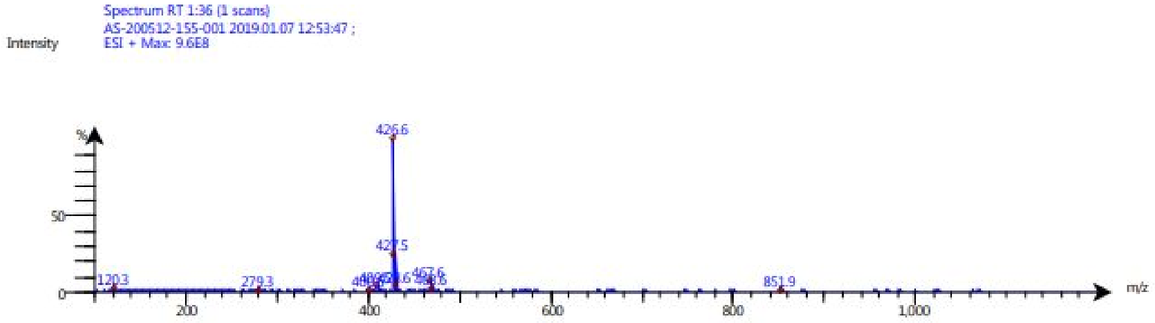
Intensity
Spectrum RT 1:36 (1 scans)
AS-200512-155-001 2019.01.07 12:53:47 ;
ESI + Max: 9.6E8
%
50
0
426.6
427.5
120.3
279.3
467.6
851.9
200
400
600
800
1,000
m/z

**Compound 23**

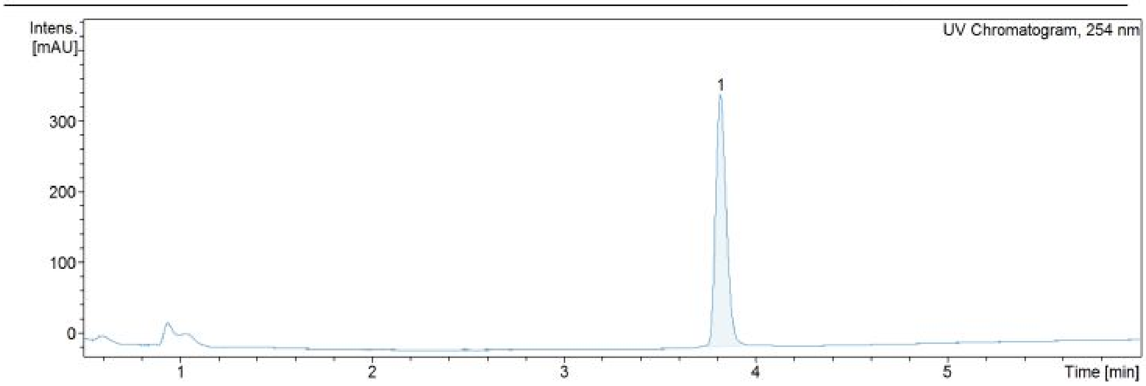


**Cmpd 2, 3.8 min**

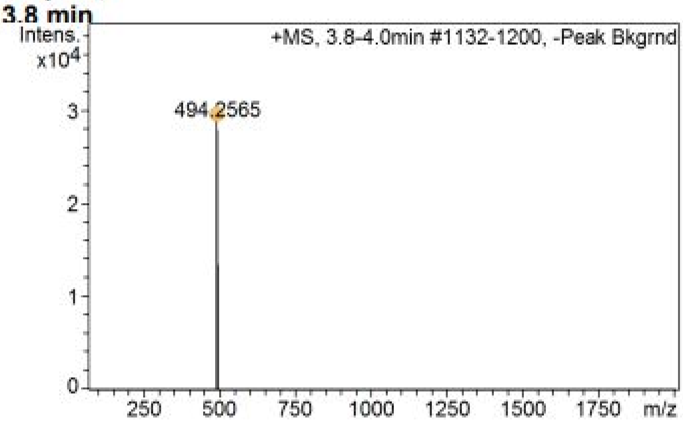

## Compound 24

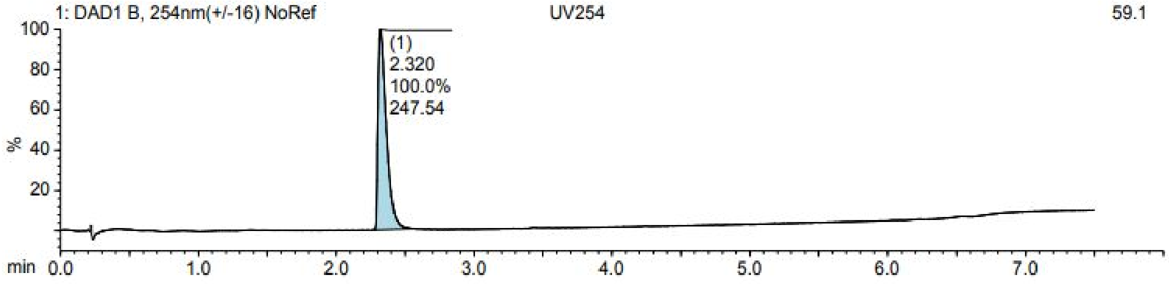

1: DAD1 B, 254nm(+/-16) NoRef
UV254
59.1
(1)
2.320
100.0%
247.54
%
min
0.0
1.0
2.0
3.0
4.0
5.0
6.0
7.0
100
80
60
40
20


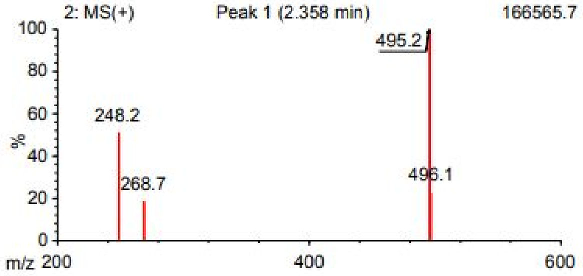

2: MS(+)
Peak 1 (2.358 min)
166565.7
495.2
248.2
268.7
496.1
%
m/z 200
400
600
100
80
60
40
20
0

## Compound 25

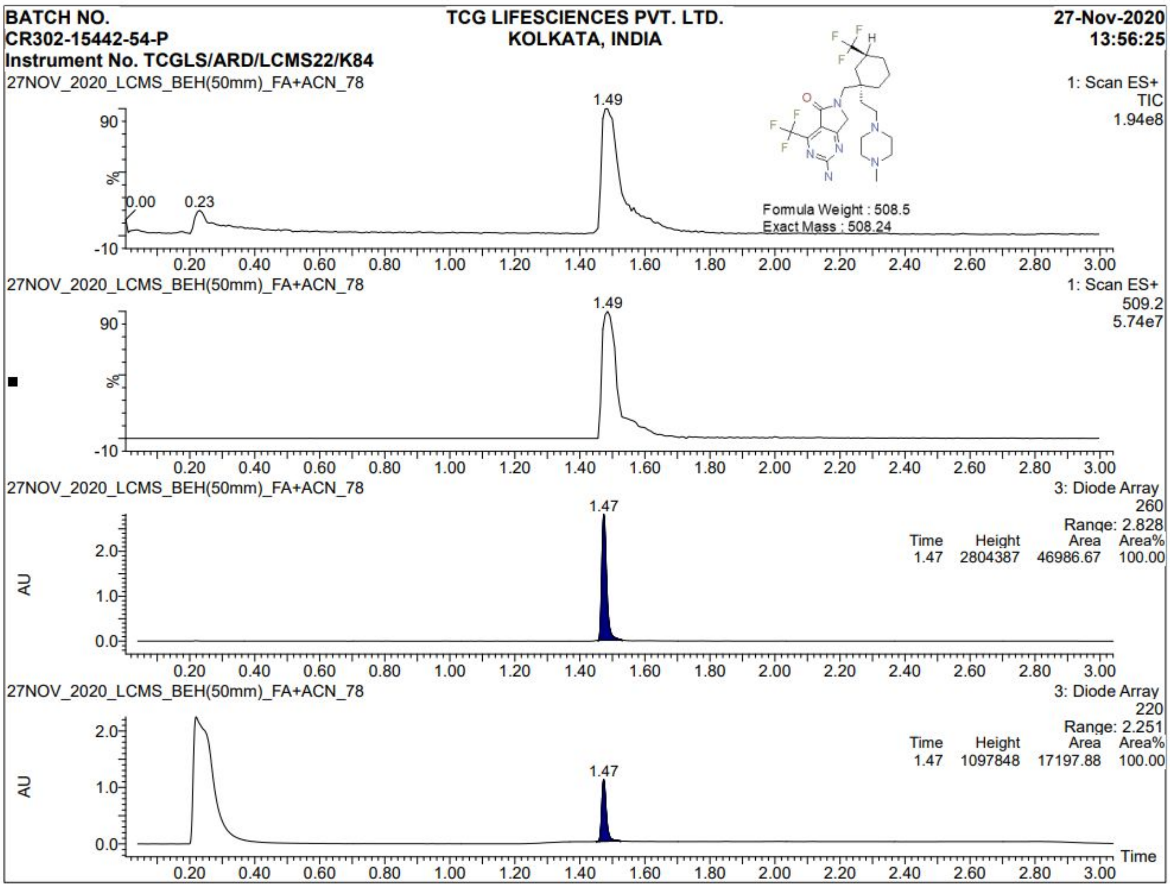
BATCH NO.
CR302-15442-54-P
Instrument No. TCGLS/ARD/LCMS22/K84
TCG LIFESCIENCES PVT. LTD.
KOLKATA, INDIA
27-Nov-2020
13:56:25
27NOV_2020_LCMS_BEH(50mm)_FA+ACN_78
1: Scan ES+
TIC
1.94e8
1.49
0.00
0.23
Formula Weight : 508.5
Exact Mass : 508.24
1: Scan ES+
509.2
5.74e7
3: Diode Array
260
Range: 2.828
Time Height Area Area%
1.47 2804387 46986.67 100.00
3: Diode Array
220
Range: 2.251
Time Height Area Area%
1.47 1097848 17197.88 100.00
1.47
AU
Time

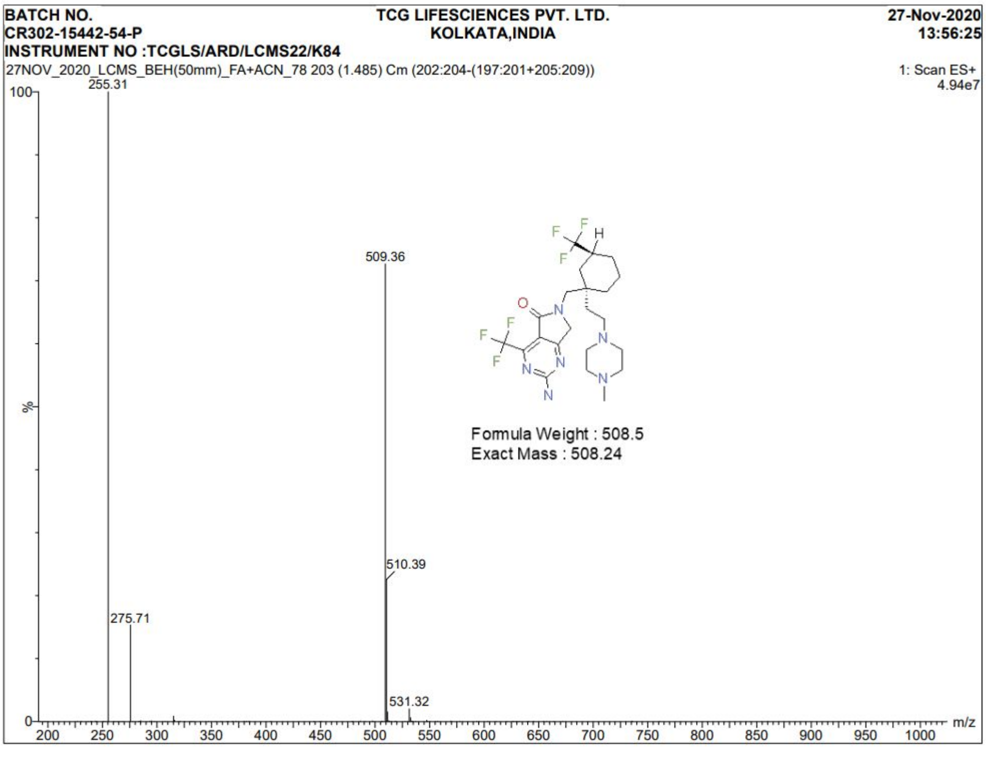
BATCH NO.
CR302-15442-54-P
INSTRUMENT NO :TCGLS/ARD/LCMS22/K84
TCG LIFESCIENCES PVT. LTD.
KOLKATA,INDIA
27-Nov-2020
13:56:25
27NOV_2020_LCMS_BEH(50mm)_FA+ACN_78 203 (1.485) Cm (202:204-(197:201+205:209))
1: Scan ES+
4.94e7
255.31
509.36
510.39
275.71
531.32
Formula Weight : 508.5
Exact Mass : 508.24
m/z

## Compound 26

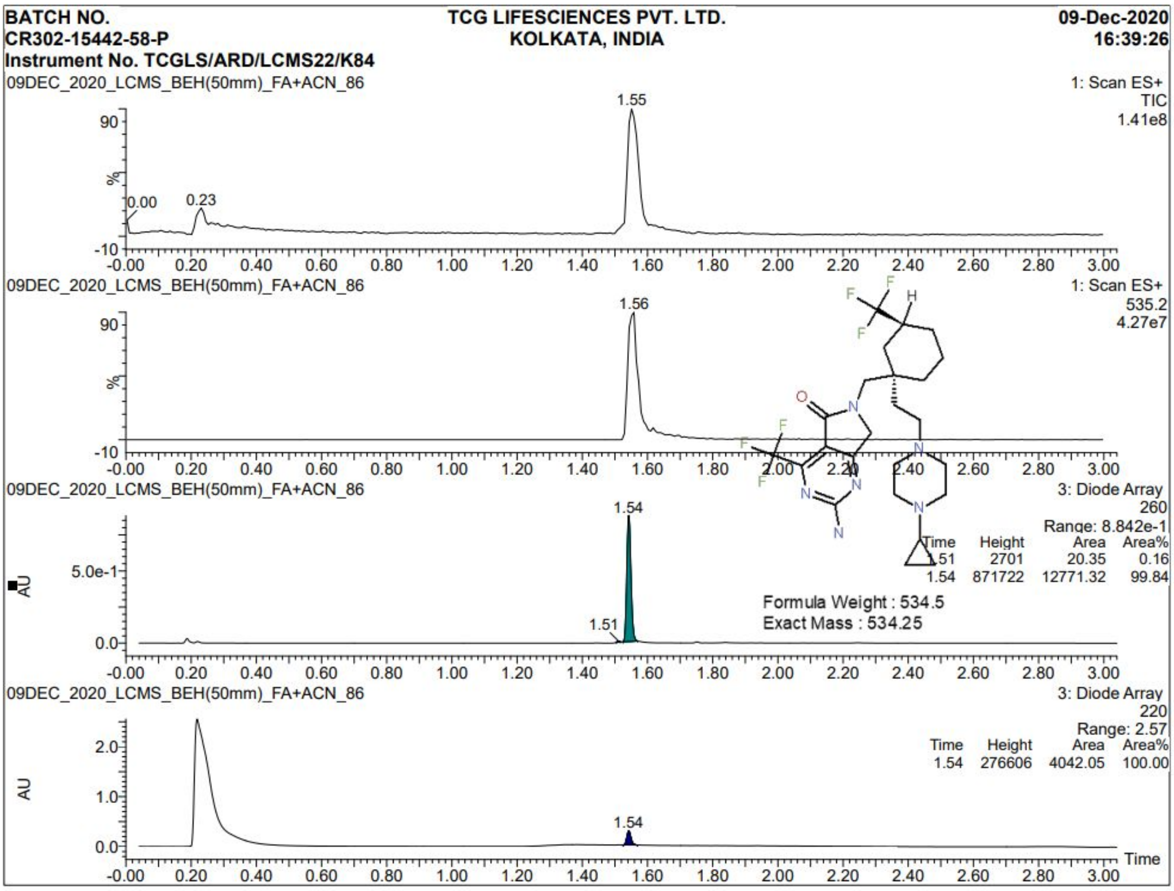
BATCH NO.
CR302-15442-58-P
Instrument No. TCGLS/ARD/LCMS22/K84
TCG LIFESCIENCES PVT. LTD.
KOLKATA, INDIA
09-Dec-2020
16:39:26
09DEC_2020_LCMS_BEH(50mm)_FA+ACN_86
1: Scan ES+
TIC
1.41e8
1.55
0.00
0.23
1: Scan ES+
535.2
4.27e7
1.56
3: Diode Array
260
Range: 8.842e-1
1.54
1.51
Time Height Area Area%
1.51 2701 20.35 0.16
1.54 871722 12771.32 99.84
Formula Weight : 534.5
Exact Mass : 534.25
3: Diode Array
220
Range: 2.57
Time Height Area Area%
1.54 276606 4042.05 100.00
AU
Time

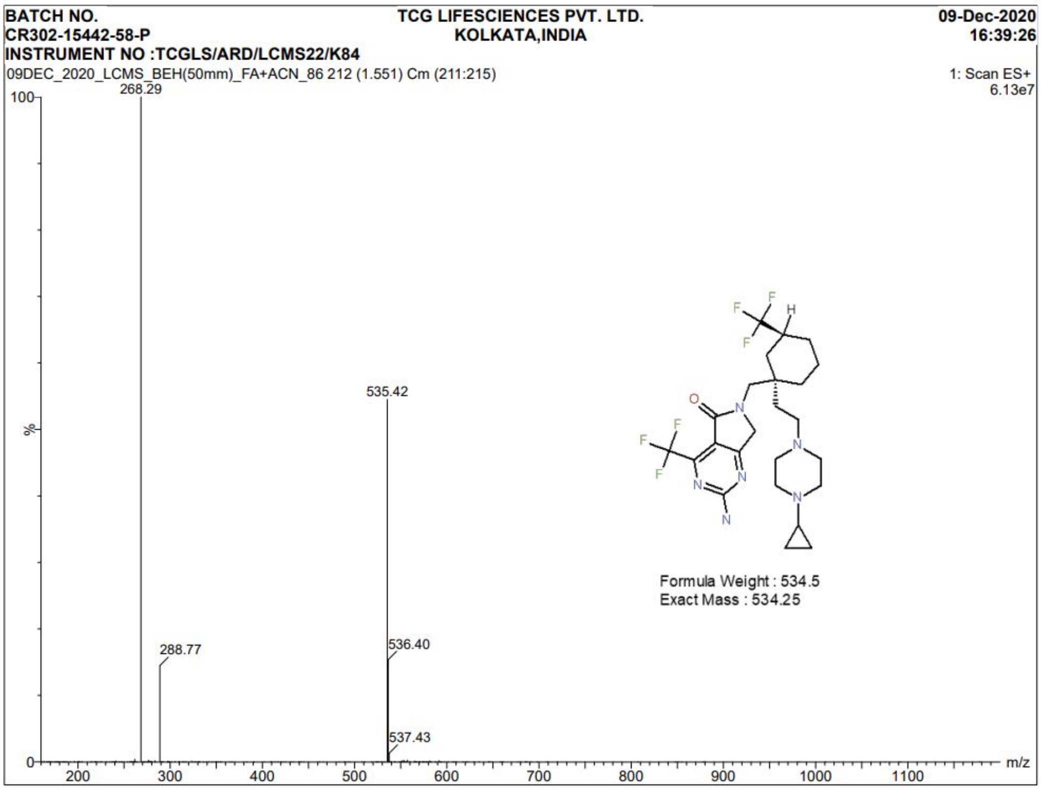
BATCH NO.
CR302-15442-58-P
INSTRUMENT NO :TCGLS/ARD/LCMS22/K84
TCG LIFESCIENCES PVT. LTD.
KOLKATA,INDIA
09-Dec-2020
16:39:26
09DEC_2020_LCMS_BEH(50mm)_FA+ACN_86 212 (1.551) Cm (211:215)
1: Scan ES+
6.13e7
268.29
288.77
535.42
536.40
537.43
Formula Weight : 534.5
Exact Mass : 534.25
m/z

**Compound 27**

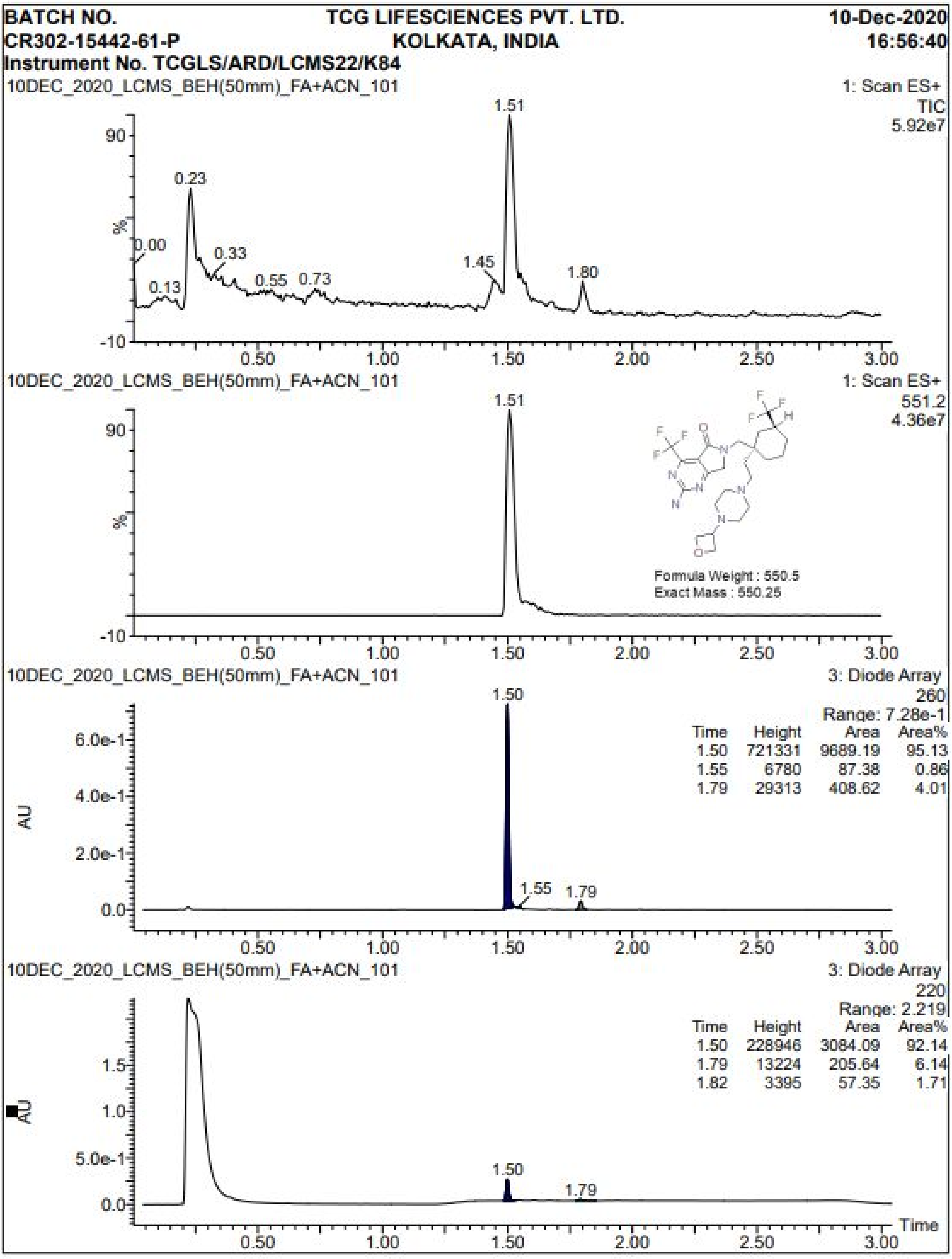

BATCH NO.
CR302-15442-61-P
Instrument No. TCGLS/ARD/LCMS22/K84
TCG LIFESCIENCES PVT. LTD.
KOLKATA, INDIA
10-Dec-2020
16:56:40
10DEC_2020_LCMS_BEH(50mm)_FA+ACN_101
1: Scan ES+
TIC
5.92e7
1.51
0.23
0.00
0.33
0.13
0.55 0.73
1.45
1.80
10DEC_2020_LCMS_BEH(50mm)_FA+ACN_101
1: Scan ES+
551.2
4.36e7
1.51
Formula Weight : 550.5
Exact Mass : 550.25
10DEC_2020_LCMS_BEH(50mm)_FA+ACN_101
3: Diode Array
260
Range: 7.28e-1
Time Height Area Area%
1.50 721331 9689.19 95.13
1.55 6780 87.38 0.86
1.79 29313 408.62 4.01
1.50
1.55 1.79
10DEC_2020_LCMS_BEH(50mm)_FA+ACN_101
3: Diode Array
220
Range: 2.219
Time Height Area Area%
1.50 228946 3084.09 92.14
1.79 13224 205.64 6.14
1.82 3395 57.35 1.71
1.50
1.79
Time

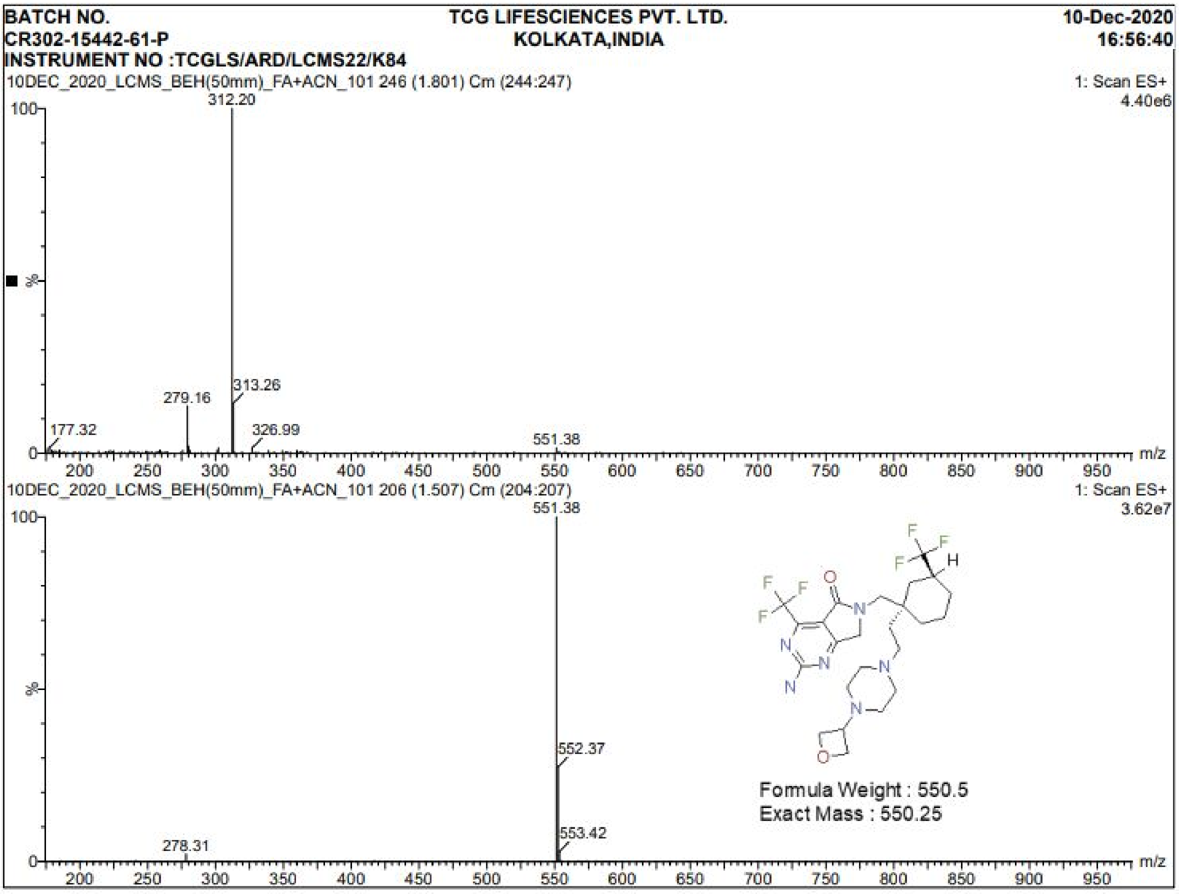
BATCH NO.
CR302-15442-61-P
INSTRUMENT NO :TCGLS/ARD/LCMS22/K84
TCG LIFESCIENCES PVT. LTD.
KOLKATA,INDIA
10-Dec-2020
16:56:40
10DEC_2020_LCMS_BEH(50mm)_FA+ACN_101 246 (1.801) Cm (244:247)
1: Scan ES+
4.40e6
312.20
313.26
279.16
177.32
326.99
551.38
10DEC_2020_LCMS_BEH(50mm)_FA+ACN_101 206 (1.507) Cm (204:207)
1: Scan ES+
3.62e7
551.38
552.37
553.42
278.31
m/z
Formula Weight : 550.5
Exact Mass : 550.25

**Compound 28**

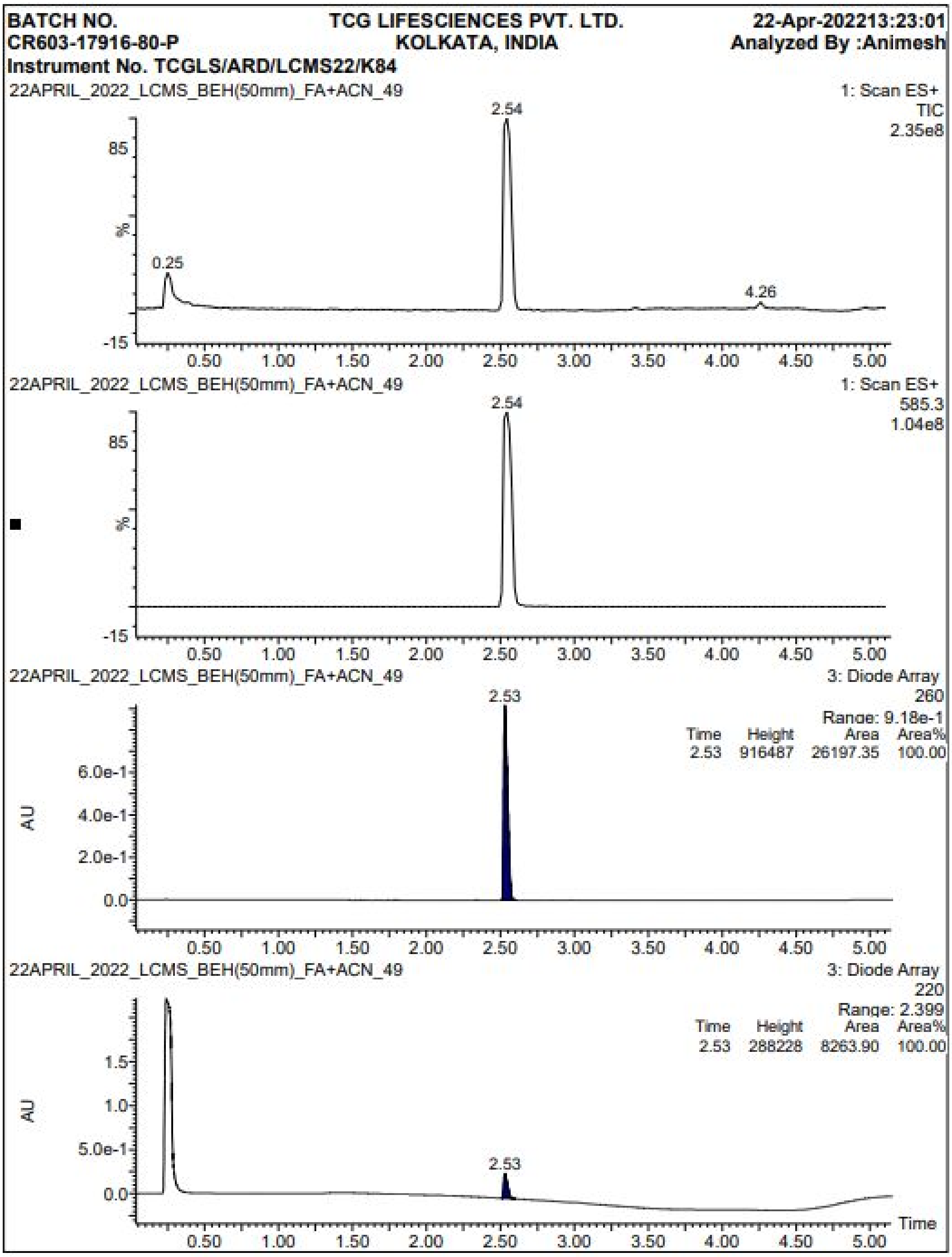
BATCH NO.
CR603-17916-80-P
TCG LIFESCIENCES PVT. LTD.
KOLKATA, INDIA
22-Apr-202213:23:01
Analyzed By :Animesh
Instrument No. TCGLS/ARD/LCMS22/K84
22APRIL_2022_LCMS_BEH(50mm)_FA+ACN_49
1: Scan ES+
TIC
2.35e8
2.54
0.25
4.26
1: Scan ES+
585.3
1.04e8
3: Diode Array
260
Range: 9.18e-1
Time Height Area Area%
2.53 916487 26197.35 100.00
3: Diode Array
220
Range: 2.399
Time Height Area Area%
2.53 288228 8263.90 100.00
AU
Time

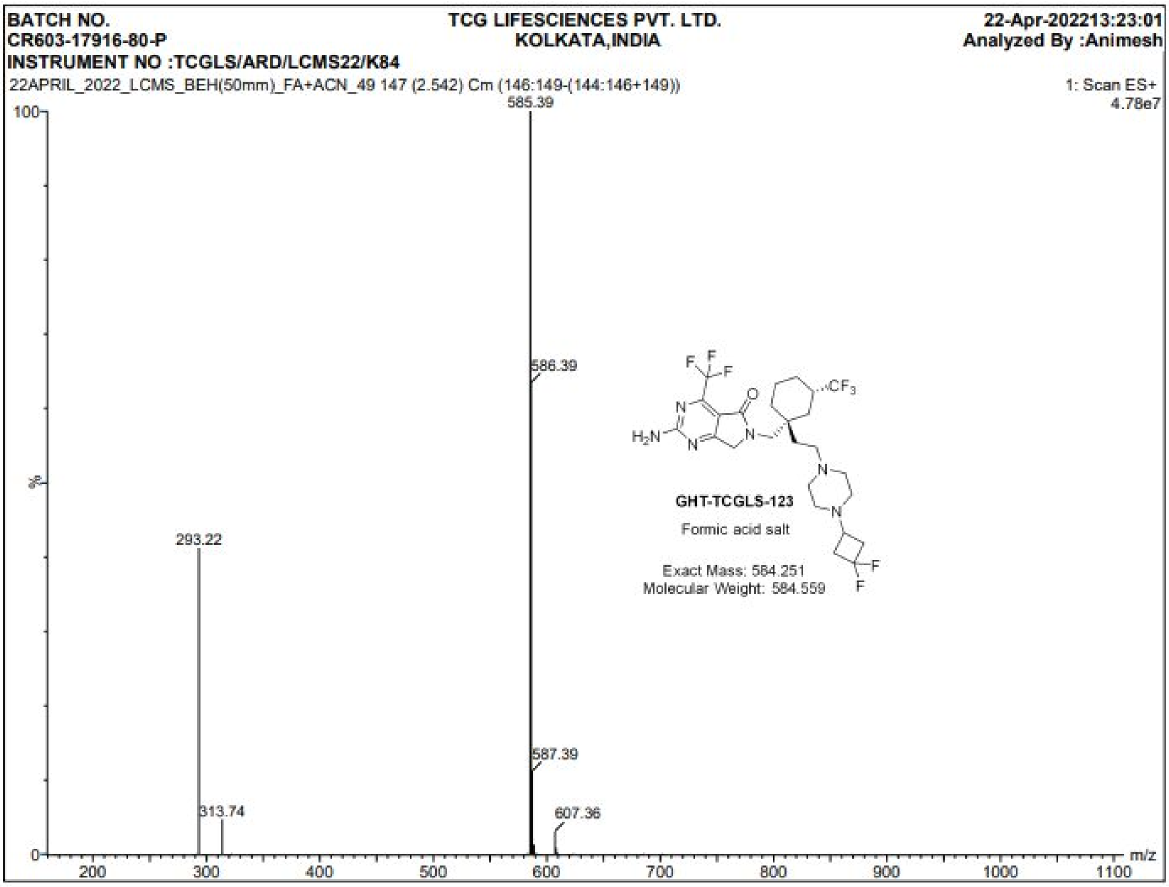
BATCH NO.
CR603-17916-80-P
INSTRUMENT NO :TCGLS/ARD/LCMS22/K84
TCG LIFESCIENCES PVT. LTD.
KOLKATA,INDIA
22-Apr-202213:23:01
Analyzed By :Animesh
22APRIL_2022_LCMS_BEH(50mm)_FA+ACN_49 147 (2.542) Cm (146:149-(144:146+149))
1: Scan ES+
4.78e7
585.39
586.39
293.22
587.39
313.74
607.36
100
%
0
m/z
200
300
400
500
600
700
800
900
1000
1100
GHT-TCGLS-123
Formic acid salt
Exact Mass: 584.251
Molecular Weight: 584.559

**Compound 29**

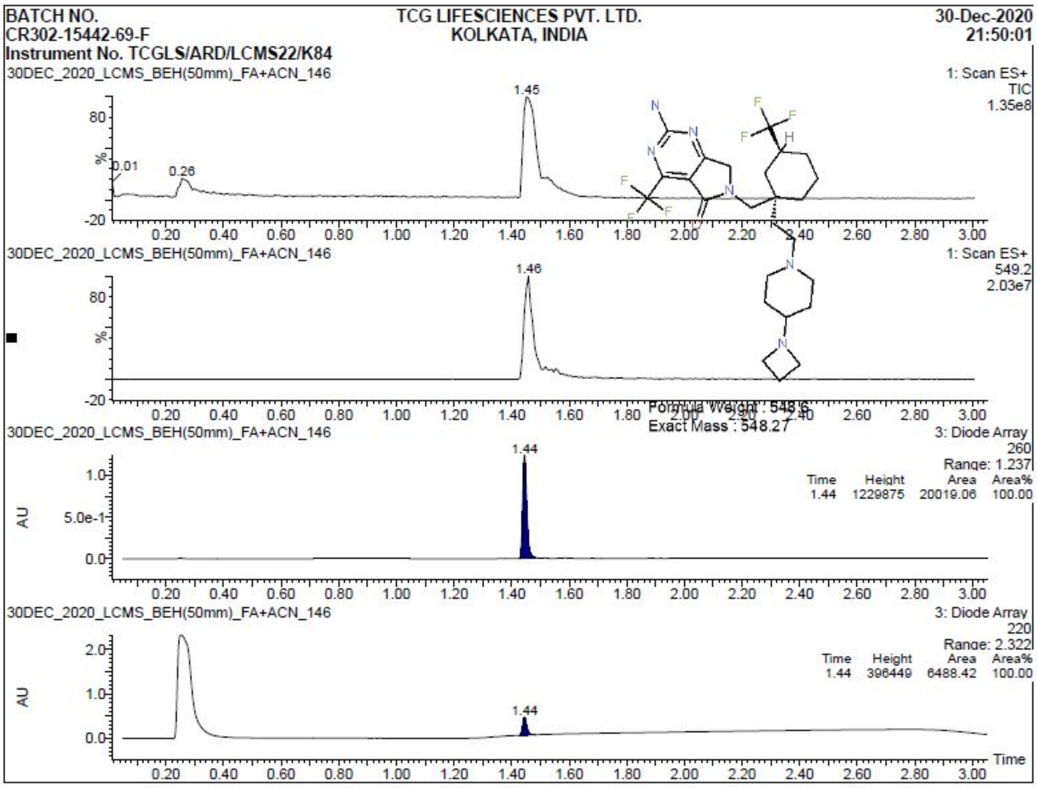

BATCH NO.
CR302-15442-69-F
Instrument No. TCGLS/ARD/LCMS22/K84
TCG LIFESCIENCES PVT. LTD.
KOLKATA, INDIA
30-Dec-2020
21:50:01
30DEC_2020_LCMS_BEH(50mm)_FA+ACN_146
1: Scan ES+
TIC
1.35e8
1: Scan ES+
549.2
2.03e7
Formula Weight : 548.6
Exact Mass : 548.27
3: Diode Array
260
Range: 1.237
Time Height Area Area%
1.44 1229875 20019.06 100.00
3: Diode Array
220
Range: 2.322
Time Height Area Area%
1.44 396449 6488.42 100.00
Time
AU


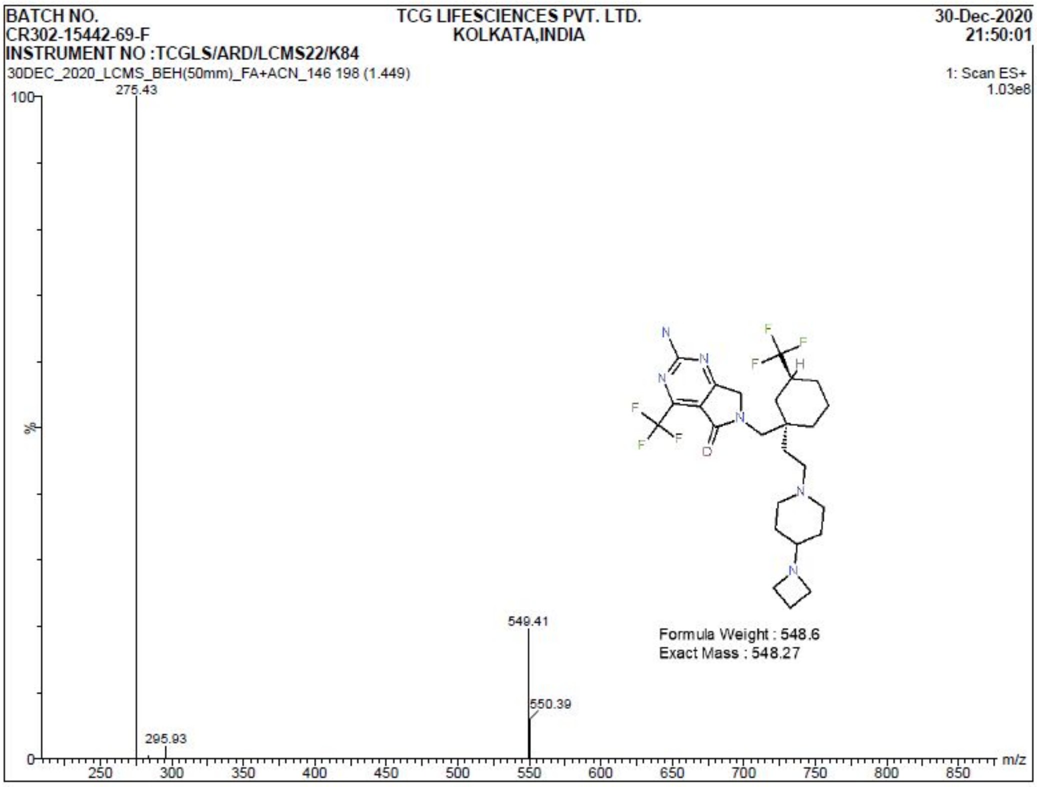

BATCH NO.
CR302-15442-69-F
INSTRUMENT NO :TCGLS/ARD/LCMS22/K84
TCG LIFESCIENCES PVT. LTD.
KOLKATA,INDIA
30-Dec-2020
21:50:01
30DEC_2020_LCMS_BEH(50mm)_FA+ACN_146 198 (1.449)
1: Scan ES+
1.03e8
275.43
295.93
549.41
550.39
Formula Weight : 548.6
Exact Mass : 548.27
m/z


**Compound 30**

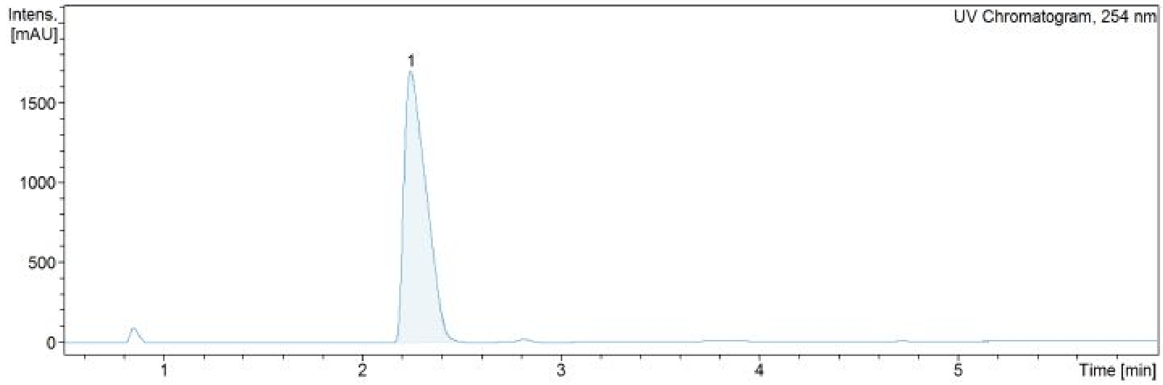
Intens.
[mAU]
UV Chromatogram, 254 nm
1
1500
1000
500
0
1
2
3
4
5
Time [min]

**Cmpd 2, 2.3 min**

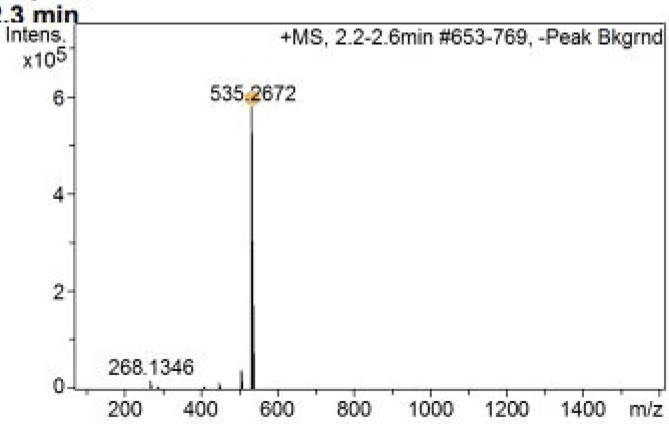
Intens.
x10^5
+MS, 2.2-2.6min #653-769, -Peak Bkgrnd
535.2672
268.1346
6
4
2
0
200
400
600
800
1000
1200
1400
m/z

**Compound 31**

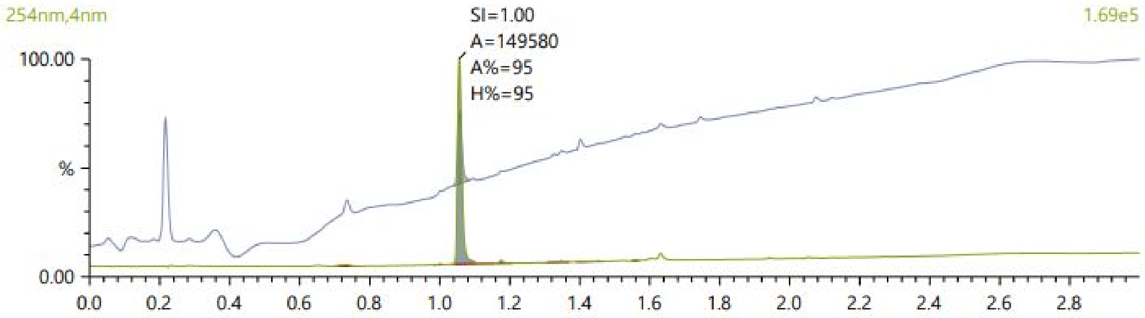

254nm,4nm
SI=1.00
A=149580
A%=95
H%=95
1.69e5
100.00
%
0.00
0.0 0.2 0.4 0.6 0.8 1.0 1.2 1.4 1.6 1.8 2.0 2.2 2.4 2.6 2.8


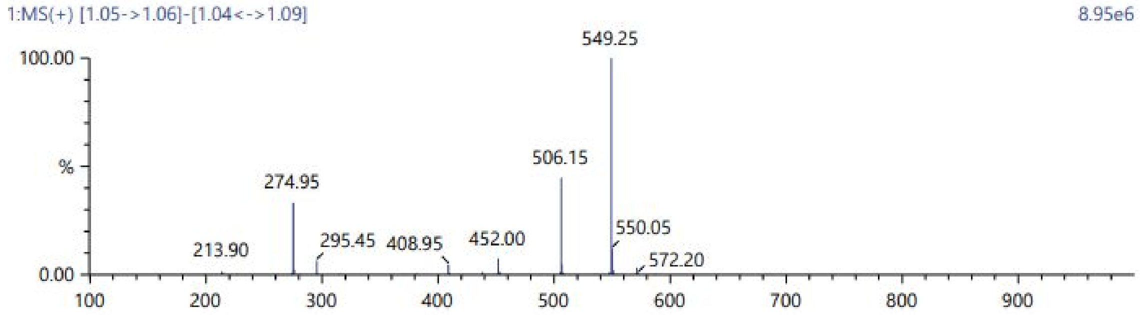

1:MS(+) [1.05->1.06]-[1.04<->1.09]
8.95e6
549.25
100.00
%
274.95
506.15
213.90
295.45
408.95
452.00
550.05
572.20
0.00
100 200 300 400 500 600 700 800 900

**Compound 32**

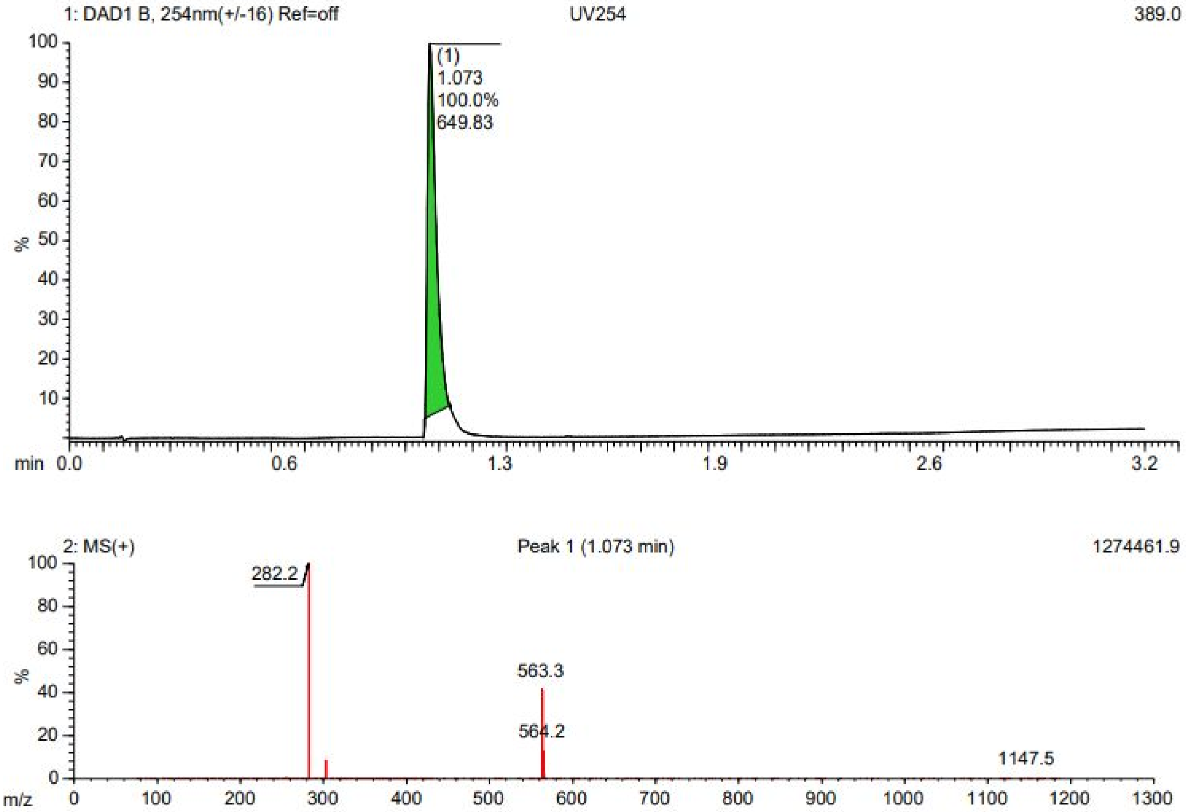

1: DAD1 B, 254nm(+/-16) Ref=off
UV254
389.0
(1)
1.073
100.0%
649.83
%
min
2: MS(+)
Peak 1 (1.073 min)
1274461.9
282.2
563.3
564.2
1147.5
m/z

## Compound 33

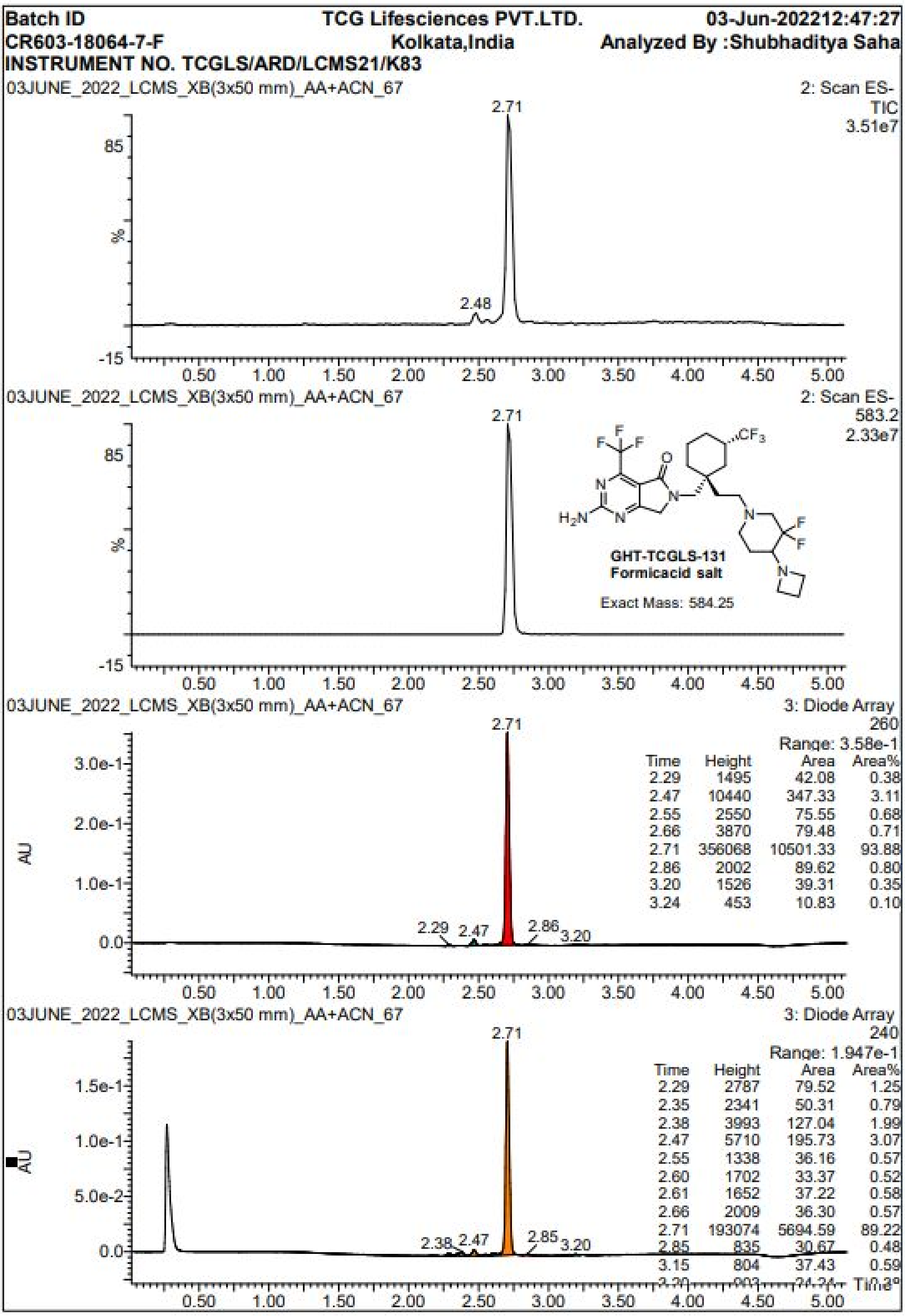


| Time | Height | Area | Area% |
|---|---|---|---|
| 2.29 | 1495 | 42.08 | 0.38 |
| 2.47 | 10440 | 347.33 | 3.11 |
| 2.55 | 2550 | 75.55 | 0.68 |
| 2.66 | 3870 | 79.48 | 0.71 |
| 2.71 | 356068 | 10501.33 | 93.88 |
| 2.86 | 2002 | 89.62 | 0.80 |
| 3.20 | 1526 | 39.31 | 0.35 |
| 3.24 | 453 | 10.83 | 0.10 |

| Time | Height | Area | Area% |
|---|---|---|---|
| 2.29 | 2787 | 79.52 | 1.25 |
| 2.35 | 2341 | 50.31 | 0.79 |
| 2.38 | 3993 | 127.04 | 1.99 |
| 2.47 | 5710 | 195.73 | 3.07 |
| 2.55 | 1338 | 36.16 | 0.57 |
| 2.60 | 1702 | 33.37 | 0.52 |
| 2.61 | 1652 | 37.22 | 0.58 |
| 2.66 | 2009 | 36.30 | 0.57 |
| 2.71 | 193074 | 5694.59 | 89.22 |
| 2.85 | 835 | 30.67 | 0.48 |
| 3.15 | 804 | 37.43 | 0.59 |
| 3.20 | [illegible] | [illegible] | [illegible] |

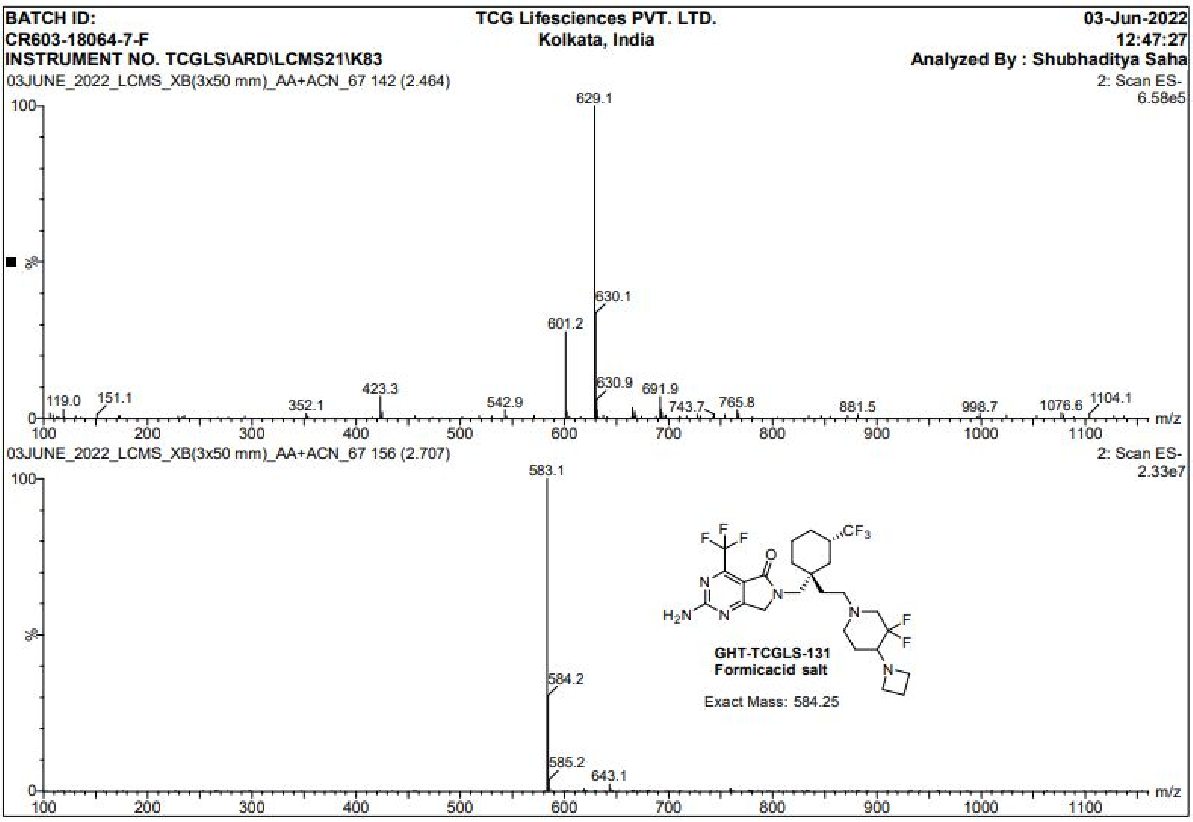

BATCH ID:
CR603-18064-7-F
INSTRUMENT NO. TCGLS\ARD\LCMS21\K83
TCG Lifesciences PVT. LTD.
Kolkata, India
03-Jun-2022
12:47:27
Analyzed By : Shubhaditya Saha
03JUNE_2022_LCMS_XB(3x50 mm)_AA+ACN_67 142 (2.464)
2: Scan ES-
6.58e5
629.1
630.1
601.2
423.3
630.9
691.9
119.0
151.1
352.1
542.9
743.7
765.8
881.5
998.7
1076.6
1104.1
m/z
03JUNE_2022_LCMS_XB(3x50 mm)_AA+ACN_67 156 (2.707)
2: Scan ES-
2.33e7
583.1
584.2
585.2
643.1
GHT-TCGLS-131
Formicacid salt
Exact Mass: 584.25

## Compound 34

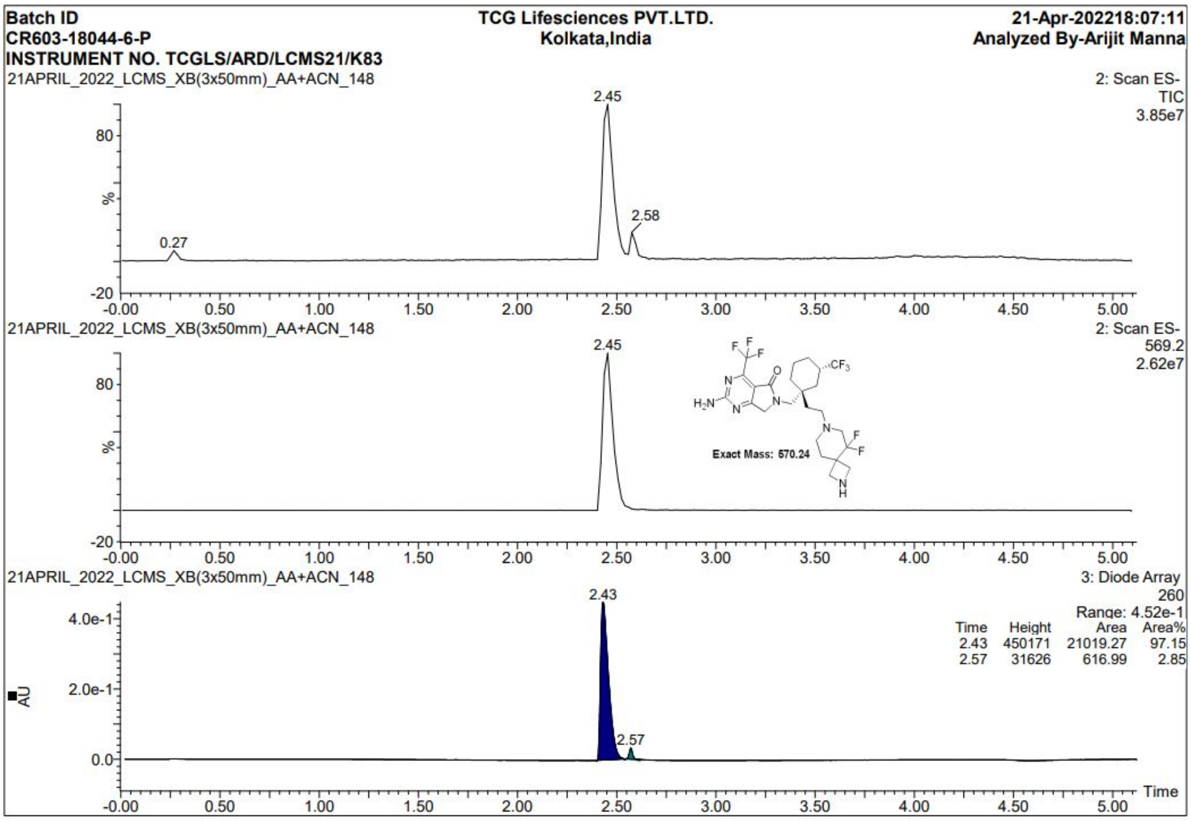

Batch ID
CR603-18044-6-P
INSTRUMENT NO. TCGLS/ARD/LCMS21/K83
TCG Lifesciences PVT.LTD.
Kolkata,India
21-Apr-202218:07:11
Analyzed By-Arijit Manna
21APRIL_2022_LCMS_XB(3x50mm)_AA+ACN_148
2: Scan ES-
TIC
3.85e7
2: Scan ES-
569.2
2.62e7
Exact Mass: 570.24
3: Diode Array
260
Range: 4.52e-1
Time Height Area Area%
2.43 450171 21019.27 97.15
2.57 31626 616.99 2.85


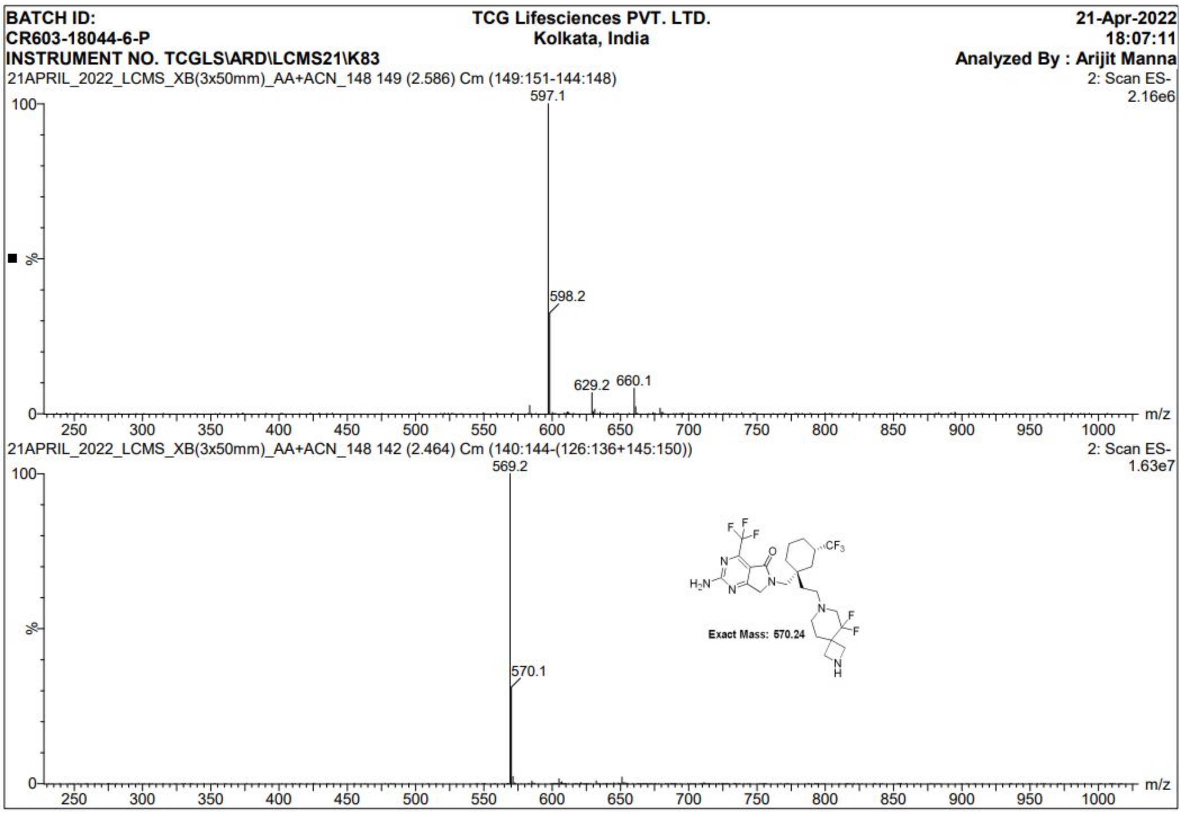

BATCH ID:
CR603-18044-6-P
INSTRUMENT NO. TCGLS\ARD\LCMS21\K83
TCG Lifesciences PVT. LTD.
Kolkata, India
21-Apr-2022
18:07:11
Analyzed By : Arijit Manna
21APRIL_2022_LCMS_XB(3x50mm)_AA+ACN_148 149 (2.586) Cm (149:151-144:148)
2: Scan ES-
2.16e6
597.1
598.2
629.2
660.1
21APRIL_2022_LCMS_XB(3x50mm)_AA+ACN_148 142 (2.464) Cm (140:144-(126:136+145:150))
2: Scan ES-
1.63e7
569.2
570.1
Exact Mass: 570.24

**Compound 35**

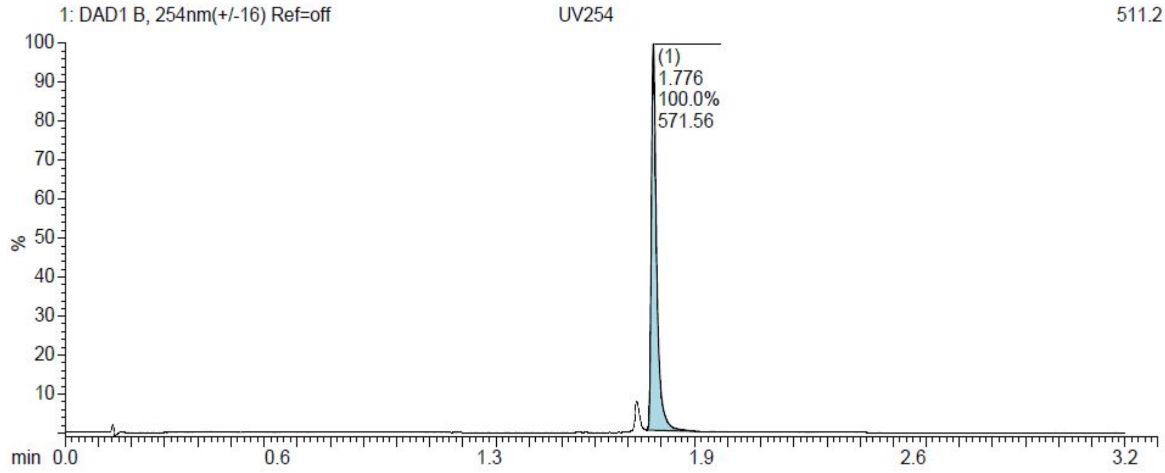

1: DAD1 B, 254nm(+/-16) Ref=off
UV254
511.2
(1)
1.776
100.0%
571.56
%
min
0.0
0.6
1.3
1.9
2.6
3.2


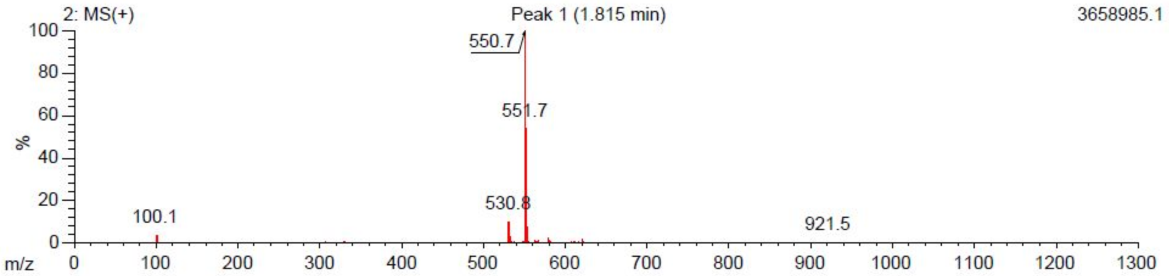

2: MS(+)
Peak 1 (1.815 min)
3658985.1
550.7
551.7
530.8
100.1
921.5
%
m/z

**Compound 36**

**Batch ID** **TCG Lifesciences PVT.LTD.** **16-Mar-202217:43:55**
**CR603-17916-27-F-AA** **Kolkata,India** **Analyzed By- Shubhaditya Saha**
**INSTRUMENT NO. TCGLS/ARD/LCMS21/K83**

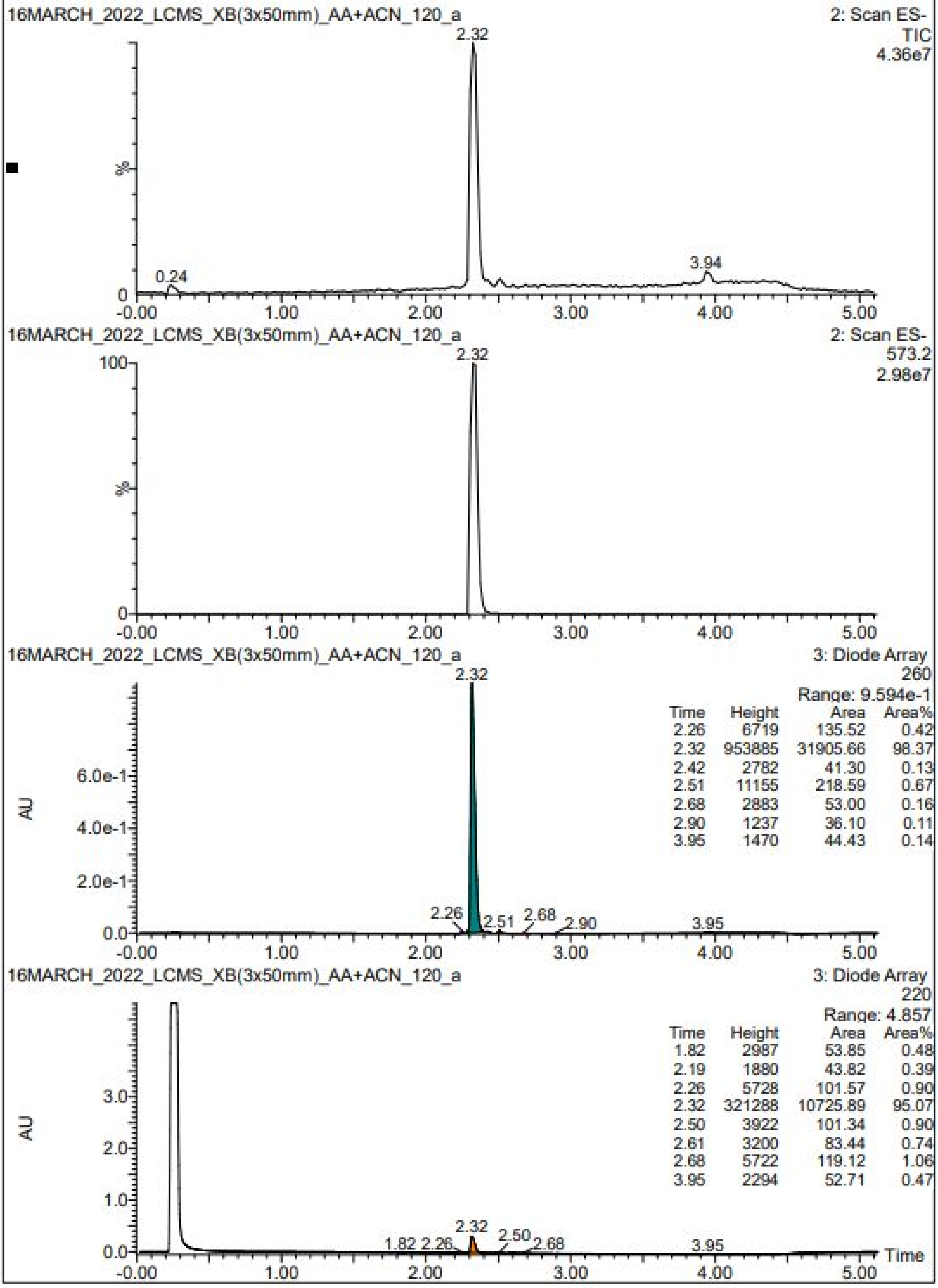


| Time | Height | Area | Area% |
|---|---|---|---|
| 2.26 | 6719 | 135.52 | 0.42 |
| 2.32 | 953885 | 31905.66 | 98.37 |
| 2.42 | 2782 | 41.30 | 0.13 |
| 2.51 | 11155 | 218.59 | 0.67 |
| 2.68 | 2883 | 53.00 | 0.16 |
| 2.90 | 1237 | 36.10 | 0.11 |
| 3.95 | 1470 | 44.43 | 0.14 |

| Time | Height | Area | Area% |
|---|---|---|---|
| 1.82 | 2987 | 53.85 | 0.48 |
| 2.19 | 1880 | 43.82 | 0.39 |
| 2.26 | 5728 | 101.57 | 0.90 |
| 2.32 | 321288 | 10725.89 | 95.07 |
| 2.50 | 3922 | 101.34 | 0.90 |
| 2.61 | 3200 | 83.44 | 0.74 |
| 2.68 | 5722 | 119.12 | 1.06 |
| 3.95 | 2294 | 52.71 | 0.47 |

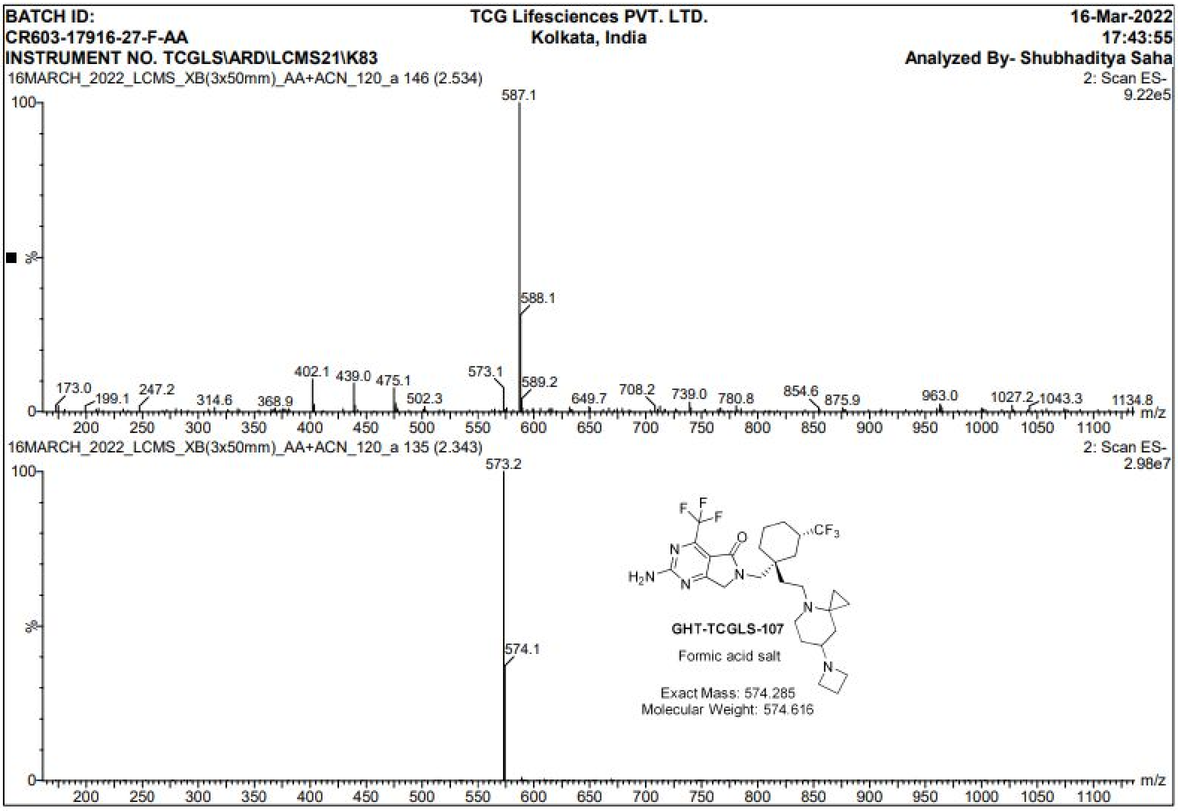
BATCH ID:
CR603-17916-27-F-AA
INSTRUMENT NO. TCGLS\ARD\LCMS21\K83
TCG Lifesciences PVT. LTD.
Kolkata, India
16-Mar-2022
17:43:55
Analyzed By- Shubhaditya Saha
16MARCH_2022_LCMS_XB(3x50mm)_AA+ACN_120_a 146 (2.534)
2: Scan ES-
9.22e5
587.1
588.1
173.0 199.1 247.2 314.6 368.9 402.1 439.0 475.1 502.3 573.1 589.2 649.7 708.2 739.0 780.8 854.6 875.9 963.0 1027.2 1043.3 1134.8
16MARCH_2022_LCMS_XB(3x50mm)_AA+ACN_120_a 135 (2.343)
2: Scan ES-
2.98e7
573.2
574.1
%
m/z
GHT-TCGLS-107
Formic acid salt
Exact Mass: 574.285
Molecular Weight: 574.616

## Compound 37

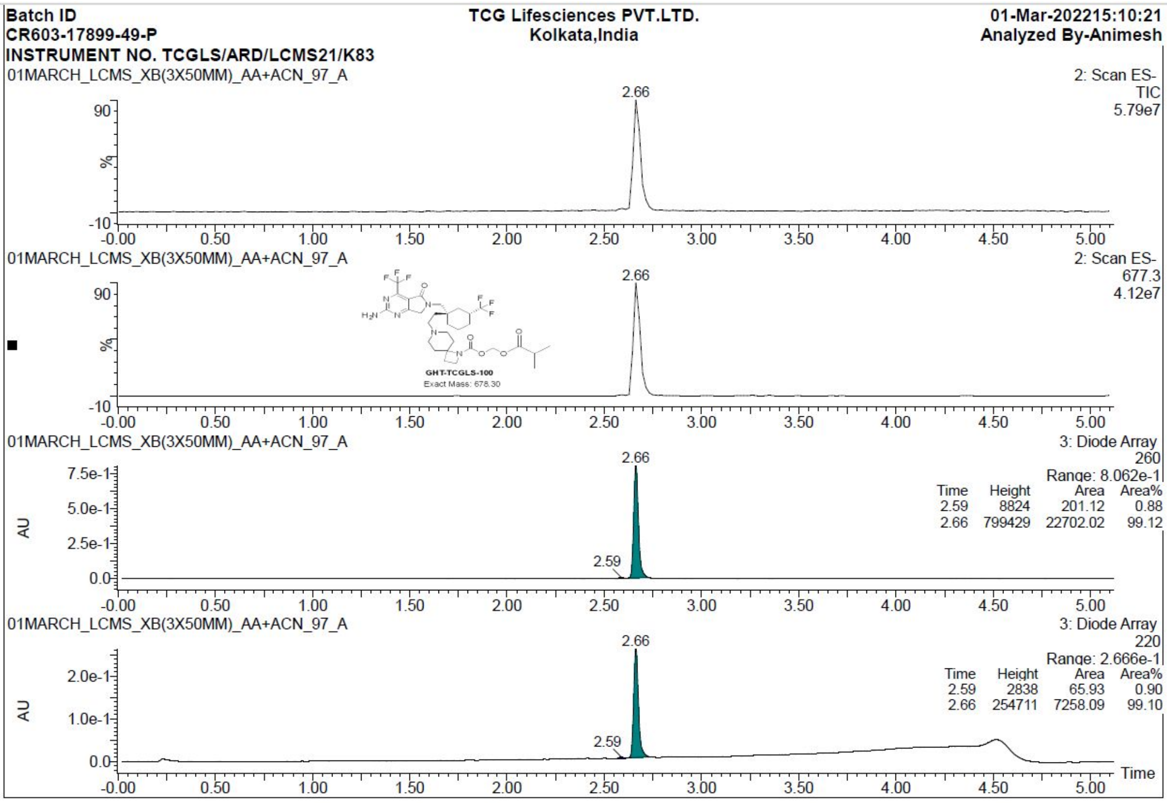


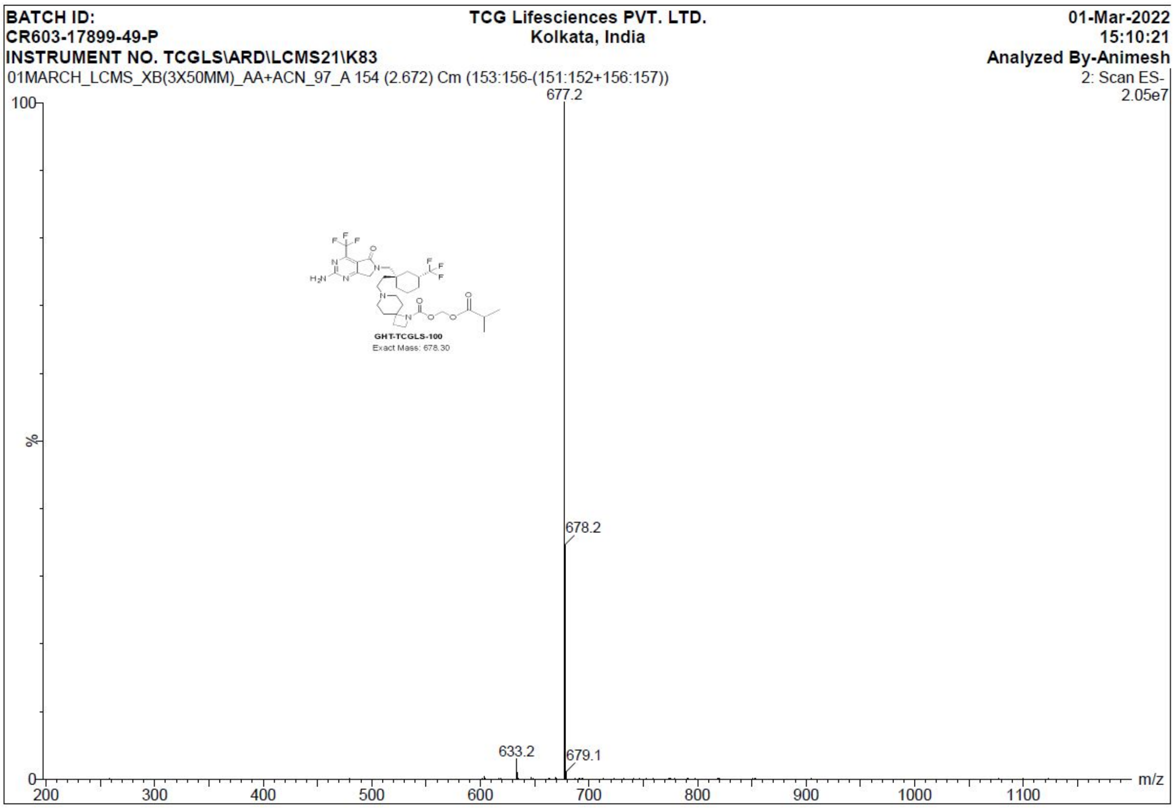


## Compound 38

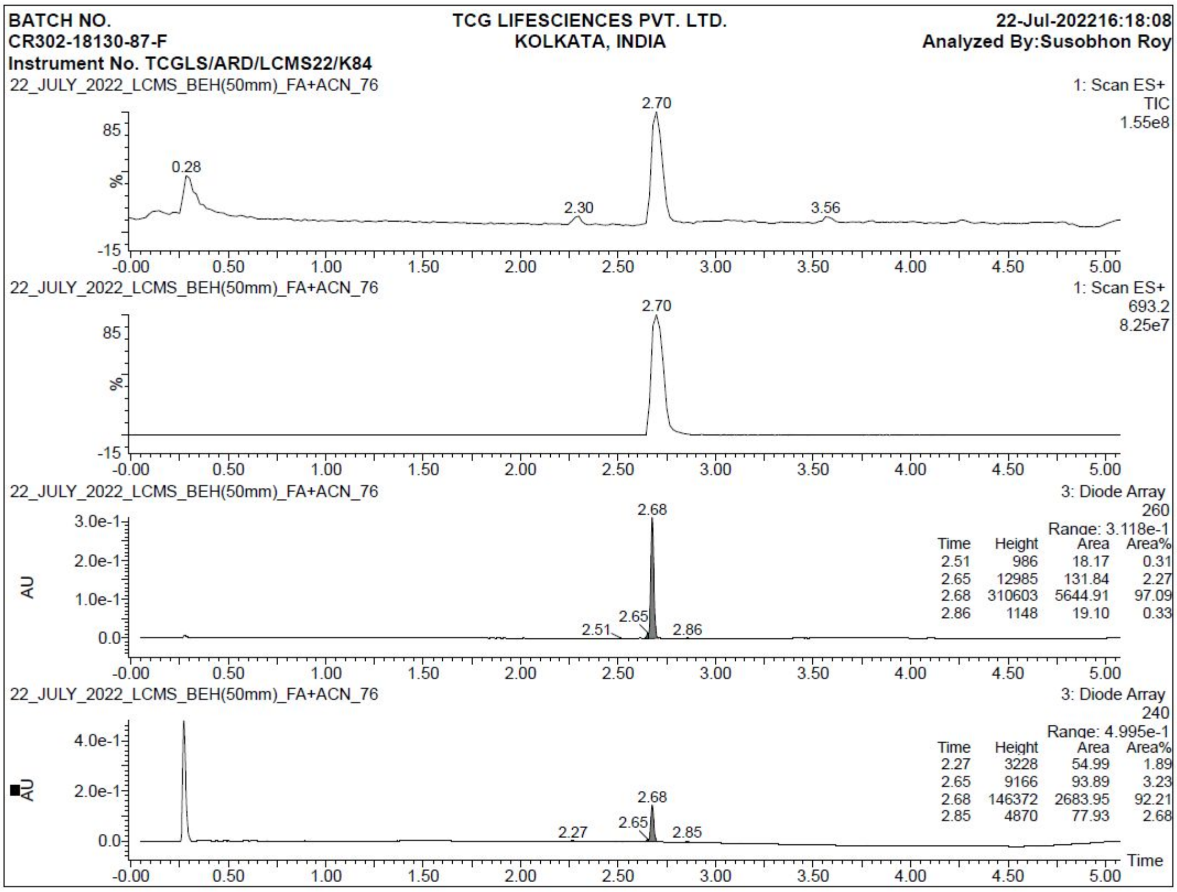
BATCH NO.
CR302-18130-87-F
Instrument No. TCGLS/ARD/LCMS22/K84
TCG LIFESCIENCES PVT. LTD.
KOLKATA, INDIA
22-Jul-202216:18:08
Analyzed By:Susobhon Roy
22_JULY_2022_LCMS_BEH(50mm)_FA+ACN_76
1: Scan ES+
TIC
1.55e8
0.28
2.30
2.70
3.56
1: Scan ES+
693.2
8.25e7
3: Diode Array
260
Range: 3.118e-1
Time Height Area Area%
2.51 986 18.17 0.31
2.65 12985 131.84 2.27
2.68 310603 5644.91 97.09
2.86 1148 19.10 0.33
3: Diode Array
240
Range: 4.995e-1
Time Height Area Area%
2.27 3228 54.99 1.89
2.65 9166 93.89 3.23
2.68 146372 2683.95 92.21
2.85 4870 77.93 2.68
AU
%
Time

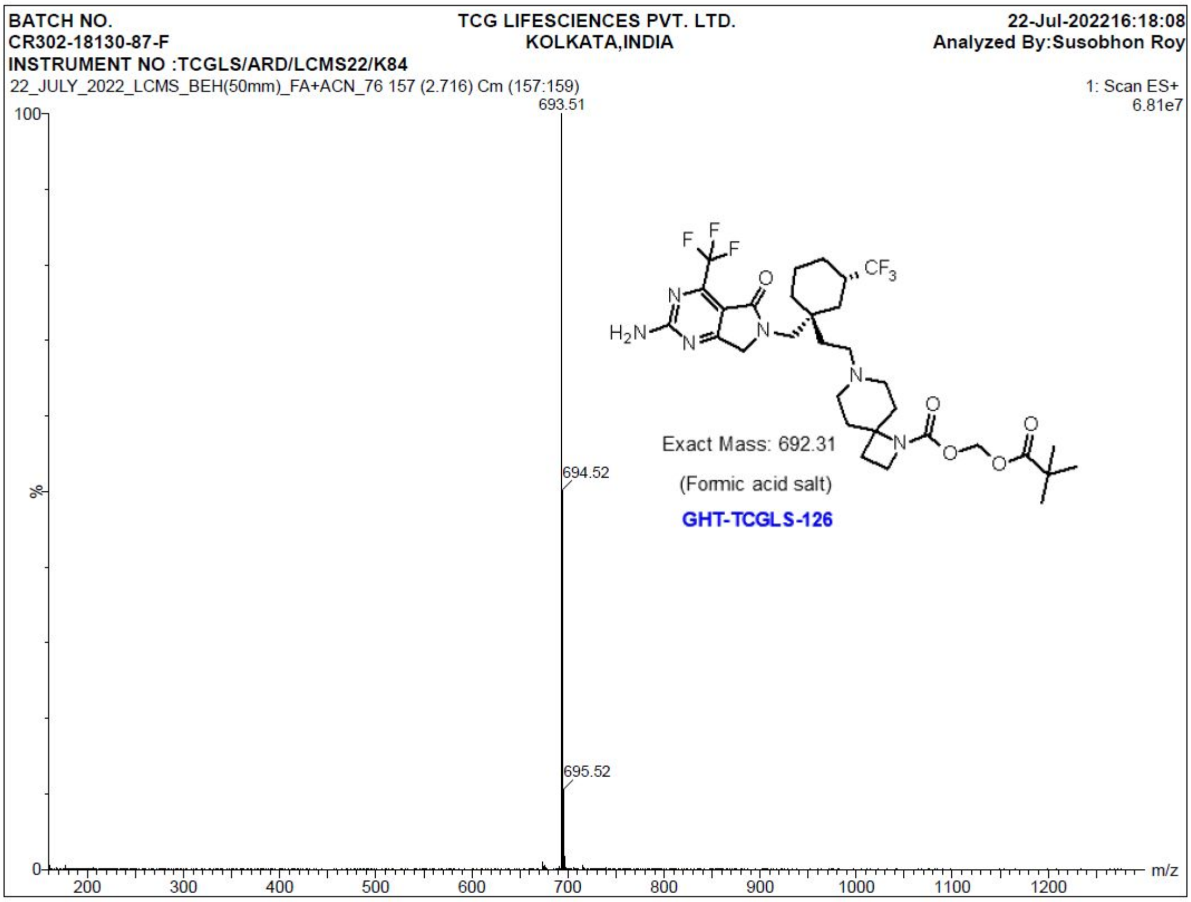
BATCH NO.
CR302-18130-87-F
INSTRUMENT NO :TCGLS/ARD/LCMS22/K84
TCG LIFESCIENCES PVT. LTD.
KOLKATA,INDIA
22-Jul-202216:18:08
Analyzed By:Susobhon Roy
22_JULY_2022_LCMS_BEH(50mm)_FA+ACN_76 157 (2.716) Cm (157:159)
1: Scan ES+
6.81e7
693.51
694.52
695.52
Exact Mass: 692.31
(Formic acid salt)
GHT-TCGLS-126
m/z

## Compound 39

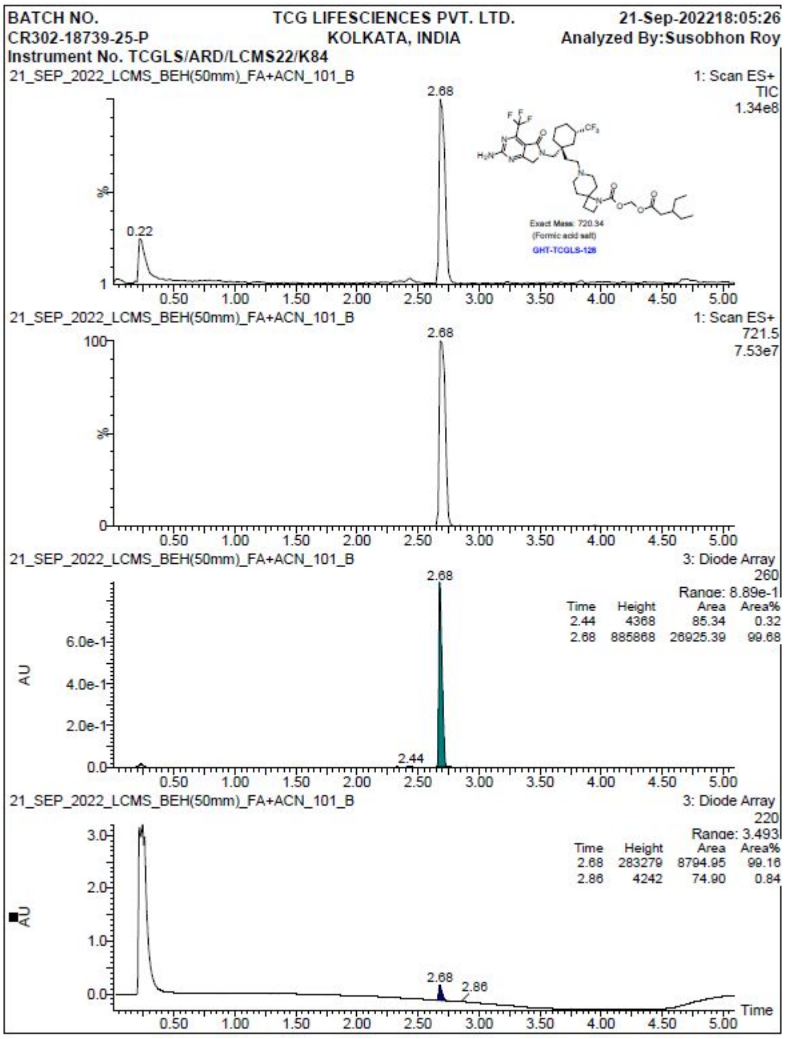
BATCH NO.
CR302-18739-25-P
TCG LIFESCIENCES PVT. LTD.
KOLKATA, INDIA
21-Sep-202218:05:26
Analyzed By:Susobhon Roy
Instrument No. TCGLS/ARD/LCMS22/K84
21_SEP_2022_LCMS_BEH(50mm)_FA+ACN_101_B
1: Scan ES+
TIC
1.34e8
2.68
0.22
Exact Mass: 720.34
(Formic acid salt)
GHT-TCGLS-128
21_SEP_2022_LCMS_BEH(50mm)_FA+ACN_101_B
1: Scan ES+
721.5
7.53e7
2.68
21_SEP_2022_LCMS_BEH(50mm)_FA+ACN_101_B
3: Diode Array
260
Range: 8.89e-1
Time Height Area Area%
2.44 4368 85.34 0.32
2.68 885868 26925.39 99.68
2.68
2.44
21_SEP_2022_LCMS_BEH(50mm)_FA+ACN_101_B
3: Diode Array
220
Range: 3.493
Time Height Area Area%
2.68 283279 8794.95 99.16
2.86 4242 74.90 0.84
2.68
2.86
Time

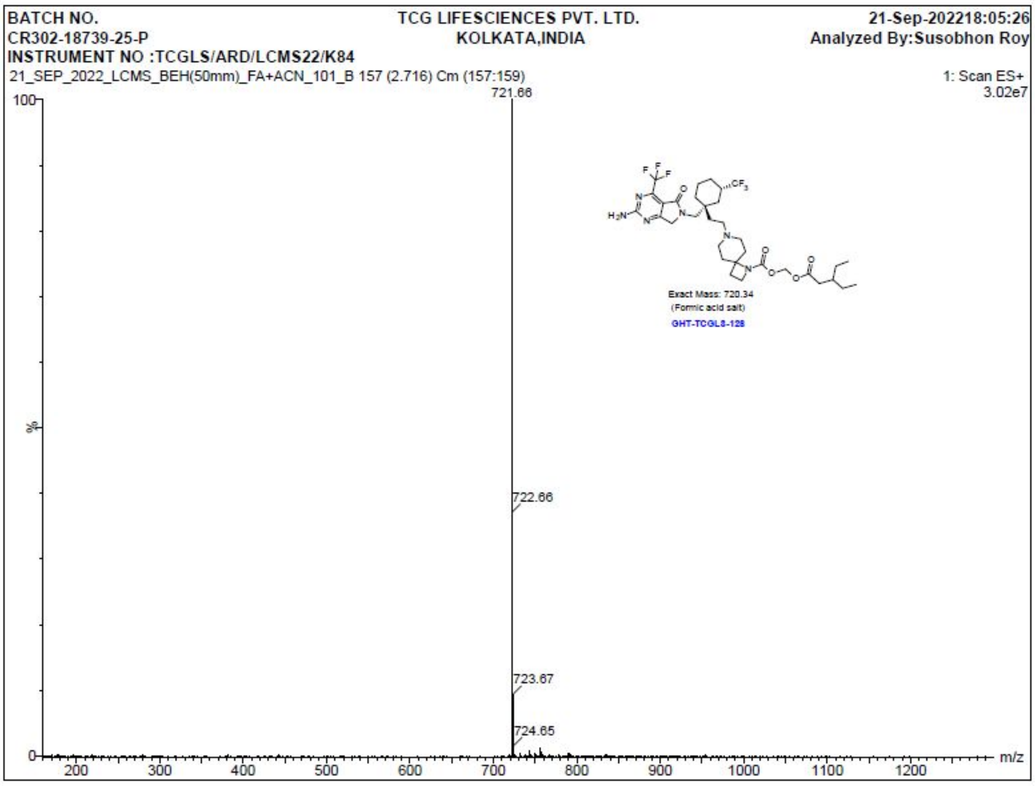
BATCH NO.
CR302-18739-25-P
INSTRUMENT NO :TCGLS/ARD/LCMS22/K84
TCG LIFESCIENCES PVT. LTD.
KOLKATA,INDIA
21-Sep-202218:05:26
Analyzed By:Susobhon Roy
21_SEP_2022_LCMS_BEH(50mm)_FA+ACN_101_B 157 (2.716) Cm (157:159)
1: Scan ES+
3.02e7
721.66
722.66
723.67
724.65
100
%
0
200
300
400
500
600
700
800
900
1000
1100
1200
m/z
Exact Mass: 720.34
(Formic acid salt)
GHT-TCGL8-128

**Compound 40**

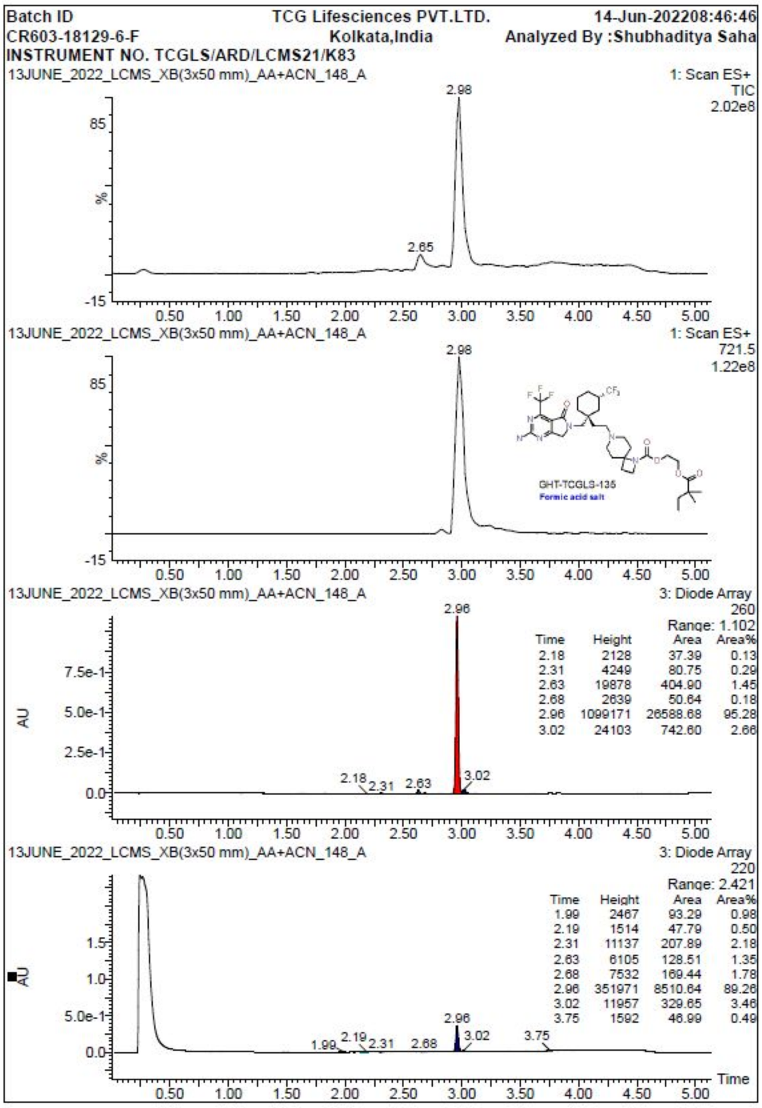

Batch ID
TCG Lifesciences PVT.LTD.
14-Jun-202208:46:46
CR603-18129-6-F
Kolkata,India
Analyzed By :Shubhaditya Saha
INSTRUMENT NO. TCGLS/ARD/LCMS21/K83
13JUNE_2022_LCMS_XB(3x50 mm)_AA+ACN_148_A
1: Scan ES+
TIC
2.02e8
2.98
2.65
1: Scan ES+
721.5
1.22e8
GHT-TCGLS-135
Formic acid salt
3: Diode Array
260
Range: 1.102
Time Height Area Area%
2.18 2128 37.39 0.13
2.31 4249 80.75 0.29
2.63 19878 404.90 1.45
2.68 2639 50.64 0.18
2.96 1099171 26588.68 95.28
3.02 24103 742.60 2.66
3: Diode Array
220
Range: 2.421
Time Height Area Area%
1.99 2467 93.29 0.98
2.19 1514 47.79 0.50
2.31 11137 207.89 2.18
2.63 6105 128.51 1.35
2.68 7532 169.44 1.78
2.96 351971 8510.64 89.26
3.02 11957 329.65 3.46
3.75 1592 46.99 0.49
Time

**BATCH ID:** **TCG Lifesciences PVT. LTD.** **14-Jun-2022**

**CR603-18129-6-F** **Kolkata, India** **08:46:46**

**INSTRUMENT NO. TCGLS\ARD\LCMS21\K83** **Analyzed By :Shubhaditya Saha**

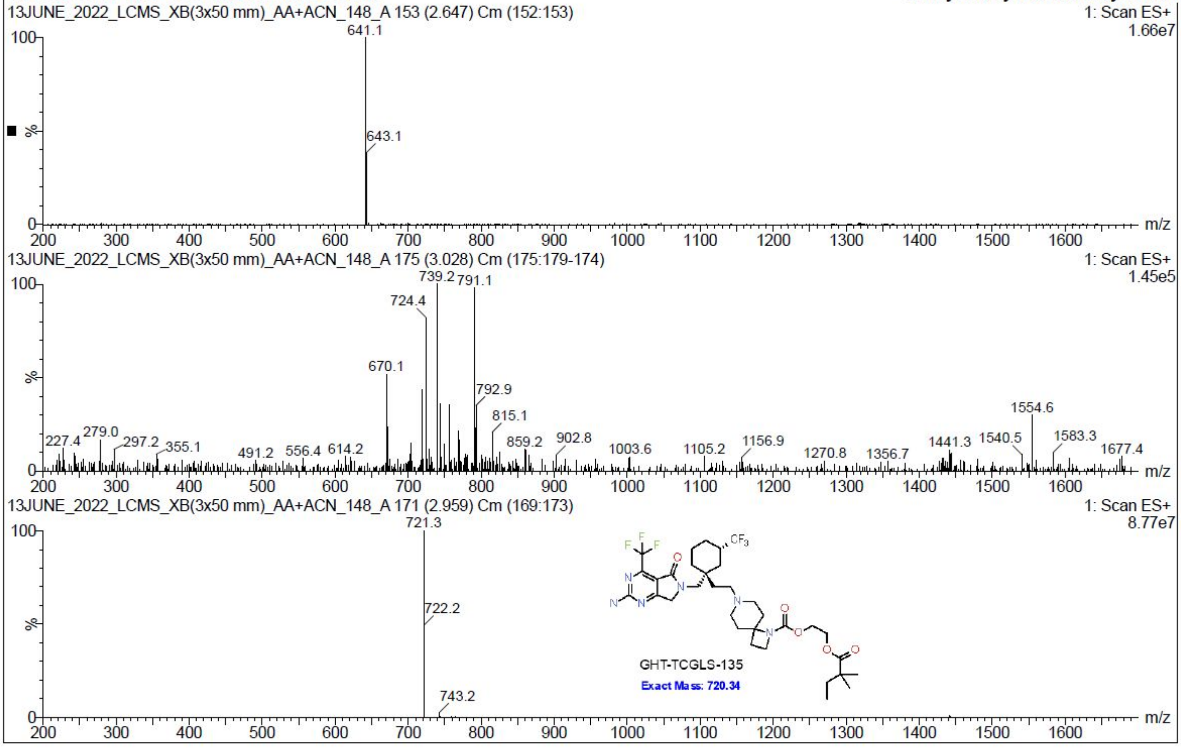

**1HNMR for key compounds**

## Compound 10

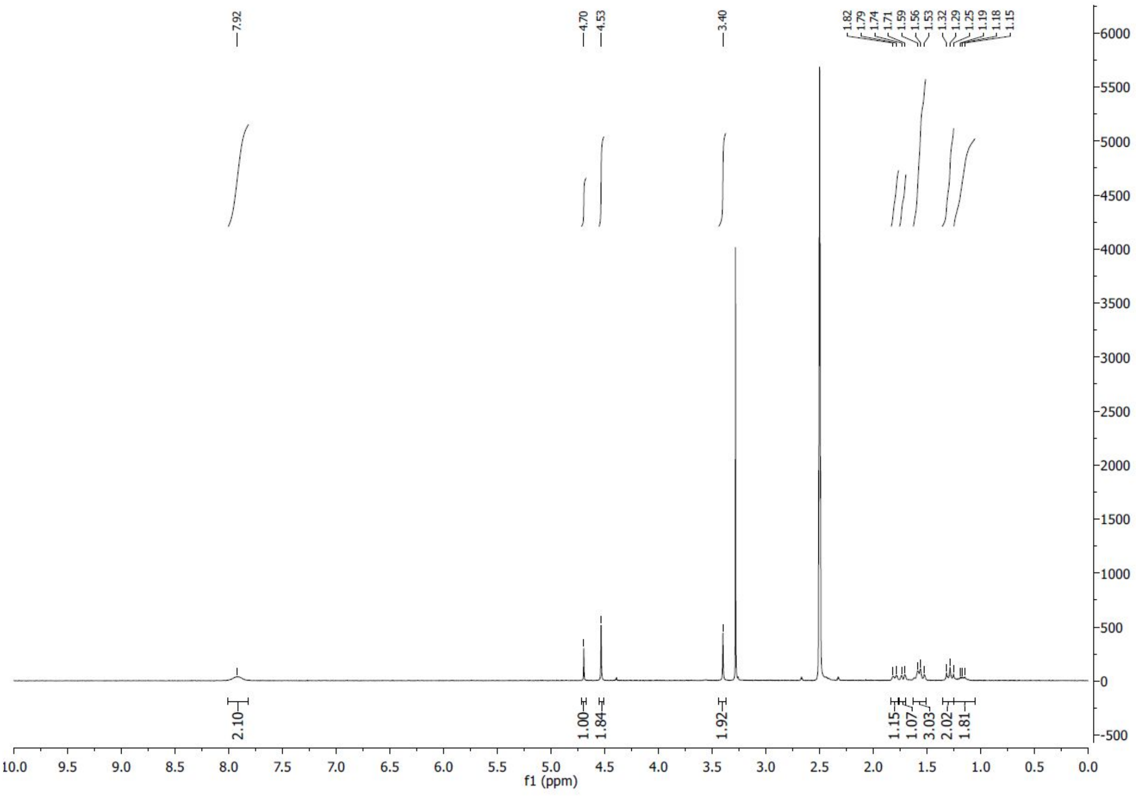

## Compound 16

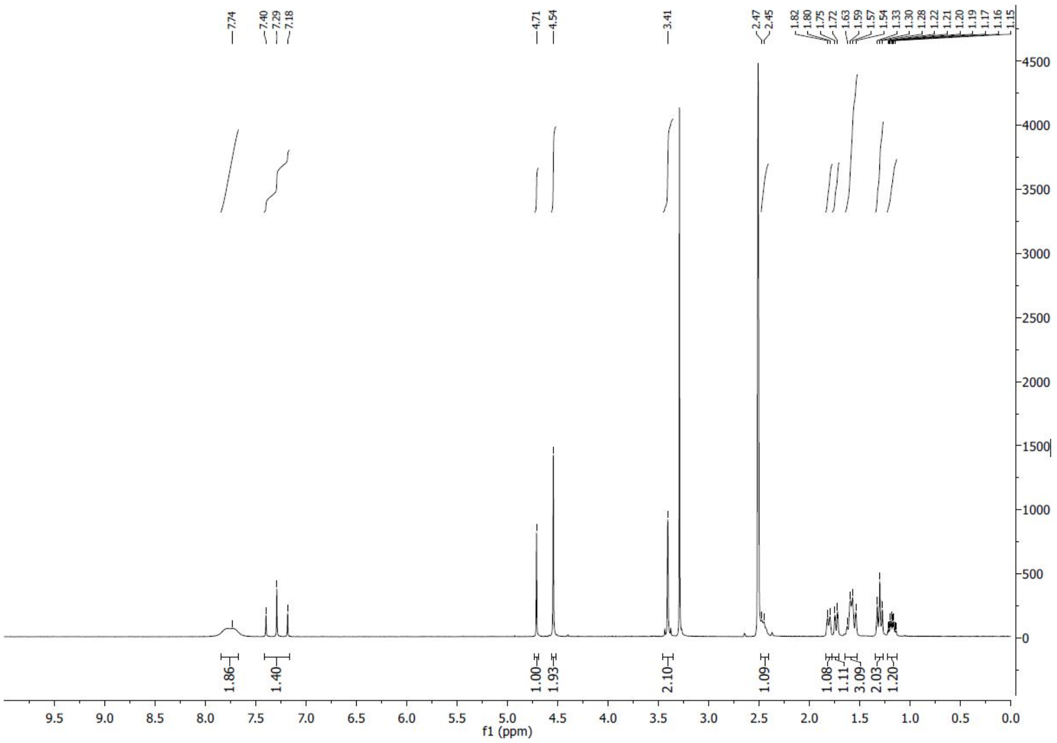

## Compound 18

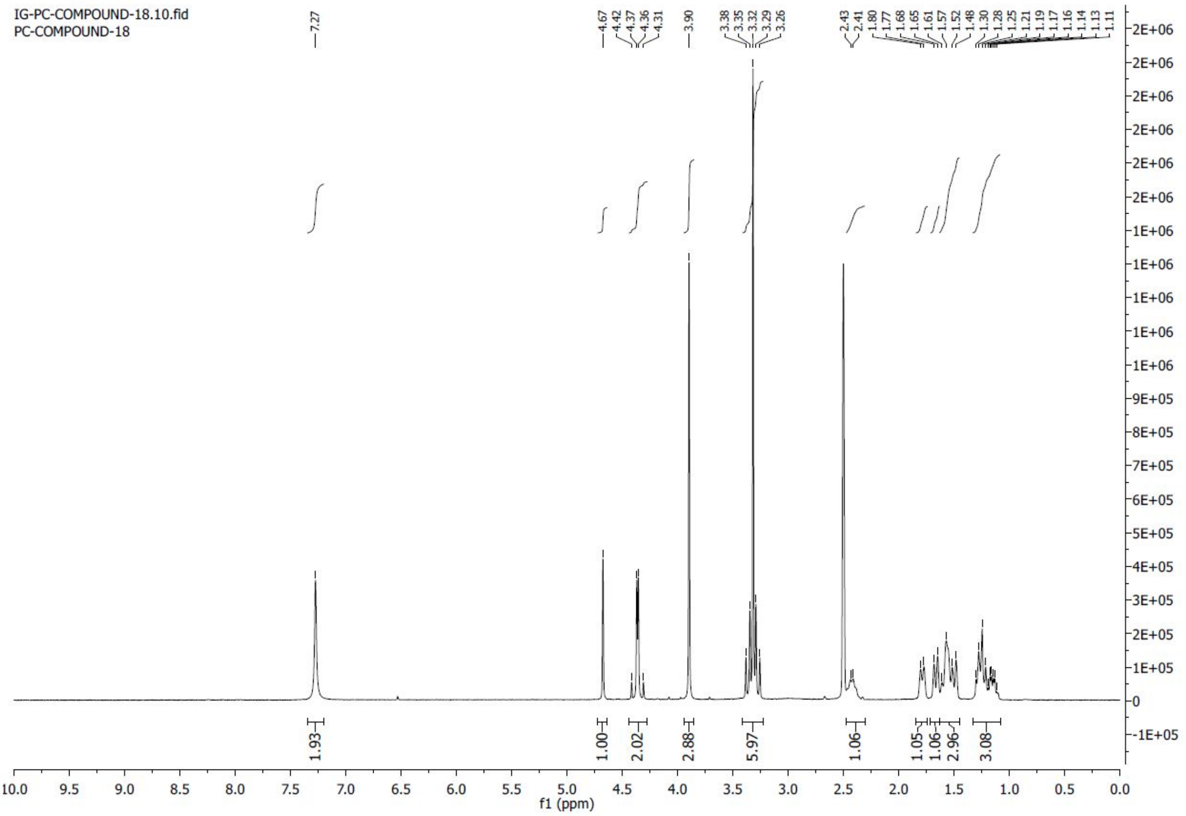


## Compound 24

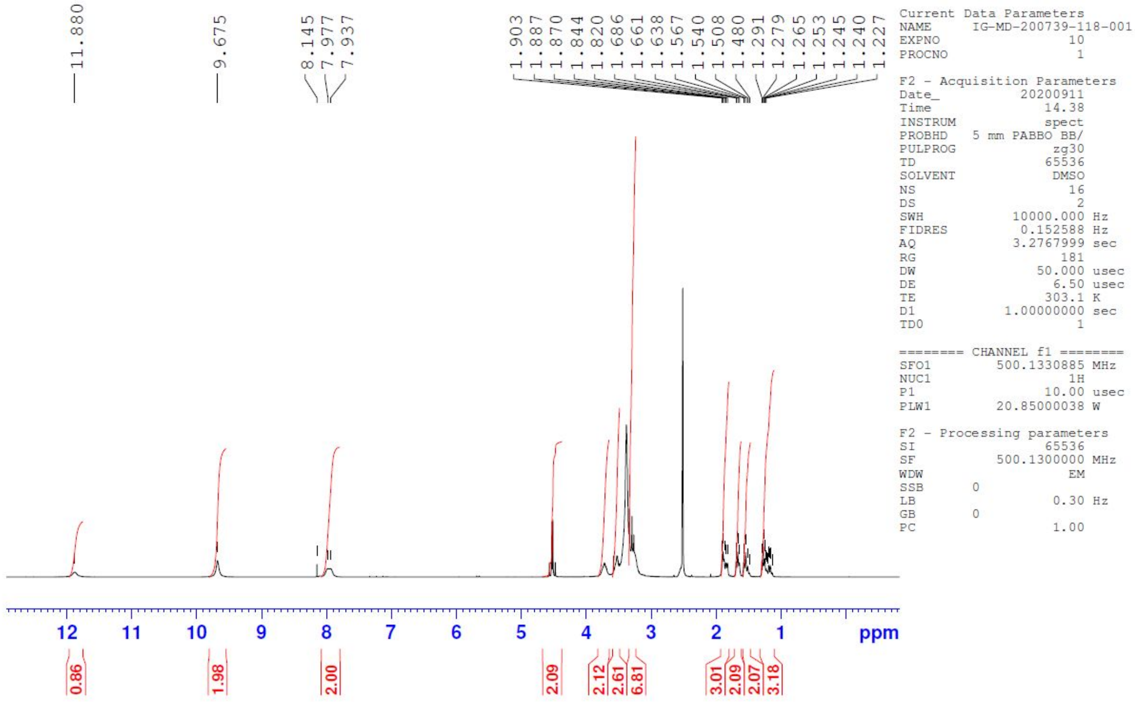


## Compound 25

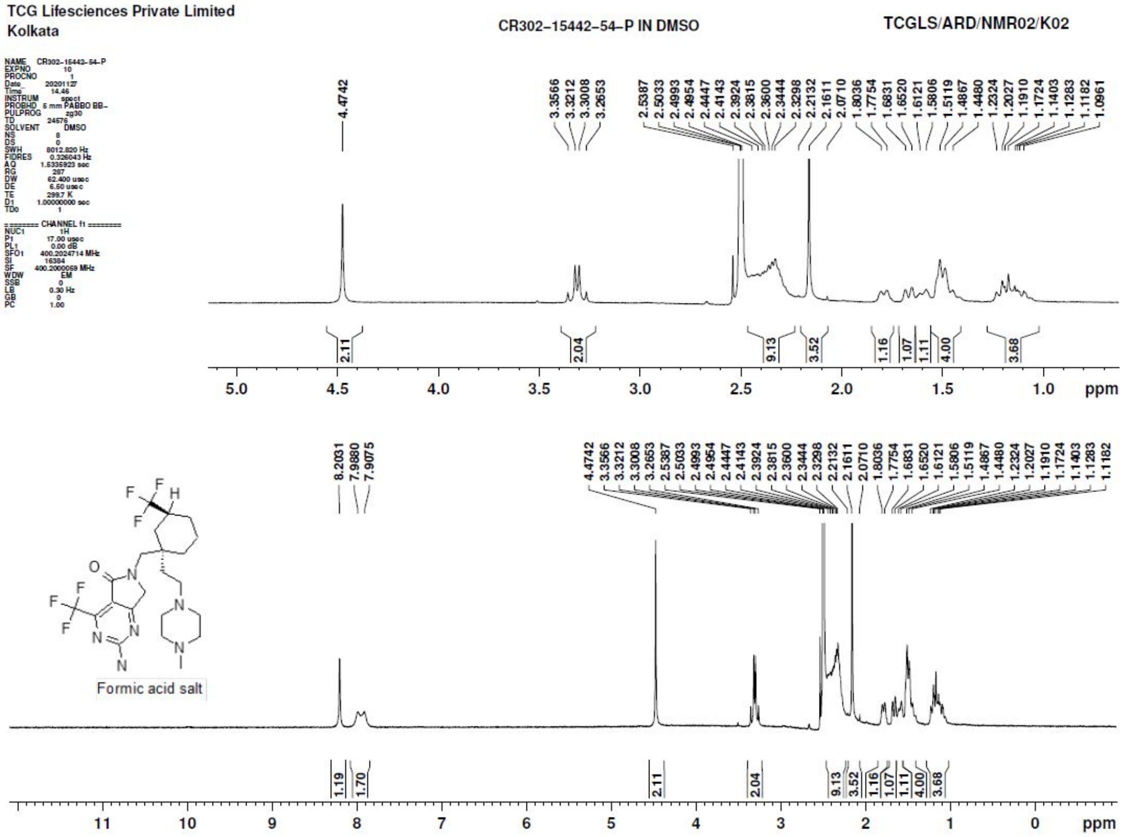
TCG Lifesciences Private Limited
Kolkata
CR302–15442–54–P IN DMSO
TCGLS/ARD/NMR02/K02
Formic acid salt
ppm

## Compound 26

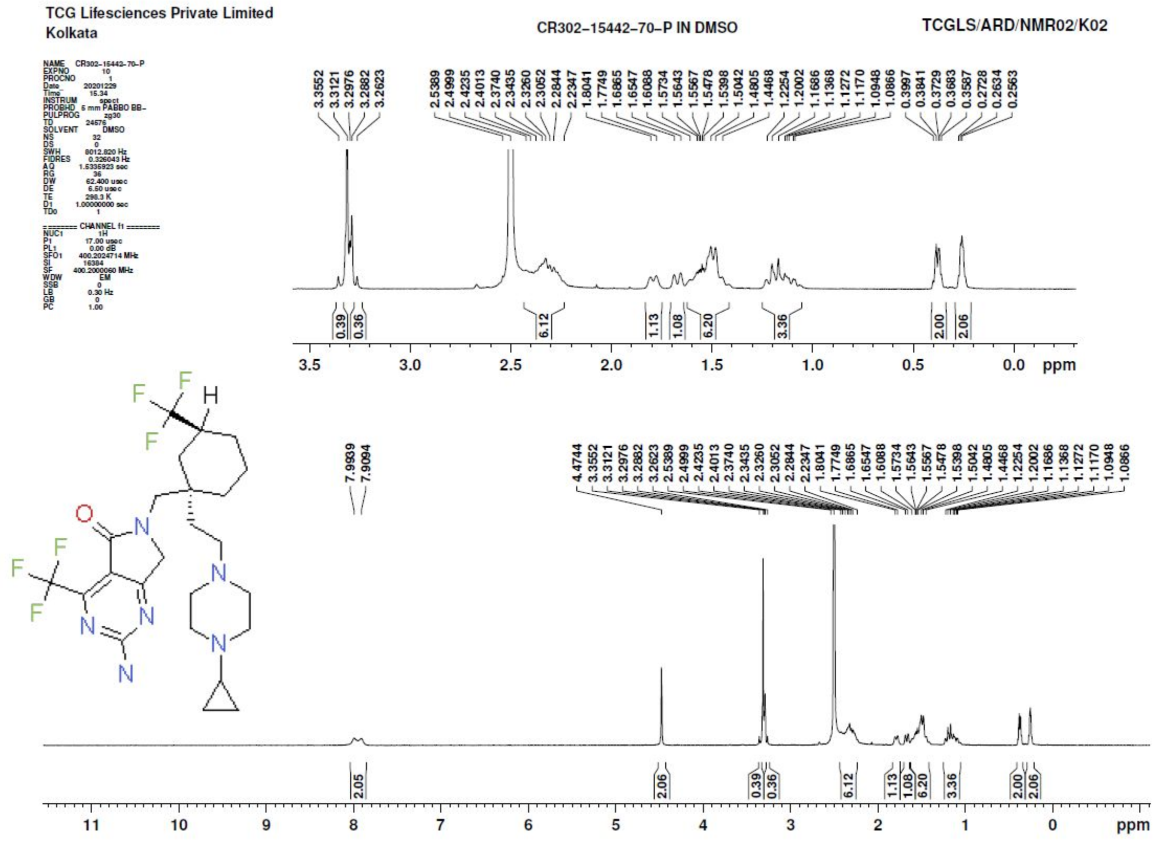
TCG Lifesciences Private Limited
Kolkata
CR302–15442–70–P IN DMSO
TCGLS/ARD/NMR02/K02
ppm

**Compound 27**

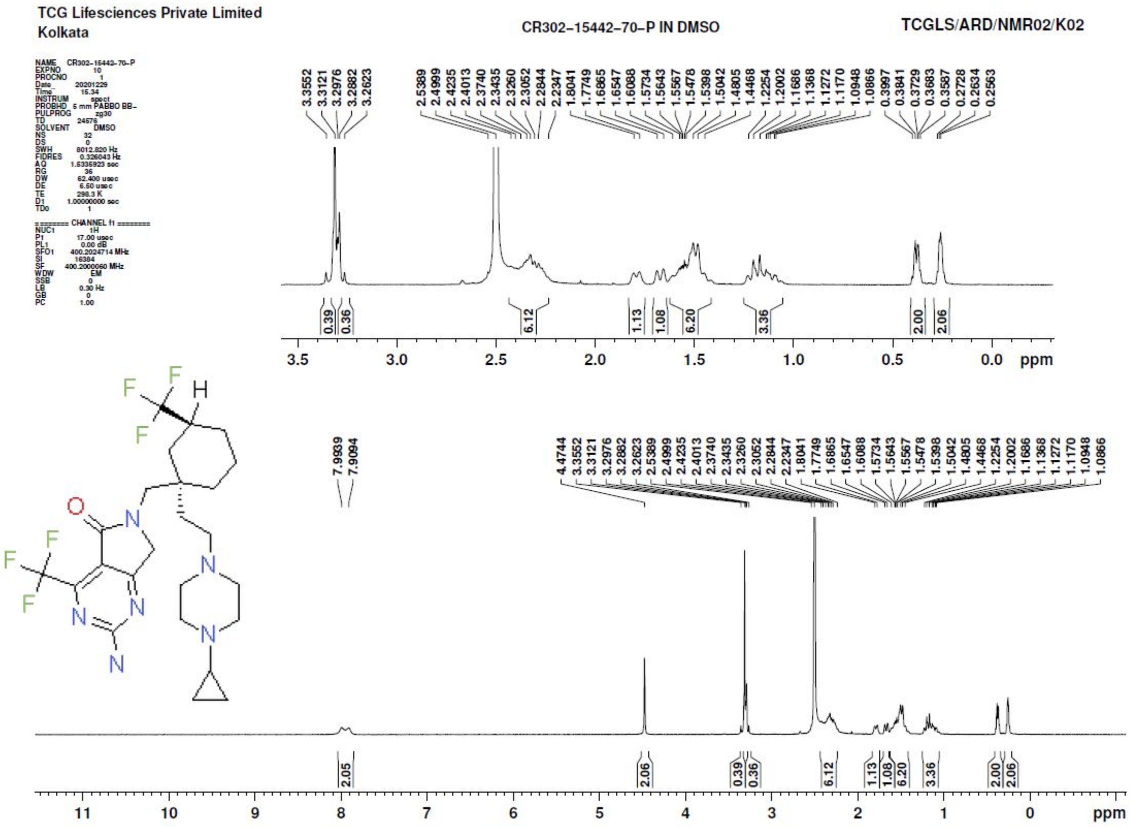


**Compound 28**

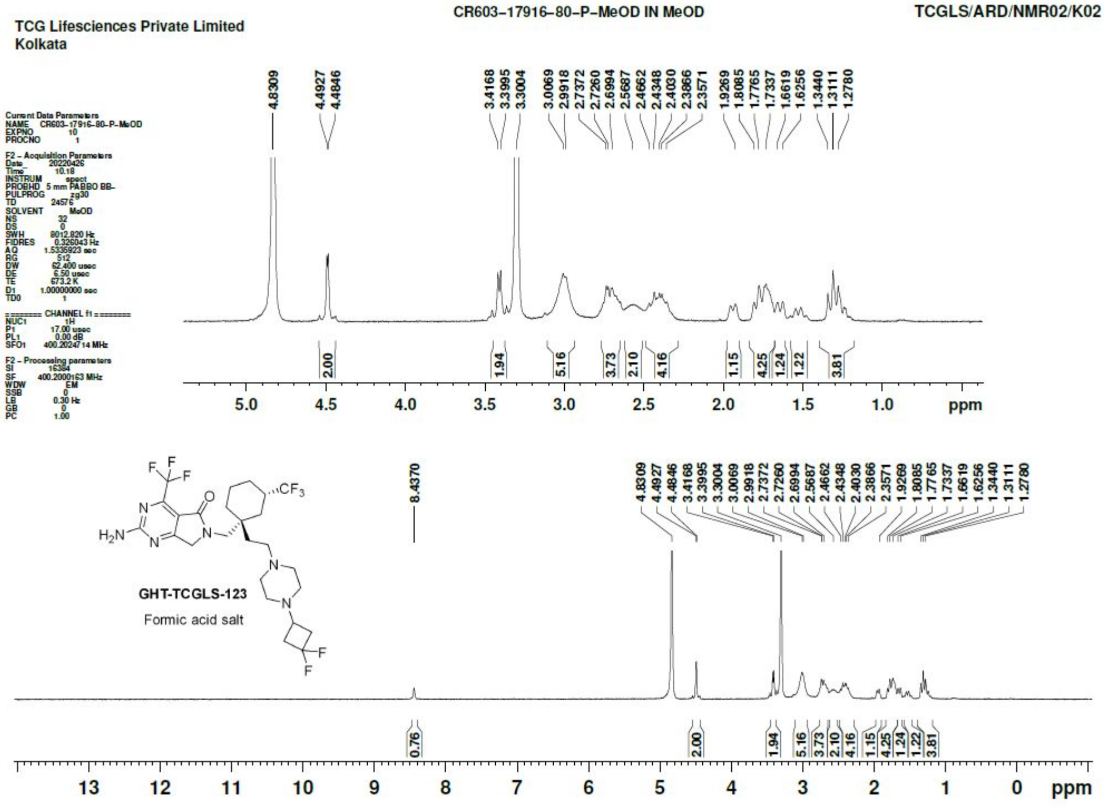

**Compound 30**

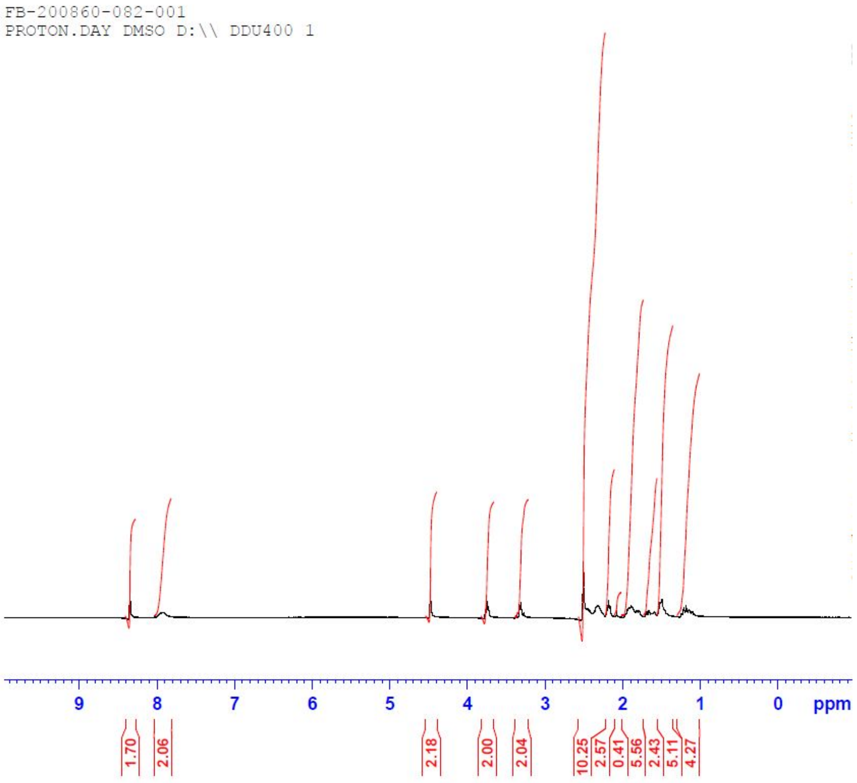

**Compound 32**

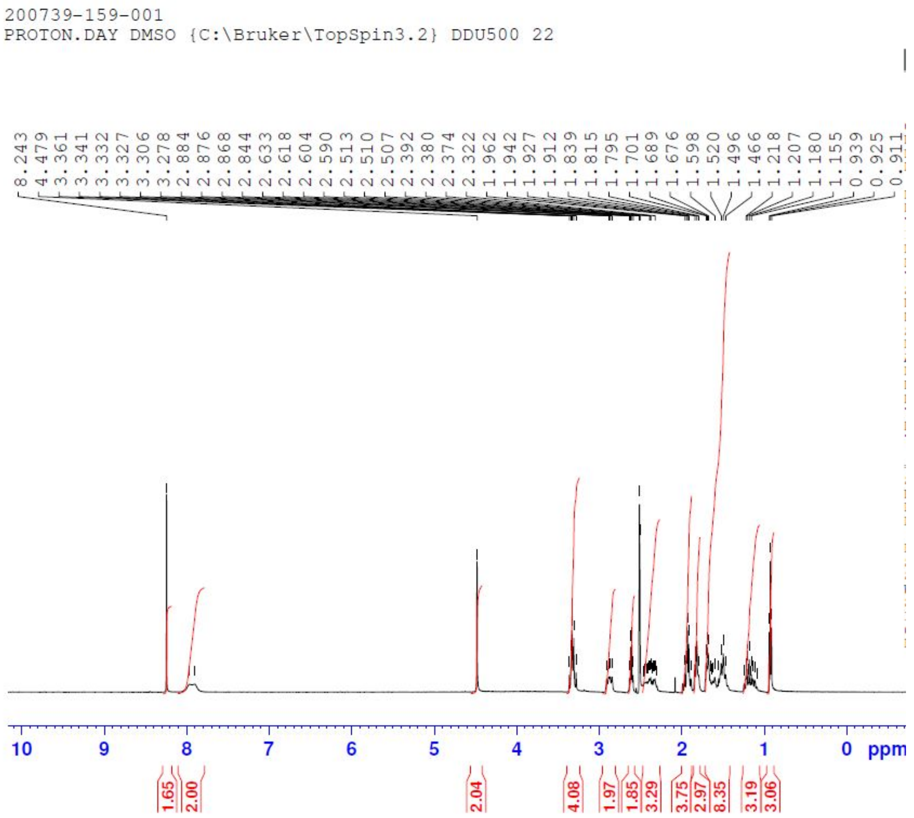
200739-159-001
PROTON.DAY DMSO {C:\Bruker\TopSpin3.2} DDU500 22
10 9 8 7 6 5 4 3 2 1 0 ppm
1.65 2.00 2.04 4.08 1.97 1.85 3.29 3.75 2.97 8.35 3.19 3.06

**Compound 39**

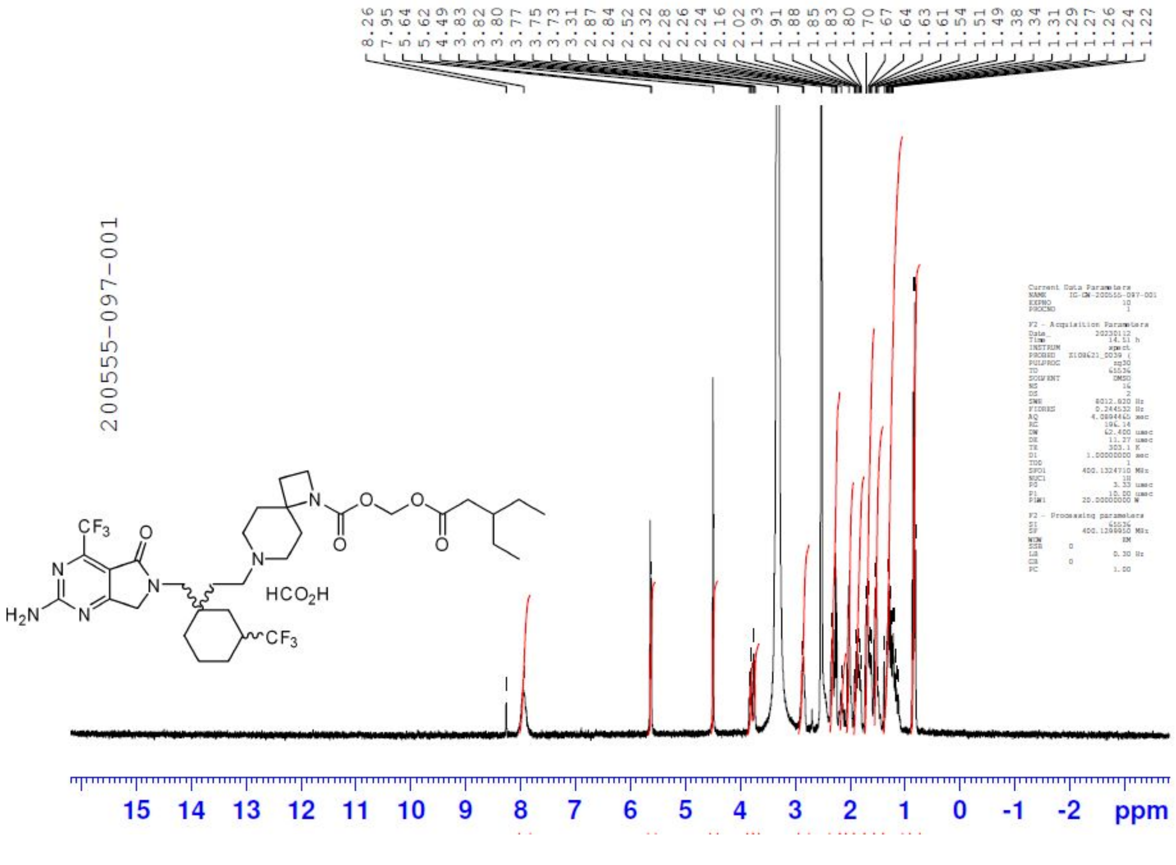
200555-097-001
HCO2H
CF3
15 14 13 12 11 10 9 8 7 6 5 4 3 2 1 0 -1 -2 ppm
